# Evaluating the expression of heat shock protein 27 and topoisomerase II α in a retrospective cohort of patients diagnosed with locally advanced breast cancer and treated with neoadjuvant anthracycline-based chemotherapies

**DOI:** 10.3389/fonc.2023.1067179

**Published:** 2023-08-15

**Authors:** Yixuan Zhuang, Fan Zhang, Yue Xu, Lifang He, Wenhe Huang, Chaoqun Hong, Yukun Cui

**Affiliations:** ^1^ Guangdong Provincial Key Laboratory for Breast Cancer Diagnosis and Treatment, Cancer Hospital of Shantou University Medical College, Shantou, Guangdong, China; ^2^ Department of Pathology, Cancer Hospital of Shantou University Medical College, Shantou, Guangdong, China; ^3^ Oncology Research Laboratory, Cancer Hospital of Shantou University Medical College, Shantou, Guangdong, China; ^4^ Breast Center, Cancer Hospital of Shantou University Medical College, Shantou, Guangdong, China; ^5^ Department of Breast and Thyroid Surgery, Xiang’an Hospital of Xiamen University, Xiamen, Fujian, China

**Keywords:** Hsp27, TopoIIα, locally advanced breast cancer, anthracycline, neoadjuvant chemotherapy

## Abstract

**Background:**

Neoadjuvant anthracycline-based chemotherapy (NAC) is a major regimen for the treatment of local advanced breast cancer (LABC), while resistance to NAC remains a paramount clinical obstacle. To investigate the role of heat shock protein 27 (Hsp27) and/or topoisomerase IIα (TopoIIα) in LABC patients treated with NAC, we performed this retrospective study.

**Methods:**

Associations of Hsp27 transcripts with clinic-pathological characteristics, survival and drug response were investigated in public databases. Hsp27-related genes were identified, followed by functional enrichment analyses. Besides, two protein-protein interaction networks were built. Then, tumors from 103 patients who were diagnosed with LABC and received NAC were collected, and Hsp27 and TopoIIα were examined by Immunohistochemistry (IHC). Chi-square or Fisher’s exact tests were performed, as well as survival analyses.

**Results:**

Either at the transcriptional level in public databases or at the protein level tested by IHC, a high level of Hsp27 was associated with aggressive tumor characteristics such as lymph node invasion and chemotherapy resistance. Hsp27-related genes mostly involved in the metabolic pathway and the gamete generation biological process. An elevated Hsp27 indicated a poor prognosis in patients with breast cancer (log-rank test *P* = 0.002 and 0.004 for disease-free survival [DFS] and overall survival [OS], respectively), while it might not be an independent predictor. Of note, tumors with high TopoIIα expression (TopoIIα+) was less likely to express Hsp27 (Hsp27+), in contrast to those with TopoIIα negativity (31.1% vs. 86.2%, *P*<0.001), and survival analyses revealed that patients with Hsp27+ and TopoIIα- tumors had a significantly lower DFS and OS (log-rank test *P* < 0.001 and 0.001, respectively), in contrast to the other three groups.

**Conclusions:**

Hsp27 was associated with aggressive breast cancers and more predictable for the prognosis of LABC patients treated with NAC when concomitantly considering TopoIIα expression.

## Introduction

Locally advanced breast cancer (LABC) composed around 30% to 40% of all breast cancers in developing countries (1). To reduce the tumor burden for later surgical adjuvant therapy, neoadjuvant chemotherapy (NAC) is commonly used for LABC, while multidrug resistance weakens the therapeutic efficacy and remains the most critical challenge (2, 3) Therefore, identifying patients who may respond to specific treatment regimens before or during the early stage of therapy is essential for avoiding ineffective treatments and achieving better outcome.

Heat shock protein 27 (Hsp27) was reported to overexpress in various cancers, and its overexpression is associated with tumorigenesis, metastasis, and invasiveness (4). Of note, Hsp27 is known for being involved in chemotherapy resistance; related medications include doxorubicin, gemcitabine, erlotinib, 5-fluorouracil, and temozolomide, etc (5). As for breast cancer, Hsp27 was demonstrated to be overexpressed in the aggressive forms, e.g., higher levels in tumors with lymph node metastasis compared to tumors without lymph node metastasis (6), while its value as a prognostic indicator remains arguable (7–9). One early study indicated nuclear Hsp27 was increased after induction chemotherapy (10), that was confirmed later in another study (11). However, this study failed to detect the prognostic value of Hsp27 in LABC patients treated with doxorubicin- or epirubicin-based monochemotherapy (11). Considered of rare evidence specific to LABC and critical choice of NAC to later surgery and outcomes (12), it is worth to further clarity its role in NAC with and without other biomarkers.

DNA topoisomerase IIα (TopoIIα) is one molecular target for the anthracycline class of chemotherapeutic drugs. Overexpression of TopoIIα predicts the responsiveness of breast cancer to anthracycline-based therapy (13). Ectopic overexpression of Hsp27 reduces the expression of TopoIIα in breast cancer cells (14). However, the relationship between Hsp27 and TopoIIα in breast cancer, especially the prognostic value of the combination of Hsp27 and TopoIIα in LABC has not been defined.

In this study, we first worked on public databases and investigated the relation between Hsp27 transcripts and clinic-pathological features, prognosis and chemotherapy resistance. Hsp27-related genes were identified followed by functional enrichment analyses, and two protein-protein interaction networks were built. Then, we detected the expression of Hsp27 and TopoIIα in pretreatment biopsies of LABC patients treated with NAC, and evaluated the association between the expression of Hsp27 and the efficacy of neoadjuvant chemotherapy, and the other clinicopathological parameters. Moreover, we assessed the association between Hsp27 expression and prognosis of LABC, and verified the predictive value of Hsp27 and TopoIIα in combination for the prognostication of LABC patients.

## Materials and methods

### Public databases

#### Associations of Hsp27 and clinic-pathological characteristics in breast cancer

To examine the difference of Hsp27 expression between normal tissues and breast cancers, and the association between Hsp27 and clinic-pathological characteristics namely TNM stage, nodal metastasis and molecular subtypes in TCGA, UALCAN (The University of ALabama at Birmingham Cancer data analysis Portal, https://ualcan.path.uab.edu/index.html, accessed on May 11st, 2023), a database providing easy access to publicly available cancer OMICS data, was utilized (15).

#### Impact of Hsp27 on survival in patients with breast cancer

To identify the prognostic value of Hsp27 in breast cancer, KM Plotter database (http://kmplot.com, accessed on May 11st, 2023), that contained gene expression and survival information from 4929 patients with breast cancer, was employed. According to the median Hsp27 expression at the transcriptional level, patients were divided into two groups of high and low expression, and survival curves related to overall survival (OS), replase-free survival (RFS) and distant metastasis-free survival (DMFS) were created with log-rank test (16). Additionally, PrognoScan, a database collecting publicly available cancer microarray datasets and employing the minimum P-value approach for grouping patients for survival analysis (http://dna00.bio.kyutech.ac.jp/PrognoScan/index.html, accessed on May 13^th^, 2023), was utilized to analyze the correlation between Hsp27 expression and survival in several individual datasets (17).

#### Association between Hsp27 and drug resistence in patients with breast cancer

To examine the relation between Hsp27 at transcriptional level and cancer drug response, CTR-DB (http://ctrdb.ncpsb.org.cn/home, accessed on May 11st, 2023), a database covering 28 tumor types, 123 drugs and 5139 tissue samples and providing drug response information, was employed (18).

#### Identification of Hsp27-related genes and functional enrichment

To identify Hsp27-related genes, Pearson’s correlation coefficients were calculated, and a volcano plot and heat maps were created in LinkedOmics database (http://www.linkedomics.org/login.php, accessed on May 11st, 2023), an online platform for analyzing 32 TCGA cancer-associated multi-dimensional datasets (19). Hsp27-related genes with correlation coefficients greater than 0.3 or less than -0.3 were extracted. Then, ClueGO and EnrichmentMap in NetworkAnalyst 3.0 tool, (accessed on May 11st, 2023) were unilized to perform functional enrichment analyses based on these potentially co-expressed genes (20). Otherwise, the GeneMANIA database (http://www.genemania.org, accessed on May 11st, 2023) was applied to build an interactive network related to Hsp27. A protein-protein interaction (PPI) network was constructed using the STRING online database (https://string-db.org/, accessed on May 11st, 2023), which aims to integrate all known and predicted associations between proteins, including both physical interactions and functional associations.

### Patients and tissue samples

A total of 103 patients diagnosed with LABC who received anthracycline (i.e., doxorubicin or epirubicin)-based NAC (median age: 51 years; range: 28-80 years) in the Cancer Hospital of Shantou University Medical College between December 2008 and September 2013 were included, and we collected the formalin-fixed, paraffin-embedded specimens of both pre-surgery needle core biopsies and post-surgery mastectomized tumors, and clinic-pathological information including age, menopausal state, tumor size (T stage), lymph node status (N stage), clinical TNM stage, histological type, expression of ER, progesterone receptor (PgR), human epidermal growth factor receptor-2 (HER-2) and Ki67. The clinical tumor stage (TNM stage) was defined in accordance with the American Joint Committee on Cancer (AJCC) 6^th^ edition of the Cancer Staging Manual (2002). The molecular classification was grouped in accordance with the 13^th^ St. Gallen International Breast Cancer Conference Expert Panel (2013) (21). ER, PgR, HER-2 and Ki67 scores were evaluated by immunohistochemical (IHC) staining in pre-surgery samples, and Ki67 scores were also detected in post-surgery samples. Immunostaining and scoring ER, PgR, HER-2 and Ki67 were conducted by experienced pathologists during the clinical practice, that followed the standard operating procedure in the pathology department. The HER-2 and Ki67 antibodies were purchased from MXB (KIT-0043 and MAB-0672, Fuzhou, China); ER and PgR were purchased from Roche (790-4325 and 790-4296, Basel, Switzerland). The cut-off value for ER and PgR positivity was set at ≥10% (22). HER-2 was identified positive if IHC staining showed a score of 3, or if fluorescence *in situ* hybridization (FISH) showed >2.2-fold amplification of the HER-2 gene in tumors, and the FISH test was only performed for tumors that were scored at 2 in IHC staining (23). A cut-off of Ki67 to distinguish luminal A from luminal B was 14% (24). Prognosis was determined by annual phone interview and medical records. Informed consent for the use of their samples was obtained from all patients. This study was approved by the medical ethics committee of the Cancer Hospital of Shantou University Medical College (No. 201733).

### Immunohistochemistry assay

IHC for Hsp27 and TopoIIα were carried out using a standard Envision complex method (25). Briefly, needle core biopsies were fixed in 10% buffered formalin and embedded in paraffin. After deparaffinization and rehydration, endogenous peroxidase activity was blocked with 3% hydrogen peroxide for 10 min. Then antigen retrieval was performed by heating the slides in a microwave oven for 20 min in an EDTA buffer (pH 8.0). The dilution of Hsp27 and TopoIIα primary antibodies (ab2790, ab52934, Abcam, Cambridge, UK) was 1:400 and 1:200, respectively. IHC staining was carried out by a GTVision antibody complex (anti-mouse/rabbit) method using a GTVision™ Detection Kit (GK5006, Gene Tech, Shanghai, China) and 3,3’-diaminobenzidine (DAB) as the chromogen substrate. A negative control was obtained by replacing the primary antibody with mouse IgG1-kappa monoclonal antibody (ab18443, Abcam, Cambridge, UK).

IHC staining for Hsp27 was scored, as described by a combination of intensity (0, no staining; 1, weak staining; 2, moderate staining; 3, strong staining) and proportion (0, < 5% of tumor cells stained; 1, 5-25% positive cells; 2, 26-50% positive cells; 3, 51-75% positive cells; 4, more than 76% positive cells) scores (26). If the multiplication product between staining intensity and the proportion of positive cells was >4 (the median multiplication value), expression was defined as Hsp27-positive; otherwise, samples were regarded as Hsp27-negative. TopoIIα status was scored for positivity in four random fields (×400 magnification) to get the total percent-score. Then, the median values of TopoIIα were chosen as cut-offs for high or low expression (27). Two pathologists independently assessed the cellular location and immunostaining intensity, and a third observer determined if any discrepancies happened.

### Statistical analysis

All analyses were performed in SPSS (version 13.0). Associations of Hsp27 expression with TopoIIα and clinicopathological features were analyzed using the chi-square test or Fisher’s exact test. Disease-free survival (DFS) in months was defined as the period from surgery to the date of relapse or metastasis or last contact, which came first. OS in months was defined as the period from surgery to the date of last contact or death from any cause, which came first. Kaplan-Meier curves were created according to Hsp27 expressions and tested by log-rank test. Univariate and Multivariate Cox regressions were used to study the effects of Hsp27 expression on DFS and OS. *P* < 0.05 was considered to indicate statistical significance, and all resulting *P*-values were two-tailed.

## Results

### Associations between Hsp27 at the transcriptional level and clinic-pathological features in breast cancer

**Figure 1:**
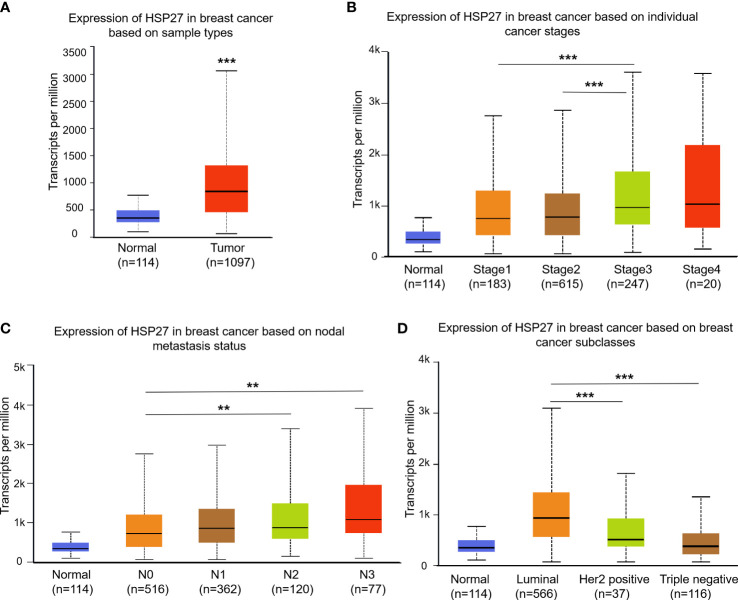
Box plots show Hsp27 expression among different clinical parameters using the UALCAN database. Difference of Hsp27 expression was separately evaluated for **(A)** sample types, **(B)** cancer stages, **(C)** nodal metastasis status and **(D)** breast cancer subclasses. N0: no regional lymph node metastasis; N1: metastases in 1 to 3 axillary lymph nodes; N2: metastases in 4 to 9 axillary lymph nodes; N3: metastases in 10 or more axillary lymph nodes. **P<0.01, ***P<0.001.

According to UALCAN database, there was a higher Hsp27 expression in breast cancers compared with normal group (**Figure 1A**), and an elevation of Hsp27 was in tumors at Stage 3 compared to those Stage 1 and 2 (**Figure 1B**). A fail of statistical significance at Stage 4 might be related to a small sample size. In term of nodal metastasis status, an increase of Hsp27 expression was seen along with increased numbers of nodes with metastasis (**Figure 1C**). Regarding molecular subtypes, Hsp27 expression was the highest in luminal breast cancer, followed by that in Her2-positive types and TNBC (**Figure 1D**). Moreover, according to CTR-DB, a high expression level of Hsp27 significantly indicated resistance to TFAC (Cyclophosphamide, Doxorubicin, fluorouracil, paclitaxel) and epirubicin, and the area under the AUC curve was greater than 0.7 (**Supplementary Figure 1**). In short, these findings suggest that Hsp27 mRNA levels were related to an aggressive character and drug resistance in patients with breast cancer.

### Prognostic values of Hsp27 at the transcriptional level in breast cancer

**Figure 2:**
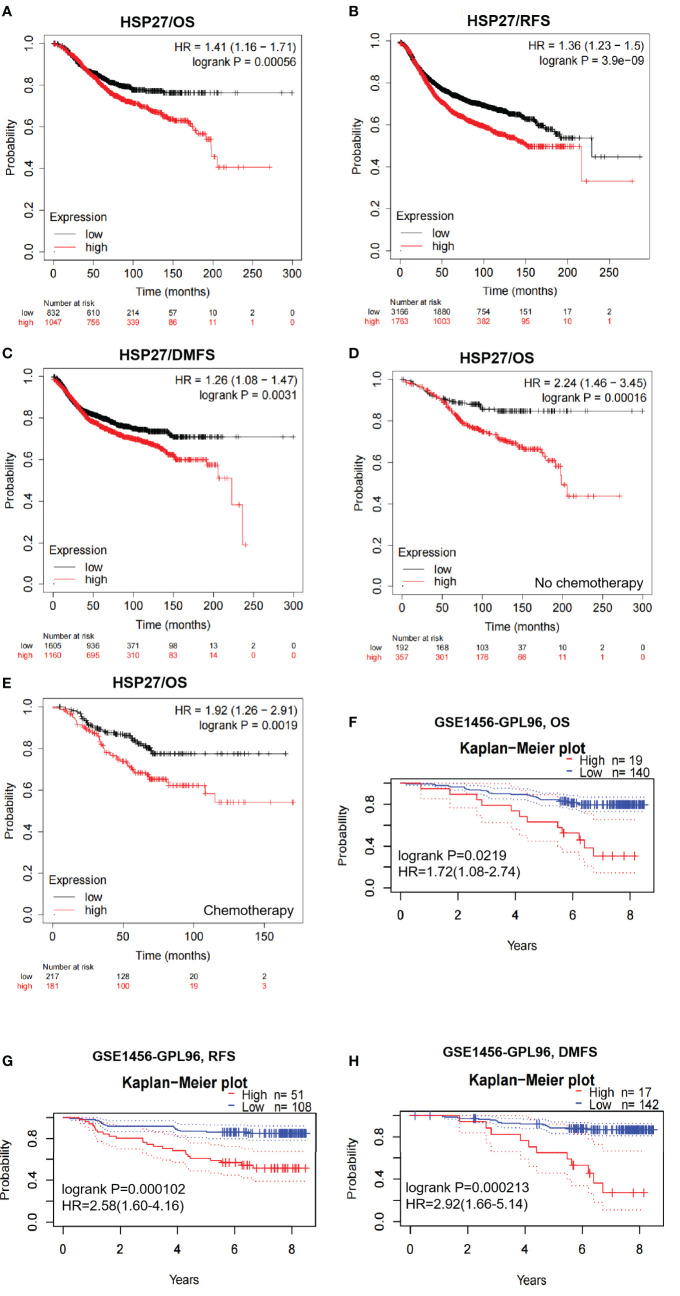
Survival curves were created to evaluate the prognostic value of Hsp27 in breast cancer. **(A–C)** OS, RFS and DMFS curves were created despite chemotherapy in the Kaplan Meier plotter database. **(D)** OS curves were created for patients without chemotherapy in the Kaplan Meier plotter database. **(E)** OS curves were created for patients receiving chemotherapy in the Kaplan Meier plotter database. **(F–H)** OS, RFS and DMFS curves were created using the PrognoScan database (dataset: GSE1456). All dotted lines represented confidence bands.

According to Kaplan-Meier plotter database, elevation of Hsp27 at the transcriptional level was associated significantly with a poor prognosis in breast cancer (HRs, 95% CIs for OS: 1.41, 1.16-1.71, *P*=5.6e-04; RFS: 1.36, 1.23-1.5, *P*=3.9e-09; DMFS: 1.26, 1.08-1.47, *P*=0.0031. **Figures 2A–C**). Similar findings were seen when patients were separately restricted to those who did not receive chemotherapy (**Figure 2D**) and those who received chemotherapy (**Figure 2E**). Regarding individual datasets, consistent findings remained using PrognoScan database (**Figures 2F–H**; **Supplementary Figure 2**). All these results demonstrate that Hsp27 expression might be correlated with a poor outcome in patients with breast cancer.

### Hsp27-related genes and functions in breast cancer

**Figure 3:**
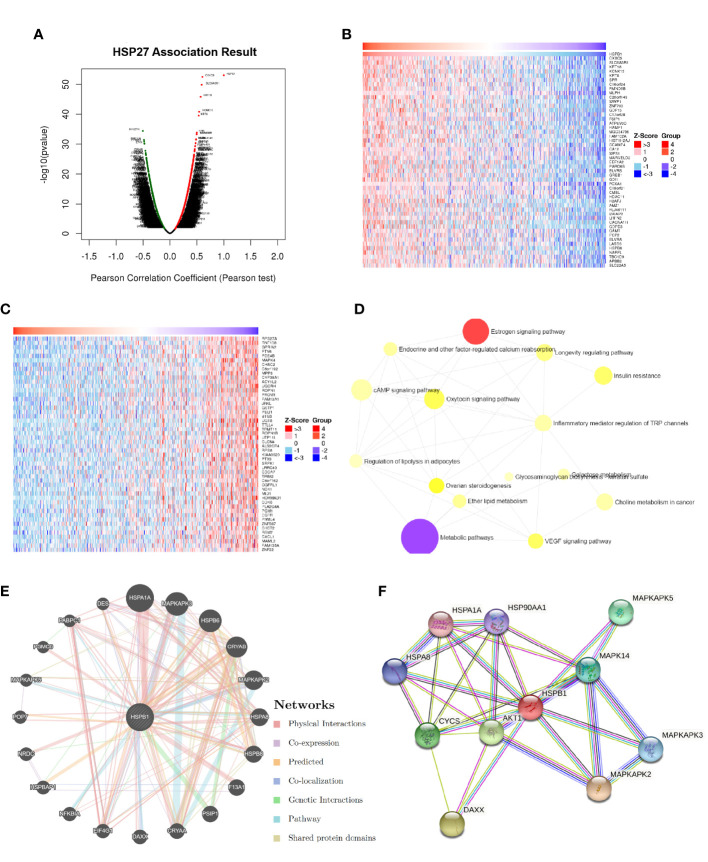
Hsp27-relevant genes and functions were estimated in breast cancer. **(A)** The global Hsp27 highly correlated genes were identified by Pearson test in the LinkedOmics database (dataset: TCGA). Heat maps separately showed the top-ranked 50 genes positively **(B)** and negatively **(C)** correlated with Hsp27 in breast cancer. **(D)** Functional enrichment analyses based on KEGG pathway were performed for Hsp27 related genes. **(E)** The gene-gene interaction network for Hsp27 (i.e., HSPB1) was constructed using GeneMANIA. **(F)** The PPI network of Hsp27 was generated using STRING.

According to LinkedOmics database, a total of 3079 genes and 3072 genes were positively and negatively correlated with Hsp27, respectively (false positive rate, FDR<0.05, **Figure 3A**). Ranking by absolute values of pearson correlation coefficients, the top 50 genes positively and negatively related to Hsp27 were separately shown in **Figures 3B, C**. An overall description of co-expressed genes is detailed in **Supplementary Table 1**. Enrichment analyses of KEGG pathway based on potentially co-expressed genes indicated related pathways such as “cAMP signaling pathway”, “metabolic pathways” and “Oxytocin signaling pathway” (**Supplementary Table 2**; **Figure 3D**). Enrichment analyses based on GO database showed that the related biological processes were gamete generation, anion transport and sexual reproduction (**Supplementary Table 3**), and the related cellular components were cytosol, vesicle and cytoplasmic vesicle, and the related molecular functions were purine nucleotide binding, purine ribonucleotide binding (**Supplementary Tables 4, 5**). Further, we used GeneMANIA to construct a gene-gene interaction network, that showed twenty genes with the highest altered frequencies related to Hsp27, including MAPKAPK2, MAPKAPK3, MAPKAPK5 (**Figure 3E**). Consistently, these genes were mostly observed in a protein-protein interaction (PPI) network generated on the STRING database (**Figure 3F**). These findings suggest that Hsp27 was related to biological metabolism, and mitogen-activated protein kinases.

### Associations of Hsp27 proteins with clinicopathological features, TopoIIα and therapeutic efficacy in patients with LABC

**Figure 4:**
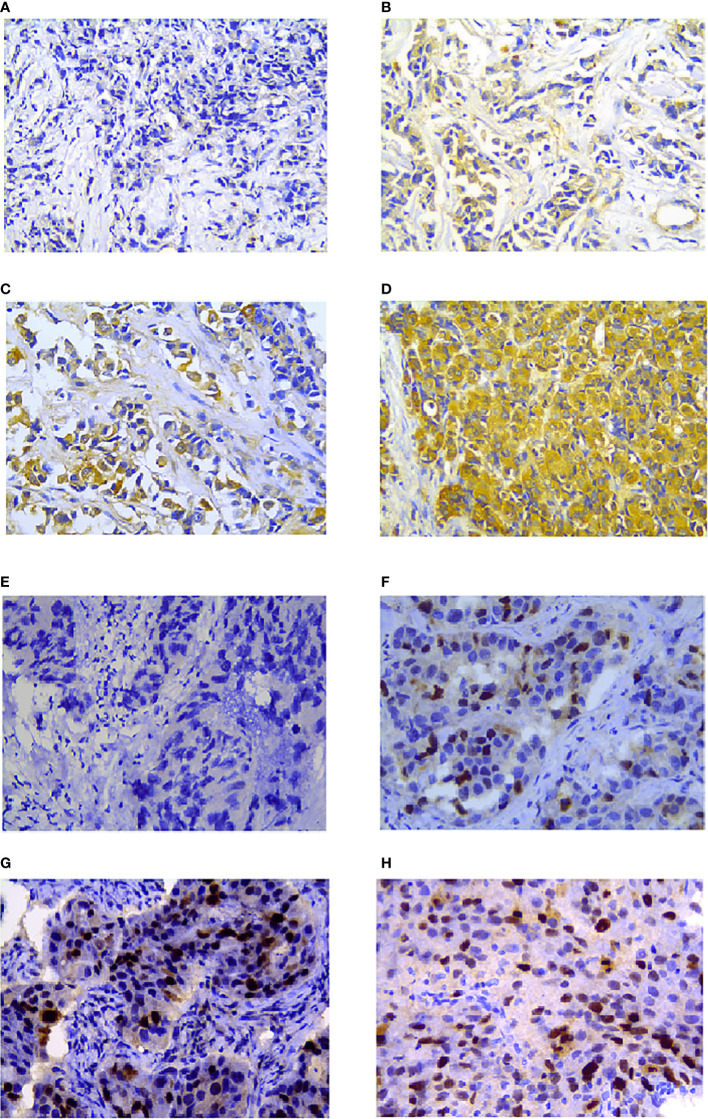
Hsp27 and TopoIIα proteins were detected by IHC in breast cancers. Tumor tissues were separately stained **(A)** negative, **(B)** week, **(C)** moderate, **(D)** strong for Hsp27, and **(E)** negative, **(F)** week, **(G)** moderate, **(H)** strong for TopoIIα. The magnification was 400×.

Hsp27 and TopoIIα proteins were detected in tumors by IHC assays (**Figure 4**). As shown in **Table 1**, out of 103 LABC patients, 48 (46.6%) were Hsp27-positive, Hsp27 (+), while 55 (53.4%) patients were Hsp27-negative, Hsp27 (-). The percentage of Hsp27 (+) tumors comprised 22.2% of the N1 group (4/18) and 51.8% of the N2-N3 group (44/85), leading to a significant association between Hsp27 and lymph node status (*P* = 0.022). Besides, Hsp27 was related to histological grade (*P*< 0.001); there was only 17.1% of well or moderately differentiated tumors (7/48), but 66.1% of poorly differentiated tumors (41/62) being detected Hsp27 (+). Additionally, Hsp27 (-) tumors showed a percentage of 60.3% (47/78) to have NAC-reduced Ki67, but only a percentage of 32.0% to have NAC-elevated Ki67 (*P*=0.014). These findings again confirm the aggressive role of Hsp27 in breast cancer and its role involving in chemotherapeutic resistance. No significant differences were found for age, menopausal status, TNM stage, ER status, PgR status, HER-2 status and molecular subtypes.

**Table 1:** Relationship of Hsp27 expression with clinicopathological features,TopoIIα and therapeutic effect of anthracycline-based NAC in LABC patients.

Terms	N (%)	Hsp27 expression (%)		Chi-square	*P*
		Negative (n=55)	Positive (*n*=48)		
Clinicopathological features					
Age					
≤50	47 (45.6)	25 (53.2)	22 (46.8)	0.001	0.969
>50	56 (54.4)	30 (53.6)	26 (46.4)		
Menopausal status					
Pre	52 (50.5)	26 (50.0)	26 (50.0)	0.487	0.485
Post	51 (49.5)	29 (56.9)	22 (43.1)		
Nodal status					
N1	18 (17.5)	14 (77.8)	4 (22.2)	5.210	*0.022*
N2-N3	85 (82.5)	41 (48.2)	44 (51.8)		
TNM stage					
TNM2-TNM3a	34 (33.0)	19 (55.9)	15 (44.1)	0.126	0.723
TNM3b-TNM3c	69 (67.0)	36 (52.2)	33 (47.8)		
Histological grade					
well/moderately differentiated	41 (39.8)	34 (82.9)	7 (17.1)	23.867	*<0.001*
poorly differentiated	62 (60.2)	21 (33.9)	41 (66.1)		
Ki67 change (after chemotherapy)					
Reduced	78 (75.7)	47 (60.3)	31 (39.7)	6.074	*0.014*
Elevated	25 (24.3)	8 (32.0)	17 (68.0)		
ER					
Negative	38 (36.9)	18 (47.4)	20 (52.6)	0.880	0.348
Positive	65 (63.1)	37 (56.9)	28 (43.1)		
PR					
Negative	47 (45.6)	23 (48.9)	24 (51.1)	0.692	0.406
Positive	56 (54.4)	32 (57.1)	24 (42.9)		
Her-2					
Negative	66 (64.1)	34 (51.5)	32 (48.5)	0.262	0.609
Positive	37 (35.9)	21 (56.8)	16 (43.2)		
Molecular subtype					
Luminal A	8 (7.7)	6 (75.0)	2 (25.0)	–	0.317*
Luminal B1	45 (43.7)	22 (48.9)	23 (51.1)		
Luminal B2	14 (13.6)	10 (71.4)	4 (28.6)		
TNBC**	13 (12.6)	5 (38.5)	8 (61.5)		
HER2-enriched	23 (22.3)	12 (52.2)	11 (47.8)		
TopoIIα					
Negative	29 (28.2)	4 (13.8)	25 (86.2)	25.443	*<0.001*
Positive	74 (71.8)	51 (68.9)	23 (31.1)		
Therapeutic effectiveness					
Chemotherapeutic efficacy					
CR+PR	79 (76.7)	47 (59.5)	32 (40.5)	5.062	*0.024*
SD+PD	24 (23.3)	8 (33.3)	16 (66.7)		
Pathologic complete response					
yes	11 (10.7)	11 (100.0)	0 (0.0)	10.748	*0.001*
no	92 (89.3)	44 (47.8)	48 (52.2)		

Statistically significant values (P< 0.05) are given in italics.* Fisher’s Exact Test. ** Triple-Negative Breast Cancer.

As shown in **Table 1**, 74 out of 103 tumors (71.8%) displayed TopoIIα-positive, among which 23 (23/74, 31.1%) were Hsp27 (+) tumors. However, 25 out of 29 TopoIIα-negative tumors displayed Hsp27 (+) (25/29, 86.2%), demonstrating a negative correlation between TopoIIα and Hsp27. Additionally, compared with patients who showed complete response (CR) and partial response (PR) to chemotherapy, patients with stable or progressive disease had a higher Hsp27 expression (32/79, 40.5% vs. 16/24, 66.7%, *P* = 0.024). All patients (11/11, 100%, *P* = 0.001) in the Hsp27 (-) group showed pathologic complete response (pCR) following NAC treatment.

### Effect of Hsp27 expression on DFS and OS

**Figure 5:**
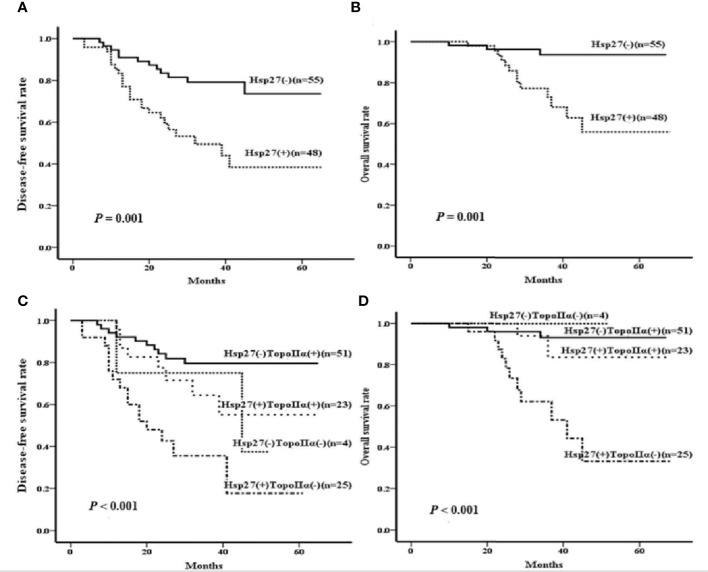
Kaplan-Meier curves were created to examine the associations of Hsp27 and TopoIIα proteins with DFS or OS. According to the expression of Hsp27, survival curves were separately created to compare **(A)** disease-free survival rate and **(B)** overall survival rate. According the expression of Hsp27 and TopoIIa in combination, survival curves were separately created to compare **(C)** disease-free survival rate and **(D)** overall survival rate.

In terms of DFS, the follow-up period ranged from 3 to 65 months (median: 31 months). Patients with Hsp27(+) tumors tended to have a shorter DFS than those with Hsp27 (-) tumors (P = 0.001, **Figure 5A**), consistent with the result of the univariate Cox regression (HR: 3.028, 95%CI: 1.506-6.086, P= 0.002, **Table 2**). Conversely, a shorter DFS was associated with TopoIIα negativity (HR: 0.270; 95%CI: 0.139~0.521; P< 0.001, **Table 2**). In the multivariate analysis (**Table 2**, right), only TopoIIα (HR: 0.316; 95%CI: 0.162~0.617; P = 0.001) remained while Hsp27 failed to be an independent prognostic factor for DFS.

**Table 2:** Cox proportional hazard regression analysis of DFS in LABC.

Terms	Univariate analysis		Multivariate analysis	
	HR (95% CI)	*P*	HR (95% CI)	*P*
Age (>50)	0.949 (0.492, 1.832)	0.876		
Menopausal status (Post)	1.036 (0.538, 1.995)	0.915		
Nodal status (N2-N3)	4.465 (1.071, 18.608)	*0.040*		
TNM stage (TNM3b-TNM3c)	1.700 (0.775, 3.733)	0.186		
Histological grade (poorly differentiated)	3.490 (1.525, 7.987)	*0.003*	2.914 (1.260, 6.741)	*0.012*
ER (Positive)	0.459 (0.238, 0.883)	*0.020*		
PR (Positive)	0.609 (0.315, 1.175)	0.139		
Her-2 (Positive)	1.214 (0.620, 2.378)	0.571		
Ki67 (Elevated)	1.765 (0.882, 3.531)	0.108		
Molecular subtypes		0.259		
Luminal B1	1.327 (0.301, 5.842)	0.709		
Luminal B2	0.873 (0.146, 5.228)	0.882		
TNBC	2.434 (0.490, 12.100)	0.227		
HER2-enriched	2.544 (0.563 11.496)	0.225		
Hsp27 (Positive)	3.028 (1.506, 6.086)	*0.002*		
TopoIIα (Positive)	0.270 (0.139, 0.521)	*<0.001*	0.316 (0.162, 0.617)	*0.001*

Statistically significant values (P< 0.05) are given in italics.

Regarding OS, the follow-up period ranged from 10 to 68 months (median: 34 months). Hsp27 (+) patients showed a lower OS than those Hsp27 (-) patients (P= 0.001, **Figure 5B**), in accordance with Cox regression analysis (HR: 6.213; 95% CI: 1.765~21.869; P = 0.004; **Table 3**). Additionally, TopoIIα (HR: 0.147; 95% CI: 0.051~0.425; P< 0.001) was a beneficial predictor. Furthermore, as shown in **Table 3**, the multivariate analysis showed that only TopoIIα (HR: 0.214; 95%CI: 0.074~0.619; P = 0.004) was an independent predictor for OS in all 103 LABC patients.

**Table 3:** Cox proportional hazard regression analysis of OS in LABC.

Terms	Univariate analysis		Multivariate analysis	
	HR (95% CI)	*P*	HR (95% CI)	*P*
Age (>50)	0.571 (0.212, 1.534)	0.266		
Menopausal status (Post)	0.503 (0.182, 1.385)	0.183		
Nodal status (N2-N3)	–			
TNM stage (TNM3b-TNM3c)	1.811 (0.515, 6.365)	0.355		
Histological grade (poorly differentiated)	–			
ER (Positive)	0.249 (0.086, 0.717)	*0.010*		
PR (Positive)	0.427 (0.154, 1.180)	0.101		
Her-2 (Positive)	1.222 (0.454, 3.292)	0.691		
Ki67 (Elevated)	2.814 (1.055, 7.505)	*0.039*		
Molecular subtypes		0.085		
Luminal B1	0.604 (0.063, 5.811)	0.662		
Luminal B2	1.101 (0.100, 12.170)	0.937		
TNBC	4.299 (0.501, 36.921)	0.184		
HER2-enriched	1.928 (0.225, 16.531)	0.549		
Hsp27 (Positive)	6.213 (1.765, 21.869)	*0.004*		
TopoIIα (Positive)	0.147 (0.051, 0.425)	*<0.001*	0.214 (0.074, 0.619)	*0.004*

For patients characterized by N1 nodal status or well-/moderately-differentiated tumors were all censored, HR (95%CI) could not be calculated by Cox regression. Statistically significant values (P < 0.05) are given in italics.

### Association between expression of TopoIIα and Hsp27 in combination and resistence to anthracycline-based NAC in LABC patients

Considering the prognostic values of Hsp27 and TopoIIα, their prognostic value in combination on survival was further examined. There were 4 patients with negative expression of both TopoIIα and Hsp27 [TopoIIα (-) Hsp27 (-), 4/103], 25 patients with negative expression of TopoIIα and positive expression of Hsp27 [TopoIIα (-) Hsp27 (+), 25/103, 24.3%], 51 patients with positive expression of TopoIIα and negative expression of Hsp27 [TopoIIα (+) Hsp27 (-), 51/103, 49.5%] and 23 patients with positive expression of both Hsp27 and TopoIIα [TopoIIα (+) Hsp27 (+), 23/103, 22.3%] (**Table 4**). Kaplan-Meier analysis indicated that patients characterized by TopoIIα (-) Hsp27 (+) had significantly lower DFS and OS (P< 0.001 and 0.001, respectively) than those in the other three groups (**Figures 5C, D** **)**.

**Table 4:** Kaplan-Meier analysis for patients with different expressions of TopoIIα and Hsp27.

Characteristic	No. of patients	DFS (%)			OS (%)		
		15 Months	30 Months	*P*	15 Months	30 Months	*P*
TopoIIα (-) Hsp27 (-)	4 (3.9%)	75.0	75.0	*<0.001*	100	100	*0.001*
TopoIIα (-) Hsp27 (+)	25 (24.3%)	60.0	35.6		96.0	62.2	
TopoIIα (+) Hsp27 (-)	51 (49.5%)	92.2	79.6		96.1	96.1	
TopoIIα (+) Hsp27 (+)	23 (22.3%)	82.6	71.5		100.0	94.1	

Statistically significant values (P< 0.05) are given in italics.

## Discussion

The NAC administration represents a pivotal therapeutic approach for patients with LABC. Nevertheless, the development of resistance to NAC remains a paramount clinical obstacle. Thus, identification of biomarkers capable of accurately predicting the responsiveness of patients to NAC is an urgent and justifiable pursuit.

The overexpression of Hsp27 has been linked to a range of biological processes such as aggressiveness, metastasis, apoptotic cell death inhibition immunity, drug resistance, and tumor prognosis, etc (28, 29). Although several cell line studies have indicated a potential association between Hsp27 and the responsiveness of breast cancer to anthracyclines (30, 31), less clinical investigations have been conducted to confirm the prognostic role of Hsp27 in breast cancers. In patients with node-negative breast cancer, an early study failed to see significant correlations between Hsp27 expression and histopathological features or Ki67 or DFS, but Hsp27 was associated with a short OS (7), that could not even be seen in patients with node-positive breast carcinomas (32). As Ki67 has been clinically utilized to define highly proliferative breast cancers (33), we previously reported that Ki67 decrease during neoadjuvant chemotherapy predicts favorable relapse-free survival in patients with LABC (34). The present study, otherwise, proposes that Ki67 in Hsp27-positive LABC patients is significantly elevated by NAC, suggesting that Hsp27 may predict poor prognosis of LABC patients treated with NAC. The current study additionally found a significant higher expression of Hsp27 in poorly differentiated LABC, which is consistent with our previous report showing Hsp27 overexpression in breast cancer stem-like cells (35).

One previous study similarly included LABC patients (n = 60) who were treated with doxorubicin- or epirubicin-based neoadjuvant monochemotherapy, indicated nuclear Hsp27 expression was significantly increased in invasive cells after doxorubicin/epirubicin administration, but did not find correlations between Hsp27 and the clinical and pathological response to neoadjuvant therapy, and the prognostic value of Hsp27 (11). However, this current study argued an existing association of Hsp27 with aggressive features, response to neoadjuvant therapy, and survival (using KM curves), albeit Hsp27 might not be an independent prognostic indicator for DFS or OS. The inconsistent findings may be due to the difference of Hsp27 antibody, evaluation method, genetic background, and treatments etc.

Previous studies have suggested that high expression of Hsp27 may induce drug resistance of tumor cells (14). Consistently, we found that patients with stable and progressive disease had a higher percentage of Hsp27 expression, and all patients in the Hsp27 negative group showed pCR. In addition, our study suggests that the LABC patients with high levels of Hsp27 tend to have a shorter DFS and OS than those with negative Hsp27 expression, but might not be an independent prognostic indicator, that could be closely related to TopoIIα.

A previous study demonstrated that Hsp27 overexpression may inhibit TopoIIα expression in breast cancer cells (14). Our results further pointed out that patients with Hsp27-positive and TopoIIα-negative tumors benefited from a considerably lower DFS and OS. However, the underlying molecular mechanisms remain unclear and warrant further study. In summary, our results suggest that Hsp27 expression is associated with the NAC outcome in LABC, though it is not an independent prognostic factor for DFS and OS. Further prospective investigations of a larger number of LABC patients treated with NAC will be necessary to determine whether Hsp27 could predict efficacy of treatment. More importantly, we co-evaluate the expression status of Hsp27 and TopoIIα to identify that higher Hsp27/lower TopoIIα could prognose worse outcome for NAC resistance in breast cancer.

## Conclusions

Although either Hsp27 or TopoIIα might be able to predict LABC patient responsiveness to anthracycline-based neoadjuvant treatments, combination of these two biomarkers could more precisely prognose the outcome of these patients.

## Data availability statement

The publicly available datasets presented in this study can be found in online repositories. The names of the repository/repositories and accession number(s) can be found in the article/**Supplementary Material**. The dataset from the Cancer Hospital of Shantou UniversityMedical College is available on reasonable request.

## Ethics statement

The studies involving human participants were reviewed and approved by Cancer Hospital of Shantou University Medical College. The patients/participants provided their written informed consent to participate in this study.

## Author contributions

(I) Conception and design: YZ, YC. (II) Administrative support: YC, WH. (III) Provision of study materials or patients: YX, CH, WH, LH. (IV) Collection and assembly of data: FZ. (V) Funding Acquisition: YZ, CH. (VI) Manuscript writing: All authors. (VII) Final approval of manuscript: All authors. All authors contributed to the article and approved the submitted version.
