# Supplementary material for: Evaluating the expression of heat shock protein 27 and topoisomerase II α in a retrospective cohort of patients diagnosed with locally advanced breast cancer and treated with neoadjuvant anthracycline-based chemotherapies
Source: Front Oncol. 2023 Aug 15;13:1067179. doi: 10.3389/fonc.2023.1067179 (PMC10478710; doi:10.3389/fonc.2023.1067179)

Supplementary Figure 1 The correlation between Hsp27 expression level and response to chemotherapy. **(A)** Boxplot shows the expression of HSP27 in patients receiving TFAC therapeutic regimen (CTR_Microarray_48), **(B)** another cohort receiving TFAC therapeutic regimen (CTR_Microarray_106), **(C)** epirubicin therapeutic regimen using the CTR database. All of these AUC curves show the accuracy of grouping detection (CTR_Microarray_52).

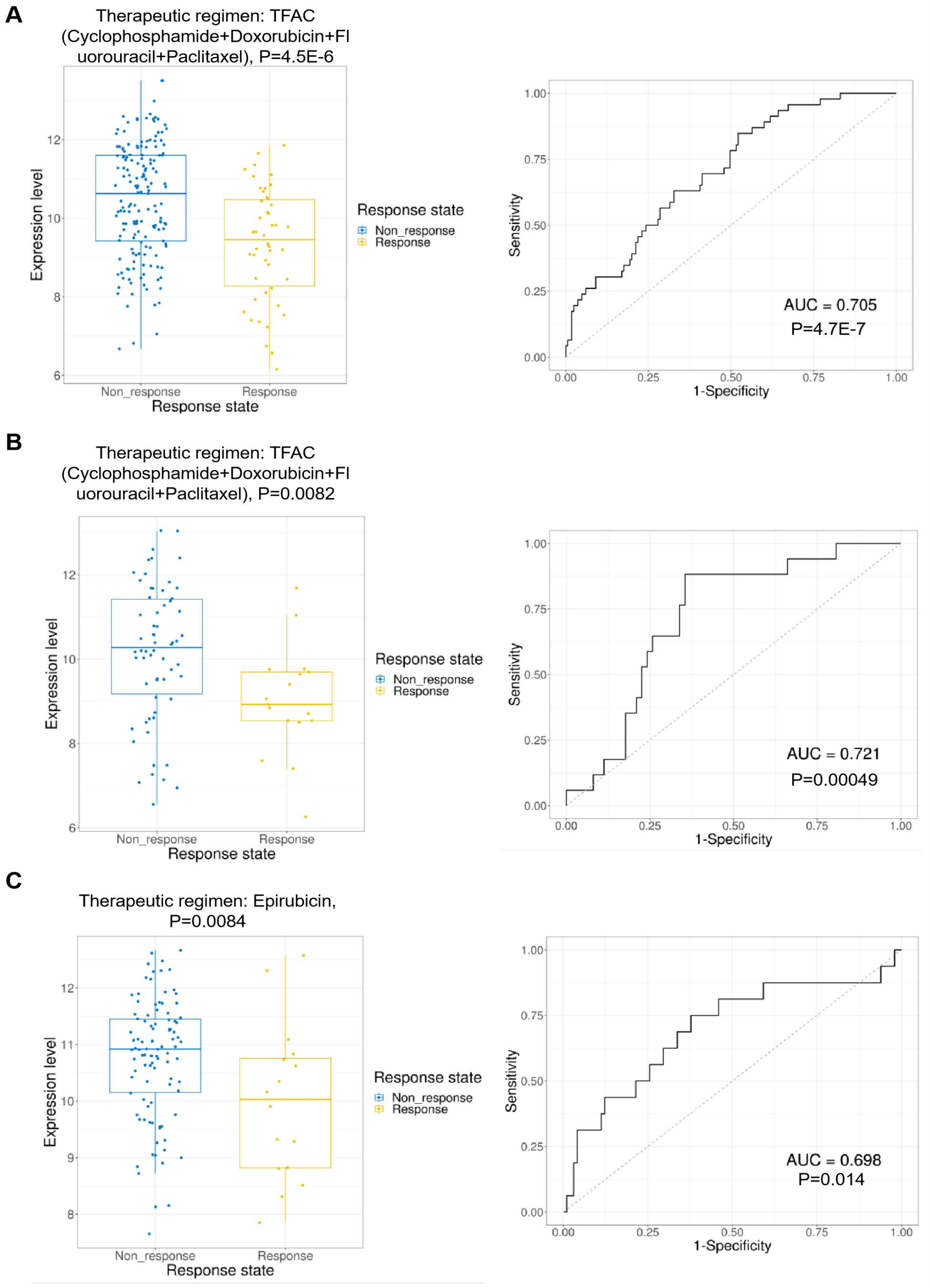

Supplementary Figure 2 The expression level of Hsp27 and survival at individual datasets. **(A, D)** Survival curves using the PrognoScan database are shown for OS, **(B, E)** RFS and **(C, F)** DMFS based on GEO database. All dotted lines represent confidence bands.

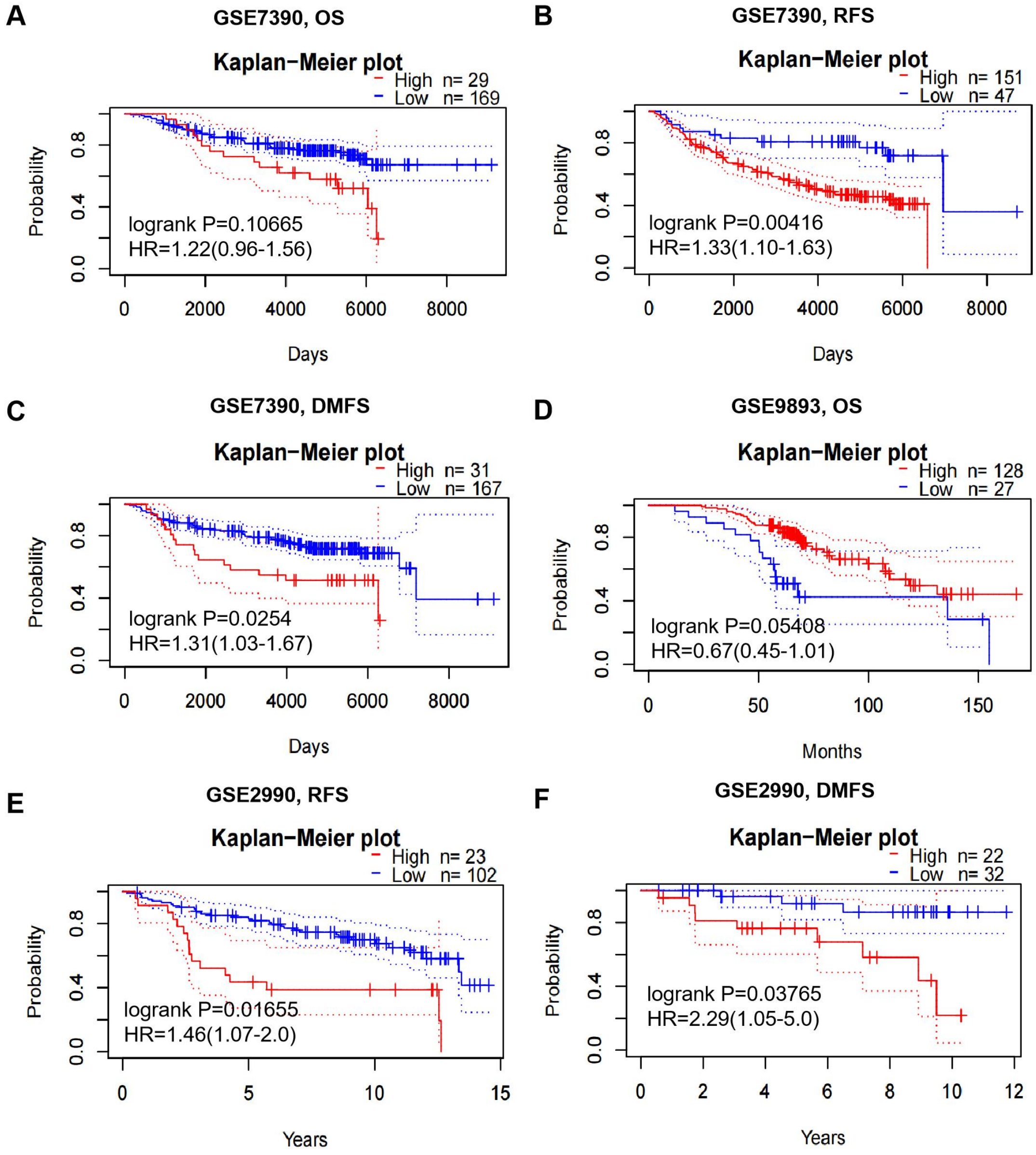

Supplementary Figure 3 Enrichment analyses of Hsp27 related genes. **(A)** GO_BP enrichment analysis of HSP27 related genes. **(B)** GO_CC enrichment analysis of HSP27 related genes. **(C)** GO_MF enrichment analysis of HSP27 related genes.

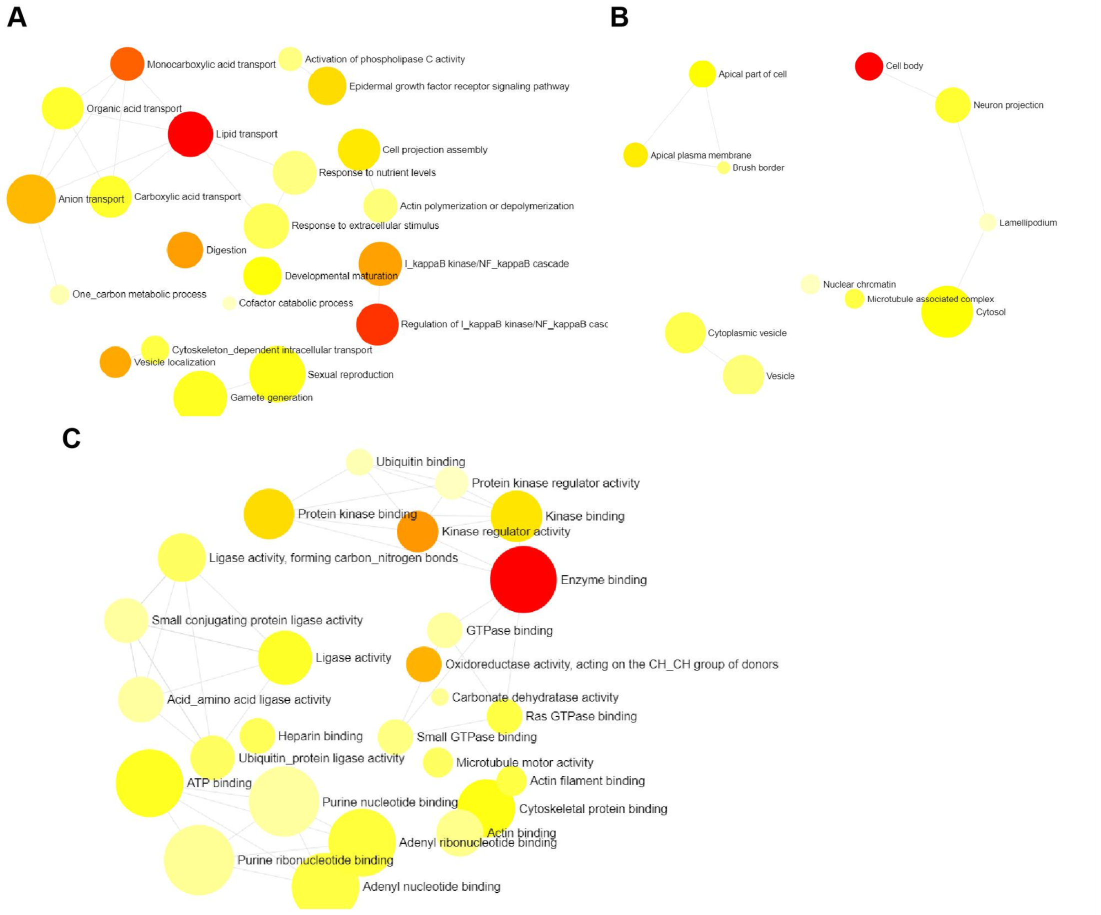

Supplement: Supplementary file 1 [file DataSheet_1.pdf]
