# Supplementary material for: Evaluating the expression of heat shock protein 27 and topoisomerase II α in a retrospective cohort of patients diagnosed with locally advanced breast cancer and treated with neoadjuvant anthracycline-based chemotherapies
Source: Front Oncol. 2023 Aug 15;13:1067179. doi: 10.3389/fonc.2023.1067179 (PMC10478710; doi:10.3389/fonc.2023.1067179)
Supplement: Supplementary file 2 [file Table_1.pdf]

Supplementary Table 1 A list of Hsp27 co-expressed genes

Query	Statistic	P-value	FDR
ELMO2	0.238224972	3.19E-08	2.02E-07
CREB3L1	0.093614691	0.031822588	0.05806442
RPS11	-0.181988656	2.68E-05	9.74E-05
PNMA1	0.16556572	0.000136447	0.000432926
MMP2	0.039444798	0.366603705	0.465080706
C10orf90	-0.248053611	8.12E-09	5.82E-08
ZHX3	0.175285476	5.30E-05	0.000183166
ERCC5	-0.110619469	0.011123871	0.022907128
GPR98	0.26197323	1.06E-09	8.72E-09
RXFP3	0.064222612	0.141307244	0.209777461
APBB2	0.415563309	2.23E-23	1.93E-21
PRO0478	0.131248804	0.002560943	0.006211929
KLHL13	-0.154326114	0.000381873	0.001095724
PRSSL1	0.251049253	5.29E-09	3.93E-08
PDCL3	0.019386846	0.657320811	0.736760056
DECR1	-0.053657853	0.219227921	0.30286059
SALL1	0.123861192	0.004442226	0.010120508
CADM4	-0.197369732	5.10E-06	2.18E-05
RPS18	-0.297566612	3.24E-12	4.05E-11
HNRPD	-0.202860639	2.73E-06	1.23E-05
CFHR5	0.073572133	0.091868096	0.146346352
SLC10A7	0.084304625	0.053317347	0.090841886
OR2K2	-0.027314541	0.533886258	0.627339308
LMAN1	-0.205922401	1.91E-06	8.86E-06
SUHW1	-0.206021491	1.89E-06	8.77E-06
CHD8	0.207733275	1.54E-06	7.31E-06
SUMO1	-0.129411828	0.002944513	0.007036848
GP1BA	-0.158632826	0.000259504	0.000770865
DDB1	0.12846435	0.00316215	0.007487123
MYO9B	-0.209610895	1.24E-06	5.96E-06
MMP7	-0.223930022	2.11E-07	1.18E-06
CRNKL1	0.197210824	5.19E-06	2.22E-05
C9orf45	-0.114598031	0.00852119	0.018061634
XAB2	0.070706086	0.10527696	0.164176522
RTN1	-0.026089917	0.550479165	0.641808188
KLHL14	-0.030655587	0.482948216	0.578991729
TBX10	0.031188169	0.475373787	0.5715826
CENPQ	-0.001279854	0.976638698	0.98239021
UTY	0.064997144	0.136561787	0.203963669
ZBTB12	0.032841158	0.452284039	0.54962343
DTNBP1	-0.06471447	0.13827928	0.206089235
KBTBD8	-0.260865887	1.25E-09	1.02E-08
ZEB1	0.124976422	0.00409513	0.009429869
ZG16	0.342720886	6.08E-16	1.62E-14
MIER1	-0.163790299	0.000161262	0.00050284
ADAM5P	0.00712509	0.872227006	0.910735925
CHD9	0.06469376	0.138405758	0.206216865
STK16	0.073505136	0.092165121	0.146667528

KIAA1486	0.059759322	0.171557465	0.247863453
TOB2	0.025175433	0.564541891	0.654518864
BANK1	-0.217676966	4.63E-07	2.44E-06
OR2V2	0.007911699	0.856349121	0.900022757
GRM2	-0.073539315	0.092013496	0.146540013
PROSC	0.145083315	0.000846277	0.002273375
SPIN2B	0.050141274	0.250984698	0.338590499
PIR	-0.04170162	0.339803456	0.435847195
IPO9	0.030554749	0.484389662	0.580267296
EVC	0.027272258	0.532556118	0.626015594
CXCL13	-0.195480638	6.29E-06	2.64E-05
KIAA1199	-0.026194748	0.548878142	0.640305766
SORL1	-0.069206447	0.112886021	0.173925368
NAT10	-0.031325618	0.47342958	0.569579042
CHD1	0.23486945	5.03E-08	3.08E-07
SYN3	0.057681047	0.186551516	0.265165342
SLC22A2	-0.06409539	0.142098659	0.210850545
SERPINF1	0.023142249	0.596411121	0.683349457
WDR34	0.148692121	0.000623613	0.001718009
OR7A17	0.0300343	0.491866174	0.58670904
C9orf11	-0.021060477	0.630190057	0.712454249
RNF216L	0.028829133	0.509413383	0.603774652
LHB	0.096715609	0.026550235	0.049622309
STK25	-0.102731326	0.018435942	0.036010994
TAOK3	-0.004029367	0.926544852	0.947974092
LOC152573	-0.196052068	5.91E-06	2.49E-05
C3orf39	0.011898707	0.785427055	0.845812777
C14orf108	-0.02395071	0.58364115	0.670805501
CDC25B	0.009552544	0.826986266	0.877655946
BMP3	0.016029938	0.71377522	0.784871929
TMEM180	-0.015606151	0.721022584	0.791284645
MAP1LC3C	-0.168276689	0.000105371	0.000341553
CRYGC	0.045377701	0.298905791	0.393401479
POU3F1	-0.070370246	0.106944715	0.166229286
C20orf32	-0.114190388	0.008760292	0.018517373
CCDC95	0.219737673	3.58E-07	1.92E-06
HIGD1B	0.093658654	0.031742003	0.057947777
USP6NL	-0.363223351	7.55E-18	2.75E-16
ABCD4	0.040350762	0.355691088	0.453764419
DIMT1L	-0.141046146	0.001181197	0.003085464
TEK	0.036404558	0.404722278	0.503059892
SLC25A46	0.055981036	0.199892245	0.280881494
LARP7	-0.019608859	0.653650724	0.73398435
CD160	-0.174690615	5.63E-05	0.000192953
MT1JP	-0.101804221	0.019524418	0.037884395
PHF20	0.275171628	1.36E-10	1.31E-09
CPNE4	0.14319697	0.000990002	0.0026296
GTPBP1	-0.153435819	0.000413109	0.001178204
RAB33B	-0.071602449	0.100926352	0.158194275
ALDOC	0.015261311	0.726938361	0.795926018
ZNF212	-0.07241439	0.097109931	0.153464139

NUDT1	-0.066956673	0.125103788	0.189707322
RFPL2	0.069422979	0.111761387	0.17253848
ZNF83	0.101598061	0.019773934	0.038235823
GDPD5	-0.13045	0.002721762	0.00656061
PDCD4	0.034830857	0.425348619	0.523617349
CEP350	-0.017784408	0.684050798	0.760098495
OR10A2	-0.057231931	0.190010433	0.269086631
CST7	-0.14256574	0.001042921	0.002755875
CIAO1	-0.001562328	0.971484665	0.978486356
SELL	-0.115924896	0.0077827	0.016610838
OR8J3	0.082717901	0.057980397	0.097864798
LTBP4	-0.004323847	0.921193341	0.945014657
SIRT6	0.106779722	0.01427998	0.028611142
CCL19	-0.055393558	0.204660244	0.286338075
PPIL1	-0.026787222	0.539872992	0.632644135
GBP7	0.03196818	0.464398684	0.561460383
STK17A	-0.124422175	0.004264439	0.009758851
ABR	0.141660768	0.001123352	0.002949399
OR9G1	-0.037272985	0.393599906	0.491919882
FOXE1	-0.068673438	0.115692285	0.177429296
CNGA3	0.086939192	0.046266414	0.080501466
GML	0.119230414	0.006185557	0.013571555
CD38	-0.196587399	5.56E-06	2.36E-05
ZDHHC6	-0.102649367	0.018529985	0.036183194
NEFH	-0.016914817	0.698725963	0.772606716
CTDSP2	-0.036191003	0.407485816	0.505779225
PGBD5	-0.16782064	0.000110084	0.000356079
CCNY	-0.25483236	3.05E-09	2.36E-08
RMND5B	0.495838743	5.46E-34	3.36E-31
ZNF257	0.034589364	0.428567137	0.526420161
FLJ22167	-0.124476574	0.004247545	0.009733024
EXOSC7	-0.047176493	0.28013969	0.371886648
ROR2	0.044749285	0.305654616	0.400227904
MAOA	0.120550895	0.005634574	0.01247192
TNNT3	-0.048271437	0.269115857	0.359113152
GYPC	-0.253418494	3.75E-09	2.85E-08
C7orf33	-0.12547704	0.003947458	0.009118301
PLIN	0.097797224	0.024897329	0.046831959
LOC90826	-0.098220331	0.02427535	0.045745978
RNF4	0.157228052	0.000294675	0.000868627
F8A1	0.009523899	0.827496926	0.87804635
PLEKHG4	-0.202579818	2.82E-06	1.27E-05
GRB2	0.137234779	0.001605595	0.004059542
HIST1H2AD	0.251030161	5.30E-09	3.93E-08
DUS3L	-0.110660056	0.011094108	0.02286115
EIF1	0.132332533	0.002356606	0.005752588
RP5-1077B9.4	-0.120780303	0.005543484	0.012310204
FPGT	0.061080587	0.161861578	0.235604945
GDF10	-0.076344401	0.080235142	0.129594401
COQ9	0.082201883	0.059567823	0.100077197
GCC2	-0.091493104	0.035924984	0.064629236

RARRES3	0.20666417	1.75E-06	8.17E-06
PLXNA1	-0.180956058	2.98E-05	0.000107619
KIAA0100	0.057040149	0.191501729	0.271011307
PMF1	-0.028682447	0.511571225	0.606215352
FNDC1	-0.074596605	0.087420979	0.140279646
HS2ST1	-0.157948764	0.000276109	0.000817821
CRELD2	-0.127826586	0.003316772	0.007808129
C8G	-0.088343183	0.042840211	0.075393377
CD82	-0.299257187	2.41E-12	3.11E-11
LIM2	-0.006277129	0.88579823	0.9194491
UNQ6490	-0.018406388	0.673626439	0.750983111
MMP16	0.105161066	0.015830851	0.031391133
DRD3	0.002034842	0.96286592	0.973312928
C5orf26	-0.023369256	0.592812646	0.679695126
C11orf73	-0.219982195	3.47E-07	1.86E-06
PTP4A2	0.241561291	2.02E-08	1.34E-07
OR4M2	-0.009396251	0.829773428	0.879854597
HPCA	0.014732568	0.73604052	0.804118215
SEC14L1	-0.080376203	0.065475317	0.108519602
CHFR	0.080332532	0.065622322	0.10870465
EMILIN1	0.144393921	0.000896403	0.002396515
NDUFS4	0.198695769	4.39E-06	1.90E-05
COL18A1	0.087406375	0.04510165	0.078742205
PDZD3	0.075287817	0.084519581	0.135907662
C9orf16	0.031535014	0.470476084	0.566697925
ERBB2IP	0.269022424	3.58E-10	3.24E-09
EMX2	0.004384283	0.920095489	0.944203465
FUS	0.00346028	0.936895902	0.955543689
TF	-0.167476429	0.000113772	0.000367042
CLCN4	-0.410024897	9.57E-23	7.65E-21
CXorf56	-0.028249741	0.517964206	0.611434421
C11orf72	0.037288659	0.393400875	0.491771087
ELAC2	0.040517097	0.353709958	0.451518067
NPR1	-0.080389124	0.065431875	0.10847684
ASS1	-0.245379179	1.19E-08	8.21E-08
USP42	-0.021403768	0.624291671	0.707346506
POLR2J	0.162794705	0.000176971	0.000546556
SEC23IP	0.08008161	0.066472241	0.11005326
UQCRC1	0.201402504	3.23E-06	1.43E-05
LOC729603	-0.066786581	0.126067756	0.190839643
C1orf71	-0.01302617	0.765662658	0.828883747
POLG	-0.006106957	0.888873916	0.920984352
ADAM23	0.112557618	0.009779006	0.020401021
TFR2	-0.093899852	0.031302972	0.057252223
RICTOR	0.048145638	0.270367076	0.36054807
MGC39606	-0.261196847	1.19E-09	9.74E-09
C19orf55	0.103687349	0.017369351	0.034122729
SNAPC1	-0.082691155	0.058061806	0.097975315
GNA11	0.123994008	0.004399539	0.010039134
CCDC52	0.173010203	6.65E-05	0.00022405
FSIP1	0.468115842	5.30E-30	1.63E-27

UPF3A	-0.1146182	0.008509513	0.018043101
IGSF11	-0.107002079	0.014077731	0.0282704
LAGE3	0.05745457	0.188289908	0.266957493
CHST6	-0.23900347	2.87E-08	1.84E-07
UNC13B	-0.092473043	0.033977383	0.061470642
TTLL4	-0.417399884	1.37E-23	1.25E-21
ZNF687	0.025542057	0.558883364	0.64963588
SDC2	-0.050065941	0.251698321	0.339331939
COX7A2	0.139780035	0.001309102	0.003383634
LAMB4	-0.118134291	0.00667916	0.014527822
FAM24A	0.015525912	0.722397619	0.792261041
LRRTM3	0.062184691	0.154396621	0.225990199
GPHB5	-0.136124689	0.001753263	0.004394951
OR4C13	-0.058978068	0.177237092	0.25415832
EIF3EIP	-0.365493018	4.55E-18	1.76E-16
HABP4	-0.182719849	2.48E-05	9.10E-05
TMEM125	0.075533026	0.083509164	0.134458719
CNTN2	-0.073145087	0.093774579	0.148881974
ASNSD1	-0.261829887	1.08E-09	8.90E-09
FUT4	-0.261567431	1.12E-09	9.23E-09
ACF	0.059864533	0.17039431	0.246489935
LOC158381	0.044848673	0.304580592	0.398991491
CDH8	0.030329699	0.487615057	0.582995331
AGPS	-0.065086449	0.136022607	0.203306517
C4orf18	0.296990162	3.58E-12	4.43E-11
PECI	0.13946305	0.00134306	0.003462672
UNG	-0.10666296	0.014387203	0.028816583
GSTP1	-0.426408507	1.19E-24	1.30E-22
DCUN1D5	-0.314429213	1.56E-13	2.57E-12
DKFZP564J0863	-0.184049847	2.16E-05	8.02E-05
SLC9A3R1	0.589522621	1.53E-50	3.13E-47
BCDO2	4.70E-05	0.999141337	0.999303852
CHMP7	-0.039300446	0.368361498	0.46654065
REM2	0.15766981	0.000283162	0.000836694
DNHD1	0.00312818	0.94294146	0.959322806
FKBP4	0.408559751	1.40E-22	1.10E-20
ZNF350	0.069373628	0.11201693	0.172802835
MGC11102	0.159818373	0.000232917	0.000700345
BST1	-0.127222239	0.003469597	0.008133644
KISS1R	0.105426634	0.015566694	0.030957181
NCR2	-0.011880976	0.785739022	0.846000569
DEFB125	0.034585199	0.431281033	0.52906258
UBE2W	0.163704294	0.000162566	0.000506391
KRT15	-0.152024845	0.000467525	0.001318116
C10orf99	0.022677657	0.603806472	0.689343854
SCN11A	0.007665363	0.86077551	0.903353759
GFI1	-0.222001987	2.69E-07	1.47E-06
RDHE2	0.153234259	0.000420504	0.001195965
FHL1	0.002861931	0.947790643	0.962897012
OSGEP	0.015114335	0.729464707	0.798127844
GATA1	0.078954681	0.070401533	0.115717463

SMC6	-0.333874245	3.66E-15	8.25E-14
TTTY14	0.025158863	0.564798275	0.654653497
LPIN3	0.153015977	0.000428651	0.001217447
RPL4	-0.254828963	3.05E-09	2.36E-08
RBPMS	0.158993916	0.00025112	0.000748854
PRPF3	-0.117268434	0.007093724	0.015315769
EMR1	-0.097059859	0.026014192	0.048694449
SPATA19	-0.023206326	0.595394382	0.682527974
XCR1	0.215395914	6.13E-07	3.14E-06
IRX3	0.05039577	0.248584267	0.335501461
RBM6	-0.020656349	0.636448387	0.718472761
KLF4	-0.036710324	0.400784972	0.499276092
UNC5CL	-0.000955844	0.982551779	0.987046379
SEBOX	0.037839707	0.386896143	0.485987239
BTK	-0.141846351	0.001106406	0.002906601
KRCC1	-0.094000132	0.031121976	0.056938122
C6orf27	0.105434195	0.015559231	0.030952349
SYTL5	0.26761904	4.45E-10	3.94E-09
PRND	0.108750861	0.012573041	0.025540678
LOC653319	0.043429453	0.320154454	0.415498045
PIGL	0.101465323	0.019936052	0.038465643
HUS1	-0.161547044	0.000198684	0.000606007
SFRS6	0.165643203	0.00013545	0.000429986
C17orf77	0.089087863	0.041111454	0.072788463
UIMC1	0.112554861	0.009780811	0.020401021
FXYD2	-0.074125661	0.089443301	0.143188455
LOC283152	0.174489342	5.74E-05	0.000196638
ZNF667	-0.297438107	3.32E-12	4.11E-11
ZCCHC12	-0.052645222	0.228062277	0.312467678
TFEC	-0.115711932	0.007897236	0.016814441
ATP7B	0.256348997	2.44E-09	1.92E-08
POLD2	0.01169142	0.789076272	0.848702116
RG9MTD1	-0.12483485	0.004137789	0.0095115
ACOT2	0.323680951	2.70E-14	5.18E-13
HIST1H4I	0.192137438	9.10E-06	3.66E-05
PPARGC1A	-0.345014296	3.78E-16	1.05E-14
ETFA	0.003379253	0.938370599	0.956392537
POLRMT	0.15578887	0.000335288	0.000976178
ZNF146	-0.324794872	2.18E-14	4.23E-13
MIA2	-0.00929606	0.831561249	0.881142533
KLHL6	-0.141334846	0.001153693	0.003017464
HOXB5	0.179265976	3.55E-05	0.000126654
NENF	-0.003434983	0.937356288	0.955854696
CUGBP1	0.001904541	0.965242231	0.974674968
PRSS22	0.241958928	1.91E-08	1.27E-07
CASC4	0.299130525	2.47E-12	3.17E-11
CUL4B	-0.138022156	0.001507873	0.003844075
CENPJ	-0.197356135	5.10E-06	2.18E-05
PITX1	0.076556984	0.07939475	0.128439442
FLJ31033	0.136226119	0.001739271	0.004363434
CELSR3	0.16960487	9.27E-05	0.000303209

ZNF568	-0.182936017	2.43E-05	8.91E-05
ITSN1	0.112790591	0.009627492	0.020122178
EHBP1L1	-0.179093213	3.61E-05	0.000128757
C19orf2	-0.231495843	7.88E-08	4.68E-07
DCTN1	0.116852851	0.007300766	0.015696647
LIN28B	-0.059677623	0.17173507	0.248061768
TNKS2	-0.048001274	0.271807847	0.362076787
C1QBP	-0.110210475	0.011427762	0.023470044
CADPS2	0.0125836	0.773403552	0.835351913
SRMS	0.183379093	2.32E-05	8.56E-05
GJA9	-0.038826646	0.374167697	0.472047018
MGC24975	0.062545853	0.152012254	0.22345765
TRIM45	0.077446316	0.07595607	0.123788464
TSP50	-0.010849716	0.803940831	0.860325822
TCP1	-0.105884566	0.015120272	0.030176746
TMED7	0.218400546	4.23E-07	2.25E-06
CMA1	-0.003901872	0.928930624	0.949618617
CENPL	-0.167517134	0.00011333	0.000366385
PTCRA	-0.033214565	0.447157017	0.544576846
FST	0.011228694	0.797238949	0.855327675
VWCE	-0.098477863	0.023903384	0.045183494
PAWR	0.095653894	0.028263801	0.052363397
ABCC12	0.022391291	0.608385196	0.693284019
LDLR	0.20769052	1.55E-06	7.34E-06
ASTN2	0.15499573	0.000359845	0.001040776
LOC441212	-0.188096836	1.41E-05	5.47E-05
GPATCH8	-0.026496492	0.544282641	0.636514636
TANC2	0.38596971	3.95E-20	2.09E-18
KIF4A	0.006527744	0.881271771	0.916600155
C18orf18	-0.199622169	3.95E-06	1.73E-05
PGM1	-0.387376296	2.81E-20	1.54E-18
KIAA0258	-0.109778402	0.011756762	0.024073369
CPD	0.163838989	0.000160528	0.000500806
SNCAIP	-0.116424194	0.007519969	0.016117215
DCT	-0.02314101	0.596430774	0.683349457
HLA-DOA	-0.123564088	0.004539073	0.01030678
OR11L1	0.07574754	0.08263325	0.133083251
UPK1B	-0.258824846	1.69E-09	1.36E-08
DNAJB4	-0.23828435	3.17E-08	2.01E-07
UGT1A8	-0.026672421	0.541612098	0.634356722
HIST1H4L	-0.109357194	0.012085523	0.024639881
PECR	0.068858387	0.114712409	0.176297577
HSPA2	0.159595298	0.000237716	0.000711645
WFIKKN1	0.203753186	2.46E-06	1.12E-05
SERP1	-0.030413093	0.486418522	0.582017414
SYDE2	0.292577794	7.67E-12	9.16E-11
TACR2	0.161100518	0.000207044	0.000629321
NUP85	0.065614564	0.132867546	0.199414825
CD177	0.052384806	0.230374521	0.314793985
LGR5	-0.203519932	2.53E-06	1.14E-05
PIGG	0.214460898	6.88E-07	3.50E-06

PTHR1	0.068205736	0.118199497	0.180753223
RAB5A	-0.033072369	0.449105531	0.546463555
FLJ13224	-0.134428558	0.002003062	0.004944531
USP9Y	0.020762053	0.634723092	0.7167883
C7orf53	0.009620723	0.825771107	0.876668946
LRP1B	0.077616514	0.075312	0.122771338
XAF1	0.017999653	0.680436384	0.756737805
ABCG8	-0.025639417	0.557385324	0.648384858
ANKDD1A	-0.195377699	6.37E-06	2.67E-05
DAND5	-0.181039519	2.96E-05	0.000106823
SPAG6	0.023759881	0.586643899	0.673249969
LINCR	-0.277600396	9.21E-11	9.12E-10
ZDHHC22	0.185103964	1.94E-05	7.29E-05
CCDC60	0.006907231	0.875021294	0.912193797
THOC7	-0.097086233	0.025973512	0.04864792
TCTA	0.302379826	1.39E-12	1.90E-11
OR8K3	-0.058342239	0.181544023	0.259306434
NY-REN-7	-0.056866498	0.192859429	0.27249371
B2M	-0.051565261	0.237759564	0.323233155
C6orf141	0.227833327	1.27E-07	7.31E-07
LPPR4	-0.116928327	0.007262767	0.01562041
SQLE	0.178834517	3.71E-05	0.000131815
SEPHS1	-0.320067534	5.40E-14	9.73E-13
BTBD14B	0.137896022	0.001523151	0.0038782
PLRG1	-0.173945776	6.06E-05	0.000206282
SPG7	0.069887491	0.109378529	0.169327436
ZNF614	-0.055254692	0.205799241	0.287800667
PARD6G	-0.077995444	0.073894004	0.120812079
INPP5B	-0.354858739	4.70E-17	1.54E-15
GRPEL2	0.042691222	0.328457189	0.424322406
PPID	0.053432002	0.221176733	0.304936262
TRIM56	0.067918822	0.119758462	0.182909783
UBE2J1	-0.333408981	4.02E-15	8.93E-14
IL20RA	0.199357973	4.07E-06	1.78E-05
LOC387856	0.119107012	0.006239442	0.013668091
C1orf107	-0.098555297	0.02379251	0.045015429
UTS2R	0.067737993	0.120749225	0.184011644
C19orf22	0.17377989	6.16E-05	0.000209239
SAFB2	0.104315888	0.016697878	0.032940409
KIAA0652	0.002758005	0.949683986	0.963949626
KLRG1	-0.246540182	1.01E-08	7.06E-08
MS4A8B	0.364165418	6.12E-18	2.30E-16
FRAG1	0.077392852	0.07615931	0.124053933
KIAA1546	-0.175673791	5.10E-05	0.000176984
E2F4	-0.120780274	0.005543496	0.012310204
CLEC4M	0.003560554	0.935071207	0.954157459
BTBD14A	-0.028560316	0.51337147	0.607880064
KIAA0999	-0.113633722	0.009096454	0.019123654
GYPA	0.004703021	0.914307979	0.940209263
TAC1	0.137296853	0.001597685	0.004045568
TRAIP	0.032286332	0.459962838	0.557302757

KIAA0232	0.406425356	2.43E-22	1.78E-20
ERCC8	-0.034994319	0.423178072	0.521676417
GPX4	0.311659909	2.60E-13	4.03E-12
KIAA0368	0.032977159	0.45041287	0.547890947
GPR157	0.116079642	0.007700409	0.016452333
CTAGE4	0.121362328	0.005318301	0.011835769
C9orf30	-0.366716946	3.46E-18	1.38E-16
OR52A1	-0.214429367	6.90E-07	3.51E-06
HSP90B3P	-0.090349327	0.03831749	0.068532357
ALG9	-0.105012914	0.015979918	0.031645898
BTBD10	0.15690817	0.000303283	0.000892719
SDK2	-0.017616948	0.686867823	0.762511328
BAIAP3	0.428812843	6.11E-25	7.22E-23
RABGGTB	-0.073076693	0.094082833	0.149255763
ANKRD40	0.197070495	5.27E-06	2.25E-05
KRT74	0.018840454	0.666687754	0.745356909
CALCOCO2	0.321800729	3.88E-14	7.16E-13
SNCA	-0.182154938	2.63E-05	9.59E-05
TMSL8	-0.351566884	9.52E-17	2.93E-15
C2orf53	0.036545119	0.402909436	0.501516219
ESRRB	-0.01539323	0.724673329	0.794157245
ARHGAP26	-0.126515965	0.003656276	0.008512924
TDRD9	0.046539756	0.286689108	0.379462137
HRAS	0.263523467	8.34E-10	7.02E-09
KLRC4	-0.084167063	0.053708844	0.091382313
JAGN1	0.044062818	0.313141155	0.407686844
BSDC1	0.097564874	0.025244708	0.047398384
RNF43	0.143022272	0.001004394	0.002666676
NDUFAF1	0.24773881	8.50E-09	6.05E-08
PHF12	0.049801884	0.254210878	0.342279019
OR1L3	0.001673256	0.969460978	0.977088273
FOLR2	-0.065968795	0.130783168	0.196732369
LYZL6	-0.00830344	0.84931929	0.895330759
tcag7.1260	-0.194174741	7.27E-06	3.00E-05
WSB1	-0.080370548	0.065494338	0.108521876
PROS1	-0.166235836	0.00012805	0.00040978
OSTN	0.045165896	0.301169287	0.39578755
PSMB8	-0.028473932	0.514646774	0.608686865
SOCS4	-0.075387778	0.084106493	0.135278793
DDIT4L	-0.138526813	0.001448144	0.003711813
MAS1	0.023083918	0.599798817	0.685532142
MGC34796	0.459432205	7.94E-29	2.12E-26
CSHL1	-0.067782507	0.120504744	0.183821302
TBCCD1	-0.097114357	0.025930192	0.048581581
ZBTB7C	0.102169501	0.01908905	0.037145116
AP2S1	0.113633036	0.009096876	0.019123654
P15RS	-0.1105649	0.011163999	0.022982065
VAT1	0.219544203	3.67E-07	1.96E-06
SHANK3	-0.073820438	0.090773928	0.144903656
TUFM	0.193249888	8.05E-06	3.28E-05
THEG	-0.013922067	0.750064653	0.815443344

KRT34	-0.114542462	0.008553438	0.018117496
SGSM3	-0.078580471	0.071747581	0.117772524
TOMM22	-0.274121576	1.61E-10	1.52E-09
SOCS3	-0.035965019	0.410422385	0.508500352
CPO	0.064587024	0.139059034	0.206939496
POP4	-0.037261763	0.39374244	0.491998022
BHLHB3	-0.210252193	1.14E-06	5.58E-06
MALL	-0.271312202	2.50E-10	2.29E-09
OR1B1	-0.007295203	0.867435039	0.907890733
PARK2	-0.03939993	0.367149498	0.465293129
GPR124	0.032419636	0.458111291	0.555387683
LCE1E	0.033638472	0.441813309	0.539672236
RUVBL2	0.201168023	3.31E-06	1.47E-05
CGRRF1	0.138322611	0.001472046	0.003765229
ACPL2	-0.153771617	0.000401058	0.00114596
WNT10B	-0.109291621	0.012137429	0.024729307
BAIAP2L2	-0.277719392	9.04E-11	8.97E-10
ISCA1	-0.041880906	0.337729481	0.433911112
C1orf125	0.042667834	0.328722493	0.424467579
RPAP1	0.129521042	0.002920323	0.006981752
RAI16	0.109754301	0.011775357	0.024095398
RPL27	-0.204609946	2.23E-06	1.02E-05
NLRP9	-0.071904945	0.099490816	0.15634271
EPN1	0.122275999	0.004981385	0.01118311
LOC388610	-0.214100935	7.19E-07	3.63E-06
SLC35A1	-0.032466263	0.457464649	0.55482251
GAL	-0.243575139	1.53E-08	1.04E-07
SLC14A2	-0.004900067	0.910732363	0.937357397
RDH11	0.102322868	0.018908793	0.036817659
FAM138F	-0.101703673	0.019645766	0.038083801
AUH	0.059577605	0.172455754	0.248725895
FLJ40243	-0.001991587	0.963654729	0.973950186
C14orf129	0.056361216	0.196850103	0.277216263
MBD2	0.072355999	0.097380492	0.15377315
ABHD14B	0.223587361	2.20E-07	1.23E-06
PIGT	0.353556578	6.22E-17	2.00E-15
ALS2CR4	-0.40799389	1.62E-22	1.25E-20
ALAS1	-0.132656502	0.002298478	0.005621853
FOXO1	-0.012367689	0.777188244	0.838997632
CRLF3	-0.278709833	7.70E-11	7.75E-10
C20orf107	0.160946375	0.000210006	0.000638007
FARS2	0.038773256	0.374825482	0.472702993
CCDC28A	-0.226296859	1.56E-07	8.84E-07
NPHP3	-0.178480079	3.84E-05	0.000136028
OR13F1	-0.001264075	0.976926636	0.982519117
TSEN54	0.162787598	0.000177088	0.000546643
DEFB106B	-0.063392591	0.146531869	0.216488338
OR8B4	0.096407599	0.027184542	0.050653863
STH	0.278668722	7.76E-11	7.79E-10
ZC3H14	-0.189880032	1.16E-05	4.60E-05
CBX2	0.074491351	0.08786974	0.140852719

TMEM49	0.238808653	2.95E-08	1.88E-07
C6orf21	0.040801109	0.350343346	0.44815087
FLJ20920	0.141484663	0.001139653	0.002985824
CRTAP	0.151233876	0.000500875	0.00140337
DDX50	-0.314698713	1.48E-13	2.46E-12
STYXL1	0.203720101	2.47E-06	1.12E-05
BLVRB	0.441150077	1.85E-26	3.08E-24
LOC147650	0.006267385	0.885974306	0.919469368
MMP24	0.19249912	8.75E-06	3.53E-05
GRID1	0.046248605	0.289717948	0.382701539
BANF1	0.121268909	0.00535388	0.011906332
CTAGEP	0.214453913	6.88E-07	3.50E-06
HMBS	-0.128868712	0.003067539	0.007288368
SLC25A24	0.053192205	0.223259404	0.307187755
C14orf50	0.173542057	6.31E-05	0.00021387
MRO	0.084483631	0.052811463	0.090029854
SLC25A15	-0.073527135	0.092067504	0.146588058
FAM84B	0.184215372	2.13E-05	7.90E-05
TDP1	-0.183572064	2.27E-05	8.41E-05
C16orf78	-0.034716828	0.42686659	0.525065545
C11orf57	-0.115556631	0.007981706	0.016976656
RFK	0.108098618	0.01311678	0.026556442
ZFYVE9	-0.127547704	0.003386518	0.007952903
STCH	-0.063933441	0.143111005	0.212250258
WIBG	0.379101263	2.01E-19	9.60E-18
LOC283871	0.351203955	1.03E-16	3.15E-15
GBA2	-0.099294627	0.022756111	0.043294346
NDUFB3	-0.068502448	0.116604033	0.178535408
HSD17B13	-0.026197965	0.548829059	0.640305766
GRIN3A	-0.163512055	0.000165515	0.000514276
FMNL1	-0.140549604	0.00122992	0.003197793
SEPT7	-0.081922986	0.060440654	0.101349763
GNLY	-0.117024345	0.007214682	0.015533078
GRAMD1C	0.060005067	0.169391379	0.245137113
ZNF165	-0.012529728	0.774347382	0.836077621
USP38	0.249369588	6.73E-09	4.91E-08
FAM83A	0.121392564	0.00530683	0.011814519
C14orf24	0.286781501	2.04E-11	2.25E-10
ARMCX3	0.016777262	0.701057814	0.774349649
ARHGDIB	-0.030599283	0.48375278	0.579730237
AK1	0.265420375	6.25E-10	5.38E-09
KIAA1045	-0.274877404	1.43E-10	1.37E-09
DNAJB13	-0.029927486	0.493408218	0.587998085
NEU2	0.007803316	0.858564183	0.901675689
HIST1H4B	-0.007636207	0.861299709	0.903620363
FAM20B	-0.06367048	0.144766516	0.214343681
HES2	0.118306646	0.006599277	0.014379503
FAM73B	0.143744163	0.000946146	0.002520734
LOC388381	-0.075014546	0.085657224	0.137629023
INTS7	-0.129656823	0.002890501	0.006915832
AMPH	0.167500197	0.000113513	0.000366594

ZNF775	0.116744497	0.007355628	0.015803549
UCKL1	0.263199527	8.76E-10	7.33E-09
C10orf97	-0.047579934	0.276042803	0.367082006
C1orf161	-0.069567052	0.111017991	0.171563113
ALDH1L1	-0.085654094	0.049601333	0.085492371
FLJ39378	0.15677898	0.000306826	0.000902283
SLC23A1	0.210543089	1.10E-06	5.41E-06
RBM4B	-0.092137739	0.03463341	0.062561938
THAP4	0.056127588	0.198715527	0.279291834
OGFRL1	-0.390244239	1.40E-20	8.37E-19
KIAA0831	0.041086884	0.346976372	0.444121089
PPP1R15A	0.006709574	0.877990088	0.914409832
C1orf96	-0.184053828	2.16E-05	8.02E-05
C12orf11	-0.321639457	4.00E-14	7.32E-13
BMF	0.045762344	0.294824251	0.388611858
MAN1A1	0.035668752	0.414291187	0.512777075
KIAA1600	-0.129786702	0.002862234	0.006850867
NLGN4X	-0.069649345	0.110595121	0.170995575
ALOX12	-0.115352457	0.008093986	0.017209515
RB1CC1	0.069115082	0.113363218	0.174442048
NEIL2	-0.00583044	0.893875186	0.923926462
EIF4E	-0.001751763	0.968028877	0.976445139
ABHD5	-0.044922713	0.303782109	0.39862488
EXOC4	0.102965528	0.0181695	0.035558325
CIP29	0.057681512	0.186547955	0.265165342
BATF2	-0.022649578	0.604254748	0.689599563
SLC29A4	-0.117465235	0.006997525	0.015128886
HTR4	-0.009248049	0.832418276	0.881746766
EMB	0.008202845	0.851123364	0.896617709
TRAF6	-0.209035155	1.32E-06	6.36E-06
LMNB1	-0.042689912	0.328472053	0.424322406
FAM19A5	-0.012798765	0.769637288	0.832307366
SHE	-0.039977473	0.360162487	0.458612369
PIK3C2B	0.115601256	0.007957352	0.016930712
C15orf15	-0.18432802	2.10E-05	7.83E-05
USP15	0.030801232	0.480870367	0.576725549
TCEAL2	-0.176680757	4.61E-05	0.000160873
C5orf39	-0.231027405	8.39E-08	4.96E-07
PTGER2	-0.004919051	0.910387967	0.937219465
SLC31A1	-0.155455698	0.000345407	0.001002789
IFT172	-0.22189583	2.73E-07	1.48E-06
ADAM29	0.246190219	1.06E-08	7.40E-08
GFOD1	-0.198063933	4.71E-06	2.03E-05
ST7L	-0.062273602	0.153807026	0.225556738
C15orf26	0.086197066	0.048168529	0.083456829
PKN3	-0.086645767	0.047010806	0.081658035
CNTD1	0.252538745	4.26E-09	3.21E-08
COMMD1	-0.137347495	0.001591258	0.004031581
NTRK2	-0.158230416	0.000269156	0.000797993
FOXN3	-0.191245381	1.00E-05	4.00E-05
MFGE8	-0.252350145	4.38E-09	3.29E-08

PFKFB2	0.059608659	0.172231751	0.248604
TAS2R4	0.051114523	0.241891567	0.327619218
ENTHD1	-0.085350141	0.050418855	0.086575129
PRMT5	0.038855644	0.373810718	0.471887108
MGC16384	0.067458835	0.122291286	0.185900895
LOC442229	-0.155649312	0.000339493	0.000986618
TSKU	0.117145272	0.007154528	0.015430794
KRTCAP3	0.00415884	0.924191544	0.94698447
PDLIM1	-0.038942556	0.372742083	0.471118411
KCNS2	-0.024201542	0.579705131	0.667154566
RNF126	0.037793685	0.387020457	0.485987239
CEP63	0.114169167	0.008772902	0.018537654
CLIC4	-0.296050415	4.22E-12	5.17E-11
hCG_1990170	-0.311878695	2.50E-13	3.88E-12
ACR	-0.147537025	0.000688147	0.001879795
KLK7	-0.204523385	2.25E-06	1.03E-05
ALOX5AP	-0.184657052	2.03E-05	7.58E-05
RIPK3	0.222002266	2.69E-07	1.47E-06
TAS2R9	-0.163931943	0.000159136	0.000496968
C19orf18	0.126242221	0.003731046	0.008657436
BIRC6	-0.062011899	0.155547351	0.227457945
ZNF16	0.117448493	0.007005664	0.015136271
RFT1	0.109477717	0.011990632	0.024487013
SLC8A2	0.079074276	0.069975727	0.115171505
TACC1	0.15324775	0.000420005	0.0011951
ITGAD	0.041937066	0.337081498	0.433350226
SAMHD1	-0.061743736	0.157346054	0.229760362
SH3PXD2B	-0.143864499	0.000936746	0.002497853
EPC2	-0.247851263	8.36E-09	5.97E-08
C20orf85	0.175731231	5.07E-05	0.000176167
ATP13A2	0.187844175	1.45E-05	5.60E-05
KRT4	-0.093349671	0.032312099	0.058813865
CAPNS1	0.24201787	1.90E-08	1.26E-07
MDM2	0.279194786	7.12E-11	7.20E-10
PCDH20	-0.021089865	0.629385118	0.711675081
KCNK9	0.220318144	3.33E-07	1.79E-06
OR2C1	0.081249317	0.062592623	0.10433235
KLHDC3	-0.224900722	1.86E-07	1.05E-06
IPPK	0.165800067	0.000133453	0.000424963
EFHD2	0.076309626	0.080373297	0.129783457
GALR3	0.068648267	0.115826149	0.177521183
NBEA	0.222182053	2.63E-07	1.44E-06
ABCA6	0.016038052	0.713636709	0.784859976
CLDN3	0.304683508	9.22E-13	1.29E-11
AKT2	0.042158338	0.334536183	0.430473402
EGFR	-0.387072497	3.03E-20	1.65E-18
RBM16	-0.089545224	0.040079196	0.07122745
ZDHHC3	0.112295374	0.009952089	0.020730146
SLC25A4	0.170966904	8.12E-05	0.000268624
CYB5B	-0.059519201	0.172877631	0.249068545
CPXM1	0.008331678	0.848813009	0.89510396

NDRG1	-0.166821489	0.000121112	0.000389089
FLJ43826	0.101047904	0.020453425	0.039376366
OR5L2	0.119358266	0.006130171	0.013471915
FARP2	0.300141484	2.07E-12	2.70E-11
MRPL46	0.0720343	0.098881922	0.155692671
LDHAL6B	0.009113663	0.834818201	0.883223867
MAPKAPK3	0.046633999	0.285713287	0.378468548
NCAM2	0.113336939	0.00928033	0.019442844
PRKD2	0.019700873	0.652132091	0.732688083
ZFP36L1	-0.165358388	0.000139148	0.000440587
CYSLTR1	-0.011349018	0.795114208	0.853553992
OR4C3	0.071875727	0.099628761	0.156479503
HIST1H2AJ	0.455562248	2.59E-28	6.11E-26
CCNB2	-0.086587189	0.047160609	0.081895111
ZNF10	-0.217412407	4.78E-07	2.52E-06
TMEM175	0.194167057	7.28E-06	3.00E-05
FAM134A	0.26770931	4.39E-10	3.89E-09
TIGD4	0.059552908	0.172634059	0.248834231
PCNP	-0.191890146	9.35E-06	3.75E-05
MGC39715	-0.023584776	0.58940548	0.676040719
LQK1	-0.071639604	0.100749147	0.158037373
CREB1	-0.103466474	0.017610858	0.03455302
TMPRSS3	-0.198698701	4.39E-06	1.90E-05
C4orf32	0.245547782	1.16E-08	8.04E-08
LAT	-0.200419197	3.61E-06	1.59E-05
KCNA3	-0.104586098	0.016416268	0.032443165
SKIV2L2	0.056474019	0.195954013	0.27629471
ROPN1B	-0.415212388	2.45E-23	2.09E-21
tcag7.23	-0.192458598	8.79E-06	3.54E-05
CDT1	-0.154052177	0.000391241	0.001120514
ZHX2	0.043905669	0.314871799	0.409593123
CD28	-0.15435472	0.000380907	0.001093473
ZNF624	0.113254643	0.0093319	0.019537573
SEPT2	-0.056708267	0.194102699	0.274063108
SOHLH2	-0.062447714	0.152657377	0.224138064
MCOLN3	0.002477392	0.954797603	0.967837739
UNQ1945	0.006276741	0.885805247	0.9194491
MASP2	0.146442528	0.000754956	0.002046835
ZNRF3	0.130368996	0.002738574	0.00658822
GPATCH3	-0.080553646	0.064880791	0.107621253
AGL	0.065558601	0.133199179	0.199680413
QRICH2	-0.086100019	0.048422041	0.083825206
PSD4	0.125311359	0.003995788	0.009216091
CCNB1IP1	-0.353383791	6.46E-17	2.07E-15
ENPP7	0.142535833	0.001045492	0.002761482
OBFC1	0.124746393	0.004164646	0.009566084
KCNG3	-0.017484834	0.689093305	0.764705781
C14orf79	0.264790914	6.88E-10	5.86E-09
ENPEP	0.061295932	0.160384681	0.233587258
SCT	0.100470784	0.021187953	0.040650459
SKI	-0.125199073	0.004028846	0.009285373

SEC61G	0.007148512	0.870076785	0.909749272
CAPN11	0.03485949	0.424967946	0.523253485
ATXN7L3	0.086343683	0.047787633	0.082866936
DBNDD1	0.212988219	8.23E-07	4.13E-06
FAIM	-0.170637674	8.39E-05	0.000276193
ANKRD36	0.002523388	0.953959289	0.967331079
GABRP	-0.289369878	1.32E-11	1.51E-10
TACSTD2	0.033641162	0.44134021	0.539308616
EIF3J	0.042677971	0.328607491	0.424408204
PPP2R2A	-0.126230903	0.003734168	0.008658964
TEKT4	0.124482269	0.00424578	0.009733024
PVALB	0.170847977	8.22E-05	0.000271319
F10	0.004606432	0.916061345	0.940691584
FAM134C	0.243004763	1.65E-08	1.12E-07
COMP	0.160036058	0.000228321	0.000689222
EFCBP1	0.030660194	0.482882417	0.578991729
SCLT1	-0.137829539	0.00153126	0.003894156
TAL1	0.031824196	0.466413958	0.562679896
ACSL1	-0.069469031	0.11152334	0.172227874
ABCC5	0.313521877	1.84E-13	2.97E-12
ABL1	0.078405428	0.072384394	0.118691104
RBBP7	0.128641599	0.003120356	0.007396711
PTPRG	0.094195711	0.03077155	0.056364093
NCOR1	0.007130883	0.870394367	0.909749272
SPINK4	0.329842025	8.15E-15	1.71E-13
TXNRD1	-0.033823797	0.438863197	0.536708393
TNRC15	0.127099386	0.003501434	0.008198902
C9orf138	0.071413341	0.101832117	0.15945141
UBE2H	0.338939722	1.32E-15	3.26E-14
BRDT	0.055169513	0.206500166	0.288387354
C8orf31	-0.017260299	0.692881799	0.76697213
CCNE2	0.016187342	0.711089903	0.782899161
SLC6A8	-0.01875061	0.667883874	0.746049371
CALCR	0.123992636	0.004399978	0.010039134
PPP1CB	-0.339658405	1.14E-15	2.83E-14
ABHD8	0.00362647	0.933871916	0.953309848
ARF5	0.096090012	0.027548752	0.051208367
SLC24A4	-0.204813492	2.17E-06	9.97E-06
CCT3	0.153868604	0.000397639	0.001136719
ZNF121	-0.132614961	0.002305857	0.00563542
SLC3A2	0.115757158	0.007872787	0.016768192
OR13A1	0.185762433	1.81E-05	6.83E-05
SLC5A10	0.250321936	5.87E-09	4.33E-08
RAD50	0.273555688	1.76E-10	1.65E-09
IER5	0.117019484	0.007217109	0.015533078
MTHFD1L	-0.360126296	1.50E-17	5.22E-16
MBTPS2	0.174190929	5.91E-05	0.00020222
MVK	0.240980677	2.19E-08	1.44E-07
NCL	-0.153286516	0.000418575	0.001192134
PSMD10	0.025873125	0.553797341	0.645187543
MOBP	0.073964481	0.090144004	0.144085127

FLJ32894	-0.010968375	0.8023977	0.858823926
HRH1	-0.009512696	0.827696661	0.878106758
C5orf30	0.306181124	7.04E-13	1.01E-11
NUDT16L1	0.300235866	2.03E-12	2.67E-11
RASGRP3	-0.230637396	8.83E-08	5.19E-07
PRKRIP1	0.147698707	0.000678756	0.001856615
CCDC75	-0.049734777	0.254852178	0.34290723
LOC253970	-0.017409528	0.690363051	0.7655622
KIAA1239	-0.04547668	0.29785192	0.392192748
MED21	-0.123276539	0.004634618	0.010488871
SYT11	-0.129814934	0.002856123	0.006838903
NTSR2	-0.162011129	0.000190332	0.000583133
EGFL11	0.039490119	0.368351979	0.46654065
CXorf59	0.038281621	0.383659236	0.482536437
OR2A25	-0.02472576	0.571519505	0.659833541
SPTBN2	-0.05178613	0.235753066	0.320931061
LRMP	-0.236608739	3.98E-08	2.48E-07
RNF111	0.096553888	0.026805325	0.050053429
PTH	-0.035370171	0.419102392	0.518106275
LOC619208	-0.171050718	8.06E-05	0.000266723
KIAA0895	-0.01941498	0.656855263	0.736641075
RANBP5	-0.326440953	1.59E-14	3.13E-13
P2RY10	-0.108039312	0.013167234	0.026633329
NME5	0.085099391	0.051101727	0.087591768
DDX21	-0.20510838	2.10E-06	9.68E-06
LRSAM1	0.341705959	7.49E-16	1.94E-14
HDAC11	0.431753372	2.69E-25	3.59E-23
VMO1	-0.053217722	0.223037121	0.30701931
NOLA2	0.253193092	3.88E-09	2.94E-08
ADAR	0.059851642	0.170486533	0.246489935
MTO1	-0.293244315	6.84E-12	8.24E-11
SF4	0.007604914	0.861862385	0.903904452
P2RX1	-0.110807646	0.010986472	0.022669736
HBM	0.021463301	0.62332769	0.706644906
EN2	0.076490671	0.079656131	0.12876066
C14orf172	0.05578378	0.201484079	0.282537195
TM9SF2	-0.090902282	0.037144487	0.066569937
INHBE	-0.028289689	0.517372264	0.611087601
TCTE3	-0.243836479	1.47E-08	1.01E-07
TOX2	-0.23321669	6.27E-08	3.79E-07
CTAGE3	0.188857426	1.30E-05	5.08E-05
HBB	-0.003771887	0.931226692	0.951423592
MED15	-0.024513565	0.574826313	0.663269495
CASR	0.003623251	0.93393048	0.953309848
C6orf66	-0.343889587	4.77E-16	1.29E-14
MTPN	0.058833963	0.177884972	0.254849648
UNC50	-0.065328386	0.134570141	0.201576565
C21orf33	0.035176637	0.42076479	0.519639023
IRF2	-0.011453568	0.793269249	0.852317423
PGR	0.266972259	4.92E-10	4.33E-09
GPR84	-0.207355542	1.61E-06	7.59E-06

CROCCL1	-0.128932757	0.003052792	0.007256134
SRPX	-0.067718546	0.120856158	0.184103444
BRE	0.021130444	0.628725687	0.711321849
FGF10	-0.000392399	0.992836584	0.994940051
SDC3	-0.160427346	0.000220273	0.00066722
ZRSR1	0.108328523	0.0129228	0.026207882
DKFZP434P211	0.176752128	4.58E-05	0.000159902
SOX6	-0.150783373	0.00056088	0.00155634
RPUSD2	0.007772919	0.858842303	0.90181375
C14orf173	0.061819289	0.156837699	0.229126873
MAPK11	0.011995261	0.783728859	0.844427677
TBC1D22A	-0.033310031	0.445851546	0.543202131
FAM123A	-0.016016157	0.714010491	0.784925894
COL4A6	0.047710487	0.274725822	0.365567859
TOMM70A	-0.023034706	0.59811932	0.684503201
NAB1	-0.358843292	1.98E-17	6.77E-16
MGC16385	-0.098889452	0.023319138	0.044215042
TSPAN18	0.013949891	0.749581822	0.815062533
MED31	0.094348473	0.030500195	0.055950388
PLG	-0.006132369	0.888414511	0.920984352
CAPSL	0.173915321	6.08E-05	0.000206792
ZNF532	-0.323854309	2.62E-14	5.04E-13
ASB14	-0.000552922	0.989906281	0.99265064
CA8	0.014666039	0.737188439	0.80418333
NUDT16P	0.167089548	0.000118055	0.000380263
SLFN11	-0.240032604	2.49E-08	1.62E-07
LRRIQ2	0.033505029	0.44319173	0.540926151
NOL7	0.031577597	0.469876704	0.566301813
BRMS1L	-0.218964883	3.94E-07	2.10E-06
JARID1A	-0.139336389	0.001356854	0.003492379
PANK2	0.015703583	0.71935412	0.789735491
ICAM3	0.023896042	0.58450063	0.671416846
MDS1	0.116629614	0.007414199	0.015918264
TAF8	0.033419686	0.444354709	0.541947412
RNF139	0.331947985	5.38E-15	1.17E-13
ZNF594	-0.103577892	0.017488668	0.034346159
ADAM8	-0.031360576	0.472935799	0.569207718
SFTPC	-0.079965204	0.066869587	0.110621762
MAN2B2	0.312472653	2.24E-13	3.55E-12
RGS12	0.274719522	1.46E-10	1.40E-09
EIF1AY	0.020730552	0.635237042	0.717236976
LRRIQ1	0.004064912	0.925898719	0.947470664
GPR150	0.08553731	0.049914116	0.085972521
CCDC21	0.033514098	0.443068244	0.540882794
PRRG3	-0.160215053	0.000224606	0.000679102
SAA4	-0.244184455	1.40E-08	9.61E-08
RAPGEF5	0.005879004	0.892996524	0.923629952
ZCCHC2	-0.078083489	0.073567667	0.120388325
MGC39372	0.004148025	0.924388109	0.947028072
PPP4R2	0.028436496	0.515199958	0.609223951
CDCA2	-0.207483751	1.59E-06	7.49E-06

OR4D5	-0.209110862	1.31E-06	6.31E-06
PTGFRN	-0.08393273	0.05438126	0.092449646
SIGLEC5	-0.148980436	0.000608405	0.001679876
C19orf61	-0.0225782	0.605394942	0.690260244
NMUR2	-0.038014368	0.384706754	0.483754975
KIAA1586	-0.289115922	1.38E-11	1.57E-10
DAGLA	0.078553041	0.071847066	0.117904352
CHCHD6	0.116007378	0.00773874	0.016522748
GPR32	0.08104896	0.063244701	0.105219606
NEUROD6	0.023883791	0.584693326	0.671497236
SLC2A4RG	0.349987527	1.33E-16	3.99E-15
CA5B	-0.005847288	0.89357034	0.923766648
FBXL3	-0.073096835	0.093991964	0.149150087
MPHOSPH9	0.02811535	0.519958103	0.613287739
HMG2L1	-0.099204088	0.022880891	0.043502875
HCN4	0.09522646	0.028980026	0.053480847
CEACAM19	-0.134295539	0.002023968	0.004994133
SH2D4B	-0.207895934	1.51E-06	7.18E-06
HFE2	-0.011915684	0.785128393	0.845639252
TGM4	0.027793078	0.524755541	0.617554416
LYPD2	0.025036069	0.566700036	0.656128558
TBC1D15	0.054649606	0.210815847	0.293016873
MRPS21	-0.171781298	7.50E-05	0.000249948
NONO	-0.119915768	0.005893824	0.013017645
CLEC5A	-0.192406845	8.84E-06	3.56E-05
ITCH	-0.0917396	0.035426355	0.063844273
MGAT3	-0.287857967	1.71E-11	1.90E-10
MBP	-0.282299564	4.29E-11	4.47E-10
RPP25	-0.038844057	0.373953333	0.471970247
SOSTDC1	-0.310513845	3.21E-13	4.84E-12
HRC	0.271302051	2.51E-10	2.29E-09
TRIM48	-0.094209557	0.030746869	0.056335667
TMEM133	-0.300548871	1.92E-12	2.54E-11
ECEL1P2	-0.016071107	0.713072537	0.784520134
HOXC11	0.054341003	0.213408097	0.295950922
DOK5	-0.265425966	6.24E-10	5.38E-09
HELZ	0.211409583	9.96E-07	4.91E-06
LOC348180	-0.136046321	0.001764144	0.004413516
MGC33894	0.117937698	0.006771332	0.014707497
ADRB3	0.071130691	0.103197937	0.161271336
DMD	-0.337293613	1.84E-15	4.37E-14
PTRH2	0.169172252	9.67E-05	0.000315154
MPEG1	-0.155001628	0.000359657	0.001040719
NDUFA12	0.19928412	4.11E-06	1.79E-05
KRTAP2-4	0.05263878	0.22811928	0.312476154
STAMBPL1	-0.233164755	6.32E-08	3.80E-07
ADCY2	-0.226848482	1.45E-07	8.27E-07
UNQ6125	0.028294236	0.517304916	0.611087601
KLHL20	0.148327978	0.000643325	0.001768352
SRM	-0.137293631	0.001598095	0.004045568
OTC	-0.093500958	0.03203188	0.058394317

TMIE	-0.073187571	0.093583506	0.148616988
SNX8	-0.018192597	0.677202646	0.753686709
LIPK	-0.031656084	0.469623614	0.566107745
CHURC1	0.198903573	4.29E-06	1.87E-05
KLC2	0.241671766	1.99E-08	1.32E-07
HDAC1	-0.007362071	0.866231321	0.907000858
FAM128A	0.383136223	7.77E-20	3.95E-18
FNDC3B	-0.308168552	4.92E-13	7.20E-12
MTCP1	-0.090554625	0.037878369	0.06780614
WFDC10B	0.158253035	0.000268604	0.000796847
PCDHGB3	0.099183845	0.022908871	0.043528415
ATRNL1	0.035039506	0.422579192	0.521236408
CAV2	-0.193364988	7.95E-06	3.24E-05
MED26	-0.211580487	9.75E-07	4.82E-06
DUS1L	0.061706828	0.157594844	0.230069016
CHRM3	-0.224196274	2.04E-07	1.14E-06
NEK9	0.143497704	0.000965672	0.002570528
WARS2	-0.240708661	2.27E-08	1.49E-07
TBX22	-0.025011029	0.569318333	0.658157254
TOMM40	-0.032445452	0.457753203	0.555062995
RP6-213H19.1	-0.058687646	0.178967989	0.256162516
TUBGCP5	0.009773511	0.823049476	0.874534513
IGSF6	-0.001945522	0.964494824	0.974367362
TPPP	0.203361072	2.57E-06	1.16E-05
UNQ6190	-0.044727946	0.307275477	0.402093404
GSTM5	0.066677876	0.126686836	0.19144688
BTD	0.10754425	0.01359512	0.027390692
PDCD1LG2	-0.198250746	4.62E-06	1.99E-05
SNRPB2	0.093455446	0.03211596	0.058530242
ERICH1	-0.07203913	0.098859243	0.155692671
APOA4	0.0195372	0.654834415	0.735045056
HOXA11	-0.126868734	0.003561924	0.008315213
NARG1	0.019401651	0.657075819	0.736685689
MKX	0.050839788	0.244434693	0.330699434
RAB28	-0.01814967	0.677921591	0.754213834
PKP3	0.187480282	1.50E-05	5.78E-05
SH3GL2	-0.193390801	7.93E-06	3.24E-05
CTSO	0.094876112	0.029578646	0.054487446
RPN2	0.193953593	7.45E-06	3.07E-05
IL28RA	-0.141436025	0.001144194	0.002993893
SFMBT1	0.04358127	0.318464116	0.413915842
WDR57	-0.123847349	0.004446697	0.010126941
FER1L3	0.279017384	7.33E-11	7.40E-10
HSF5	-0.065200693	0.13533525	0.202476022
TTC9B	0.25029459	5.90E-09	4.34E-08
C4BPA	-0.152008265	0.000468202	0.001319421
ALB	0.025790173	0.555069561	0.646302354
SORBS3	0.090231559	0.03857133	0.06887787
UPF2	-0.222688804	2.47E-07	1.36E-06
JPH1	0.062946265	0.149401559	0.220146223
AGBL2	0.206630754	1.76E-06	8.20E-06

DOPEY1	-0.271538625	2.42E-10	2.21E-09
TERF1	0.069343324	0.112174076	0.173001854
KIF22	0.276904057	1.03E-10	1.01E-09
NINJ1	0.316286074	1.10E-13	1.90E-12
SEC61A2	0.045097721	0.30190026	0.396578658
HIST1H1D	0.198727369	4.37E-06	1.90E-05
SFXN4	0.030441411	0.48601257	0.581758087
UCP3	-0.151515289	0.000488764	0.001373588
ZNF703	0.476014857	4.22E-31	1.62E-28
MYL6B	-0.169417042	9.44E-05	0.000308114
TREM1	-0.055189497	0.206335564	0.28822294
OR52E6	0.120448011	0.005675861	0.012558786
CKMT2	-0.165898001	0.000132221	0.000421912
HLA-C	-0.085736742	0.049380966	0.085197407
SLC13A3	-0.107787273	0.013383566	0.027026452
TIMP4	0.009357167	0.830470739	0.880290394
SLIT2	0.034706568	0.427003321	0.525102124
RSF1	-0.077030289	0.077549286	0.12601759
LONRF1	-0.149956788	0.000559424	0.001553
MON1A	-0.004681383	0.914700738	0.940209263
CACNG6	0.247070503	9.34E-09	6.60E-08
DPPA4	0.002887266	0.947329137	0.962830886
ZSWIM3	-0.019307499	0.658634505	0.737963478
ZNF804A	-0.019014655	0.663491977	0.742384166
CCIN	-0.035927171	0.410915421	0.509008647
SLC25A31	0.018239578	0.676416136	0.753430525
KCNMB4	-0.023692013	0.587713536	0.674225845
RABL5	0.240045611	2.49E-08	1.62E-07
GALNS	0.057319357	0.189333448	0.268251468
STX6	0.002284031	0.958322334	0.969995725
HIST1H1C	0.322656487	3.29E-14	6.19E-13
CIDEB	-0.205544581	2.00E-06	9.23E-06
CASP4	-0.163006201	0.000173518	0.000536702
PDK3	-0.017621569	0.68679003	0.762511328
KCNJ11	0.224961232	1.85E-07	1.04E-06
TPR	-0.084660285	0.052316153	0.089309279
ZSCAN20	-0.271879116	2.29E-10	2.11E-09
MTX2	0.065572032	0.133119529	0.199646825
HIST1H2BH	0.207290264	1.63E-06	7.63E-06
LOC283767	0.01616768	0.711425135	0.783128026
LYRM7	-0.054047065	0.215898407	0.298932516
BRD3	0.126675855	0.003613234	0.008425399
HIST1H2BO	0.21975064	3.58E-07	1.92E-06
MAGEB10	0.044486098	0.308510798	0.40311072
SLC45A1	0.097766101	0.024943619	0.046890344
SERPINA3	0.201109029	3.34E-06	1.48E-05
KIAA0143	0.256962435	2.23E-09	1.76E-08
KCNJ16	-0.272267281	2.16E-10	2.00E-09
KRT79	0.042155937	0.334563732	0.430473402
FABP2	-0.082312557	0.05922436	0.099609024
NUT	-0.210186336	1.15E-06	5.61E-06

ZNF57	0.029054723	0.506104121	0.60043107
FBXL4	-0.130836913	0.002642752	0.006380165
CLEC9A	-0.213534335	7.70E-07	3.88E-06
UGT8	-0.420514032	5.92E-24	6.07E-22
BMP2K	-0.131587955	0.002495323	0.006071919
MAPK4	-0.451458489	8.91E-28	1.96E-25
SLC25A23	0.106425904	0.014607074	0.029218899
HINT1	-0.014749604	0.735746661	0.803999683
KRTAP13-1	0.006023091	0.890390308	0.921718856
SFXN5	0.258289799	1.83E-09	1.47E-08
CHCHD2	0.159079746	0.000249165	0.000743744
FAM3D	-0.258132661	1.88E-09	1.50E-08
NDP	-0.13093695	0.002622667	0.006341635
RHOBTB1	0.000287746	0.994747041	0.996367414
SLC4A4	-0.187599919	1.49E-05	5.73E-05
RPL38	-0.033979229	0.436761409	0.534669701
HTF9C	-0.05216715	0.232319802	0.316888745
AP2A2	0.121708151	0.005188437	0.01158304
ZBTB46	0.055878849	0.200715737	0.281742741
MAP7D1	-0.027912373	0.522977045	0.615835502
AOX1	0.004171058	0.923969516	0.946914759
CYR61	-0.094486148	0.030257399	0.055621149
DTNA	0.164058123	0.000157265	0.000491873
JRKL	-0.428272481	7.09E-25	8.08E-23
TMOD3	0.133084711	0.002223656	0.005460567
EEA1	0.074278797	0.088781627	0.142203236
ADCK5	0.242094876	1.88E-08	1.25E-07
IL1R1	-0.063628815	0.145030159	0.214665412
KLK3	0.069981152	0.108902952	0.168718632
HRSP12	0.076507746	0.079588759	0.128685585
KTN1	0.162034021	0.000189928	0.000582187
LOH11CR2A	-0.087418185	0.045072527	0.07871371
RELL2	0.113725194	0.00904044	0.019024525
MAB21L1	-0.025347166	0.561887913	0.652388365
C20orf59	0.177566152	4.22E-05	0.000148215
PHKB	0.070693653	0.105338332	0.164199051
ADAM2	0.193135734	8.16E-06	3.32E-05
TBC1D8B	-0.049910499	0.253175288	0.341024062
FAM13A1	-0.428734062	6.24E-25	7.24E-23
LAPTM4B	0.006111769	0.888786913	0.920984352
LCN8	0.036917341	0.398132324	0.49617261
TMEM147	0.054994434	0.207946271	0.289683194
SYT4	0.065773439	0.131929539	0.198152109
XPO7	-0.192453254	8.79E-06	3.54E-05
C9orf62	0.07057505	0.10592521	0.164852978
GPR75	-0.093345739	0.03231941	0.058813865
TRIM5	-0.05434758	0.21335261	0.295940717
APOC1	0.076788905	0.078486066	0.12720726
RNASE4	0.140087558	0.001276914	0.003311575
PARD6B	0.441995138	1.45E-26	2.47E-24
ARID1A	0.037556635	0.390007423	0.489120057

TPD52L3	-0.130298327	0.002753318	0.006615924
RRAGB	0.120737726	0.005560289	0.01234304
RCN2	-0.139609513	0.00132727	0.003426273
HIST2H2BE	0.256456009	2.40E-09	1.89E-08
STARD7	-0.194425824	7.08E-06	2.94E-05
SHMT2	-0.076788341	0.078488266	0.12720726
KIAA1751	0.121605913	0.005226529	0.011661076
MLYCD	0.079262275	0.069310639	0.114199121
LOC162632	0.204421506	2.28E-06	1.04E-05
UQCRH	-0.434324041	1.30E-25	1.86E-23
RP11-217H1.1	0.004732815	0.913767221	0.939905427
SDHA	0.023490378	0.590896722	0.677624756
NCLN	0.005295241	0.903566968	0.932063963
ZNF17	0.032193554	0.461253942	0.558536922
RCBTB2	-0.162149532	0.000187905	0.000576692
VEGFB	0.107695685	0.013462951	0.027153753
RP4-747L4.3	-0.298814293	2.61E-12	3.33E-11
COLQ	0.136045506	0.001764258	0.004413516
MPN2	0.099758936	0.02212543	0.042211998
DRG2	0.242203704	1.85E-08	1.24E-07
KLRB1	-0.119333404	0.006140906	0.013490687
ALPK2	-0.093195776	0.032599296	0.059270571
DNASE2B	0.063042423	0.148779721	0.219387651
FLJ23834	0.136790508	0.001663267	0.004195008
AXUD1	0.162920357	0.000174912	0.000540741
SAFB	0.038771965	0.374841404	0.472702993
NSUN4	-0.206751863	1.73E-06	8.09E-06
RFX2	0.235063164	4.90E-08	3.02E-07
MAPK8IP1	0.025002979	0.567213044	0.656587539
FANCD2	-0.155981719	0.000329558	0.000961772
ANKZF1	-0.169477917	9.38E-05	0.000306468
C19orf50	-0.227245125	1.38E-07	7.87E-07
DUSP8	0.168959468	9.87E-05	0.000320672
SENP5	0.027582479	0.527902746	0.620901681
NFKBIL2	0.128947063	0.003049507	0.007251129
LBR	-0.341143584	8.41E-16	2.15E-14
IGFL1	0.016907097	0.699130465	0.772682716
LZTS2	0.023107917	0.596956201	0.683681073
IL2RG	-0.160067845	0.000227657	0.000687894
CCDC51	0.195196622	6.50E-06	2.72E-05
KLF3	-0.004248625	0.922560003	0.946163407
ANKRD37	0.012627972	0.772626424	0.834805813
KCTD14	-0.290910574	1.02E-11	1.18E-10
FZR1	0.163588583	0.000164335	0.000511126
SLC44A4	0.334721815	3.09E-15	7.07E-14
ESPL1	0.072778122	0.095437998	0.151132693
GMPR2	0.175988518	4.94E-05	0.000172033
TBC1D19	0.064346289	0.140541125	0.208841802
ERGIC1	0.337510166	1.76E-15	4.22E-14
ERBB4	0.240419185	2.37E-08	1.54E-07
TSPAN32	-0.197367884	5.10E-06	2.18E-05

MAP4	0.081947517	0.060363461	0.101247932
GPHN	0.201387222	3.23E-06	1.44E-05
SLC6A2	-0.193842099	7.55E-06	3.10E-05
HIVEP1	-0.154607127	0.00037248	0.001074287
DFFB	-0.372981673	8.34E-19	3.66E-17
EIF4EBP2	-0.145198959	0.000838126	0.00225345
DMRT1	-0.304423338	9.66E-13	1.35E-11
HSPB6	0.068759405	0.115236014	0.176925406
IER2	-0.037303455	0.393213037	0.491771087
AIFM1	0.212592255	8.63E-07	4.31E-06
WWC2	0.19364592	7.71E-06	3.16E-05
MRPL4	-0.135650697	0.001820028	0.004541944
FLJ21062	0.142615341	0.00103867	0.002745822
EPB41L4A	0.139789612	0.001308088	0.003382436
SH2D6	0.008157502	0.851936787	0.897167204
TAF4B	-0.366385869	3.73E-18	1.46E-16
GAL3ST3	-0.006337689	0.884704098	0.918771407
MALT1	-0.367715775	2.76E-18	1.11E-16
RTDR1	0.01458879	0.738522077	0.805040372
ARVCF	-0.095575793	0.028393522	0.052493015
MEX3B	-0.275484985	1.29E-10	1.25E-09
FBXO16	0.104205505	0.016814129	0.033148471
KIF7	-0.098355978	0.02407881	0.045473158
C1QC	-0.066139993	0.129784904	0.19540827
ZNF783	-0.103144965	0.017967642	0.035196888
ZNF85	0.001933918	0.964706461	0.974372541
MMP13	0.173242372	6.50E-05	0.000219455
KIAA0329	0.072696886	0.0958094	0.151642728
RTP3	-0.115867693	0.007872485	0.016768192
ZBED3	-0.18758138	1.49E-05	5.74E-05
CLGN	0.165783676	0.000133661	0.000425403
SLC25A37	-0.364621024	5.53E-18	2.10E-16
hCG_18290	-0.207617084	1.57E-06	7.40E-06
OR5AS1	0.00821453	0.850913763	0.896570127
SMARCC2	0.216886228	5.11E-07	2.67E-06
FAM109A	0.146806139	0.000732122	0.001990194
CCDC12	0.159818997	0.000232904	0.000700345
USF2	0.115658261	0.007926338	0.016870561
DEPDC7	-0.301673975	1.58E-12	2.11E-11
C20orf24	0.087693956	0.044396995	0.077732666
JMJD3	0.1000666	0.021715923	0.041546735
DSP	-0.047870006	0.273122456	0.363512983
SLIC1	-0.216063177	5.65E-07	2.91E-06
FAM20A	-0.198202191	4.64E-06	2.00E-05
IRF2BP2	-0.046207127	0.290151181	0.383191498
ZNF230	-0.17622229	4.83E-05	0.000168177
MSN	-0.274289133	1.57E-10	1.48E-09
SLC9A5	0.076702201	0.078824786	0.127618117
EPDR1	0.096832601	0.026367006	0.049324831
MUSK	0.035232725	0.420023994	0.518932598
ZNF434	0.268585547	3.83E-10	3.44E-09

SMARCD1	-0.035352124	0.418449551	0.517403235
ZFP106	-0.196698041	5.49E-06	2.34E-05
ZNF347	-0.107305445	0.013805865	0.027769795
GTF2E1	0.123390896	0.004596405	0.010413888
RY1	-0.12440236	0.004270607	0.009769332
ATAD2B	-0.073822395	0.090765349	0.144903656
ARHGAP17	-0.070940632	0.104124475	0.162664964
KCNIP3	-0.081744252	0.061005553	0.102121658
SFPQ	-0.13926183	0.001365034	0.003511965
GFRA4	0.060350842	0.166942538	0.242069644
AKR1B10	0.028528957	0.513834246	0.608112054
TIGD6	0.234611945	5.21E-08	3.19E-07
RGS16	0.043064042	0.324246913	0.419922971
URB1	-0.109080716	0.012305712	0.025047278
OR4C46	-0.035037118	0.42261083	0.521236408
TOP3B	-0.099767726	0.022113638	0.042211998
NFATC4	0.321430033	4.16E-14	7.60E-13
CA14	0.071278462	0.102482079	0.160428285
BMPR1A	-0.156322057	0.000319668	0.00093602
SNRP70	0.059659833	0.171863093	0.248188388
PRL	-0.046830192	0.283689029	0.376111221
C6orf130	-0.037384813	0.392181197	0.491045038
STAG2	-0.065035554	0.136329682	0.203666476
CD55	-0.014524404	0.739634246	0.805999108
RPS23	0.068518746	0.116516887	0.17844641
SSX2	0.103287712	0.017808462	0.03492958
FDPSL2A	-0.071868898	0.099661024	0.156490203
FBXO27	-0.145533965	0.000814924	0.002194905
SYNGR3	0.268739741	3.74E-10	3.36E-09
TMSL3	-0.024840928	0.569728545	0.658507673
EML1	0.102594803	0.018592826	0.036282859
NUP93	-0.123804972	0.004460408	0.010154405
SMAD3	0.288600684	1.51E-11	1.68E-10
KIAA1189	-0.03001714	0.492113735	0.586890488
HNRPUL2	-0.054499572	0.212073276	0.294564846
TBC1D12	0.047042483	0.281509619	0.373382797
C16orf24	0.49614842	4.90E-34	3.35E-31
MRVI1	0.041445182	0.342784048	0.439303692
ZNF581	-0.010982028	0.801599354	0.858268227
ELOVL3	-0.044206506	0.311564238	0.405977644
OR51Q1	-0.104189004	0.016831569	0.033172217
CACNB3	0.189309338	1.24E-05	4.86E-05
GALNT13	-0.262569988	9.64E-10	8.00E-09
C10orf84	-0.00281061	0.948725579	0.963453937
NEDD4	0.242808031	1.70E-08	1.15E-07
SPO11	0.036914286	0.401799912	0.500236417
OR5AU1	-0.051578262	0.23764112	0.323143575
NEK4	0.14855097	0.000631186	0.001736539
PRKAR2A	0.151117572	0.000505961	0.001416093
IHPK1	0.014384929	0.742045309	0.808153844
ATP6V0B	0.249397949	6.71E-09	4.90E-08

CACNA1E	0.081532601	0.061680122	0.103034792
CEACAM8	0.188288161	1.38E-05	5.37E-05
PEX14	-0.044951018	0.303477228	0.398394849
FLJ12993	0.20659943	1.76E-06	8.22E-06
ZBTB38	0.038125789	0.382859219	0.481727305
PCTK2	0.190863824	1.05E-05	4.16E-05
LRRC16	-0.263107732	8.89E-10	7.41E-09
FBLIM1	-0.242220442	1.84E-08	1.23E-07
FYCO1	0.337316114	1.83E-15	4.37E-14
RP5-1022P6.2	-0.109817791	0.011726426	0.024027255
CMTM1	-0.026279844	0.54758022	0.639155424
PLTP	-0.145478131	0.000818749	0.002204243
RAPH1	0.043870025	0.315265211	0.410018144
DOCK8	-0.144656132	0.000877023	0.002348786
EZH2	-0.146605581	0.000744636	0.002020639
SLC25A1	0.196342858	5.72E-06	2.42E-05
PLEKHB1	-0.121806904	0.005151881	0.011532187
GRB7	0.035968192	0.41038107	0.508500352
ZFP37	-0.344728801	4.01E-16	1.11E-14
MRPL33	-0.136450648	0.001708661	0.004293091
PELO	0.084848148	0.05179367	0.088589506
ARMC1	0.011029206	0.800764892	0.857524089
C9orf27	-0.00817521	0.851619097	0.896986267
FLJ25778	0.066271373	0.129022826	0.194403665
C9orf37	-0.029021729	0.506587423	0.600888516
TMEM66	0.040609994	0.352606541	0.450747641
SPRN	0.163763104	0.000161673	0.000503866
HBEGF	-0.102363504	0.018861281	0.036736781
PI4KA	0.11813535	0.006678666	0.014527822
LEPRE1	-0.057421023	0.188548419	0.267262385
POU2AF1	-0.231242641	8.15E-08	4.84E-07
MRPL12	0.043944401	0.314444675	0.40920959
REP15	0.051868864	0.235004544	0.320053807
ZC3H3	0.266682733	5.14E-10	4.51E-09
RASAL1	-0.315952619	1.17E-13	1.98E-12
DDAH1	-0.058198352	0.18262518	0.260669041
ACBD5	0.257055942	2.20E-09	1.74E-08
TMC2	0.048925024	0.262678972	0.352128406
CCDC137	0.080976612	0.063481535	0.105499449
SAMD13	-0.078345758	0.072602527	0.119017046
UGT2B15	0.208735924	1.37E-06	6.56E-06
TIPARP	0.173157198	6.55E-05	0.000221187
DNASE1L3	-0.092675655	0.033586127	0.060884757
TRIM72	0.060147307	0.168380782	0.243904223
DBX2	0.01060229	0.808324138	0.86381389
IPO8	-0.067633617	0.121324011	0.184704467
C21orf88	0.052404408	0.230199895	0.314625284
MAP3K14	-0.235911362	4.37E-08	2.72E-07
LOC51233	0.258518493	1.77E-09	1.42E-08
GGTLA4	0.005134725	0.906476564	0.934438288
PDE6D	-0.019902056	0.648816686	0.730423618

ZNF117	0.08230677	0.059242278	0.099611914
CLK2	-0.102893792	0.018250751	0.035694615
NKRF	-0.105394497	0.015598454	0.031010311
TNFSF15	-0.253570081	3.67E-09	2.79E-08
DUSP2	-0.20373883	2.46E-06	1.12E-05
SECISBP2	0.256499468	2.39E-09	1.88E-08
GABRR2	-0.01060115	0.808883644	0.864261604
PPAP2C	-0.012061028	0.782572727	0.843625473
LOC51145	0.062738354	0.150752857	0.221818454
PAG1	-0.2331893	6.30E-08	3.80E-07
PIK3C3	-0.253728774	3.59E-09	2.74E-08
GNG10	0.085839118	0.049109136	0.084799797
APOL4	-0.034383595	0.431320656	0.52906258
ANKRD28	-0.058181739	0.182750319	0.260787122
STMN3	0.245738018	1.13E-08	7.85E-08
RAB14	0.105120714	0.015871331	0.031451117
CDK2AP2	0.180768278	3.04E-05	0.000109528
HDDC3	0.198542655	4.47E-06	1.94E-05
COMMD7	0.179953646	3.31E-05	0.000118463
CXXC1	-0.203655945	2.49E-06	1.13E-05
HMCN1	0.050449682	0.248077829	0.334964991
CD40	-0.187843361	1.45E-05	5.60E-05
DYNC1LI2	0.050459358	0.247987009	0.334915905
GDI1	0.438869499	3.57E-26	5.49E-24
LOC646938	-0.184748711	2.01E-05	7.53E-05
VSNL1	-0.312336416	2.29E-13	3.63E-12
PIH1D1	0.170033616	8.89E-05	0.000291956
RAET1G	0.058957332	0.176975601	0.253842541
KRTAP5-9	0.077378551	0.076213752	0.124109736
EFTUD2	0.137538846	0.001567188	0.003973872
ZNF311	0.127316098	0.00344545	0.00808012
ATP6V1G3	-0.061640605	0.158041975	0.230667008
OR2W3	-0.020296196	0.64234157	0.724062019
SCN4A	0.007079527	0.871319635	0.910166567
MED10	0.058869904	0.177619688	0.254588219
FAM135A	-0.379771391	1.72E-19	8.27E-18
ARHGAP4	-0.100959745	0.020564176	0.039577188
EHMT2	-0.038114935	0.382994778	0.481799282
UFD1L	-0.133687691	0.002122066	0.005221522
ERMP1	0.122730351	0.004821115	0.01086695
MAG1	-0.149368857	0.000588461	0.001626999
THAP8	0.159005491	0.000250855	0.000748428
HACE1	-0.251118838	5.24E-09	3.89E-08
FAM82C	0.275020306	1.39E-10	1.34E-09
C3orf20	-0.066765824	0.126185787	0.190877344
UNC84A	0.049771079	0.254505119	0.342515207
SCD5	-0.046385244	0.288293826	0.381311839
LASS6	0.418204944	1.10E-23	1.04E-21
LSG1	0.110864532	0.010945233	0.022607403
MAL	-0.216436503	5.40E-07	2.81E-06
GPR22	-0.025518976	0.560366746	0.651237029

WDR5B	0.010950358	0.802159647	0.858718605
ACTRT1	-0.082470451	0.059466086	0.099933579
C17orf60	-0.095461551	0.028584194	0.052797899
GRIN2C	0.15429689	0.000382862	0.001098051
ARMC8	-0.107868425	0.013313572	0.02689394
SLC47A1	0.138096576	0.001498925	0.003822849
DMPK	0.184257852	2.12E-05	7.87E-05
DHRS13	0.076861007	0.078205292	0.126882411
SMC1A	0.005609472	0.897874818	0.927281198
KRTAP17-1	-0.083478257	0.055705328	0.094439499
SMYD5	0.031863636	0.465861448	0.562454751
TUSC2	0.363376887	7.29E-18	2.68E-16
CRHR2	-0.114160658	0.008777962	0.018541975
KIR3DL2	-0.138295036	0.001490842	0.00380539
CCDC104	0.098253032	0.024227842	0.045684453
ATP2C1	-0.264124184	7.61E-10	6.45E-09
CROT	0.129070511	0.003021292	0.007191096
PABPC3	-0.131806341	0.002453884	0.005978183
EGR1	-0.087481607	0.044916407	0.078485645
THSD1	0.054313044	0.213644072	0.296161186
KHK	-0.072231369	0.097959994	0.154569156
SLC12A2	0.001295199	0.976358692	0.98226924
CD58	-0.284534864	2.97E-11	3.19E-10
STOX2	-0.371868888	1.08E-18	4.63E-17
CCDC76	-0.203781957	2.45E-06	1.11E-05
CCDC48	0.331277497	6.14E-15	1.31E-13
DNAH1	-0.050229395	0.250151707	0.337542867
ZIC4	-0.276320522	1.13E-10	1.10E-09
OR1G1	0.001815345	0.966900743	0.975787407
PSMC6	0.089128801	0.041018151	0.07269538
PROKR1	-0.003394503	0.938093028	0.956288798
ABCB1	-0.05298829	0.225041409	0.309086357
TRAT1	-0.182013158	2.67E-05	9.72E-05
LLGL1	-0.062891009	0.149759788	0.220515551
MTF1	0.006048725	0.889926769	0.921549293
USP54	-0.064890289	0.137209083	0.204781226
PAGE2B	0.121896589	0.005161456	0.011549415
ITGB7	-0.123337766	0.004614123	0.010450181
CCDC81	-0.084278701	0.053390944	0.090916896
LOC149837	0.05934572	0.174135284	0.250389585
SCUBE1	0.141456182	0.00114231	0.002990237
ZSCAN10	0.070781638	0.104904623	0.163658972
HUWE1	-0.022580589	0.605356767	0.690260244
CDH17	-0.020354088	0.643310162	0.724888068
CD180	-0.148575496	0.000629864	0.001733678
IL17A	-0.003203566	0.941568829	0.958719445
TMPO	-0.065589009	0.133018903	0.199544581
KIAA1524	-0.051166105	0.241416163	0.327047365
HDGFRP3	0.11090873	0.010913287	0.022548992
OXCT1	-0.317248383	9.19E-14	1.61E-12
RRAS2	-0.349874865	1.36E-16	4.07E-15

LTBP2	0.14022716	0.001262544	0.003277072
SV2B	-0.157673832	0.000283059	0.000836694
CYP2A6	0.203735467	2.47E-06	1.12E-05
PKD1L2	0.042905059	0.326038012	0.421620975
PPM1M	0.021660189	0.620144171	0.704204341
FLJ22662	-0.18524104	1.91E-05	7.19E-05
ZNF502	-0.211348207	1.00E-06	4.94E-06
GP6	0.052209122	0.231943779	0.316501441
CRYBA2	-0.016295799	0.709241681	0.781563996
LEF1	0.139090466	0.001384009	0.003557806
CTPS	-0.305823294	7.51E-13	1.07E-11
EYA1	0.122851899	0.004779036	0.010780005
EPS8L1	0.292148225	8.26E-12	9.78E-11
MAPK14	-0.0622243	0.154133749	0.225820449
SERPINB2	-0.211891029	9.40E-07	4.66E-06
GTF2F2	-0.178255252	3.93E-05	0.000139011
ZNHIT4	0.001693103	0.969098924	0.97704366
PLA1A	0.141622977	0.001126832	0.002956011
C20orf114	0.320127835	5.33E-14	9.65E-13
HPR	-0.112222211	0.01000086	0.020824682
C18orf2	0.079068945	0.069994662	0.115171842
SATB2	0.322117292	3.65E-14	6.82E-13
KCNJ9	0.056976355	0.191999692	0.271528543
MGC157906	0.011177924	0.798135912	0.855752
MOCS3	0.193362924	7.96E-06	3.24E-05
C17orf71	0.225027253	1.83E-07	1.03E-06
PPHLN1	0.081640308	0.061336075	0.102541739
HIST1H2BN	0.189726906	1.18E-05	4.67E-05
RAPGEF1	-0.116266562	0.007602045	0.016281775
MAP3K8	-0.135990019	0.001772	0.00442928
DLG4	0.104675585	0.016323926	0.032295953
STC1	0.18357676	2.27E-05	8.41E-05
CDGAP	-0.190482082	1.09E-05	4.32E-05
DDX26B	-0.372502157	9.31E-19	4.06E-17
LOC150223	0.07202939	0.09890498	0.155692671
CPSF3	-0.235071563	4.89E-08	3.02E-07
TMEM14A	0.035103376	0.421733553	0.520626303
MYH3	-0.294093552	5.92E-12	7.16E-11
GPKOW	0.177700059	4.16E-05	0.000146387
SULT1A1	0.296808595	3.70E-12	4.56E-11
SPON1	0.016087537	0.712792169	0.784352013
YY1AP1	-0.063607543	0.145164909	0.214726732
RAB23	-0.141540464	0.001134465	0.002974765
PLA2G4A	-0.388195047	2.31E-20	1.29E-18
MAPRE3	0.121572945	0.005238866	0.011675891
ZNF516	0.136720649	0.001672506	0.004214853
GGPS1	-0.019700462	0.652138865	0.732688083
EXOC3L2	0.098852788	0.023370675	0.044299101
C19orf42	0.042281796	0.333121415	0.429156417
MAP2K2	-0.013357053	0.759890396	0.823649929
HIST1H2BB	0.198531587	4.47E-06	1.94E-05

RNF19B	-0.081740571	0.061017234	0.102121658
C6orf128	-0.255820897	2.64E-09	2.06E-08
TLR8	-0.175808713	5.03E-05	0.000175003
PCDHA9	0.005005897	0.908899451	0.936464934
CARS2	-0.235303127	4.74E-08	2.93E-07
CLUL1	-0.207967949	1.50E-06	7.13E-06
RHAG	-0.060649404	0.164849492	0.239603522
UNK	0.032957614	0.450681505	0.548109291
EXOC8	0.022095701	0.613127521	0.697137782
C9orf95	0.007837661	0.857679028	0.901053877
C14orf143	0.341879462	7.23E-16	1.88E-14
MAML3	0.057528494	0.187721176	0.266273936
LDHA	-0.062638491	0.151405195	0.222671739
MRPL20	0.003889994	0.92907881	0.949618617
KLHDC6	-0.304805533	9.02E-13	1.26E-11
ATP5S	-0.024939352	0.568200082	0.657358853
C8orf55	0.229624935	1.01E-07	5.89E-07
PHF19	-0.305756397	7.60E-13	1.08E-11
KRTAP13-4	-0.004073453	0.92574347	0.947470664
TTC5	0.093408715	0.032202488	0.058653169
XKR5	-0.06247493	0.152478265	0.224029542
SILV	0.073112562	0.093921069	0.149076059
TEX28	-0.014663504	0.737232191	0.80418333
TCTN1	0.281653445	4.77E-11	4.92E-10
CX40.1	-0.037492538	0.390817481	0.489781556
PPP2R5C	-0.250644194	5.61E-09	4.14E-08
C12orf30	-0.107148646	0.0139458	0.028032927
CAPG	-0.016408727	0.707319103	0.779864652
MPZL1	-0.29467726	5.35E-12	6.51E-11
ARSB	-0.15110908	0.000506335	0.001416493
TDH	0.07192045	0.099417673	0.156303986
WASF4	-0.051509393	0.238269004	0.323584989
TSSK3	-0.099933775	0.021891901	0.041870388
7A5	-0.017475183	0.68925599	0.764748301
CRISPLD1	-0.191383503	9.88E-06	3.95E-05
MAD1L1	0.089871362	0.039356572	0.070105319
SPIN4	-0.248715489	7.39E-09	5.35E-08
AMPD1	-0.158656146	0.000258955	0.000769605
DPYSL5	-0.08049923	0.065062639	0.107893788
INPP1	-0.226535096	1.51E-07	8.59E-07
ANKRD11	-0.143250275	0.000985649	0.002619169
NPAS4	-0.085494597	0.050028927	0.08599224
GCET2	-0.217797164	4.56E-07	2.41E-06
RNASE9	0.015817035	0.717671463	0.788310437
GUCY2D	0.120626567	0.005604379	0.012422974
CCDC98	0.023958587	0.583517346	0.670788588
FGF4	0.030881409	0.479728596	0.57569304
CPM	-0.12159379	0.005231062	0.011663257
SLC26A4	-3.79E-05	0.999307538	0.999307538
PLD5	-0.07964167	0.067984209	0.112314589
FAM59A	-0.095700504	0.028186627	0.05228343

FBXO5	-0.248431793	7.70E-09	5.54E-08
SIPA1L1	0.049466609	0.257426081	0.346067549
DPYS	-0.264199324	7.53E-10	6.38E-09
ATG4D	0.122640554	0.004852417	0.010928017
TGM3	0.240188047	2.44E-08	1.59E-07
MTCH1	-0.036202343	0.407338797	0.505698822
HK1	0.210671047	1.09E-06	5.34E-06
CDC26	-0.297491217	3.29E-12	4.09E-11
GALNT12	-0.234401292	5.36E-08	3.27E-07
LOC339229	0.247929718	8.27E-09	5.91E-08
MRPL35	-0.00300996	0.945094346	0.961195027
ORC4L	-0.0304177	0.486352457	0.582017414
TNKS	-0.085175742	0.050892984	0.087267417
C2orf24	0.169902811	9.01E-05	0.000295368
ZNF553	0.132684422	0.00229353	0.005616454
GGTLA1	0.112041262	0.010122394	0.02104924
ZNF497	0.245385741	1.19E-08	8.21E-08
CDY1B	0.103271059	0.017826968	0.034954728
SLC30A4	0.018232978	0.676526614	0.753430525
TUB	-0.231980605	7.39E-08	4.41E-07
ARHGEF18	-0.144836324	0.00086393	0.002318771
ARRB1	0.213443954	7.79E-07	3.92E-06
KCNK1	-0.084751548	0.052061786	0.088949131
EREG	0.050467696	0.247908772	0.334883796
SCAMP5	0.161395401	0.000201487	0.00061334
RUNDC3B	0.100386419	0.021297226	0.040809175
ADAMTS20	0.0334029	0.444583659	0.542087035
IL17RB	0.051925002	0.234497608	0.319575752
FLJ20323	-0.08541332	0.050248005	0.086329976
MCAM	-0.135150616	0.001892986	0.004703019
POLR3E	0.18821764	1.39E-05	5.41E-05
AQR	0.067773054	0.120556627	0.183854836
IPMK	-0.010360997	0.812604579	0.866881602
CDCA7	-0.399674879	1.36E-21	8.78E-20
CAMP	0.087598532	0.044629776	0.078029142
GRHL3	-0.132011401	0.002415547	0.005894125
ADAMTSL2	0.169668803	9.21E-05	0.000301499
CLMN	-0.035239665	0.419932384	0.518923679
SSTR3	0.101765971	0.019570503	0.037949866
MAGEA5	-0.015997551	0.714328196	0.785058817
OVOL2	0.202900243	2.72E-06	1.22E-05
JMJD1B	0.183695861	2.24E-05	8.31E-05
RBL2	0.189975924	1.15E-05	4.55E-05
PYGO2	0.106838293	0.014226459	0.028522495
PPP1R10	0.092020466	0.034865388	0.062962488
CSE1L	0.080969328	0.063505418	0.105510623
LCA5	0.005860642	0.893328728	0.923672162
RDH16	0.255912929	2.60E-09	2.03E-08
ASRGL1	-0.274099872	1.61E-10	1.52E-09
TOM1	0.064821167	0.137629059	0.205208799
PTX3	-0.404886946	3.61E-22	2.55E-20

TTC15	0.03375369	0.43981309	0.537656201
SCGB3A1	-0.051186004	0.241232948	0.326871176
MRPL50	-0.178824921	3.71E-05	0.000131868
RCAN3	-0.202542388	2.83E-06	1.27E-05
SLC26A11	0.24193337	1.92E-08	1.27E-07
STYX	-0.122496942	0.004902859	0.011026948
CINP	0.123531032	0.004549966	0.0103277
7-Mar	-0.205590515	1.99E-06	9.19E-06
PFKM	0.01601211	0.714079598	0.784925894
SGMS1	-0.00673574	0.877517998	0.91424232
RIOK3	-0.334978589	2.94E-15	6.74E-14
C1orf110	-0.015253998	0.727063985	0.795926018
CES7	0.002290605	0.958202487	0.969995725
LOC440248	0.014607995	0.738190447	0.804953549
PPP1R12C	0.075486703	0.083699294	0.13469431
C10orf27	0.171781909	7.50E-05	0.000249948
ATG9A	-0.090484343	0.038028217	0.068054571
MRPS26	0.199821359	3.86E-06	1.69E-05
TMEM40	0.004932048	0.910152191	0.937219465
ELP3	-0.046366036	0.288493743	0.3814942
ZNF787	0.069913083	0.10924842	0.169168606
HIAT1	-0.114520245	0.008566361	0.018138621
C8orf34	0.056213867	0.198025131	0.278480872
MGC4655	-0.068306289	0.117656908	0.180013019
PELI1	-0.42568133	1.45E-24	1.56E-22
PPT1	-0.025646142	0.557281921	0.648384858
SLC35C2	0.140245323	0.001260685	0.003273629
C6orf125	0.109757302	0.01177304	0.024095398
MUC4	-0.121734034	0.005178833	0.011575661
RFC4	-0.309272441	4.02E-13	6.00E-12
GNB2	0.201368047	3.24E-06	1.44E-05
NUP50	-0.232077533	7.30E-08	4.37E-07
SULT4A1	-0.194324077	7.16E-06	2.96E-05
C7	-0.005034544	0.908293142	0.93615396
CCDC130	-0.120109249	0.005813735	0.012849984
ARRDC4	-0.045870937	0.293678711	0.387267938
RQCD1	-0.274666253	1.47E-10	1.41E-09
GLYCTK	0.099179749	0.022914535	0.043528415
AYTL2	-0.024087391	0.581494833	0.668838707
MTUS1	0.159596682	0.000237686	0.000711645
LEMD3	0.003758106	0.931477355	0.951437584
PLEKHF2	0.301055501	1.76E-12	2.33E-11
HOXA7	-0.189697248	1.19E-05	4.68E-05
GTF3C2	-0.155974458	0.000329772	0.00096194
DYNLRB2	0.317078397	9.49E-14	1.65E-12
CNOT10	0.006899167	0.87457051	0.912193797
MR1	0.010671687	0.807094121	0.863099435
FFAR1	-0.0048836	0.911031109	0.937404667
PRIC285	0.08509614	0.05111063	0.087591768
SLITRK6	0.076562071	0.079374726	0.128439442
LIX1	-0.043685126	0.317311142	0.412591714

UBE1L2	-0.026329473	0.546823955	0.638725334
F8	-0.055142663	0.206721462	0.288499834
ACHE	0.085811104	0.049183394	0.08490418
KPNA5	-0.362266225	9.33E-18	3.36E-16
TNFRSF12A	0.085499537	0.050015638	0.08599224
EGR3	0.120614291	0.005609268	0.012424852
SERPIND1	0.044365661	0.309823664	0.404453996
OASL	0.10160528	0.01976515	0.038230861
IFRD1	-0.310844238	3.02E-13	4.59E-12
WDFY1	-0.014330028	0.742995057	0.808901665
ZNF267	-0.204704979	2.20E-06	1.01E-05
ACCN5	-0.029683451	0.49735222	0.591760604
ZBTB6	-0.05725826	0.189806367	0.268859561
PPP1R3A	0.074747928	0.087080723	0.139770129
PRRT3	0.242482269	1.78E-08	1.19E-07
FBXL19	0.004127801	0.924755658	0.947089031
TXNIP	0.0125683	0.773671569	0.83549464
ACTN2	-0.069734377	0.110159512	0.170364899
ATG9B	0.074510914	0.087786188	0.140755482
C9orf117	0.249408877	6.70E-09	4.90E-08
IL27	-0.032928104	0.451087292	0.548385876
RPL36AL	0.044889008	0.304145428	0.39881652
KLK15	0.056243315	0.197789894	0.278245266
CHAD	0.211619735	9.71E-07	4.80E-06
RAP2B	-0.20751612	1.58E-06	7.47E-06
HEBP2	-0.204854191	2.16E-06	9.93E-06
ZNF342	0.079553781	0.068289617	0.112788842
CAMK2G	0.029232946	0.503497702	0.597800226
TLR3	-0.043573876	0.318546306	0.413935173
FGF14	-0.134464305	0.001997477	0.004934708
HMGB2	-0.100172212	0.021576876	0.04131928
TRPM5	0.100420828	0.021252599	0.040736356
OR5M11	-0.091102483	0.036727378	0.065956965
KIF3B	0.346050542	3.05E-16	8.67E-15
PRICKLE2	0.122391562	0.004940172	0.011102748
MTMR9	0.114015782	0.008864523	0.018679901
C3orf27	-0.016351436	0.708294239	0.780799799
GLRA3	0.241031334	2.17E-08	1.43E-07
NSDHL	0.152177565	0.000461329	0.00130184
TMEM32	-0.275875232	1.22E-10	1.18E-09
POLR2D	-0.127340716	0.003439142	0.008068404
MYADML	0.045045294	0.302463198	0.397148453
C9orf114	0.107105622	0.013984414	0.028101359
MRGPRX1	-0.006504605	0.882026542	0.916917405
TOPORS	-0.201578864	3.16E-06	1.41E-05
CLDN19	0.022186491	0.611669187	0.695865649
C1QL4	-0.164406214	0.000152209	0.000477761
RNF165	-0.202003303	3.01E-06	1.35E-05
DHRS9	-0.025627555	0.557567742	0.648474379
DNAH2	-0.01949295	0.655565781	0.735463235
FXR1	-0.147860721	0.000669465	0.001835283

ZMYM3	-0.009699888	0.824360665	0.875776387
CASP3	0.076475168	0.079717341	0.128825736
FAM120C	-0.109671572	0.011839387	0.02421836
SCLY	-0.026674466	0.5415811	0.634356722
CA7	-0.030157673	0.490088302	0.584815247
ENTPD5	0.076464995	0.079757525	0.128856811
ZNF461	-0.213143897	8.07E-07	4.06E-06
PDIA5	-0.166676861	0.000122792	0.000394074
C1orf19	-0.212086634	9.18E-07	4.57E-06
TMEM67	0.051629471	0.237175006	0.322652459
KCTD20	-0.072041663	0.098847352	0.155692671
WDR47	0.030174058	0.48985243	0.584647243
FLJ38723	-0.010412683	0.81168722	0.866641797
KLRF1	-0.136943416	0.001643207	0.004151223
TAS2R16	-0.131463882	0.002519149	0.006125048
CLDN12	0.0397642	0.362732876	0.460930865
PRKCE	0.099271705	0.022787647	0.043340935
UBXD4	-0.312079295	2.40E-13	3.77E-12
ITGAM	-0.139380054	0.001352084	0.003484477
GLT8D3	-0.038250289	0.381306309	0.480042997
WDR31	0.119230969	0.006185316	0.013571555
RGS2	-0.237160024	3.69E-08	2.31E-07
OR51L1	0.113534724	0.009157426	0.019211536
MST1R	0.05280732	0.226631361	0.310891147
KIAA1737	0.087013071	0.046080558	0.080246206
OR4A5	0.06105218	0.164891511	0.239603522
GLIS1	0.006716242	0.877869782	0.914409832
PTMA	-0.14588643	0.000791155	0.002135564
NAPA	0.268108588	4.13E-10	3.66E-09
PRDM11	0.032831429	0.452418061	0.549677664
LIPF	0.006722109	0.878927957	0.914626503
DIRAS3	-0.109820573	0.011724286	0.024027255
ASGR2	-0.054398477	0.212923588	0.295423677
C1QTNF4	0.114142949	0.008788502	0.018557865
PIK3R4	0.059853142	0.170475796	0.246489935
ARMC3	0.081049459	0.06324307	0.105219606
NDUFV3	0.123036343	0.004715816	0.010656947
BTBD3	-0.229352274	1.05E-07	6.07E-07
PPP1R2	-0.01340364	0.759078761	0.823217484
UBE2NL	0.026919772	0.537868466	0.63093346
FOSL2	0.052509808	0.229262556	0.313623016
FAM119A	-0.161338439	0.00020255	0.000616269
TUBA4A	-0.221000751	3.06E-07	1.65E-06
KRTAP12-1	0.084290531	0.053357348	0.090884857
SFRS2	-0.02077364	0.634534075	0.71670647
RHPN1	0.397946824	2.09E-21	1.31E-19
EEF2	-0.057060032	0.191346723	0.270854282
ZDHHC11	0.255411088	2.80E-09	2.19E-08
EPHA3	0.122637343	0.00485354	0.010928017
RBM12	0.11067054	0.011086432	0.022852989
H2AFJ	0.430041233	4.34E-25	5.44E-23

EDIL3	0.053865102	0.217450457	0.300939451
KIF26A	-0.0398075	0.36221009	0.460490643
SERGEF	-0.041768721	0.339026285	0.434941087
B3GALT4	0.260440629	1.33E-09	1.08E-08
LOC90925	-0.12826131	0.00321065	0.007590268
OSCAR	-0.093864064	0.031367783	0.057336652
NPFF	-0.010369809	0.812448157	0.866881602
DEDD	-0.072033949	0.09888357	0.155692671
TMEM155	-0.195498954	6.28E-06	2.64E-05
PTPN1	0.317525158	8.72E-14	1.54E-12
SCYL2	-0.039423686	0.366860452	0.465142903
SKAP1	0.196708638	5.49E-06	2.33E-05
LEAP2	0.034460709	0.43028755	0.528216839
GADD45G	0.08552471	0.049947961	0.08599224
IFITM3	0.189359189	1.23E-05	4.84E-05
PILRB	0.004919372	0.910382134	0.937219465
SLU7	0.156084303	0.000326548	0.00095426
DSC3	-0.284242146	3.12E-11	3.33E-10
DNMT3L	-0.10042478	0.021247478	0.040736356
PAPD5	0.300453891	1.95E-12	2.58E-11
B3GNT3	-0.114367768	0.008655526	0.018321111
LHCGR	-0.054863799	0.209901964	0.291944623
MSL-1	0.103950409	0.017085502	0.033608046
UBE2S	0.053686558	0.218981109	0.302655616
SAP130	-0.085492985	0.050033264	0.08599224
ANAPC11	0.246967599	9.48E-09	6.68E-08
MAGED4B	-0.128986244	0.003040526	0.00723257
ATP6V1B2	-0.154161195	0.000387488	0.001110798
C14orf179	0.339958148	1.07E-15	2.68E-14
CAPZA1	-0.122187155	0.00501328	0.011250606
CDYL2	0.202171723	2.95E-06	1.32E-05
GLRX3	-0.14768627	0.000679474	0.001857753
LOC136288	0.057669706	0.186638283	0.265180801
MOBKL1A	-0.07787317	0.07434917	0.121362635
HTR2B	0.063020601	0.148920668	0.219490217
CRYGD	0.024808896	0.570226402	0.658835428
NUS1	-0.29856807	2.72E-12	3.44E-11
PGRMC1	-0.23986524	2.55E-08	1.65E-07
MYOM2	-0.024468091	0.575536144	0.663598678
FLJ39653	0.243002949	1.65E-08	1.12E-07
CHM	0.119738077	0.005968249	0.013158396
OR5M8	0.021419613	0.624035033	0.707186034
ZNF619	-0.098230117	0.024261125	0.045733187
FAM105A	-0.200210286	3.70E-06	1.62E-05
CCNL1	-0.183415992	2.31E-05	8.53E-05
NAP1L3	-0.012327626	0.777891074	0.839461603
C10orf57	0.166671323	0.000122856	0.000394076
B3GALNT1	-0.011144684	0.798723315	0.85591919
CSN1S2A	-0.024821476	0.57003086	0.658733276
TCP10L	0.071186378	0.102927703	0.161084867
GDAP2	-0.198523739	4.48E-06	1.94E-05

DMKN	-0.070284803	0.107372331	0.166767483
COX6B1	0.141852261	0.00110587	0.002906601
DNASE2	0.025173331	0.564574411	0.654518864
MSH5	-0.062191585	0.154350843	0.225976984
LGMN	-0.045518571	0.29740664	0.391763802
USP31	-0.085882945	0.048993148	0.084687206
OR13C8	-0.036991857	0.397180085	0.495186606
SDCBP	-0.088280955	0.042987405	0.075565911
NUDT11	-0.199368862	4.07E-06	1.78E-05
PYGL	0.122163547	0.005021787	0.011265584
SNPH	0.051555981	0.237844126	0.323246738
B3GNT4	-0.080735758	0.064275249	0.106731975
MIZF	-0.268271691	4.02E-10	3.59E-09
NUBPL	0.175486197	5.20E-05	0.000179931
NOD1	-0.216279696	5.50E-07	2.85E-06
CDH22	-0.023125203	0.596681724	0.683494024
NUBP1	0.223111587	2.34E-07	1.29E-06
DSCAM	-0.00934102	0.830758866	0.880444031
DGKI	0.001586889	0.971036572	0.978195263
FAM136A	-0.289704943	1.25E-11	1.43E-10
AKAP1	0.053444828	0.22106573	0.304851575
SLC16A6	0.407439438	1.87E-22	1.42E-20
RIN3	-0.215973328	5.71E-07	2.94E-06
PSG2	0.045725262	0.295216098	0.38904496
DIP2B	0.079362495	0.06895821	0.113648897
PSORS1C1	0.006000875	0.890792067	0.921979535
KIAA0495	-0.166086232	0.000129881	0.00041531
FLJ90709	0.135437405	0.001850825	0.004609445
LPA	-0.100102291	0.021668845	0.04146957
PIGA	-0.140573258	0.001227558	0.003194352
LY75	-0.354718268	4.85E-17	1.58E-15
UTS2	-0.106036095	0.014975048	0.029906323
RREB1	0.175545511	5.17E-05	0.000178968
GALNACT-2	-0.187607152	1.48E-05	5.73E-05
MGC3196	0.037161118	0.395022191	0.493196235
FLJ31568	-0.153266184	0.000419324	0.001193715
LPHN1	0.107190876	0.01390799	0.027966065
SP1	0.236512211	4.03E-08	2.51E-07
TOX4	0.281662562	4.76E-11	4.92E-10
HSPA9	0.365204115	4.86E-18	1.85E-16
APOBEC1	0.131569923	0.002596037	0.00628961
SLC35E4	0.143347215	0.000977777	0.002601624
LSM5	-0.146038061	0.000781129	0.002112208
SURF1	0.321643877	4.00E-14	7.32E-13
ZBTB1	-0.094142287	0.030866935	0.056521972
GTF2F1	0.054773551	0.209781139	0.291842585
RPS15A	-0.162069963	0.000189296	0.00058054
DUSP21	0.079726354	0.067690997	0.111920393
GINS4	-0.05846251	0.180643946	0.258140745
MYO15A	0.130109104	0.002793152	0.006706401
GIMAP7	-0.114582863	0.008529981	0.018074037

MGC13379	-0.218085601	4.40E-07	2.33E-06
ATP6V1E2	-0.268998104	3.60E-10	3.25E-09
UTP3	-0.05732069	0.189323139	0.268251468
HNRPA3	-0.221444268	2.89E-07	1.57E-06
MT4	0.005755704	0.89552656	0.925322268
C14orf155	0.054419493	0.212746617	0.295299988
U1SNRNPBP	0.31699617	9.64E-14	1.67E-12
CKLF	-0.119811294	0.005937481	0.013099953
PLEKHN1	0.092005285	0.034895513	0.062998388
MBNL1	-0.236249903	4.18E-08	2.60E-07
NUP160	-0.254085601	3.40E-09	2.61E-08
ACSM2A	-0.011188188	0.79795455	0.855706754
LOC129881	0.083728463	0.0549731	0.093352552
KIAA1529	-0.187969412	1.43E-05	5.54E-05
FLJ22639	-0.215129298	6.34E-07	3.23E-06
HAND1	0.097399679	0.025494222	0.04780847
GSX1	0.094366564	0.030468196	0.055908367
FGA	0.081469158	0.061883521	0.103346489
SERPINB1	0.158954643	0.000252019	0.000751172
ZNF642	-0.037583543	0.389667669	0.488793655
IGFBP1	0.0023592	0.956951996	0.969726075
SLC1A1	0.01879193	0.667195835	0.745653796
DHX57	-0.26365363	8.18E-10	6.90E-09
ZNF766	-0.010032055	0.818448858	0.871604092
PTPN21	-0.164118904	0.000156371	0.000489326
GDPD3	0.419854288	7.08E-24	7.13E-22
PNPLA5	-0.10774315	0.013421759	0.02709468
TBR1	-0.038596819	0.377004338	0.475040917
FAM116A	-0.064665606	0.138577845	0.206396914
IQGAP1	-0.068842551	0.114796055	0.176382044
FOS	0.031865384	0.465836973	0.562454751
ZNF226	-0.015476154	0.723250754	0.792739552
FIGNL1	-0.284300408	3.09E-11	3.31E-10
C14orf1	0.128014119	0.003270608	0.007714219
ZMYND17	-0.032010963	0.463800799	0.560958126
PUS7	-0.213858863	7.40E-07	3.74E-06
TUBB6	-0.213070419	8.15E-07	4.09E-06
KCNQ2	-0.028233308	0.518207802	0.611604563
6-Mar	0.189422499	1.22E-05	4.81E-05
CCDC33	0.08425921	0.053446331	0.090986016
PRODH	-0.07608197	0.081282518	0.131113905
RBM11	0.156592213	0.000312016	0.000916231
EPHA6	0.095769914	0.028072037	0.052102311
SLC43A1	0.058729083	0.178660781	0.255799188
LOC196541	-0.168976259	9.85E-05	0.000320323
NTN1	-0.017385538	0.690767748	0.76587286
ING4	-0.200758436	3.47E-06	1.53E-05
PCDHB10	-0.153180501	0.000422497	0.001201078
DPH2	-0.309569097	3.81E-13	5.70E-12
SPACA4	0.332417023	4.90E-15	1.08E-13
FBXL21	0.042419941	0.333862797	0.429841361

DIAPH1	0.005521977	0.899459224	0.928449684
ZNF71	-0.244080176	1.42E-08	9.75E-08
CEP76	-0.221804907	2.76E-07	1.50E-06
CORO1A	-0.178195415	3.96E-05	0.000139778
RRM2	-0.05797673	0.184299742	0.262510798
EDG4	-0.054311626	0.213656045	0.296161186
OS9	0.085872734	0.04902015	0.084692643
SLC4A1AP	-0.117562578	0.006950376	0.015043247
COG5	-0.042535864	0.330222049	0.42604603
COPS8	0.088621834	0.042186285	0.074434281
NGLY1	-0.099490045	0.022488797	0.042831985
NCBP2	-0.073622317	0.091646108	0.146030556
C17orf42	-0.091957052	0.034991376	0.063134382
GPSM3	0.042028964	0.336022882	0.432169985
SIL1	0.394385132	5.08E-21	3.12E-19
ASB6	-0.104525814	0.016478732	0.032549864
SMAD5OS	-0.148885308	0.000613385	0.001692105
UNC93A	-0.07737239	0.076237213	0.124115071
A1BG	0.298304349	2.85E-12	3.58E-11
C21orf62	-0.091672559	0.035561383	0.064050072
FMO5	0.112574764	0.009767783	0.020387765
ATRIP	0.371668799	1.13E-18	4.81E-17
CEBPG	-0.212857363	8.36E-07	4.19E-06
C7orf38	-0.019040501	0.663062685	0.74211366
TNFRSF1B	-0.12693539	0.003544346	0.008280466
CLEC1A	-0.119228142	0.006186546	0.013571555
IQSEC1	0.093739534	0.031594201	0.057733355
PATZ1	0.128202347	0.00322486	0.007618005
RBM22	0.162351721	0.000184412	0.000567706
BAG2	-0.306918069	6.17E-13	8.90E-12
PAQR5	0.078259483	0.072918867	0.11947192
C9orf127	0.049528681	0.256828718	0.345339993
THNSL1	-0.191817651	9.42E-06	3.78E-05
SHROOM3	0.102625178	0.018557821	0.036226044
JAM2	0.029414326	0.50085238	0.59523411
SNRPN	0.091208385	0.036508348	0.065582773
ALX4	-0.088925848	0.041482466	0.073381958
CACNA1S	0.014522126	0.7396736	0.805999108
FAM130A1	0.130414023	0.002729217	0.006573426
CORIN	0.083387446	0.055973086	0.094753353
CD300LB	0.260545784	1.31E-09	1.07E-08
PLEKHG6	-0.125231959	0.004019139	0.009266473
LRRC40	-0.401507115	8.54E-22	5.65E-20
PCLKC	0.083451851	0.055783078	0.094519192
PCDHB16	0.100855197	0.020696191	0.039793895
WNT2B	-0.08502828	0.051296783	0.087886297
ASNS	-0.20649924	1.79E-06	8.31E-06
MRPL49	0.17338687	6.40E-05	0.000216581
FLJ46111	0.429019209	5.77E-25	6.95E-23
ISG20	-0.055948574	0.200153578	0.281120226
SMU1	-0.212701082	8.52E-07	4.26E-06

CASZ1	0.062226113	0.154121728	0.225820449
POLR1D	-0.051992446	0.233889595	0.318817805
GIN1	0.096906907	0.026251196	0.04912313
SNAG1	0.026322491	0.546930314	0.638725334
ANKRD29	0.022543664	0.605946971	0.690633535
CDKN2AIP	-0.005871201	0.893137696	0.923629952
KRR1	0.277950834	8.71E-11	8.68E-10
CXCL1	-0.384897257	5.10E-20	2.64E-18
EPM2A	-0.272822	1.97E-10	1.84E-09
PC	-0.108311973	0.012936679	0.026218734
DEFB127	-0.052204504	0.23741123	0.322902378
PDZRN4	0.021746813	0.618745759	0.702746153
FAH	0.207738408	1.54E-06	7.31E-06
OR51E1	0.069179135	0.113028509	0.174057677
CDC2L6	-0.100938532	0.020590902	0.039609672
DNTTIP1	0.13982369	0.001304487	0.003374544
PAX8	0.056932394	0.192343391	0.27195206
TMEM116	0.128561627	0.00313915	0.007438395
C1orf150	0.010463135	0.810792009	0.865849264
PRO2012	-0.118053666	0.006716824	0.014604578
MRPL40	0.059201942	0.175182765	0.251564415
BEX1	0.120593639	0.0056175	0.012438606
SLC2A4	-0.001417673	0.974123917	0.980662732
PKMYT1	0.110350204	0.011323124	0.023271431
FEZF2	-0.030381608	0.486870089	0.582444392
SLC26A9	-0.31736114	9.00E-14	1.58E-12
MAP2	-0.3091783	4.09E-13	6.08E-12
LYL1	-0.034064004	0.435617501	0.533481779
SLC25A19	-0.044104968	0.312678039	0.40717011
NOS3	0.062195039	0.154327909	0.225976984
ZNF34	0.227891023	1.26E-07	7.26E-07
TMPRSS11F	0.05645195	0.196129088	0.276414798
FAM43A	0.118591234	0.006469252	0.014126218
FCRL4	-0.18561952	1.83E-05	6.93E-05
KLF14	-0.017091837	0.695729243	0.769569907
FLRT2	-0.043271214	0.321922518	0.417528277
WRN	-0.21518464	6.30E-07	3.21E-06
SDF2	0.14751644	0.000689352	0.001882248
KRT8P12	0.388198456	2.31E-20	1.29E-18
C6orf195	-0.140876129	0.001223951	0.003186315
C9orf125	-0.045345739	0.299246635	0.39361508
DZIP3	0.266025427	5.69E-10	4.96E-09
RIT1	-0.071999385	0.09904598	0.155842817
SCML1	-0.007121372	0.870565706	0.909773713
RHBDF2	-0.042971027	0.325294035	0.421013054
OR2G3	0.066697603	0.126574316	0.191323861
REXO1L1	-0.019398095	0.657134657	0.736685689
MAP3K7IP3	0.024337526	0.577576482	0.665576797
C3orf57	0.131262363	0.00255829	0.006207941
FBXW11	0.173440718	6.37E-05	0.00021557
ETAA1	-0.085249448	0.05069215	0.086971549

C14orf131	0.081836376	0.060713847	0.101724645
AKT1S1	0.156117373	0.000325583	0.000952025
SLC12A5	0.139969049	0.001289231	0.003337946
C9orf164	0.021009607	0.630690238	0.712888653
NRIP3	0.05543702	0.204304709	0.285905702
NOS1AP	0.201257482	3.28E-06	1.45E-05
TMEM121	0.20681303	1.72E-06	8.04E-06
SAP30BP	0.033264766	0.446470261	0.543848185
DGCR6	0.081880511	0.060574504	0.10151884
WDR76	-0.073772318	0.090985152	0.14520314
FAM82B	0.14217246	0.001077198	0.002839131
LOC606495	0.003714761	0.932265755	0.952084725
MAP9	0.092738638	0.033465288	0.060683591
BCDIN3D	0.212671673	8.55E-07	4.27E-06
CXorf36	-0.033182216	0.447599865	0.545008232
DSCR3	0.010625591	0.807911089	0.863577543
ZFAND3	0.18190537	2.70E-05	9.81E-05
C7orf43	0.236399994	4.09E-08	2.55E-07
SPSB3	0.19591192	6.00E-06	2.53E-05
C19orf19	0.15771773	0.000281938	0.000833881
FAM133A	-0.021637346	0.620513157	0.704233186
C12orf25	0.067327173	0.123023885	0.186829802
SLC39A3	0.170671178	8.36E-05	0.000275441
DISP2	0.082120085	0.059822729	0.100478001
PI4KAP2	0.014218472	0.74492611	0.810860445
MKRN3	-0.086971339	0.046185465	0.080406123
ADAMTS13	0.096345088	0.027137792	0.05058208
CBLN3	0.142188731	0.00107576	0.002836556
TTYH1	-0.2998668	2.17E-12	2.81E-11
C3orf18	0.241936153	1.92E-08	1.27E-07
FLJ13236	0.123429423	0.004583595	0.010392524
ZMYND12	0.05832734	0.181655753	0.259405765
C18orf25	-0.140644708	0.001220447	0.003179886
GLB1L3	-0.024904502	0.568741057	0.65779554
ATP13A5	-0.18404067	2.41E-05	8.87E-05
RANBP10	0.036429765	0.404396814	0.502756977
CD96	-0.198829889	4.32E-06	1.88E-05
DENND1C	-0.218837196	4.01E-07	2.13E-06
RBMS3	0.039545575	0.365379619	0.463623457
SLC41A3	-0.150116082	0.000551789	0.001534117
DGCR6L	0.04710902	0.280828871	0.372721072
TMEM128	0.201810268	3.08E-06	1.37E-05
CSNK1G3	0.184723089	2.01E-05	7.54E-05
MOBKL2C	-0.14741641	0.000695231	0.001894097
TSPAN6	-0.28517814	2.67E-11	2.89E-10
MATN2	-0.070352197	0.107034935	0.166285451
MSL2L1	-0.135195633	0.00188631	0.004690223
ST6GALNAC2	0.275210222	1.35E-10	1.30E-09
FGFBP2	-0.251481789	4.97E-09	3.71E-08
FGL1	0.027811597	0.52447924	0.617347406
MPP3	0.126906371	0.003551989	0.00829517

ARHGEF6	-0.149296764	0.000592116	0.001636369
TGFBR2	-0.134533671	0.001986681	0.004911984
ACMSD	0.150123998	0.000551412	0.001534117
IL33	-0.083162282	0.056641592	0.095815447
C9orf5	-0.019833956	0.64993817	0.731418339
DEAF1	0.073057846	0.094167915	0.149352208
AMN	0.058007748	0.184064695	0.262297523
DEFA6	-0.03503404	0.422651607	0.521236408
RNF212	0.003467629	0.936762158	0.955543689
METT5D1	0.182353705	2.58E-05	9.41E-05
CIB1	0.371867251	1.08E-18	4.63E-17
TSSK1B	0.044829876	0.30478352	0.399172282
KIAA1727	0.044879085	0.304252447	0.39881652
ZNF680	0.135764789	0.001803748	0.004506804
LOC399900	-0.030175985	0.489824696	0.584647243
LOC152217	-0.104922279	0.016071717	0.031817446
CTNNAL1	-0.262730254	9.41E-10	7.83E-09
CIT	0.018250786	0.676228551	0.753420794
TLE6	0.126327665	0.003707561	0.008619202
ZNF607	0.05077816	0.245007723	0.331369609
HERC4	-0.130543616	0.00270245	0.006519171
DRAP1	0.16652557	0.000124572	0.000399163
PEMT	0.051014812	0.242812401	0.32864923
C10orf111	-0.153780475	0.000405911	0.001159288
ZNF575	0.009676219	0.824782307	0.876072966
KCTD7	-0.171118555	8.00E-05	0.000265248
MYO1F	-0.136447123	0.001709138	0.004293091
LOC285382	0.012021233	0.783272235	0.844083592
RAB11A	0.323586555	2.75E-14	5.26E-13
PLCD3	0.153986587	0.000393516	0.001126504
C15orf28	0.029760556	0.495823288	0.590169841
PTBP2	-0.313743776	1.77E-13	2.86E-12
CTB-1048E9.5	-0.110532112	0.011188172	0.023024119
C19orf60	0.052235901	0.231704091	0.316329586
C7orf25	-0.009854159	0.821613755	0.873763919
SETD7	0.065828857	0.131603553	0.197759102
HOXB9	-0.055519442	0.203631683	0.28522895
VANGL1	0.147477231	0.000691651	0.001887688
CHAF1B	-0.174710533	5.62E-05	0.000192786
NDUFA3	0.233960315	5.68E-08	3.45E-07
KIAA1328	-0.151623806	0.000484167	0.001362537
SHARPIN	0.333173512	4.21E-15	9.32E-14
TTC23	-0.184739121	2.01E-05	7.53E-05
UGP2	-0.343607345	5.06E-16	1.35E-14
ANKIB1	0.11207616	0.010098853	0.021021614
CIRBP	0.145357001	0.000827105	0.002225764
SEC14L4	-0.265965643	5.74E-10	5.00E-09
OVCH1	-0.064494213	0.139628998	0.207636931
VPS52	0.174589207	5.68E-05	0.000194803
FAT	-0.373426862	7.53E-19	3.33E-17
M6PRBP1	0.132665534	0.002296876	0.005621853

GPRIN3	-0.086742649	0.046763922	0.08131081
PPM1F	-0.207507867	1.59E-06	7.48E-06
TSR1	-0.158034574	0.000273973	0.000811885
CCDC85A	0.114228172	0.008737882	0.018482708
PCSK5	0.071915556	0.099440753	0.156303986
ZFHX3	0.327272847	1.35E-14	2.69E-13
HEMK1	0.209766411	1.21E-06	5.86E-06
PGBD2	-0.089382619	0.040443653	0.071792155
RSRC2	-0.061360253	0.159945523	0.233058062
AURKC	0.118803288	0.006373865	0.013932776
SCRIB	0.185795448	1.80E-05	6.81E-05
ORM2	-0.132661111	0.00229766	0.005621853
FAM115A	0.154405378	0.000379201	0.001091144
FZD6	-0.078918998	0.070528988	0.115895977
UNC119	0.121564524	0.005242021	0.01167869
GPX3	-0.063299722	0.14712549	0.217052457
NOV	-0.272205341	2.18E-10	2.01E-09
CABC1	-0.09582913	0.027974595	0.051937133
CDC42SE2	-0.147959764	0.000663843	0.001820683
EIF2S2	-0.039983185	0.360093804	0.458612369
RNF130	-0.224090079	2.06E-07	1.16E-06
CKAP5	-0.014142938	0.746234514	0.81214089
RP11-413M3.2	0.082574851	0.058416909	0.098493439
C10orf18	-0.193265733	8.04E-06	3.28E-05
TMEM93	0.146435914	0.000755377	0.002047075
DYX1C1	0.103973541	0.017060737	0.033570071
KCNMB2	-0.056297981	0.197353744	0.277758794
ANK3	-0.145948189	0.000787057	0.002125435
KRT5	-0.244835957	1.28E-08	8.84E-08
CDH12	-0.16704484	0.00011856	0.000381689
QRSL1	-0.171859271	7.44E-05	0.000248449
JUB	-0.072072222	0.098703982	0.155583385
SHC4	-0.308707675	4.46E-13	6.59E-12
CCL15	0.033342676	0.44540562	0.542766381
CCDC22	0.253731141	3.59E-09	2.74E-08
SNX24	0.23566442	4.52E-08	2.80E-07
RARS	0.140947585	0.001190725	0.003107711
MORC2	-0.127781071	0.003328065	0.00782872
FAM48A	-0.046320519	0.288967831	0.381874746
MT1H	-0.049162807	0.26036367	0.349405545
PPP1R14C	-0.368494491	2.32E-18	9.44E-17
FOXD1	-0.013474107	0.757851561	0.822600044
C1orf213	-0.298984211	2.53E-12	3.24E-11
AMT	0.037399249	0.391998283	0.490915976
DSN1	0.026427441	0.545332588	0.637257712
PTPLAD2	-0.044486006	0.308511798	0.40311072
DIS3L	0.251289067	5.11E-09	3.80E-08
RASL11A	-0.004612027	0.91595977	0.940691584
GPRC5B	-0.281302257	5.05E-11	5.18E-10
FRMD7	0.109353241	0.012170974	0.024789441
STRN4	0.178059792	4.01E-05	0.000141552

KITLG	0.187491888	1.50E-05	5.78E-05
HDGF	-0.099103559	0.023020133	0.043702006
OR1S1	0.065152208	0.135626633	0.202813269
SETX	-0.044872211	0.304326595	0.398828695
DDR2	-0.124446369	0.004256918	0.009745268
KCTD12	-0.154939368	0.000361653	0.00104502
LYZL2	0.200310017	3.65E-06	1.60E-05
WDR52	0.117746175	0.006862223	0.014873391
TMEM2	0.028007804	0.521556547	0.61461311
ZNF579	0.080998375	0.063410217	0.105437919
LOC200810	0.40181058	7.91E-22	5.29E-20
TNFSF9	-0.018748815	0.667913772	0.746049371
PPFIA4	-0.054178568	0.214781714	0.297654442
CNIH3	0.207964152	1.50E-06	7.13E-06
MAP4K4	-0.261273228	1.17E-09	9.64E-09
ROD1	0.033777422	0.439491414	0.537369796
ALS2CR12	0.178602909	3.79E-05	0.000134416
DOCK3	-0.092138252	0.034632398	0.062561938
PAQR9	-0.019011412	0.663545841	0.742384166
ASB17	0.013592523	0.755790723	0.820507973
STX16	0.188950017	1.29E-05	5.04E-05
FEZ2	-0.159455599	0.000240769	0.000720432
DLAT	-0.205422651	2.03E-06	9.36E-06
KIF21B	-0.144825091	0.000864741	0.002319935
CDC5L	-0.200637261	3.52E-06	1.55E-05
TMEM119	-0.015245544	0.727209224	0.79594331
CRIP3	-0.205976872	1.90E-06	8.81E-06
TPSD1	0.087696389	0.044391072	0.077732666
TEPP	0.014019904	0.748367292	0.814173829
GNGT2	-0.093688705	0.031687017	0.057868569
C21orf121	-0.068153103	0.118484285	0.181143678
WNK1	0.018455612	0.672804066	0.750421223
FLJ10490	0.118251012	0.006624968	0.014420152
OR51B5	0.054998734	0.207910669	0.289683194
LOC203547	-0.188353601	1.37E-05	5.35E-05
HAS1	-0.090341963	0.038333322	0.068535123
PPA1	-0.228260155	1.21E-07	6.94E-07
ST7	0.057988246	0.184212447	0.262447251
C11orf46	-0.166232933	0.000128085	0.00040978
POPDC3	-0.117095789	0.007179088	0.015472911
ACOX2	0.288859011	1.44E-11	1.63E-10
ATCAY	0.066934371	0.125229855	0.189851671
TM4SF19	0.029140236	0.504852652	0.599177564
MFSD9	-0.003058089	0.944217836	0.960462444
PDHB	0.16567635	0.000135026	0.000429082
ERN1	0.080829637	0.063964911	0.106245337
LCE3C	0.031932059	0.464903791	0.561791956
GPR111	-0.010193659	0.815922678	0.869365543
NOTCH3	0.032000608	0.463945464	0.561022745
ADAMTS5	-0.162403531	0.000183527	0.000565667
B3GALT1	-0.046414892	0.287985445	0.380985908

UGCGL1	-0.002898456	0.947125312	0.962782864
FAM58A	-0.210476392	1.11E-06	5.44E-06
FBXO32	-0.186216386	1.72E-05	6.53E-05
CLPP	0.04429534	0.310591924	0.405225915
NXPH1	-0.021880939	0.616583233	0.700678304
MTMR3	-0.097949539	0.024671855	0.046450471
ATP1B3	-0.103255555	0.017844213	0.034966242
TMEM16A	0.206450393	1.80E-06	8.35E-06
HIST1H3F	0.197852758	4.83E-06	2.08E-05
TRIM25	0.057820924	0.185483772	0.26389165
SDCBP2	0.012170652	0.780646698	0.842134437
CRKL	-0.060120664	0.168569731	0.244120413
HOXB2	0.265893645	5.81E-10	5.04E-09
ANP32B	-0.235402113	4.68E-08	2.89E-07
GATM	0.055808316	0.201285573	0.282323219
AP4E1	0.190140352	1.13E-05	4.47E-05
EDG5	-0.211704758	9.61E-07	4.75E-06
CDKN3	-0.046329738	0.288871768	0.381829859
CDH4	-0.095654414	0.02826294	0.052363397
PGD	-0.111249898	0.010669461	0.022119527
RND1	0.30020948	2.04E-12	2.68E-11
GAD1	0.018929174	0.664912532	0.743642627
MPG	0.321939119	3.78E-14	7.02E-13
LOC440350	0.090859529	0.03723408	0.06671106
ZNF133	-0.273722593	1.71E-10	1.61E-09
SERPINB12	0.023881035	0.586175002	0.672837425
AMELY	-0.0228252	0.601453434	0.687167812
DHX36	-0.257052666	2.20E-09	1.74E-08
TNFAIP8L2	-0.137748722	0.001541172	0.003915814
PHTF2	0.178784018	3.73E-05	0.000132192
CCDC112	0.053199886	0.223192475	0.307164398
IQCC	-0.002300878	0.958015205	0.969995725
HEYL	0.14433414	0.000900877	0.002407428
FTSJ2	0.078280277	0.072842523	0.119378645
APPL1	-0.073489507	0.092234521	0.146739992
RAB43	-0.094389768	0.030427195	0.055849797
OR10G2	-0.100632253	0.020980171	0.040289528
WAC	-0.106853718	0.014212393	0.02850359
ADCY9	0.410269351	8.98E-23	7.27E-21
RUNDC2B	0.052871522	0.226066384	0.310216959
PYCRL	0.344694946	4.04E-16	1.11E-14
AGPAT7	0.095591425	0.028367519	0.052460714
SLC22A9	0.015540071	0.722154919	0.792244531
CDKAL1	-0.275818954	1.23E-10	1.19E-09
PDYN	0.011677108	0.789328401	0.848824824
C20orf74	0.003128291	0.942939446	0.959322806
MTMR11	-0.048974586	0.262195216	0.351633236
VAV3	0.288791035	1.46E-11	1.64E-10
DAPL1	-0.100624549	0.020990043	0.040295903
STXBP3	-0.05933987	0.174177815	0.250389585
EIF3G	-0.144122013	0.000916918	0.002447104

ARHGAP22	-0.25322334	3.86E-09	2.93E-08
NPFFR1	0.051888749	0.234824886	0.319879979
NPC1	-0.164245826	0.00015452	0.000484025
ALDH9A1	-0.099876186	0.021968584	0.041996535
ZNF600	0.052517753	0.229192012	0.313596279
ZNF678	0.05787248	0.18509136	0.263455271
RASSF1	-0.00942585	0.829245422	0.879446379
ADD2	-0.342347494	6.57E-16	1.73E-14
PITPNB	-0.281323143	5.04E-11	5.17E-10
PKD2L2	0.015730751	0.718889119	0.789380421
LRP11	0.17987044	3.33E-05	0.000119407
CDKL1	-0.283681298	3.42E-11	3.62E-10
SMEK2	-0.221866992	2.74E-07	1.49E-06
PRODH2	-0.007358821	0.86628981	0.907000858
C11orf54	-0.163240849	0.000169762	0.000526142
SFRS11	-0.254270332	3.31E-09	2.54E-08
IL7	-0.255365005	2.82E-09	2.20E-08
ALS2CR16	0.09012514	0.038801933	0.069217606
BTG3	-0.423588979	2.57E-24	2.68E-22
PAK2	0.036389048	0.404922606	0.503207176
RP11-679B17.1	0.024758	0.571017876	0.6593782
GATA4	0.134523091	0.001988325	0.004914071
ATP2B1	0.051508025	0.238281482	0.323584989
LOC130940	0.103022073	0.018105677	0.035455989
C1orf172	-0.122577652	0.004874452	0.010971086
ATF7IP2	0.030484625	0.485393441	0.581130114
SLC25A43	0.059796727	0.170879794	0.247000436
CENTG3	0.045140607	0.301440298	0.396059058
IGF2BP1	0.014689197	0.736788798	0.80418333
FCHSD1	-0.014686116	0.736841958	0.80418333
CAMK2N2	0.097689968	0.025057168	0.047075016
ELAVL3	-0.046465788	0.287456573	0.380368079
NBPF15	0.037356344	0.392542076	0.491196831
UBE2J2	-0.036490484	0.403613492	0.502189268
GNL2	-0.257123805	2.18E-09	1.73E-08
PRR3	-0.069797366	0.109837693	0.169952686
NLF2	0.05891369	0.177296897	0.254184803
OR4F6	-0.010216688	0.815167242	0.868711156
KLHL24	-0.309816298	3.64E-13	5.48E-12
CCDC88A	-0.301405913	1.65E-12	2.20E-11
SGPP1	-0.024298605	0.578185373	0.665904075
C10orf11	-0.124473804	0.004248404	0.009733024
SLC35B4	-0.230265802	9.27E-08	5.44E-07
UGT3A2	-0.029809583	0.495113336	0.589553041
ARNT2	-0.070384817	0.106871928	0.16615815
CBR1	-0.131060033	0.002598145	0.006292239
ITPR3	-0.01725085	0.693041402	0.767010724
TRAPPC6B	0.087820439	0.04409002	0.077261195
AMZ1	0.429951119	4.45E-25	5.47E-23
ARP11	-0.018368366	0.674261908	0.751501989
WDSUB1	-0.025377953	0.561412769	0.652143074

APBA1	-0.24306998	1.64E-08	1.11E-07
RAB2A	0.099520445	0.022447459	0.042773295
C6orf162	-0.391855909	9.46E-21	5.70E-19
HPSE2	-0.076532284	0.079492025	0.128562983
PLCE1	-0.188071551	1.41E-05	5.48E-05
INSL3	-0.119079028	0.00625172	0.013690109
DLG1	-0.118782627	0.006383103	0.013948011
PTPLA	-0.353359253	6.49E-17	2.07E-15
PIGX	0.165762836	0.000133925	0.000426023
TFIP11	-0.034977254	0.423404362	0.521850756
FIBIN	0.000637191	0.988368046	0.991916944
POLR2G	0.019810843	0.650318978	0.731579107
GRAP2	-0.240548354	2.32E-08	1.52E-07
DNAJB8	0.005577981	0.898445024	0.927714266
CNBP	-0.138821502	0.00141428	0.003629553
WASF1	-0.165724901	0.000134407	0.000427335
INPP5E	0.026432732	0.545252103	0.637257712
HSPB1	1	1.00E-53	1.00E-49
TMEM167	0.091032022	0.036873724	0.066136959
CUBN	-0.166026786	0.000130616	0.000417225
IGF1	0.005270288	0.904019205	0.932217691
ITPK1	0.329037511	9.55E-15	1.95E-13
NAALAD2	-0.098202387	0.024301453	0.045781138
G3BP1	-0.006477815	0.882173259	0.916917405
NT5DC1	0.008104658	0.852884995	0.897858215
CYP39A1	-0.438900175	3.54E-26	5.49E-24
TMEM139	-0.080161218	0.066201619	0.109634731
POLK	0.085226642	0.050754222	0.087053754
GLULD1	-0.060455908	0.166203726	0.241205266
RBM15	-0.089693815	0.039748583	0.070721654
AMZ2	0.22340577	2.25E-07	1.25E-06
GDF15	0.474753771	6.34E-31	2.29E-28
MESDC2	0.018764058	0.667659917	0.746036858
INCA	-0.195273906	6.44E-06	2.70E-05
ACY1L2	-0.436647519	6.74E-26	1.01E-23
GZMM	-0.12961288	0.002900122	0.006936152
PAIP1	-0.007321663	0.866958681	0.907546634
CACNA2D1	0.088451113	0.042585918	0.075053256
STK32C	0.013612551	0.755442334	0.820419447
SH3BP4	0.080013943	0.066702981	0.110375843
1-Dec	-0.025082675	0.565977881	0.655651467
PADI1	-0.009872471	0.821287829	0.873748492
UBB	0.228393621	1.18E-07	6.85E-07
PON3	-0.146445511	0.000754766	0.002046835
PROP1	0.053249702	0.222758761	0.306704796
ANKRD13B	-0.00921371	0.833031365	0.882092279
ADCK1	0.184835134	1.99E-05	7.48E-05
TCF25	0.106865064	0.014202055	0.028492148
SLC38A5	-0.13519707	0.001886098	0.004690223
CXorf26	-0.007994549	0.854861394	0.898927555
C19orf39	0.220547828	3.24E-07	1.74E-06

PPP1R13B	0.127544514	0.003387324	0.007952903
ARL2	-0.0119374	0.784746388	0.845375883
TCL6	0.042608247	0.329399029	0.4251626
TOP3A	0.033583815	0.442119634	0.539831936
SLC16A14	0.170781979	8.27E-05	0.000272777
FXYD6	-0.282723416	4.00E-11	4.19E-10
HIST1H4E	-0.080987641	0.063445382	0.10546787
BBC3	0.209984356	1.18E-06	5.72E-06
UNC5A	-0.019424347	0.656700297	0.736601629
FAM86C	0.158251596	0.000268639	0.000796847
PI4KB	-0.039702892	0.363473872	0.461681644
B3GAT1	-0.29033023	1.12E-11	1.30E-10
SUSD2	0.041901168	0.337495604	0.433791905
OAZ2	0.029498979	0.49962029	0.593999452
NOC4L	0.312254292	2.33E-13	3.68E-12
C10orf12	-0.125724363	0.003876297	0.0089674
FADS1	-0.007443703	0.864762224	0.906417106
LOC144097	0.014724695	0.736176335	0.804118215
DKK2	0.102581917	0.018607694	0.03629538
KIAA1949	-0.223432477	2.25E-07	1.25E-06
RHOT1	0.068673013	0.115694544	0.177429296
OXT	0.004642997	0.915397538	0.940481113
GPR153	0.073244645	0.093327312	0.148325056
ARL4A	-0.16748414	0.000113688	0.000366964
SAAL1	-0.342492441	6.37E-16	1.69E-14
CCDC64	0.238415009	3.11E-08	1.98E-07
USE1	-0.094551331	0.030143024	0.055444048
HNMT	0.058632807	0.179375152	0.256625829
PCGF3	0.282655392	4.05E-11	4.23E-10
CYP2C19	0.028997837	0.506937534	0.601071904
C20orf4	0.350467113	1.20E-16	3.64E-15
CCDC11	0.197349229	5.11E-06	2.18E-05
ACSBG2	0.10883109	0.012507553	0.025424444
RWDD2A	-0.019050543	0.662895923	0.742062084
PALLD	0.024400465	0.576592511	0.66456745
CPLX4	0.082242562	0.060919189	0.102040887
LOC492311	0.152073574	0.000465539	0.00131312
KPNA2	0.001710952	0.96877332	0.976875557
MACROD1	0.210256636	1.14E-06	5.58E-06
TMCO3	-0.006678227	0.878555694	0.914626503
C15orf52	-0.237219511	3.66E-08	2.30E-07
BIRC5	0.002705032	0.950649163	0.964619462
PRR16	-0.240952202	2.20E-08	1.44E-07
FAM63B	0.249026841	7.07E-09	5.14E-08
KATNB1	0.083513992	0.055600258	0.094287365
WNT8B	-0.051371765	0.239527251	0.324846288
CPLX3	-0.046606002	0.286002947	0.378770648
GHR	0.163868207	0.00016009	0.000499691
CCDC124	0.039070118	0.371177038	0.469526354
BCLAF1	-0.171926043	7.39E-05	0.000246962
GOLGA3	0.112729936	0.009666736	0.020197336

CLEC4E	-0.276980206	1.02E-10	1.00E-09
AKR1CL1	-0.003144352	0.942646986	0.959322806
BBS7	-0.222036951	2.68E-07	1.47E-06
MGAT4B	0.013445947	0.758341904	0.822696607
KIAA2018	-0.036659273	0.401440749	0.499890475
SERPINB9	-0.212236095	9.01E-07	4.49E-06
OR6M1	-0.081401562	0.062100845	0.103653121
PLEC1	0.27093736	2.66E-10	2.42E-09
RP13-36C9.6	-0.006852723	0.875407977	0.912353161
PIP3-E	-0.214519618	6.83E-07	3.48E-06
KNTC1	0.006869429	0.875106718	0.912193797
CCDC57	0.371531085	1.16E-18	4.93E-17
LAIR1	-0.112059345	0.01011019	0.021038091
C21orf96	-0.135557306	0.001833454	0.004569886
GTF3C3	-0.087234286	0.045527796	0.079373523
LRRC8D	-0.351664657	9.32E-17	2.89E-15
METTL2B	0.198194073	4.64E-06	2.00E-05
DNAJC5	0.131105765	0.002589088	0.006275246
FLJ20035	0.105188292	0.015803591	0.031347186
C21orf56	0.131021228	0.002605854	0.006308423
C14orf145	-0.257659992	2.01E-09	1.60E-08
RASGRF1	-0.175203101	5.35E-05	0.000184265
C4orf15	0.036291275	0.406186835	0.504370527
ALDH2	-0.050595413	0.246712459	0.333341004
RIBC1	0.175301172	5.29E-05	0.000182981
EMP2	0.377997542	2.61E-19	1.22E-17
C3	-0.22976224	9.91E-08	5.79E-07
MRAP	0.004918726	0.910393864	0.937219465
TRIM41	0.161581361	0.000198055	0.000604471
POLE3	-0.178843919	3.70E-05	0.000131765
MGC26356	0.037489691	0.390853487	0.489781556
APOC4	0.013446901	0.758325283	0.822696607
CTSL2	-0.207280735	1.63E-06	7.63E-06
TRIM2	-0.398635623	1.76E-21	1.13E-19
CP110	0.014484675	0.740320764	0.80656137
KRTAP19-1	-0.00610375	0.88893188	0.920984352
MRGPRD	0.022243676	0.61075143	0.695464916
KIAA1622	0.033827472	0.43881343	0.536708393
DNM1	0.076937118	0.077909788	0.126502857
HYOU1	-0.136690495	0.001676509	0.00422148
UGT2B10	-0.04005625	0.359215941	0.457691426
KRT26	0.033034003	0.450933786	0.548307662
ZNF25	-0.026145092	0.549636233	0.640946936
USP7	0.20211851	2.97E-06	1.33E-05
HNRNPR	-0.164247582	0.000154494	0.000484025
SERPING1	-0.059358063	0.174045578	0.250389585
AADACL4	0.010229668	0.815110299	0.868711156
TPCN1	0.203427208	2.55E-06	1.15E-05
STARD13	0.024011835	0.582680837	0.670077514
KLRG2	-0.086286638	0.047935529	0.083099962
SLC7A3	-0.044244164	0.311151819	0.405784207

ADI1	-0.33466174	3.13E-15	7.10E-14
WBSCR22	0.110978455	0.010863058	0.022467858
LRRC4C	0.017301541	0.692185361	0.766615235
SLC36A3	-0.067798453	0.120417256	0.183733426
SLC35D2	-0.014709372	0.736440677	0.80418333
UNQ2541	0.057604407	0.187138457	0.265604264
RACGAP1	0.020243639	0.643203447	0.724888068
OBP2A	-0.304235167	9.99E-13	1.38E-11
PSMD3	0.086939622	0.04626533	0.080501466
RAB35	0.040087635	0.358839277	0.457495897
ERLIN2	0.104099653	0.016926274	0.033337495
C2orf13	-0.339793016	1.11E-15	2.76E-14
C1orf168	0.182947265	2.43E-05	8.91E-05
BCAM	0.351157857	1.04E-16	3.16E-15
OR52D1	0.10257005	0.018621396	0.036304047
FKRP	-0.113482822	0.009189537	0.019265756
TDRD5	-0.090706683	0.037555884	0.067248437
HLA-DRA	-0.127947734	0.003286883	0.007746662
SSX7	0.070663601	0.105486791	0.164378681
NLRP10	-0.024754965	0.573279708	0.661741491
RP11-125A7.3	-0.030336492	0.487517532	0.582992086
Rgr	-0.215676596	5.93E-07	3.04E-06
NLRP5	0.026149475	0.549569294	0.640946936
PDCL2	-0.163660976	0.000163226	0.000507933
NIPBL	0.086489074	0.047412419	0.082285906
ZNF331	-0.018439457	0.673073922	0.750586062
C2orf57	0.030278727	0.488347193	0.583500528
ADCK4	0.118318474	0.006593827	0.014372719
HMGN4	-0.296595618	3.84E-12	4.72E-11
GHRL	-0.327138969	1.38E-14	2.75E-13
EFHC1	0.153521126	0.000410016	0.001169925
EIF3M	-0.249798099	6.33E-09	4.65E-08
SLC17A3	-0.121171706	0.005391128	0.011984832
C8ORFK29	0.146082661	0.000778202	0.00210522
ZNF24	0.142191544	0.001075511	0.002836556
ESRRA	-0.152241874	0.000458742	0.001295136
FUCA2	0.039021164	0.371777172	0.470185939
IRF3	0.082313992	0.059219918	0.099609024
GPR19	-0.288602663	1.51E-11	1.68E-10
EBPL	-0.044657067	0.306653399	0.401364783
GMFG	-0.182493239	2.54E-05	9.29E-05
PIK3AP1	-0.263679605	8.15E-10	6.88E-09
PRSS21	-0.079018898	0.070172632	0.115433792
PHF16	-0.110108035	0.011505019	0.023597185
ZMAT5	0.187377558	1.52E-05	5.84E-05
SLAMF1	-0.196369572	5.70E-06	2.41E-05
MBD5	0.146410274	0.000757013	0.002050604
PHLDA1	-0.01552416	0.722427656	0.792261041
LIF	-0.17946129	3.48E-05	0.000124223
ACTC1	0.013134332	0.763774317	0.827422177
OXTR	0.062279004	0.153771257	0.225556738

USP19	0.096526075	0.026849406	0.050120521
CNTFR	-0.027460881	0.529724268	0.622924942
SUV39H2	-0.354938844	4.62E-17	1.52E-15
ERO1L	-0.02291844	0.599968566	0.68559872
EPX	-0.039360581	0.367628591	0.465804288
TMEM87B	0.196541879	5.59E-06	2.37E-05
LOC124512	0.102949889	0.018187186	0.035581613
AFAP1L1	-0.044222129	0.311393091	0.4059554
ENDOG	0.134130774	0.002050141	0.005052631
FAM47B	0.060260488	0.167579865	0.242858494
WNT3	0.256751376	2.30E-09	1.82E-08
ZNF549	-0.016049411	0.713442826	0.784787109
DPPA5	-0.048792402	0.263976463	0.35348242
LSM12	0.137762211	0.001539514	0.003913382
LGI4	-0.109527823	0.011951377	0.024423069
KRT37	0.396297155	3.16E-21	1.96E-19
NAG18	0.087180874	0.045867968	0.079921263
NACAD	0.053484045	0.220726569	0.304452147
PPP1R2P3	-0.020491915	0.639136204	0.72084529
MFAP5	0.046537721	0.286710211	0.379462137
CST3	0.343769098	4.89E-16	1.31E-14
WDR6	0.193026054	8.26E-06	3.35E-05
CD300A	-0.142298121	0.001066135	0.002814797
VASH1	-0.016890412	0.69913947	0.772682716
CNIH	0.029343809	0.501879952	0.595994559
DHX16	0.034792455	0.425859479	0.5241413
CLEC3B	0.093411772	0.032196821	0.058653169
C9orf102	-0.181560342	2.80E-05	0.000101529
SLC35A5	-0.105226709	0.015765194	0.031291213
SLC22A16	-0.286793108	2.04E-11	2.25E-10
ARL2BP	0.089351674	0.040513328	0.071895081
CRP	0.08471829	0.052154363	0.08908255
SLC10A4	-0.184667905	2.03E-05	7.58E-05
GLA	0.098903356	0.023299619	0.044191658
TTLL11	-0.021582871	0.621393484	0.70484201
C17orf65	0.139980132	0.001288074	0.003337703
NEBL	0.299862775	2.17E-12	2.81E-11
CCDC18	-0.317614579	8.58E-14	1.52E-12
LYSMD2	-0.156079993	0.000326674	0.00095426
THEX1	-0.183317718	2.33E-05	8.60E-05
SAC3D1	-0.011580393	0.791032766	0.850360224
STK40	-0.286764638	2.05E-11	2.25E-10
PIGP	0.186942659	1.59E-05	6.09E-05
EFHA2	-0.116094066	0.007692778	0.01644746
MYH13	-0.053506657	0.220531189	0.304250905
TMED9	0.100936125	0.020593937	0.039609672
UGT2B4	0.115106941	0.008230866	0.017470348
PJA2	0.151231949	0.000500959	0.00140337
PKIB	0.273562187	1.76E-10	1.65E-09
COLEC11	-0.190741921	1.06E-05	4.21E-05
MGC88374	0.309226033	4.06E-13	6.04E-12

SCYE1	0.04607399	0.291544732	0.384866586
MGST1	-0.049801107	0.254218292	0.342279019
CYP7A1	0.03027319	0.488426748	0.583500528
PHF1	0.057840937	0.185331373	0.263735851
LOC644096	-0.123729737	0.004484844	0.010206257
RHOBTB2	0.065787566	0.131846382	0.198075593
SRD5A2	0.054910289	0.208643886	0.290391864
UTP14C	0.02119749	0.627636772	0.710482053
RABEP2	0.300024344	2.11E-12	2.75E-11
FUBP1	-0.133765956	0.002109196	0.005191932
IL27RA	-0.314187419	1.63E-13	2.68E-12
IGLL1	-0.125893398	0.003828332	0.008869786
KIAA0586	0.01506938	0.73023802	0.798831807
MGC34800	0.06922112	0.112809534	0.173851084
SMPD2	0.08118545	0.062799878	0.104649444
FBXO36	0.193045489	8.24E-06	3.35E-05
CSRP3	-0.027123401	0.534796316	0.628168586
MMP20	-0.223919377	2.29E-07	1.27E-06
SEPT3	-0.184272255	2.11E-05	7.87E-05
CBX6	-0.039422107	0.366879668	0.465142903
ALPP	-0.048555753	0.266302614	0.356131964
PRG3	0.127984537	0.003277851	0.007728338
ASH1L	0.055888039	0.200641577	0.281741279
CHRNA2	0.082915221	0.057382722	0.096962451
RBM38	-0.171333304	7.84E-05	0.000260444
RDH8	0.05515007	0.206660399	0.288499834
TTC21B	0.0481767	0.270057756	0.360291851
DGKD	0.023070258	0.597554365	0.684038145
C5orf4	0.062177777	0.154442541	0.226003614
NR1I3	-0.142904792	0.001014181	0.002689176
FAM83H	0.307145761	5.92E-13	8.58E-12
FAM22D	0.039710863	0.363377486	0.461654579
LILRP2	-0.067766462	0.120592823	0.183864436
OPA1	-0.123092557	0.004696698	0.010621552
STRC	0.16057232	0.000217359	0.000659043
MMP23B	0.039950486	0.3604871	0.458740724
TMEM140	0.01214913	0.781024716	0.842252013
FLJ40292	-0.028261273	0.517793285	0.611350021
IFI16	-0.194513331	7.01E-06	2.91E-05
CSTA	-0.041456563	0.342651408	0.439225247
PRPF39	-0.009179424	0.833643619	0.882318131
USP4	0.116261483	0.007604703	0.016281797
CAPN6	-0.282899874	3.89E-11	4.08E-10
NUAK1	0.187488502	1.50E-05	5.78E-05
NPPA	-0.066485165	0.127790138	0.192924518
LAMB3	-0.166923614	0.000119939	0.000385723
PPL	-0.134171039	0.002043716	0.005040838
CCL26	-0.03165449	0.468795443	0.565220232
RALGPS1	0.127586174	0.003376819	0.007934298
LCN1	0.018284538	0.675663799	0.752927999
CCDC6	0.052547112	0.228931462	0.313309495

NCOA3	0.261698272	1.10E-09	9.06E-09
MTHFD1	0.021530094	0.622246904	0.705679862
FCMD	0.13123314	0.002564012	0.006216919
PHF21B	0.238163097	3.22E-08	2.04E-07
C8orf13	0.051017832	0.242784478	0.32864923
S100A3	-0.171154547	7.97E-05	0.000264461
C10orf59	0.089095815	0.041093316	0.072788463
PAFAH1B3	0.301905864	1.51E-12	2.04E-11
ZNF107	0.168616119	0.000101987	0.000330759
ALDH6A1	0.09227411	0.034365307	0.06211413
G6PC2	0.002826101	0.948443371	0.963326443
GRWD1	0.065140073	0.13569964	0.202873107
FLJ22222	0.04537064	0.298981067	0.393416346
BCKDK	0.300172494	2.05E-12	2.69E-11
CTSB	-0.000529984	0.990325011	0.992908608
PFKFB1	0.323099319	3.02E-14	5.74E-13
ZFP36	0.021959698	0.615314946	0.699366286
CMYA5	0.33341144	4.02E-15	8.93E-14
TNF	-0.263453523	8.43E-10	7.08E-09
ZNF417	0.15580532	0.000334796	0.000975206
SIRT2	0.039958765	0.360387493	0.4587089
C1orf198	-0.178606647	3.79E-05	0.000134416
PGAM1	-0.011052822	0.800347261	0.857226147
GRM6	-0.009642867	0.82537653	0.876499907
MEIS1	-0.072147157	0.098353112	0.155070073
KLHL10	-0.066313337	0.128780145	0.194137071
NGFRAP1	-0.138266855	0.001478635	0.003775799
OR13H1	0.074700827	0.086978439	0.139642408
CRYBB3	-0.062826555	0.150178472	0.221079105
NEDD4L	0.125537022	0.003930091	0.009081598
EDAR	-0.185468924	2.16E-05	8.02E-05
C6orf60	-0.252049521	4.58E-09	3.43E-08
IL1A	-0.188280645	1.38E-05	5.37E-05
C20orf160	0.13294626	0.002247601	0.005510564
CACNA1H	0.423850021	2.39E-24	2.54E-22
TXNDC3	-0.113568943	0.00913631	0.019193431
ERCC1	-0.109477858	0.011990521	0.024487013
FAM3B	0.173130087	6.57E-05	0.000221537
CAV3	-0.055455548	0.204153279	0.285758824
CREBBP	0.067801755	0.120399146	0.183733426
BVES	-0.194373791	7.12E-06	2.95E-05
SPACA1	-0.010744308	0.805807455	0.861873376
PARK7	0.132397154	0.002344905	0.005726299
WBP1	0.282140083	4.41E-11	4.57E-10
KCNG4	0.139718406	0.001315641	0.003399108
COQ5	0.286331986	2.20E-11	2.41E-10
TUBA1A	0.001090838	0.980088041	0.985376286
KCNH4	0.014699191	0.736616352	0.80418333
PRMT8	0.091375358	0.036165267	0.065023459
TCEAL6	0.249061505	7.04E-09	5.12E-08
SELP	-0.036147815	0.408046061	0.506357236

RARS2	-0.275257048	1.34E-10	1.29E-09
EPS8L3	-0.028279035	0.517530104	0.611156637
DCLK2	-0.276817223	1.05E-10	1.02E-09
MEMO1	-0.174462628	5.76E-05	0.000197052
LRBA	0.306268968	6.93E-13	9.96E-12
NAPB	-0.037783503	0.387148468	0.486030202
MYST3	0.072465521	0.096873501	0.153129861
KRT8	0.534818638	2.99E-40	3.07E-37
TMIGD2	-0.008584491	0.844283105	0.891703335
LMAN2L	0.116086412	0.007696827	0.016450395
C1GALT1C1	-0.021445318	0.623618798	0.706844606
DPP7	0.148769567	0.000619494	0.001707426
FHIT	-0.031928968	0.464947027	0.561791956
PPOX	0.077957467	0.074035129	0.121010635
ZNF439	-0.200990407	3.38E-06	1.49E-05
EPB49	-0.024133627	0.580769607	0.668129525
ROPN1	-0.431587658	2.82E-25	3.68E-23
LOC51252	0.150952069	0.000513282	0.00143332
C7orf49	-0.062493866	0.152353741	0.223906108
CST8	0.133901268	0.002087113	0.00513963
SENP8	0.074298346	0.088697442	0.142105412
PANK1	-0.306441688	6.72E-13	9.68E-12
GTPBP5	0.221923111	2.72E-07	1.48E-06
LTB4DH	-0.042949519	0.325536482	0.42123818
SPP1	-0.081149994	0.062915181	0.10481318
GLI1	-0.019963409	0.64780699	0.729554062
HYPK	0.378978952	2.07E-19	9.81E-18
ZNF157	0.11978646	0.005947901	0.013118237
SFTPD	0.163940136	0.000159014	0.000496839
SH3BGRL2	0.008467224	0.846383673	0.893154832
TRPA1	0.169006369	9.82E-05	0.000319563
FAM81B	0.129082535	0.003018556	0.007188654
ASPSCR1	0.163513161	0.000165498	0.000514276
PHOSPHO2	0.043284178	0.321777431	0.417428149
FDFT1	0.073519245	0.092102505	0.146605825
PTGS2	-0.313919578	1.71E-13	2.78E-12
BMP7	-0.011756435	0.787931202	0.847767097
CCDC90B	-0.325683113	1.84E-14	3.60E-13
UBE2D3	-0.107877405	0.013305846	0.026887166
SLC25A34	0.071674125	0.100584723	0.157819714
ARFGEF2	0.216234902	5.53E-07	2.86E-06
REXO1	0.054492264	0.212134657	0.294583561
NEFL	-0.012757039	0.770367243	0.832665987
FLJ23861	-0.239767478	2.59E-08	1.67E-07
ZNF561	-0.16545378	0.000137899	0.000437308
COX7B	0.021110887	0.629043459	0.711550447
ENTPD2	-0.072791478	0.095377047	0.151075081
ATP6V1A	0.084170777	0.053698244	0.091382313
TRAPPC5	0.310906189	2.98E-13	4.55E-12
ADH1C	0.033345481	0.445367323	0.542766381
ANKRD17	-0.041816469	0.338473958	0.434591602

IL21R	-0.215486916	6.07E-07	3.11E-06
C6orf48	-0.095891906	0.02787161	0.051761561
TGIF2	-0.031031164	0.477599944	0.57358634
IGF2AS	0.074102062	0.08954562	0.143277652
DNMT3A	-0.151252878	0.000500049	0.001402097
FCAR	-0.152923643	0.000432141	0.001225661
3-Mar	0.052682483	0.227732795	0.312085794
FKHL18	0.121772907	0.00516444	0.011551888
CTSK	0.092812634	0.033323794	0.060480523
TRIM35	-0.02450711	0.574927046	0.663269495
HNF4G	-0.029479731	0.49990029	0.594217453
EXOSC3	-0.114103098	0.008812263	0.018584402
FBXL10	-0.233495361	6.04E-08	3.66E-07
SMCHD1	-0.290275136	1.14E-11	1.31E-10
EIF2C3	-0.242427107	1.79E-08	1.20E-07
POP7	0.081262699	0.062549269	0.104288356
UBE2Q2	-0.028180805	0.518986481	0.61228854
UGT2A3	0.042638139	0.32905953	0.424813574
PGGT1B	0.187091984	1.57E-05	6.00E-05
SYT7	0.186257685	1.71E-05	6.51E-05
DEPDC6	0.210229983	1.15E-06	5.58E-06
OR5U1	0.026589099	0.542876087	0.635353076
SLCO1B1	0.071983247	0.099121882	0.155922347
ZNF565	0.001782271	0.967472376	0.976204076
CCNDBP1	0.066842298	0.125751359	0.190501381
SST	-0.068624707	0.115951556	0.177669105
KCNN3	-0.119467481	0.006083211	0.013378277
GLOD4	0.051412281	0.239156346	0.324486402
DPY19L3	-0.148857464	0.000614849	0.001695384
SCCPDH	0.28884996	1.44E-11	1.63E-10
ZNF790	-0.147037516	0.000717927	0.001952469
OLIG3	-0.011198426	0.797773676	0.855662016
PRMT1	-0.069249101	0.112663784	0.173669995
ITIH3	-0.000127389	0.997674433	0.998486342
TEX10	-0.333633527	3.84E-15	8.60E-14
EDA2R	0.038640201	0.376467882	0.474559452
TNFRSF19	-0.055048649	0.207497688	0.289254882
PLCXD3	-0.044220117	0.311415128	0.4059554
NARFL	0.417176567	1.45E-23	1.31E-21
DENND2A	-0.126773443	0.003587191	0.008371019
RHOV	-0.028528179	0.513845731	0.608112054
C1orf103	-0.034701782	0.42706712	0.525102124
PIM3	0.032053881	0.463201465	0.560564024
KCNAB1	-0.12389916	0.004429986	0.010096361
FLJ20254	0.050013638	0.252194618	0.339777543
DMTF1	-0.162450706	0.000182724	0.000563476
GPR1	-0.000186634	0.996592881	0.997566274
MXRA5	0.074989909	0.085760396	0.137758798
GRM1	0.059053874	0.176266386	0.253061408
RAPSN	-0.102493358	0.018710154	0.036465528
ACOT9	-0.242094149	1.88E-08	1.25E-07

PDE4D	-0.113773976	0.009010692	0.018968417
TRPC4	0.042339162	0.332465332	0.428400949
GEMIN4	-0.219826157	3.54E-07	1.90E-06
CNTN5	-0.033491813	0.444243916	0.541947412
GRTP1	0.24770074	8.54E-09	6.07E-08
C20orf54	0.245797477	1.12E-08	7.81E-08
ITGB8	-0.277338537	9.61E-11	9.47E-10
THEM4	-0.015650161	0.72026877	0.790598476
FRS3	-0.011164767	0.798368414	0.855852054
OR10A6	0.010842328	0.804622442	0.860905411
OTOF	0.03888553	0.373443043	0.471519769
PPIL5	-0.046160698	0.290636644	0.38375021
TEX14	0.187828217	1.45E-05	5.61E-05
ZNF385	0.361823524	1.03E-17	3.66E-16
RRH	0.119861072	0.005916644	0.013058666
CDR2L	0.106415202	0.014617068	0.029229383
PDZD7	0.143826768	0.000939684	0.002504602
SLC19A1	0.100633881	0.020978084	0.040289528
C1orf217	-0.013598519	0.755686412	0.820507973
LIMS1	-0.108137663	0.013083656	0.026499145
FAM89A	-0.254980135	2.99E-09	2.31E-08
MFAP3L	-0.091589441	0.035729402	0.064296193
PIK3CD	-0.319143036	6.43E-14	1.15E-12
DERL2	0.070159018	0.108004306	0.167537456
FHL5	0.001237767	0.977406714	0.982841191
ACAN	-0.041268826	0.344843477	0.441574873
BRWD2	-0.120930639	0.005484512	0.012188025
TINAGL1	-0.271115146	2.58E-10	2.35E-09
DCUN1D2	-0.073753002	0.09107005	0.14529826
C3orf36	0.006344082	0.884588593	0.918771407
MGC10850	-0.194393367	7.10E-06	2.94E-05
hCG_31916	-0.190696759	1.06E-05	4.23E-05
FHAD1	0.088907144	0.041525478	0.073436919
LCE1C	0.005638698	0.897345651	0.926890375
ARPC1A	-0.026940165	0.537560398	0.630692404
CHST2	-0.385750619	4.16E-20	2.18E-18
SPATA2	0.265115301	6.54E-10	5.61E-09
PGLYRP4	-0.159613821	0.000237314	0.000711527
RUFY1	0.114211292	0.008747887	0.01849751
TXNDC12	-0.216528167	5.34E-07	2.78E-06
RPS4Y1	0.034731725	0.4266681	0.525031449
TNFRSF8	-0.202394941	2.88E-06	1.29E-05
PTGIR	0.009823302	0.822163012	0.874045706
FOXE3	0.085651769	0.049607542	0.085492371
ART4	0.03417158	0.434168429	0.532025043
ZC3H12C	-0.329903706	8.05E-15	1.69E-13
KIAA1841	-0.106154286	0.014862627	0.029691453
EVX1	0.089420833	0.040357752	0.071701768
WDR38	0.098255112	0.024224823	0.045684453
LOC402057	-0.341484063	7.84E-16	2.01E-14
ACAA2	-0.16467919	0.000148352	0.000466367

GLCE	0.068814069	0.114946616	0.176525161
GPR18	-0.214307458	7.01E-07	3.56E-06
HIST1H2AG	0.36636795	3.74E-18	1.46E-16
PIGK	-0.130864888	0.002637121	0.006371574
C16orf67	0.060476939	0.166056133	0.24104796
DAG1	0.134728815	0.001956596	0.004849298
OR4D2	0.037616287	0.389254459	0.488374957
C21orf81	0.101645487	0.019716289	0.038160359
PLOD2	-0.046354693	0.288611837	0.381568305
TTC27	-0.232756864	6.67E-08	4.00E-07
TSPAN2	-0.069199583	0.112921816	0.173936936
PI3	-0.232868559	6.57E-08	3.95E-07
ZFAND6	0.050634614	0.246346079	0.332992314
C6orf57	-0.002047844	0.9626288	0.973233228
NUF2	-0.095730577	0.02813693	0.052206996
ARID2	0.18100318	2.97E-05	0.000107161
RCC1	0.073948731	0.090212713	0.144157476
CD86	-0.087178746	0.04566605	0.079591989
FAM91A1	0.147712419	0.000677965	0.001855277
CALM2	-0.064485349	0.139683526	0.207667795
GYG2	-0.030294161	0.488125438	0.583492092
PARS2	-0.192842815	8.42E-06	3.41E-05
INTS12	0.06333139	0.146922863	0.216909648
CTSF	0.171031478	8.07E-05	0.00026708
BNIPL	0.13786532	0.001526891	0.003886114
GNA13	0.110999843	0.010847691	0.022443625
HUNK	0.062212379	0.154212827	0.225882485
ZBTB4	0.151540004	0.000487714	0.001371716
B4GALT4	0.055116647	0.206936055	0.288733787
CHD1L	-0.291029294	9.99E-12	1.16E-10
MSTO1	0.027174945	0.534020084	0.627376671
FUT8	0.349065826	1.62E-16	4.80E-15
AGA	0.032517575	0.456753618	0.554069441
TRMT11	-0.416877144	1.57E-23	1.40E-21
WWP1	0.483620884	3.46E-32	1.64E-29
B9D2	0.124361564	0.004283333	0.009791157
STAT1	-0.088443978	0.042602689	0.0750613
PTTG1	-0.029844435	0.494608971	0.589089556
TMEM62	0.278590832	7.85E-11	7.88E-10
SSBP2	0.194156568	7.29E-06	3.00E-05
MRFAP1	0.284529083	2.97E-11	3.19E-10
NME4	0.214287347	7.03E-07	3.56E-06
LOC55565	0.005802506	0.89438066	0.924293559
DLL4	0.110828219	0.010971542	0.022654133
MYOCD	0.121333496	0.005329258	0.011855865
HTR3D	-0.071570527	0.101405175	0.158863801
C9orf156	0.019986255	0.647431174	0.729264387
CHMP4C	-0.00392457	0.928450106	0.949449477
PROCA1	0.105736897	0.015262986	0.030412217
GCDH	0.036774321	0.399963799	0.498354083
APOF	0.229905375	9.72E-08	5.69E-07

WEE1	-0.079709519	0.067749205	0.111986522
SSR4	0.079158675	0.069676504	0.114771182
RGS1	-0.088772455	0.041836335	0.073901645
ACCN4	0.003350399	0.93889579	0.956472865
FLJ20489	0.19106372	1.02E-05	4.08E-05
ZNF215	-0.175278525	5.31E-05	0.00018319
AGPAT6	0.174062064	5.99E-05	0.000204142
PDE7B	-0.08938862	0.040430154	0.071788915
BBX	-0.041338226	0.344032113	0.440709221
MS4A3	-0.022224832	0.6110538	0.695551614
OR4A16	0.032703635	0.454180591	0.551601117
EFEMP1	-0.028189373	0.518859365	0.612256042
TULP2	-0.193469585	7.86E-06	3.22E-05
RERE	0.036062095	0.409159387	0.507549137
BNC1	-0.133637187	0.002130409	0.005239954
PIGB	0.071429166	0.101756074	0.159372924
COMMD8	-0.104222443	0.016796245	0.033123833
TRIP11	0.001402912	0.974393235	0.980773285
FLJ40142	0.01647161	0.706249327	0.779170776
PCDHB6	0.004067297	0.925855375	0.947470664
FKBP8	0.026510097	0.544075897	0.636393892
FLJ12716	0.139027403	0.001391053	0.003571433
POT1	-0.128034594	0.003265603	0.00770537
KIAA1109	0.126293291	0.003716993	0.008634601
PTPRC	-0.207296773	1.63E-06	7.63E-06
UNQ9391	-0.095994027	0.028306298	0.05239477
CCT7	0.044916484	0.303849237	0.398627898
EEF1A2	0.442300025	1.33E-26	2.33E-24
MIPEP	0.145873302	0.000792029	0.002136984
ZFX	0.126487425	0.003664007	0.008527623
UCHL3	-0.368712034	2.21E-18	9.04E-17
LOC388419	0.009362035	0.830383881	0.880290394
GSG1L	0.069260832	0.112602725	0.173619398
RAB24	0.200949636	3.40E-06	1.50E-05
SLA2	-0.180138235	3.24E-05	0.000116377
SDS	0.198264343	4.61E-06	1.99E-05
LYPLA3	0.210236551	1.15E-06	5.58E-06
CASQ1	0.061099946	0.161728394	0.235488491
SLC25A40	-0.092855236	0.033242564	0.060368732
IRAK1BP1	-0.239809663	2.57E-08	1.66E-07
ACOT6	0.217099905	4.97E-07	2.61E-06
COL9A3	-0.268371492	3.96E-10	3.54E-09
ASB11	-0.018201572	0.677052368	0.753655868
C2orf18	0.238233851	3.19E-08	2.02E-07
FOXD2	0.309125814	4.13E-13	6.12E-12
C6orf211	0.375192498	5.01E-19	2.26E-17
OR8G1	0.044002476	0.313804949	0.408464571
MDGA1	0.176765879	4.57E-05	0.000159796
ADARB1	-0.08206432	0.059997023	0.100696911
GGT1	0.051995581	0.233861353	0.318817805
WNT1	-0.050984036	0.24309711	0.328962176

DBP	0.26332685	8.60E-10	7.20E-09
COL5A3	0.174691488	5.63E-05	0.000192953
RHOD	0.216362812	5.45E-07	2.82E-06
COL4A2	-0.108556068	0.012733307	0.02584916
LOC201164	0.128851198	0.003071583	0.007295157
HEBP1	0.186284891	1.71E-05	6.50E-05
LUM	0.094965359	0.029425158	0.054220946
ZCCHC6	-0.089997567	0.039079914	0.069673063
PAGE1	0.034880011	0.424695244	0.523127215
DTX2	0.018183499	0.677354995	0.753719845
SLC7A13	0.283907401	3.29E-11	3.50E-10
H3F3A	0.01317162	0.76312366	0.826862975
RABIF	0.170676948	8.35E-05	0.000275434
D4S234E	-0.19439765	7.10E-06	2.94E-05
DYRK3	-0.042439688	0.331317662	0.427190669
PFAS	0.055876554	0.200734257	0.281742741
ALOXE3	0.211806432	9.49E-07	4.70E-06
RPLP0	-0.116804847	0.007325026	0.015743301
RBM34	-0.310668078	3.12E-13	4.73E-12
C12orf28	0.15724056	0.000294343	0.000868482
U2AF2	0.200807032	3.45E-06	1.52E-05
MKNK2	0.089090006	0.041106566	0.072788463
SEC16A	0.240359622	2.38E-08	1.55E-07
ZNF44	0.118429891	0.006542683	0.014271358
YWHAG	0.226432269	1.53E-07	8.69E-07
IGF2BP2	-0.204261412	2.32E-06	1.06E-05
OR1D5	-0.055840458	0.201025755	0.282087486
SIX6	-0.16500936	0.000143808	0.000453289
CCR6	-0.112618064	0.009739493	0.020342439
PALM	0.340825546	8.98E-16	2.28E-14
PUM2	-0.114729862	0.00844512	0.017918923
SPRYD5	0.05550526	0.205920323	0.287904517
ALG10B	0.073734398	0.091151876	0.145356039
ZNF365	0.247329791	9.00E-09	6.37E-08
PHC1	-0.287768829	1.73E-11	1.93E-10
KIAA0913	0.043525714	0.319082009	0.414543688
ARX	0.137150645	0.001616374	0.004085114
PPP3CB	0.092818336	0.033312912	0.060478624
IRX6	-0.149966463	0.000558957	0.001552406
ANGPTL4	-0.121834991	0.005141527	0.0115132
LSM14B	0.054985343	0.208021559	0.289722438
PCDHGB7	0.02160414	0.621383309	0.70484201
INSM1	0.031400455	0.472372852	0.568641477
WBP2NL	-0.009276559	0.832384496	0.881746766
ZNF493	-0.113552661	0.009146352	0.019206115
NGEF	-0.014993855	0.731537831	0.799969078
RNASE13	-0.038136631	0.382723832	0.481655515
SPPL2A	0.174142327	5.94E-05	0.000202972
SFXN1	0.152286124	0.00045697	0.00129132
FAM102A	0.457066199	1.64E-28	4.03E-26
SAPS2	-0.08564536	0.049624667	0.08549792

JTV1	0.095638603	0.028289158	0.052394588
OR51B4	0.056832107	0.193129157	0.272812126
SCGB1A1	0.079082052	0.069948116	0.115156885
NEUROD2	-0.052407288	0.230174248	0.314625284
tAKR	0.025367726	0.561570593	0.652143074
C1orf26	-0.105532935	0.015462047	0.030768974
RICH2	-0.137936133	0.001518277	0.003867392
TEDDM1	0.011590206	0.790859783	0.850322925
CYP2S1	-0.000688875	0.987424622	0.991131897
TBCE	0.000379082	0.993079685	0.995021506
MAPK1	-0.250957258	5.36E-09	3.97E-08
HDHD1A	0.230878634	8.55E-08	5.05E-07
MRM1	0.21374382	7.51E-07	3.79E-06
ATP9A	0.310926759	2.97E-13	4.55E-12
HSD17B3	0.071145643	0.103125326	0.161271336
HN1L	0.331491594	5.89E-15	1.27E-13
RNF216	-0.00606481	0.889635936	0.921549293
HOXD12	0.043424663	0.32299698	0.418745188
PPP1R14B	-0.17718372	4.38E-05	0.000153704
SBF1	-0.145734813	0.0008013	0.00216105
TAS2R42	0.000620829	0.988775464	0.991995742
USP46	-0.148502002	0.000633834	0.001743042
LILRB3	-0.167978932	0.000108426	0.000351086
SPI1	-0.079703395	0.067770388	0.111991431
OXSM	0.009637658	0.825469338	0.876499907
GYS2	-0.094314599	0.030560189	0.056010314
NUPL2	-0.246996176	9.44E-09	6.66E-08
C8orf46	-0.235403242	4.68E-08	2.89E-07
SF3A1	-0.293438677	6.62E-12	7.98E-11
C21orf99	-0.053834631	0.217711147	0.301103429
HOXB4	0.055147804	0.206679078	0.288499834
YRDC	-0.141232708	0.001163355	0.003041442
GPRC5D	0.204300076	2.31E-06	1.05E-05
BLVRA	0.418359863	1.06E-23	1.02E-21
KIF12	0.237181174	3.68E-08	2.31E-07
LRRC23	0.137644924	0.001553989	0.003943655
FAM14A	-0.087847524	0.044024518	0.077190408
RASL12	-0.129382415	0.002951059	0.007048036
DAZAP2	0.283291893	3.65E-11	3.85E-10
IKBKB	0.211157301	1.03E-06	5.05E-06
ZNF271	-0.113591926	0.009122152	0.019170237
BOK	0.020857418	0.633168236	0.715558075
CXorf6	-0.228514668	1.17E-07	6.75E-07
MYEOV	0.019125012	0.661659781	0.740948096
BTN2A2	-0.176271673	4.80E-05	0.000167439
FRG1	-0.063827502	0.143776208	0.213082647
HSP90AB6P	0.19015469	1.13E-05	4.47E-05
ENOX1	-0.020625851	0.636946546	0.718771208
ZNF706	0.165169377	0.000141654	0.000447369
DOK1	0.150961721	0.000512852	0.001432771
PGAP1	-0.019848561	0.649697586	0.731281431

TMEM136	-0.090841984	0.037270901	0.066757579
FSCN1	-0.333780618	3.73E-15	8.38E-14
KIF17	-0.108088842	0.013125086	0.026556812
TRIM66	-0.070361529	0.106988278	0.166254971
CBR3	-0.205262656	2.06E-06	9.51E-06
C13orf24	-0.196893929	5.38E-06	2.29E-05
C19orf52	-0.144154346	0.000914456	0.002441593
BNIP1	0.221980352	2.70E-07	1.47E-06
AQP3	0.031093476	0.476715755	0.572860109
KRT6C	-0.26835756	3.97E-10	3.54E-09
SIRPA	-0.234549215	5.25E-08	3.21E-07
IGFBP6	0.122884327	0.004767866	0.010758756
PLEKHK1	-0.194336184	7.15E-06	2.96E-05
RNASE7	-0.066742042	0.126321125	0.1909881
ARHGEF15	0.113548012	0.009149221	0.019206115
NPHS2	-0.048127399	0.27054882	0.360712205
SRD5A1	-0.330361223	7.36E-15	1.56E-13
REXO4	-0.033141376	0.448159328	0.545581411
EEF1DP3	0.130343334	0.002743919	0.006598498
SLC37A2	0.046814654	0.283848993	0.376242177
ZNF142	-0.124937457	0.004106831	0.009447402
ANKHD1	0.077212962	0.076846412	0.124974501
MUT	0.031045055	0.477402761	0.573461531
VPS37A	0.10410982	0.016915475	0.0333269
GPRIN1	0.048036457	0.271456236	0.361701108
SLC38A3	-0.134565451	0.001981753	0.00490177
BAZ2B	-0.023848538	0.585247967	0.671898758
WDR87	0.002612166	0.95234137	0.966009087
BRD7	-0.106901811	0.014168617	0.028434343
POU6F2	0.096751421	0.026494031	0.04953232
NISCH	0.085340987	0.050443647	0.086593519
TCEB1	0.156230605	0.000322298	0.000943271
LINGO2	-0.129354169	0.002957357	0.007059313
TAX1BP3	-0.075244066	0.084700893	0.136163606
RPL34	-0.161497206	0.000199602	0.000608503
MARK2	0.092582795	0.033764966	0.061154868
AKAP12	0.088525029	0.042412502	0.0747905
AMBN	-0.06686212	0.125638943	0.190377984
SLC25A27	-0.358732176	2.03E-17	6.89E-16
FLJ21865	0.09975952	0.022124647	0.042211998
KIR2DS2	-0.183313601	2.34E-05	8.60E-05
WDR77	-0.217725035	4.60E-07	2.43E-06
ATF2	-0.168635469	0.000101797	0.000330318
ITFG3	0.381529491	1.14E-19	5.64E-18
SLC39A13	0.083549773	0.055495215	0.094135193
ARL6IP5	-0.077906571	0.074224608	0.121255875
C10orf137	-0.159734858	0.000234703	0.000704338
QTRT1	-0.123069839	0.004704416	0.010635093
CCNT1	0.241596878	2.01E-08	1.33E-07
DYNLL1	0.178710612	3.75E-05	0.000133104
WDR53	-0.10432757	0.016685616	0.032926783

LIPG	-0.357340906	2.75E-17	9.23E-16
ASAH3	0.09599904	0.02769661	0.05146765
HELB	-0.055513279	0.203681952	0.28522895
PHACTR2	-0.133565994	0.002142221	0.0052669
VENTX	-0.081413987	0.062060852	0.103614494
LAD1	-0.180201243	3.22E-05	0.000115695
PAOX	0.164769369	0.000147097	0.000463135
MAPK8	-0.095665684	0.028244263	0.052363397
CCDC38	-0.008282248	0.849699281	0.895577799
DNAJC8	-0.173757612	6.17E-05	0.000209585
RBBP8	-0.0266138	0.54250123	0.635156143
WNT11	-0.132796935	0.00227369	0.005572308
KCNJ12	-0.038897705	0.373293317	0.471519769
HDAC8	-0.100736643	0.020846783	0.040058396
STARD4	-0.198069885	4.71E-06	2.03E-05
ACVR1	-0.041813163	0.33851218	0.434591602
C14orf65	-0.051517282	0.238197017	0.323584989
KLB	0.099159745	0.02294222	0.043567544
C1orf65	0.013035316	0.765502923	0.828856749
ZFYVE28	0.252771467	4.12E-09	3.12E-08
NSUN6	-0.187324798	1.53E-05	5.87E-05
KIF27	-0.027027824	0.536237193	0.629587067
SYTL2	0.404695856	3.79E-22	2.65E-20
UBXD2	0.039290105	0.368487623	0.466604282
OR6T1	-0.223693101	2.17E-07	1.21E-06
CCDC91	-0.028525893	0.513879462	0.608112054
GRID2	0.032536728	0.456488377	0.553856952
CALN1	-0.051648338	0.237003436	0.322490402
ZNF423	0.020840065	0.633451043	0.715715653
PSMB4	-0.091856494	0.035191959	0.063459049
XPNPEP3	0.060640668	0.164910457	0.239603522
ARPP-21	0.092792609	0.033362037	0.060532064
SART1	-0.072564667	0.096416359	0.152524875
RACGAP1P	-0.067396737	0.122636387	0.186379422
SPTA1	-0.093538342	0.031962956	0.058303239
C6orf113	-0.140552115	0.001229669	0.003197793
C7orf16	-0.167255883	0.000116195	0.000374665
CHST7	-0.041561797	0.341426566	0.437746445
C21orf29	0.101491821	0.019903597	0.038426128
SEMA6D	-0.115134101	0.008215623	0.017444014
PCMTD1	0.079011374	0.070199421	0.115446974
KIAA1754	-0.12428839	0.004306244	0.009839871
MYCN	-0.159870129	0.000231816	0.000698402
KCNJ3	0.400999724	9.71E-22	6.35E-20
MAPK13	0.107024217	0.014057733	0.028239465
ERO1LB	0.073748007	0.091092014	0.14529826
NTF3	-0.147296172	0.000702361	0.001912673
NKX6-2	-0.040607468	0.352636521	0.450747641
GTF2B	-0.195069191	6.59E-06	2.75E-05
GSPT1	0.051418302	0.239101269	0.324483271
GUSB	0.067169558	0.123905388	0.188029178

LOC221091	-0.02390171	0.584411484	0.671416846
LIG1	0.043500085	0.319367314	0.414739094
EXTL3	-0.2318674	7.51E-08	4.47E-07
NID2	0.116173274	0.007650995	0.016368191
TTC29	0.148206319	0.000650038	0.00178521
TMEM97	0.10586898	0.01513528	0.030177314
EXTL2	-0.277863761	8.83E-11	8.79E-10
SUZ12	-0.026451537	0.544966093	0.637071579
IL1F8	-0.002306497	0.957912766	0.969995725
KRT18	0.569311882	1.63E-46	2.50E-43
MRPS16	0.0594024	0.173723634	0.250049875
PI4K2B	-0.11962502	0.006016041	0.01325901
LACRT	-0.140730804	0.00121193	0.003159031
OR51F2	0.039571168	0.365069163	0.463325136
JMJD2C	-0.138270279	0.001478229	0.003775799
KGFLP1	-0.220075983	3.43E-07	1.84E-06
CDK5RAP3	0.282871241	3.91E-11	4.09E-10
YTHDF2	-0.09485154	0.029621027	0.054549174
GGCX	0.040970629	0.348343586	0.445685542
ARPC4	0.101464895	0.019936576	0.038465643
EGLN2	0.135661946	0.001818417	0.004539766
KBTBD4	0.066907506	0.125381847	0.190035242
ROBO3	-0.197732661	4.89E-06	2.10E-05
DEFB118	-0.032114353	0.463643135	0.560877756
KIAA1543	0.125886011	0.003830417	0.008871274
RTCD1	-0.161631417	0.000197141	0.000602195
MZF1	0.280071639	6.18E-11	6.29E-10
C18orf26	-0.006670953	0.878801977	0.914626503
CNIH4	-0.072533289	0.09656085	0.152714164
ZFP2	0.02277174	0.60230554	0.687885729
HTATSF1	-0.070038281	0.108613676	0.168355304
WFDC2	0.047512231	0.276727456	0.367912892
NDUFA7	0.279330296	6.97E-11	7.06E-10
TTC22	-0.34665321	2.69E-16	7.75E-15
FAM40B	0.007908598	0.856404806	0.900022757
DCPS	-0.289515509	1.29E-11	1.48E-10
SH2D1B	-0.179780325	3.37E-05	0.000120374
MRGPRE	0.03981278	0.362146376	0.460490643
SBK1	-0.089213347	0.040826026	0.072408201
UNQ6411	0.084795021	0.051940982	0.088803656
OSBPL9	-0.230117871	9.45E-08	5.54E-07
NUP107	0.000812514	0.985167832	0.989351135
MYOZ3	0.114101596	0.008813159	0.018584402
PDE4B	-0.457116298	1.61E-28	4.03E-26
FAM113A	0.141844449	0.001106579	0.002906601
IDH3G	0.146321536	0.000762699	0.002064188
FBXL7	0.316069037	1.15E-13	1.95E-12
ARFGAP3	-0.322584616	3.34E-14	6.26E-13
MAPRE2	-0.286997131	1.97E-11	2.18E-10
IL1RN	-0.00314728	0.942593672	0.959322806
KIF13A	-0.047489679	0.276955771	0.36805728

RAC3	0.038923709	0.372973654	0.471314221
TCTE1	0.09643166	0.026999517	0.050367716
TMEM14B	-0.094391718	0.030423751	0.055849797
ADIPOR1	0.304829657	8.98E-13	1.26E-11
GRINA	0.406820851	2.20E-22	1.65E-20
CLIP4	-0.268154798	4.10E-10	3.64E-09
LRIT2	0.04933964	0.260019927	0.349096622
TFPI	-0.022993915	0.598767824	0.68462524
FABP6	0.090228529	0.03857788	0.06887787
SLITRK2	-0.064713274	0.138286581	0.206089235
HKR1	0.053972271	0.2165354	0.299747001
SMTN	-0.026261433	0.547860907	0.639361684
C1orf75	-0.186275469	1.71E-05	6.50E-05
CD209	-0.129228485	0.002985532	0.007115518
CYB5R2	-0.285598416	2.49E-11	2.71E-10
DNTTIP2	-0.266536177	5.26E-10	4.60E-09
CSGlcA-T	0.029008283	0.50678444	0.601006273
GABRB3	-0.008846212	0.839599017	0.887518369
PCBD1	0.223668292	2.18E-07	1.21E-06
TAF3	0.054558213	0.211581168	0.294014148
HOXD3	0.00030105	0.994504157	0.996286423
GIPC3	0.15485951	0.000364228	0.001051967
P11	-0.117864697	0.006805848	0.014773239
BFSP1	-0.043377536	0.320733845	0.416162147
LCP2	-0.135582587	0.001829811	0.004562654
TAS2R8	-0.05131787	0.240470757	0.325982073
SEZ6L	0.155280784	0.000350833	0.0010171
NR2C1	-0.135390403	0.001857675	0.004624633
EXDL2	0.208479506	1.41E-06	6.73E-06
TNFRSF13B	-0.115881232	0.007806062	0.016649141
MKI67	-0.056357698	0.196878097	0.277216263
GLS	-0.198290431	4.59E-06	1.99E-05
C7orf54	0.015947071	0.715190362	0.785865892
LGALS13	-0.024434365	0.576062855	0.664081458
IL4R	0.062791207	0.150408467	0.221364688
SEC11A	-0.010105188	0.817148632	0.870392577
SPP2	0.063314099	0.147033471	0.216968758
C18orf32	0.197877909	4.81E-06	2.07E-05
CLSPN	-0.099487552	0.02249219	0.042831985
SPAG1	0.214188296	7.11E-07	3.60E-06
C9orf82	-0.194697713	6.87E-06	2.86E-05
TM4SF1	-0.235806682	4.43E-08	2.75E-07
EMILIN2	-0.233742644	5.85E-08	3.55E-07
SMG7	0.18425646	2.12E-05	7.87E-05
TAS2R13	-0.043120452	0.323612937	0.419366902
ZNF628	0.018636445	0.669786331	0.747733505
DZIP1L	-0.078049089	0.073695032	0.120550878
ANKRD13A	-0.016937651	0.69833915	0.772317884
VASP	0.067701007	0.120952657	0.184184718
ZCCHC11	-0.362610451	8.65E-18	3.13E-16
SYPL1	-0.012035514	0.783021185	0.843960959

MGC34774	0.004368739	0.920377836	0.94433561
C4orf28	-0.172486081	7.00E-05	0.000234893
KIAA1211	0.117952936	0.006764147	0.014697082
RPS27L	0.187052986	1.57E-05	6.02E-05
TATDN3	0.033876241	0.438153393	0.536266962
PDCD1	-0.172886652	6.73E-05	0.000226549
OR5P2	-0.058074294	0.183561162	0.261701272
IFIT1L	0.307728635	5.33E-13	7.76E-12
MIPOL1	0.119573749	0.006037828	0.013297495
OR51D1	0.152292513	0.000456715	0.001291192
C1orf92	0.059568919	0.172518449	0.248725895
LAMP2	-0.052485812	0.229475716	0.31384479
CAT	-0.152807211	0.00043658	0.00123711
C16orf80	-0.122428829	0.004926947	0.011077074
C15orf32	0.077727889	0.076296139	0.124178126
ZNF746	0.149829651	0.000565588	0.001568696
C1orf76	-0.050081127	0.251554354	0.339212221
ATXN1	-0.00207014	0.962222236	0.972982162
LAMC2	-0.190557546	1.08E-05	4.29E-05
SLC2A7	0.004652242	0.915229716	0.940465829
CPOX	-0.159936682	0.000230409	0.000694842
APH1B	0.267176064	4.77E-10	4.20E-09
LOC442245	0.047613605	0.275702728	0.366788419
CTNND1	0.103835238	0.017209271	0.033829862
GABRG2	0.032897429	0.451509303	0.548790414
MADCAM1	-0.10466266	0.016337235	0.032301498
F5	-0.160777391	0.000213298	0.00064737
SEMA4F	0.020532107	0.638478788	0.720368086
NUDCD3	0.273597265	1.75E-10	1.64E-09
PDZD11	0.264902553	6.76E-10	5.78E-09
TRIML1	-0.012841378	0.769536394	0.832307366
GCNT3	-0.130383396	0.002735578	0.006586167
TMEM120A	0.20167445	3.13E-06	1.39E-05
CNDP1	-0.016736913	0.701742349	0.774966541
N4BP1	0.005912623	0.892388323	0.923320848
SLC35F2	-0.335813641	2.48E-15	5.72E-14
LCP1	-0.216393746	5.43E-07	2.82E-06
IGBP1	0.122554096	0.004882727	0.01098569
DCAKD	0.079466613	0.068593627	0.113169362
ELA2A	0.042918021	0.325891738	0.421599198
C12orf56	-0.037191475	0.394635926	0.492873125
PITRM1	-0.081899764	0.060513802	0.101444757
GUK1	0.131265434	0.002557689	0.006207941
RASSF8	0.066583935	0.127223735	0.192210994
OR2A14	-0.070626288	0.105671354	0.164624565
ADM	-0.197517281	5.01E-06	2.15E-05
FGD3	0.216509801	5.35E-07	2.78E-06
GHRHR	-0.117476789	0.006991914	0.015122505
RHPN2	-0.019346088	0.657995476	0.737381845
C4orf39	-0.264583212	7.10E-10	6.04E-09
VPS72	-0.089397359	0.040410501	0.071774746

SERF2	0.363934443	6.44E-18	2.40E-16
CD22	0.17004124	8.89E-05	0.000291896
CD47	-0.085517586	0.049967107	0.08599224
PPIC	0.059349315	0.174109154	0.250389585
IMPDH1	0.099806708	0.022061409	0.042129069
ACP6	0.013719625	0.753580634	0.818748003
PRKACA	0.08586776	0.049033307	0.084692643
PPP1R1A	-0.314238684	1.61E-13	2.66E-12
TRPV3	-0.030507	0.485073036	0.58085961
ASXL1	-0.222022881	2.69E-07	1.47E-06
C17orf55	0.2169787	5.05E-07	2.65E-06
FXYD1	-0.037860329	0.386183212	0.485313831
LMOD2	0.00694748	0.873699491	0.911724372
ANKRD33	0.067562143	0.121718834	0.185168013
LCE2C	0.126285604	0.003719105	0.008636245
ZNF620	0.04243179	0.331407743	0.427217235
DKFZP566E164	0.056900784	0.192590805	0.272176708
VSIG2	0.008417363	0.847277145	0.893944263
KIAA1128	-0.1227226	0.00482381	0.010869039
USO1	0.019680933	0.652461058	0.732916158
NUDT4	0.284003452	3.24E-11	3.45E-10
CLDN1	-0.222859575	2.42E-07	1.33E-06
OR4Q3	-0.059573584	0.172484773	0.248725895
FASTK	0.292785754	7.40E-12	8.86E-11
ICOS	-0.17313103	6.57E-05	0.000221537
LDB1	0.112472015	0.009835208	0.020507527
GSTA5	-0.130000604	0.002816229	0.006756533
ABCC1	-0.00740498	0.865459056	0.906752187
FAM54A	-0.103895501	0.017144412	0.033713139
PCBP2	0.064223685	0.141300586	0.209777461
NUP205	-0.180880839	3.01E-05	0.000108393
ACTA1	0.14949851	0.00058194	0.001612594
GABBR2	-0.299178364	2.45E-12	3.15E-11
PIP5K1B	-0.254974129	2.99E-09	2.31E-08
AGXT	0.053311647	0.222220288	0.306031927
RNF181	0.315025107	1.39E-13	2.33E-12
ATP8A2	-0.068893349	0.114527913	0.17610206
AFTPH	0.253055383	3.96E-09	3.00E-08
FGF21	-0.063516846	0.145740492	0.215422665
FCER1G	-0.1164362	0.00751375	0.016109501
SNTB1	-0.221135621	3.00E-07	1.63E-06
SLC24A3	0.099868868	0.021978346	0.041996535
TXNL4B	-0.205895264	1.92E-06	8.88E-06
RPL10L	-0.143897816	0.000934158	0.002492035
LOC389517	0.175329684	5.28E-05	0.000182563
TSGA13	-0.053228859	0.223382627	0.307288539
SHOX2	-0.090092755	0.038872341	0.069323092
ITGA7	-0.147713473	0.000677904	0.001855277
KCNIP2	-0.017270297	0.692712943	0.766923279
KLF13	-0.144155961	0.000914333	0.002441593
ZFAND2A	0.15439636	0.000379504	0.001091475

CEACAM1	-0.145682415	0.000804834	0.002169629
PFKFB4	0.199463601	4.02E-06	1.76E-05
MED19	-0.011710723	0.788736248	0.848484812
LRRC57	0.037118632	0.395563171	0.493594118
RNF11	-0.085495864	0.050025518	0.08599224
ANKRD32	0.125433492	0.003960109	0.009140658
P117	0.14744131	0.000693763	0.001890936
OBFC2A	-0.375999882	4.15E-19	1.89E-17
POLD3	-0.234129945	5.55E-08	3.38E-07
RAB18	0.239754353	2.59E-08	1.67E-07
TPH2	0.059857894	0.170441803	0.246489935
PHB	0.21429327	7.02E-07	3.56E-06
JDP2	-0.166852119	0.000120759	0.000388158
MORF4L1	-0.086431388	0.047560994	0.082497193
POU2F1	0.039435537	0.36671631	0.465127597
CNNM2	0.091089607	0.036754084	0.065966394
LOXHD1	0.031852579	0.46601631	0.562531271
ZC3H15	-0.215375763	6.15E-07	3.14E-06
ELK3	0.151749171	0.000478906	0.001348967
FAM111B	-0.071534648	0.101250361	0.158661689
CBLC	0.062464478	0.152547031	0.224029542
SBNO1	0.026799911	0.539680942	0.632576842
ANKMY2	0.074117568	0.089478377	0.143207325
PLEKHA5	0.050648723	0.246214309	0.332887376
DHX58	0.187176475	1.55E-05	5.95E-05
ARCN1	-0.120615428	0.005608815	0.012424852
TREML1	-0.033844658	0.438580781	0.536683228
KNCN	-0.009066182	0.836131358	0.884460988
SEC24A	0.048851928	0.263393557	0.352932443
PSCA	0.192162848	9.07E-06	3.65E-05
MGC24125	0.02950595	0.499518889	0.593993744
DNA2L	-0.214981852	6.45E-07	3.29E-06
CIB4	-0.115281224	0.008133489	0.017281556
HIGD2A	0.177940534	4.06E-05	0.000143028
TBX6	0.2170127	5.03E-07	2.64E-06
TTLL5	0.149138322	0.000600224	0.00165803
SGK3	0.103006358	0.018123395	0.035479388
GCN1L1	0.048953239	0.262403508	0.351835842
AMOT	-0.150671274	0.000525928	0.001467967
LDOC1	0.053554418	0.220118904	0.30388665
NRK	-0.036508997	0.403374845	0.501993913
ASB9	-0.29716301	3.48E-12	4.30E-11
NAT1	0.368247878	2.45E-18	9.91E-17
TRAFD1	0.235540457	4.60E-08	2.85E-07
PEAR1	0.126515849	0.003656307	0.008512924
FAM36A	-0.103492934	0.017581772	0.034506964
OR1S2	-0.026843624	0.53901959	0.631922109
LOC388323	0.089181294	0.040898776	0.072516313
PGS1	0.159762529	0.00023411	0.000703244
LEPREL1	-0.289825967	1.22E-11	1.41E-10
TFF1	0.320156802	5.31E-14	9.62E-13

HAP1	0.004133689	0.924648652	0.947089031
EPHB2	-0.087779488	0.044189213	0.077412955
ACTG1	0.174086187	5.97E-05	0.00020388
ZFP42	-0.122961458	0.004741392	0.010702944
HAVCR2	-0.06430901	0.140771718	0.20913392
NME1	0.187583638	1.49E-05	5.74E-05
SNX26	0.077861608	0.074392326	0.121400852
LACTB	0.0593971	0.173762094	0.250049875
ZKSCAN2	0.229353574	1.05E-07	6.07E-07
C5orf35	0.042017998	0.336149091	0.432241899
ANKS3	0.034635722	0.427948198	0.525794933
RBM28	-0.173724571	6.19E-05	0.000210157
DKFZP586P0123	-0.087312673	0.045333265	0.07907922
HNRNPA1	-0.070475661	0.106419	0.165537675
BCAS3	0.291814436	8.74E-12	1.03E-10
FLJ20184	-0.00814179	0.852218696	0.897310404
POLA2	-0.129933959	0.00283049	0.006785452
TMC7	0.001070584	0.980457687	0.985586778
HSD17B6	0.070565128	0.105974424	0.164887837
ZNF658B	-0.189526179	1.21E-05	4.76E-05
TTTY10	0.05302279	0.224739202	0.308911895
RANBP9	0.077225849	0.07679702	0.124960273
CPNE7	0.043176797	0.322980493	0.418745188
EVL	0.332217619	5.10E-15	1.12E-13
LNX1	0.246142494	1.07E-08	7.44E-08
IFNA21	-0.053330816	0.222053842	0.305871209
CFD	0.230719837	8.73E-08	5.14E-07
PYCARD	0.144750061	0.000870175	0.002331462
MYBPC2	-0.16087562	0.000211379	0.000641861
ENPP3	-0.108139236	0.013082324	0.026499145
ACSL4	-0.322994043	3.09E-14	5.83E-13
LOC440258	0.195563961	6.24E-06	2.62E-05
TMEM176B	-0.000710173	0.987035859	0.990903428
SOX2	0.217740428	4.59E-07	2.43E-06
SCO1	-0.016229553	0.710370376	0.782247034
COMT	-0.078684076	0.071372822	0.117188647
AOC2	-0.019709468	0.651990305	0.732688083
PDLIM5	0.046544698	0.286637886	0.379462137
SPHK2	0.137607269	0.001558662	0.003953884
NXPH2	-0.007703434	0.860886986	0.903353759
GPR108	0.142062312	0.001086983	0.002861241
RAD51L1	-0.0468834	0.283141727	0.375466569
TMEM54	0.161579872	0.000198082	0.000604471
LETMD1	0.004172583	0.923941792	0.946914759
SLC6A17	0.062244888	0.153997248	0.225781849
KRT75	-0.079117305	0.070359351	0.11567905
STT3B	0.017737222	0.68484412	0.760676751
CD3EAP	-0.013980973	0.749042554	0.81462021
TMEM63A	0.068459923	0.116831657	0.178839398
DUSP13	0.353287222	6.59E-17	2.09E-15
CD1C	-0.176904275	4.51E-05	0.000157918

LASS2	0.392646333	7.79E-21	4.74E-19
AVP	0.073995988	0.090006689	0.143903051
PITPNM1	-0.026073958	0.550723089	0.641923632
FLJ22795	-0.077882193	0.074315503	0.12133989
MCTP1	-0.065950981	0.13088738	0.196779095
TRIM68	-0.028366027	0.516242105	0.610104306
UCK2	-0.212762454	8.46E-07	4.23E-06
ABHD1	0.181686669	2.77E-05	0.00010033
FAM50A	0.321895613	3.81E-14	7.06E-13
RNASEH1	-0.312019082	2.43E-13	3.80E-12
PCP2	0.418988722	8.93E-24	8.72E-22
OR52H1	-0.02263789	0.604441382	0.689684553
C20orf149	0.483773372	3.29E-32	1.64E-29
RBP5	-0.193451507	7.88E-06	3.22E-05
HYAL3	0.063875976	0.143471542	0.212733665
CLPB	-0.123014348	0.004723315	0.010669972
SMNDC1	-0.298491116	2.76E-12	3.47E-11
DONSON	-0.172010487	7.33E-05	0.000245058
FLJ27523	0.011391801	0.794359089	0.852940352
BARHL2	0.026520077	0.543924258	0.636337569
SLC30A9	-0.031920723	0.465062371	0.561820928
TMPRSS11B	0.002306924	0.957904981	0.969995725
E2F8	-0.023722941	0.587225982	0.67379223
CCDC25	-0.068237933	0.118025547	0.180532112
C14orf48	0.05857273	0.179822001	0.257154569
C20orf116	0.323534909	2.78E-14	5.30E-13
TSPAN11	-0.035633894	0.414747792	0.513238915
YIF1B	0.048796923	0.263932157	0.35348242
FAM12B	0.179026999	3.63E-05	0.000129554
OR1L6	-0.001839039	0.966436931	0.975639581
HPN	0.19464525	6.91E-06	2.87E-05
NBN	0.018106623	0.678642834	0.754879664
C14orf94	0.032656583	0.454830513	0.552281364
OCLM	-0.053340533	0.221969507	0.305823548
ZSCAN18	0.074058676	0.089733973	0.143539032
L3MBTL	0.119520008	0.00606074	0.013338401
TSTA3	0.215222495	6.27E-07	3.20E-06
RAC1	0.151148642	0.000504598	0.00141292
C19orf15	0.058229843	0.182388154	0.260391168
NFE2	0.138704754	0.001427608	0.003660702
KLK14	0.170055935	8.87E-05	0.000291636
ARSF	-0.132489747	0.002328232	0.005687843
MAST2	0.091692567	0.035521039	0.063996153
AMICA1	-0.184332922	2.10E-05	7.83E-05
GTF2A1	0.073685844	0.091365719	0.145635023
ATP1A3	-0.285264221	2.63E-11	2.86E-10
TC2N	-0.131301635	0.002550619	0.006194216
PNKP	0.186250361	1.71E-05	6.51E-05
ODZ2	0.05813525	0.183100823	0.261226673
MATR3	0.182242093	2.61E-05	9.51E-05
S100P	0.162147076	0.000187948	0.000576692

KRT82	-0.024901254	0.568791492	0.65779554
CA13	-0.330250279	7.52E-15	1.59E-13
PROZ	0.117414602	0.007022163	0.01516659
AASDH	0.034559372	0.428967841	0.526807121
C19orf40	-0.080069066	0.066514964	0.110094352
DCK	-0.092335564	0.034245069	0.061915004
FAM5C	-0.072616655	0.096177338	0.152185911
SLC6A4	0.057943841	0.184549212	0.26274441
MID1IP1	0.183881764	2.20E-05	8.16E-05
TESSP5	0.024310981	0.577991726	0.665805756
TMOD4	-0.199187291	4.15E-06	1.81E-05
DOCK2	-0.169685279	9.20E-05	0.000301339
TUG1	-0.109787279	0.011749919	0.024067372
NUP214	0.127014821	0.003523503	0.008244299
DPYSL2	-0.268205805	4.07E-10	3.62E-09
GOLM1	-0.051215147	0.240964788	0.326579784
MPFL	0.101084176	0.020408009	0.039301236
SOX13	0.162343228	0.000184557	0.000567706
SDCCAG8	-0.117002321	0.007225686	0.015546096
KEL	-0.154354611	0.00038091	0.001093473
NUP210L	-0.067997292	0.119330501	0.182301429
GK	-0.07608864	0.081255758	0.131105132
DNAJB1	0.260157788	1.39E-09	1.13E-08
ALPK3	0.043103483	0.323803564	0.419525519
CHID1	0.159104938	0.000248593	0.0007424
CYLC2	-0.083706556	0.055036889	0.093409282
IKZF5	0.049716116	0.255030705	0.34307237
C8orf51	0.172191459	7.20E-05	0.000241136
PPM1J	0.352361151	8.03E-17	2.52E-15
GIMAP8	-0.134455548	0.001998844	0.004936101
GPR101	-0.025886628	0.553590373	0.645068638
NR2F1	0.085879365	0.049002615	0.084687206
ACAD8	-0.044538366	0.307942175	0.402794391
RBM35A	0.196029117	5.92E-06	2.50E-05
GNAI2	0.129380493	0.002951487	0.007048036
METTL8	-0.336757184	2.05E-15	4.84E-14
SLC39A7	-0.009236682	0.832621215	0.881809826
FBXO8	0.13560323	0.001826841	0.004557096
CAMK1	0.052346299	0.230717831	0.315193055
RFC3	-0.21679672	5.16E-07	2.70E-06
FAM129A	-0.064205291	0.141414795	0.209886453
ILF2	-0.267602822	4.46E-10	3.94E-09
FGFBP3	-0.188381017	1.37E-05	5.34E-05
NOM1	-0.051854411	0.235135181	0.320160813
PSMA3	0.070437526	0.106608947	0.165791203
ASCC3	-0.087934948	0.04381365	0.076914552
ZYG11A	-0.065162907	0.135562291	0.202766365
SOX21	-0.012163134	0.780778733	0.842134437
LYRM1	0.048793428	0.263966402	0.35348242
DEFB1	-0.24396609	1.45E-08	9.89E-08
LOC91431	-0.085691599	0.04950123	0.085380943

OR7C2	0.157438147	0.000289146	0.000853557
FAM46B	-0.083756086	0.054892752	0.09324186
TMEM18	-0.086469576	0.047462593	0.082349742
ARHGAP30	-0.239386497	2.73E-08	1.75E-07
TMEM86A	0.079399592	0.068828128	0.113495349
EPHA2	-0.230934049	8.49E-08	5.02E-07
C10orf46	-0.070601079	0.105796191	0.164735573
TCHH	0.006198617	0.887217042	0.920293116
C3orf30	-0.025373757	0.561477523	0.652143074
LOC285636	0.021336166	0.62538709	0.708457114
PAIP2	0.101971567	0.019323901	0.037530848
CYP2U1	-0.110948948	0.01088429	0.022504202
C12orf34	0.177448355	4.27E-05	0.00014982
SARS2	-0.055503588	0.203761015	0.2852747
ZCWPW1	-0.001649094	0.969901762	0.97721218
SAMD12	0.231896983	7.48E-08	4.46E-07
KIAA1430	0.082062669	0.060002192	0.100696911
ACAT1	-0.004104371	0.925181504	0.947209635
MEOX1	-0.092524317	0.033878004	0.061323475
ADAMDEC1	-0.118272444	0.006615061	0.014408288
PHKA2	0.0118135	0.786926522	0.847023012
CARD11	-0.149475792	0.000583077	0.001615019
CALML4	-0.177121805	4.41E-05	0.000154579
TSSC1	-0.000112064	0.997954187	0.998598334
TMEM45A	-0.201717966	3.11E-06	1.39E-05
MPP7	0.360358822	1.42E-17	4.99E-16
POU1F1	0.012203085	0.780077112	0.841672954
SLC2A13	-0.087909435	0.043875101	0.076994291
FBN2	0.089989198	0.03909821	0.069685477
ZC3H7A	0.126946601	0.003541398	0.008276722
LAIR2	-0.161487639	0.000199778	0.000608739
ST3GAL1	0.20093067	3.40E-06	1.50E-05
LCT	-0.336452672	2.18E-15	5.11E-14
GEMIN8	0.182112228	2.65E-05	9.62E-05
KLF16	0.082643191	0.058208035	0.098168186
HIF3A	-0.283015319	3.82E-11	4.01E-10
FAM44A	0.144783015	0.000867784	0.002326729
AQP10	0.070085324	0.108375923	0.168029136
PLA2G2A	-0.157643482	0.000283836	0.000838284
FOLH1	-0.328138253	1.14E-14	2.30E-13
C20orf186	-0.05778718	0.185740937	0.264196396
MAPKAP1	-0.077416021	0.076071182	0.123943216
SPRR2D	0.021240856	0.626932897	0.709815943
UBQLN4	-0.037292132	0.393356779	0.491771087
RSHL1	-0.048469367	0.267155242	0.357116866
PIAS3	0.249388352	6.72E-09	4.90E-08
MRPL24	0.233185455	6.30E-08	3.80E-07
GREB1	0.440907204	1.99E-26	3.21E-24
FAM27E3	-0.128780276	0.003088008	0.007328507
NUP62CL	0.159613107	0.00023733	0.000711527
NEUROG3	0.040241537	0.356995792	0.455334396

REEP3	0.03774964	0.387574386	0.486465585
MARK1	-0.073226797	0.093407367	0.148375587
LMBRD1	-0.102869068	0.018278828	0.03573816
PRPF19	-0.078844031	0.070797375	0.11630592
PNMT	0.018465603	0.672637207	0.750371224
CTGLF1	-0.022329344	0.609377694	0.694157732
SLC25A16	0.093388342	0.032240272	0.0587046
EIF2B3	-0.251572844	4.90E-09	3.66E-08
RPA2	-0.29804338	2.98E-12	3.74E-11
PAK6	0.028425699	0.515359561	0.609295508
CCDC26	0.009965385	0.819634622	0.872413586
SEMA3E	0.029926326	0.493424967	0.587998085
MXD4	0.110150737	0.011472759	0.02353887
TNFSF10	-0.059528783	0.172808364	0.249027099
SMARCB1	-0.15298312	0.00042989	0.001219978
DTX3L	0.058729387	0.178658531	0.255799188
PLA2G4E	0.005281633	0.903813578	0.932161975
PPAP2A	0.066230656	0.129258638	0.194711261
ULK1	0.313801837	1.75E-13	2.84E-12
TAS1R3	0.01125763	0.796727854	0.854987709
SLC2A3	-0.165062125	0.000143094	0.000451688
ARID3A	0.344435326	4.26E-16	1.17E-14
GNG5	-0.002545641	0.953553731	0.967079316
ACOX1	0.044883538	0.304204419	0.39881652
KIF5B	-0.066488026	0.127773702	0.192924518
NUP153	-0.174108123	5.96E-05	0.00020355
MUC7	-0.025042854	0.566594876	0.656128558
CSDE1	-0.35779726	2.49E-17	8.41E-16
CLPTM1	0.178783837	3.73E-05	0.000132192
C3orf23	-0.123980743	0.004403786	0.010044095
LRRC17	0.25958785	1.51E-09	1.22E-08
TTYH3	-0.239898126	2.54E-08	1.64E-07
ATP5B	0.10202149	0.01926443	0.037438995
ELF3	0.117153819	0.007150294	0.015427072
CPSF3L	0.117099814	0.007177087	0.015472911
ZNF665	0.008508765	0.845639444	0.892829145
TLR6	-0.300097938	2.08E-12	2.72E-11
GPI	-0.076920344	0.077974836	0.126541902
RAD9A	0.127723196	0.003342476	0.007859611
NDST4	0.179692811	3.40E-05	0.000121387
AGPAT3	0.123316739	0.004621152	0.010462247
MAGI3	0.2240521	2.07E-07	1.16E-06
ADORA2A	0.048732092	0.264567946	0.354120222
CACNG7	0.13133199	0.002544704	0.006182293
CAMK2D	-0.188353083	1.37E-05	5.35E-05
CCHCR1	-0.089821098	0.039467218	0.070282051
RPS27A	-0.503423227	3.81E-35	3.35E-32
OR10G7	-0.02510657	0.565607783	0.655346195
GCM2	0.013840869	0.751703924	0.816936626
FAM135B	0.154533472	0.000374921	0.001080313
E2F1	-0.024781693	0.570649365	0.659076436

PLCB3	0.017129782	0.695087511	0.7689984
OR2AE1	-0.030267253	0.48851207	0.583500528
COIL	0.124904624	0.004116714	0.009466595
CDC25C	0.125880413	0.003831997	0.008871593
RAB11FIP2	-0.120693532	0.005577781	0.012372935
TSC2	0.24373072	1.49E-08	1.02E-07
CTGLF5	-0.035009199	0.422980804	0.521537791
CCDC108	0.058392694	0.181166025	0.258826646
OR13C4	0.045289394	0.299848121	0.39421982
C10orf81	0.039275317	0.368668024	0.466736602
PTPRB	0.116561053	0.007449352	0.015988155
ACP2	0.071737549	0.100283194	0.157386769
LAG3	-0.139564321	0.001332124	0.00343736
MRPL54	0.171712341	7.55E-05	0.00025137
LOC201175	0.063776098	0.144099829	0.21340796
ITGB1BP3	0.057662717	0.186691769	0.265180801
SPTAN1	0.071137164	0.103166496	0.161271336
SIPA1L2	-0.094315931	0.030557828	0.056010314
RCAN2	-0.094474673	0.030277572	0.055641599
CDX2	0.150114309	0.000551873	0.001534117
ECOP	0.070778825	0.104918469	0.163658972
ACTR1A	0.070845833	0.104589077	0.163227978
PPARG	0.01329733	0.760931295	0.824632804
BBS10	0.060635838	0.164944172	0.239603522
TMEM44	0.144637899	0.000878358	0.002351337
BPIL2	0.413167587	4.20E-23	3.49E-21
CITED1	0.193619073	7.73E-06	3.17E-05
IRF6	0.04452829	0.308051736	0.402852004
PRDM4	0.18591656	1.78E-05	6.73E-05
RRP9	0.096427341	0.027006401	0.050367716
OR10H4	0.048584319	0.266021078	0.355832849
IL31RA	-0.09174597	0.035413547	0.063839901
GNB1L	-0.102809498	0.018346632	0.035847931
MYBL2	-0.034065123	0.435602422	0.533481779
ZNF407	-0.036983992	0.397280528	0.495211426
PPIG	-0.107724663	0.013437789	0.027118138
TTC18	0.158845848	0.000254527	0.000758278
RPSA	-0.210219607	1.15E-06	5.59E-06
MAPT	0.331435444	5.95E-15	1.28E-13
MRE11A	-0.201328712	3.25E-06	1.44E-05
C8orf37	0.031658695	0.468736355	0.565220232
RASGEF1C	-0.329548136	8.64E-15	1.79E-13
STBD1	0.359468067	1.73E-17	6.00E-16
CTAG2	0.035429422	0.417432119	0.516456761
MGAT5B	0.042186246	0.334216034	0.430206069
ECM1	0.366515938	3.62E-18	1.43E-16
RLN1	-0.008089014	0.853165747	0.89800003
PARP14	0.03120814	0.475091031	0.571354342
EPB41L1	0.379828088	1.70E-19	8.22E-18
HOXA3	-0.26487774	6.79E-10	5.80E-09
MAGEA9	-0.031351793	0.473059828	0.569245574

RPS8	-0.405491014	3.09E-22	2.24E-20
RPS19BP1	-0.161158895	0.000205933	0.000626252
FOXJ2	-0.10167604	0.019679231	0.038112627
C10orf76	0.176811689	4.55E-05	0.000159308
IL17RE	-0.088403021	0.04269907	0.075209563
C10orf65	0.309957572	3.55E-13	5.35E-12
ZNF343	0.105710541	0.015288582	0.030443487
FBXO33	-0.026605351	0.542629428	0.635185294
UHMK1	0.13684279	0.001656383	0.004181075
LY6G6C	0.141910661	0.001100589	0.002895816
FGF19	-0.008024555	0.854322712	0.898756263
C14orf128	-0.137426617	0.001581265	0.004007913
IFIT2	0.018595624	0.670467085	0.748357616
TIGD1	-0.192798379	8.46E-06	3.43E-05
S100G	0.258206253	1.99E-09	1.59E-08
GUCY1B3	-0.105154025	0.015837908	0.031395002
NR3C1	-0.127807434	0.00332152	0.007816313
CORO1B	0.222058342	2.67E-07	1.46E-06
PARP11	-0.040586406	0.352886509	0.450747641
DNALI1	0.403371928	5.32E-22	3.59E-20
OR4N4	-0.006990046	0.872932201	0.911317505
MAP2K6	-0.248866454	7.24E-09	5.25E-08
FSTL4	0.146642138	0.00074234	0.002015299
ANKRD47	0.121593154	0.0052313	0.011663257
TMEM171	-0.16956684	9.30E-05	0.000304004
PNLIP	0.03086518	0.479959581	0.575857847
YY1	0.034131134	0.434712915	0.532586115
CCDC138	-0.308570757	4.57E-13	6.72E-12
AASDHPPT	-0.084550657	0.052623073	0.089808291
CKS1B	-0.110167858	0.011459847	0.023520226
MCM3	-0.218096145	4.40E-07	2.33E-06
ANAPC7	0.027992275	0.52178757	0.614767536
FAM110A	0.312092968	2.40E-13	3.77E-12
CDC37L1	-0.153887375	0.00039698	0.001135364
THTPA	0.192590175	8.66E-06	3.50E-05
NBPF20	0.115305311	0.008120112	0.017259097
WDR24	0.356796275	3.09E-17	1.03E-15
NPTX2	-0.077745991	0.074825015	0.122009816
CBLB	-0.028987222	0.507093145	0.601140495
CETN1	0.094542222	0.030158986	0.055456819
RPUSD1	0.207303016	1.62E-06	7.63E-06
FAF1	-0.321648692	3.99E-14	7.32E-13
CDK6	-0.38845696	2.17E-20	1.23E-18
HMX2	0.040076955	0.358967427	0.457498994
CSK	-0.134575199	0.001980244	0.004900008
TEAD2	-0.218906243	3.97E-07	2.12E-06
SNAP25	0.064231328	0.141253149	0.209777461
TUFT1	0.32382575	2.63E-14	5.05E-13
TMTC3	0.231692029	7.68E-08	4.57E-07
LCK	-0.250737337	5.53E-09	4.09E-08
SGOL1	-0.101684489	0.019668993	0.038112627

AKTIP	0.045994121	0.29238287	0.385724581
FURIN	-0.03209457	0.462633651	0.560097326
SOX12	0.25205284	4.58E-09	3.43E-08
DEFB103A	-0.044935706	0.303642128	0.398526243
RAMP1	0.460006937	6.65E-29	1.86E-26
KIR3DX1	-0.032872513	0.455329173	0.552777707
GAS2L3	-0.025280359	0.562919649	0.653262321
PDE8A	-0.038974303	0.3723522	0.470722385
EDN3	-0.165023264	0.00014362	0.000453114
GMIP	-0.014884689	0.733417944	0.801862831
SF3A2	0.077826315	0.074524191	0.121583775
FN3KRP	0.103505508	0.017567966	0.034490876
SMAD7	0.02796656	0.522170227	0.615100523
RHBDD2	0.284632428	2.92E-11	3.15E-10
OR11H6	0.030294471	0.4889531	0.583913888
PPP1R3B	-0.08059249	0.064751238	0.107435338
C9orf23	0.008049193	0.853880458	0.898444719
CADPS	0.022998932	0.598688043	0.68462524
GOLGA8A	-0.187684994	1.47E-05	5.69E-05
TMEM57	0.210170477	1.16E-06	5.61E-06
RGL3	0.234178298	5.52E-08	3.36E-07
S100A14	0.166992254	0.000119156	0.000383408
FGFR2	-0.110696197	0.011067665	0.022821955
XRCC3	0.034637854	0.427919754	0.525794933
RTN4RL2	0.240202487	2.44E-08	1.59E-07
MGC3771	0.27621828	1.15E-10	1.12E-09
GH2	0.020446933	0.639872295	0.72154314
BTBD2	0.102055813	0.019223635	0.037383344
LMO2	-0.051295157	0.240229653	0.325727042
RDBP	-0.029760671	0.495821619	0.590169841
ACRBP	0.069534268	0.11118681	0.171780827
AMY2A	-0.330136174	7.69E-15	1.62E-13
DUOXA1	-0.113316187	0.00929331	0.019463407
PTK7	-0.265868752	5.83E-10	5.05E-09
TWF2	0.007671323	0.860668356	0.903353759
FAM80A	0.175129857	5.39E-05	0.000185513
TNNI2	-0.248054716	8.12E-09	5.82E-08
GLT25D1	-0.14270249	0.00103124	0.002728526
OCC-1	0.000976311	0.982178239	0.986916844
CYC1	0.229299523	1.05E-07	6.11E-07
RPL22	-0.342220662	6.74E-16	1.76E-14
MORN3	-0.044361534	0.309868723	0.404453996
DISP1	0.058842346	0.177823068	0.254820332
PRB2	0.038830841	0.374116041	0.472047018
CHUK	-0.069118169	0.113347068	0.174442048
HR	-0.046691519	0.28511881	0.377762457
CCDC134	0.000509558	0.990697866	0.993120505
DENND4B	-0.159988488	0.000229318	0.000691892
C14orf130	0.065375246	0.134290208	0.201206259
RAB33A	-0.133075656	0.002225215	0.005462213
DCST2	0.053002114	0.224920281	0.309058058

TNMD	-0.016468064	0.706309629	0.779170776
PEX7	0.165008289	0.000143823	0.000453289
FAM62A	0.234457854	5.31E-08	3.25E-07
SRD5A2L	0.183789243	2.22E-05	8.23E-05
IL22	0.058674363	0.179066547	0.256243936
RPS26	0.055071086	0.207312244	0.289193055
HOXC5	0.254063699	3.42E-09	2.61E-08
SPATA6	-0.141855201	0.001105604	0.002906601
FLJ38482	0.316366834	1.08E-13	1.87E-12
ZNF234	-0.006672683	0.878655726	0.914626503
C18orf22	-0.021839258	0.617254909	0.70131198
SPATA22	-0.111164647	0.010729931	0.022222414
THOC1	-0.25630825	2.46E-09	1.93E-08
CYP7B1	-0.283594214	3.47E-11	3.67E-10
KCNC3	-0.091425754	0.036062257	0.064857215
C8orf42	-0.164011997	0.000157947	0.000493754
ALDH1B1	-0.028637617	0.512231651	0.606764095
CCDC100	0.210024937	1.18E-06	5.70E-06
ARMC4	0.127480795	0.003403448	0.007987712
FAM18B2	-0.042496729	0.330667588	0.426531361
SLC44A1	-0.043938438	0.314510412	0.40920959
FBXO17	-0.137710087	0.001545931	0.003924827
C6orf107	0.111740052	0.010327613	0.021446974
C19orf29	-0.040035382	0.35946653	0.457915826
ZC3HAV1L	-0.153175238	0.000422692	0.001201079
PARP6	0.13025932	0.002761487	0.006632961
SULT2A1	0.047331119	0.278564643	0.369955506
C1orf159	-0.02564785	0.557255669	0.648384858
TMC1	-0.067599873	0.121510287	0.184942266
CHST14	0.060293499	0.16734681	0.24257792
GAMT	0.41971483	7.35E-24	7.29E-22
SMCP	-0.084531659	0.052676413	0.08987438
TSPAN33	-0.234958941	4.97E-08	3.05E-07
MIDN	0.206079731	1.88E-06	8.72E-06
NOX4	0.153384657	0.000414974	0.001182976
RNASEN	-0.048435512	0.267489891	0.357408809
TBX1	0.054743579	0.210031009	0.292058044
SALL2	0.083719788	0.054998352	0.093369648
C10orf35	0.018987209	0.663947948	0.742698914
CYP2E1	0.110703401	0.011062401	0.022818752
LRFN2	0.427793111	8.10E-25	9.05E-23
ACO1	-0.035178588	0.420739005	0.519639023
IQCG	-0.278526617	7.94E-11	7.95E-10
MEGF9	0.057026492	0.191608254	0.271099667
TM7SF4	0.081057245	0.063217627	0.105219606
PLEKHA1	-0.159604056	0.000237526	0.000711645
STK33	-0.194907993	6.71E-06	2.80E-05
C1orf210	0.200642511	3.52E-06	1.55E-05
SNUPN	-0.090418656	0.03816872	0.068286138
KIAA0406	0.120109595	0.005813592	0.012849984
C20orf29	0.239591796	2.65E-08	1.70E-07

TMEM55B	0.325687406	1.84E-14	3.60E-13
OSTM1	0.118865676	0.006346044	0.013881829
CLCN7	0.341656789	7.57E-16	1.95E-14
OTP	0.006299362	0.885396526	0.919335231
FLJ23049	0.136777463	0.001664989	0.004197628
HEATR4	0.277504008	9.36E-11	9.25E-10
MAP3K10	0.131753043	0.002463939	0.006000301
PCDHGA9	0.15522712	0.000352513	0.001021009
AMDHD2	0.293673765	6.36E-12	7.68E-11
LCTL	-0.20878504	1.36E-06	6.54E-06
PDCD2L	-0.135458336	0.001847781	0.004603731
CABLES2	0.021328195	0.625516302	0.708472968
SLC5A9	0.019930888	0.648342106	0.730023001
CLCA2	-0.163328767	0.000168374	0.000522367
MGC16025	-0.216982296	5.05E-07	2.65E-06
STRAP	-0.184464211	2.07E-05	7.73E-05
C20orf196	0.05145569	0.238759414	0.324162428
RRBP1	0.053813393	0.217892966	0.301287125
NAT13	-0.238936143	2.90E-08	1.85E-07
MAT2B	-0.089726696	0.039675737	0.070612477
CSNK1D	0.147471735	0.000691974	0.001887708
KIR3DL1	-0.060348818	0.1669568	0.242069644
PRKAG3	-0.186391829	1.82E-05	6.88E-05
ZNF599	0.002762612	0.949600056	0.963949626
PRM3	0.180019096	3.28E-05	0.000117739
PER2	0.133264987	0.002192827	0.005387012
ASPHD1	-0.044281876	0.310739162	0.405332012
PRMT6	-0.129272546	0.002975627	0.007094661
KCNE1L	-0.265702908	5.98E-10	5.17E-09
FAM118A	-0.161749408	0.000195001	0.000596251
TAF4	0.241112583	2.15E-08	1.42E-07
NDUFB6	-0.063849027	0.143640856	0.212933371
TRIM9	0.025152066	0.564903473	0.654653497
PMFBP1	-0.116169387	0.007653041	0.016368191
KY	-0.047517642	0.2780533	0.369435985
DKFZp762E1312	-0.108665069	0.012643406	0.025675133
CSMD1	0.002470066	0.954931137	0.967837739
TBP	-0.101555561	0.019825715	0.038323899
OR1Q1	-0.095598627	0.028355544	0.052460714
RETNLB	0.037730239	0.387818542	0.486672697
HPGD	0.056643007	0.194617169	0.274661687
DNAJC12	0.314024438	1.68E-13	2.75E-12
FKBP1B	0.010104004	0.817169677	0.870392577
ANKRD24	0.110935566	0.010893931	0.022516565
CXXC5	0.601942553	3.67E-53	1.13E-49
IL3	0.047596828	0.275872139	0.366934411
DRAM	0.098602777	0.023724747	0.044901036
PTCH1	-0.356106881	3.59E-17	1.19E-15
TP53BP1	0.136392831	0.001716496	0.004309813
SLC17A7	0.01155731	0.791439685	0.850648947
COL25A1	-0.067147906	0.124026861	0.188167078

AMACR	0.238088304	3.25E-08	2.05E-07
RHCG	-0.28984475	1.22E-11	1.41E-10
VPS13A	-0.04874397	0.264451384	0.354041272
FAM55D	-0.007085225	0.871461048	0.910166567
PRPF38B	-0.183101979	2.39E-05	8.79E-05
OSBPL6	0.06904626	0.113723719	0.174953002
PFDN5	0.183865812	2.20E-05	8.17E-05
CMTM6	0.073428759	0.092504673	0.147131721
KCNK12	-0.070273132	0.107430849	0.16677412
RP2	0.036303949	0.406022833	0.504370527
C16orf52	-0.311893889	2.49E-13	3.88E-12
PICK1	-0.051690108	0.236623908	0.322045243
IFNE1	-0.098986278	0.023183504	0.043998569
SEMA4B	0.215985426	5.71E-07	2.94E-06
TYRO3	-0.147657198	0.000681156	0.001861523
OR12D2	-0.052947615	0.225398075	0.309437991
CSNK1A1	0.308403268	4.71E-13	6.91E-12
FANCF	0.121714267	0.005186166	0.01158304
LONP2	0.246699144	9.85E-09	6.92E-08
TBL1Y	0.097722568	0.025008492	0.046997928
LDOC1L	-0.251452104	4.99E-09	3.72E-08
CCNC	-0.298721923	2.65E-12	3.38E-11
C3orf60	0.359233492	1.82E-17	6.25E-16
CHKA	-0.034355224	0.431701104	0.529212538
UBAP1	-0.088310328	0.042917874	0.075495257
MAP3K1	0.15988425	0.000231517	0.000697842
ANKRD9	0.080654651	0.06454436	0.107149912
FAM92A1	-0.201029134	3.37E-06	1.49E-05
GAB2	-0.031534604	0.470481852	0.566697925
AZU1	0.219519372	3.68E-07	1.96E-06
DIS3	-0.106521068	0.014518456	0.029051085
C21orf109	-0.002862566	0.947779092	0.962897012
IQCB1	-0.244732547	1.30E-08	8.94E-08
SPATS2	0.007941955	0.855805751	0.899700729
EFCAB3	0.103263118	0.017835799	0.034960896
PRB3	0.11204905	0.010117136	0.021045424
FUZ	0.198747359	4.36E-06	1.90E-05
ZNF813	0.026552702	0.543428688	0.635878782
BMPER	0.036320839	0.405804325	0.504201009
HEG1	-0.002229149	0.959322908	0.970848677
ALS2CR11	-0.087832391	0.044061107	0.077232539
SURF2	0.006890013	0.874735568	0.912193797
PSMC1	0.061393135	0.15972137	0.232893749
OR2D2	-0.05305104	0.224491961	0.308676446
SLC7A8	0.288632203	1.50E-11	1.68E-10
C4orf40	-0.135243287	0.001898247	0.004714185
SPATA7	-0.052891037	0.225894857	0.310050776
MAZ	0.154413959	0.000378913	0.001091144
PIN4	-0.096222586	0.027334496	0.050871615
PDE1A	0.025332326	0.562117028	0.652531169
TAF6L	0.01506072	0.730387022	0.798852685

OR2T34	0.10403	0.017000424	0.033462102
KIAA0284	-0.009789322	0.822767958	0.874386481
ACADS	0.11122236	0.010688961	0.022152483
MKRN2	-0.124447555	0.00425655	0.009745268
C18orf56	-0.161946862	0.000191468	0.000586032
MS4A6E	-0.02424873	0.578966054	0.666553504
GALNT4	0.247950576	8.24E-09	5.90E-08
C22orf31	0.004232555	0.922851995	0.946242607
FLJ36070	0.004390581	0.919981086	0.944203465
PSME4	-0.291053537	9.95E-12	1.16E-10
TFG	-0.008398522	0.847614799	0.894147092
EPHX2	-0.038512607	0.37804703	0.476159604
ANXA5	-0.139076583	0.001385557	0.003560296
KRTAP1-1	-0.13006334	0.002802865	0.006727095
BATF	0.211762006	9.54E-07	4.72E-06
KARS	-0.192482845	8.76E-06	3.54E-05
MSTP9	-0.057665393	0.186671287	0.265180801
GPR26	-0.003624366	0.933910202	0.953309848
CCDC72	0.257426971	2.08E-09	1.66E-08
TEF	0.055769738	0.201597747	0.282632136
FOXK1	-0.188298272	1.38E-05	5.37E-05
PRLHR	0.008499208	0.84581064	0.892856588
EMX1	0.292916165	7.24E-12	8.68E-11
C11orf30	-0.01873677	0.668114395	0.746069904
ICK	-0.022140477	0.612408111	0.69644858
THSD7B	-0.098907561	0.023293718	0.044191658
C21orf100	-0.002312878	0.957796436	0.969995725
DUOX1	-0.036033145	0.409535799	0.507811177
EFCAB4B	0.163464783	0.000166248	0.000516032
UBE2G2	0.037144164	0.395238023	0.493365531
C3orf54	0.014720573	0.736247447	0.804118215
PARP1	-0.162272933	0.000185766	0.000571137
FAM60A	-0.238140598	3.23E-08	2.04E-07
C6orf146	-0.006935443	0.874035965	0.911850551
OR9K2	0.095633206	0.028298113	0.05239477
DDX55	0.00917766	0.833675125	0.882318131
RPS15	0.097406104	0.025484479	0.047804777
ZNF618	-0.19385311	7.54E-06	3.10E-05
DKFZp686D0972	-0.092472055	0.033979299	0.061470642
SSPO	0.07430169	0.088683048	0.142105412
SHFM3P1	0.107996654	0.01320363	0.026698166
CPA6	0.265894821	5.81E-10	5.04E-09
JAG2	0.030133012	0.490443404	0.585125434
DEFA3	-0.044511476	0.308234626	0.403005467
PPBPL2	0.009596222	0.826207743	0.876981083
CD34	0.039015113	0.371851394	0.470185939
SLCO4A1	-0.082796787	0.057740837	0.097513981
AFG3L1	0.048305931	0.268773465	0.358734108
SHD	-0.019158925	0.661097148	0.740452889
RP13-122B23.3	0.023877707	0.584789023	0.671497236
PRKCSH	-0.044501622	0.308341841	0.403059945

DPH5	-0.240929749	2.20E-08	1.45E-07
HLA-F	-0.139359099	0.001354371	0.003487449
TBC1D4	-0.338561336	1.42E-15	3.49E-14
RIG	0.045218497	0.300606089	0.39513186
GLUD1	0.119867039	0.00591415	0.013057849
HNRPCL1	-0.077884271	0.074307753	0.12133989
HBXIP	0.041692121	0.339913575	0.435897512
RNF207	-0.096772886	0.026460393	0.049484475
APIP	-0.111028658	0.01082702	0.022408396
PLA2G3	-0.125803775	0.003853696	0.00891847
CCDC84	-0.140036205	0.001282238	0.00332398
MYLIP	0.311543072	2.65E-13	4.11E-12
PHIP	-0.162233056	0.000186455	0.000572682
AARS2	-0.050022564	0.252109876	0.339777543
DHX32	-0.033394928	0.444692419	0.54211215
SCAPER	0.018403528	0.67367423	0.750983111
MEN1	-0.00476027	0.913268952	0.939550073
NIP7	-0.204767031	2.19E-06	1.00E-05
FLJ25404	0.024788907	0.570537194	0.659070675
FASTKD3	0.006422389	0.883174166	0.917802594
TMEM158	-0.202023225	3.00E-06	1.34E-05
RARA	0.397962354	2.09E-21	1.31E-19
BDH1	0.063403483	0.14646237	0.216437662
ANKRD16	-0.062278506	0.153774557	0.225556738
CARM1	-0.028664365	0.511837552	0.606414086
SS18	-0.113543967	0.009151718	0.019206115
IKZF2	-0.004679812	0.914729258	0.940209263
MYD88	0.01647746	0.70614984	0.779170776
PML	-0.161434582	0.00020076	0.000611427
TAF1A	-0.322987445	3.09E-14	5.83E-13
CBFB	-0.210659546	1.09E-06	5.34E-06
HIST1H3H	0.228255782	1.21E-07	6.94E-07
C7orf29	-0.224807146	1.88E-07	1.06E-06
COMMD4	0.315095326	1.38E-13	2.31E-12
DPP3	0.166569558	0.000124052	0.000397704
DAB2	-0.0298431	0.494628293	0.589089556
LOC388882	-0.088149902	0.043298794	0.076069795
YPEL4	-0.070022693	0.108692544	0.168435094
AGBL3	0.075473919	0.083751824	0.134743581
LRP6	-0.317801407	8.28E-14	1.47E-12
SERPINH1	-0.08405586	0.05402707	0.091872913
TLE1	-0.264405317	7.29E-10	6.19E-09
CD244	-0.202769422	2.76E-06	1.24E-05
ZDHHC15	-0.187532196	1.50E-05	5.77E-05
MGLL	0.247848677	8.36E-09	5.97E-08
PLDN	0.044590075	0.307380308	0.402145003
LOC654346	-0.039814471	0.362125968	0.460490643
FAP	0.152356265	0.000454175	0.001284601
GPR37	-0.130973186	0.002615426	0.006329104
SCARA5	-0.105952222	0.015055279	0.030056789
EBF4	0.350328748	1.24E-16	3.73E-15

LSM6	-0.327656852	1.25E-14	2.51E-13
MLLT1	0.009836511	0.82192787	0.873946823
SLC5A12	0.147466657	0.000692272	0.001887708
A2BP1	0.026306813	0.54716919	0.638796914
COPS5	0.095560079	0.028419685	0.052525591
TPM4	-0.031842296	0.466160356	0.562547675
TNFSF4	0.167150696	0.000117368	0.000378249
ACADSB	0.356721993	3.14E-17	1.04E-15
HERPUD1	0.048315902	0.268674549	0.3586984
BCL2L11	-0.132639598	0.002301478	0.005626954
CEP78	-0.171353763	7.82E-05	0.000260064
CDCA3	-0.105720345	0.015279055	0.030434374
WBSCR19	0.055263212	0.205729229	0.287768205
MYO1A	-0.084956927	0.05149313	0.088131654
PPEF1	0.227902747	1.26E-07	7.26E-07
LOC440348	0.073921726	0.090330612	0.144270891
CPEB2	0.289380785	1.32E-11	1.51E-10
BPTF	0.204901854	2.15E-06	9.88E-06
RPL21	-0.304919794	8.84E-13	1.24E-11
GSX2	-0.043587764	0.319318141	0.414739094
ADPRH	-0.193022709	8.26E-06	3.35E-05
C17orf68	0.181217831	2.90E-05	0.000104938
KCNS1	-0.291834632	8.71E-12	1.03E-10
MLLT6	0.011397848	0.794252388	0.852940352
PIWIL4	-0.38687711	3.17E-20	1.71E-18
RNF26	-0.10466635	0.016333435	0.032301498
RAP1B	0.06938681	0.111948628	0.172740807
ADAMTS1	-0.263146525	8.83E-10	7.38E-09
ZNF571	-0.061387905	0.159757006	0.232893749
P2RY6	-0.079513816	0.068428861	0.112988471
TRIM21	0.000606376	0.988930541	0.991995742
CADM3	0.027911203	0.52299447	0.615835502
NLRC5	-0.127844316	0.003312382	0.007800781
ADRA2B	-0.037296234	0.393304696	0.491771087
LOC90835	0.240567508	2.32E-08	1.51E-07
PCF11	-0.053616768	0.219581514	0.303280937
LOC400451	0.364363028	5.86E-18	2.21E-16
GLTSCR1	0.139953015	0.001290905	0.003340816
C17orf88	0.151536205	0.000487875	0.001371716
CDH16	-0.035283479	0.419354303	0.518313489
FGF7	-0.202022087	3.00E-06	1.34E-05
PCSK4	0.314020498	1.68E-13	2.75E-12
NPC1L1	0.363364122	7.32E-18	2.68E-16
TAT	0.038526128	0.377879493	0.476046097
TBCA	0.376014195	4.14E-19	1.89E-17
MGC33407	0.003365097	0.938628257	0.956392537
GPR115	0.215684062	5.92E-07	3.04E-06
CYGB	0.086071267	0.048497365	0.083922338
FNBP4	-0.254400517	3.25E-09	2.50E-08
C12orf43	0.188277565	1.38E-05	5.37E-05
CBL	-0.224478135	1.96E-07	1.10E-06

CLECL1	-0.151343577	0.000496121	0.00139299
PPAPDC1A	0.115843764	0.007826159	0.016686217
WDR25	0.2224716	2.54E-07	1.39E-06
SGCA	0.027060558	0.535743498	0.629160956
C22orf29	-0.010537979	0.809464441	0.864581701
YIPF1	0.040517158	0.353709237	0.451518067
GALK2	-0.02196982	0.615152023	0.699310369
RAB3B	0.038615399	0.376774527	0.47484865
LOC440087	0.189155789	1.26E-05	4.93E-05
UCP1	-0.071145252	0.103127225	0.161271336
REEP5	0.408395077	1.46E-22	1.14E-20
FADD	0.233026607	6.43E-08	3.87E-07
FOXA1	0.435192343	1.02E-25	1.49E-23
CACNA1A	0.097297534	0.025649566	0.048070461
ABI1	-0.225641025	1.69E-07	9.61E-07
GRIN2D	0.093654703	0.031749238	0.057947777
SLC1A4	0.128370858	0.003184399	0.007534001
LOC401127	-0.028395404	0.515807531	0.609707902
HINT2	0.208309688	1.44E-06	6.86E-06
PLD4	-0.031878897	0.465647757	0.562417611
ZNF286A	-0.353581352	6.19E-17	2.00E-15
ENY2	0.031767437	0.467209704	0.56352932
IL1F6	0.07272873	0.095663676	0.151451067
PXDNL	0.007991056	0.854924108	0.898927555
C20orf79	-0.04257094	0.329823054	0.425620558
TNFSF13B	-0.141492959	0.001138881	0.002985071
DENND3	-0.128218555	0.003220948	0.007611688
JARID1D	0.062640726	0.151390575	0.222671739
HIST1H2AK	0.380050421	1.61E-19	7.87E-18
LOC93349	0.031838547	0.466212872	0.562547675
SSH1	0.114628381	0.008503623	0.018036834
ENSA	0.002947457	0.946232747	0.962193676
LOC219854	-0.053350754	0.221880818	0.305769868
CKAP2	-0.163154807	0.00017113	0.000529849
DKFZP564J102	-0.096154938	0.027443643	0.051059292
MGC87315	0.123570109	0.004537092	0.010306087
HNRPAB	0.014656486	0.737353328	0.80418333
AMH	0.121846612	0.005137248	0.01150781
ZNF526	-0.066779757	0.126106552	0.190851388
BRUNOL5	-0.050774925	0.24503783	0.331369609
CACNG3	0.087512772	0.044839857	0.078374156
TRPM1	-0.057751326	0.186014474	0.264524284
PPP2R1A	0.182559798	2.53E-05	9.24E-05
COL2A1	0.003411056	0.937791766	0.956140203
DDN	-0.12622962	0.003734521	0.008658964
FLJ25770	0.001674233	0.969443164	0.977088273
HK2	-0.136713144	0.001673502	0.004215634
ELOVL6	-0.042480016	0.330857977	0.426687438
MDK	0.008064324	0.853608865	0.898312667
EPHX1	0.204973282	2.13E-06	9.80E-06
RASSF2	-0.315194708	1.35E-13	2.27E-12

DKFZP434B0335	-0.034007352	0.436381752	0.534311309
DLX3	0.128047409	0.003262474	0.007700943
PRTN3	0.247685361	8.56E-09	6.08E-08
AVPR1A	-0.021512078	0.622538334	0.70588018
C21orf125	0.061539805	0.158724411	0.231608069
TNFAIP8	-0.229389706	1.04E-07	6.05E-07
GNB2L1	-0.05809026	0.183440506	0.261589905
CALCRL	0.044382592	0.309638882	0.40432565
SCGB2A2	0.163079301	0.00017234	0.000533325
UBXD7	-0.010552668	0.809203945	0.864453623
ZNF674	-0.149665836	0.000587752	0.001626116
TMEM35	-0.076823913	0.078349639	0.127049559
BRSK2	0.065412638	0.134067156	0.200921019
HECTD3	0.061378075	0.159824	0.232936188
TMEM188	0.025779863	0.555227784	0.646364189
LGALS9	-0.068670391	0.115708486	0.177429296
SCARB2	0.065961761	0.130824306	0.196732369
USP34	-0.260516489	1.31E-09	1.07E-08
C17orf28	0.468892439	4.14E-30	1.34E-27
ZDHHC23	-0.088855712	0.041643952	0.073625261
AQP12B	-0.015870193	0.716763316	0.78745357
SLC16A3	0.155716151	0.000337473	0.000981609
APLP2	0.072395048	0.097199488	0.153561275
ITIH2	-0.042780764	0.327442781	0.423348541
MICAL3	-0.336882903	2.00E-15	4.73E-14
TNNI3K	0.018228302	0.676604872	0.753430525
HDAC2	-0.242524155	1.77E-08	1.19E-07
PRR7	0.087359863	0.045216494	0.078920302
THBS2	0.067476379	0.122193925	0.185798823
LOC751071	-0.187494031	1.50E-05	5.78E-05
CA2	-0.091608293	0.035691235	0.064253409
RANBP17	-0.04419214	0.311721657	0.406044683
RLN3	-0.045324789	0.299470181	0.393807131
CRYZ	0.064827324	0.13759161	0.20520272
GBAS	-0.135300046	0.001870909	0.004655695
TAS1R1	-0.156780781	0.000306776	0.000902283
MPZL3	-0.051381704	0.239436225	0.324794474
PCDH8	-0.305998475	7.28E-13	1.04E-11
HSP90B1	-0.034416892	0.430874388	0.528726124
KCNK15	0.54215611	1.60E-41	1.97E-38
TNIP2	0.184814177	2.00E-05	7.49E-05
GPR146	0.017304716	0.692131765	0.766615235
NOL6	-0.02606978	0.550786971	0.641923632
SPC25	-0.047492005	0.276932218	0.36805728
STEAP2	0.029526132	0.499225422	0.593759598
VAMP3	0.085771487	0.04928857	0.085061863
TCIRG1	0.209845261	1.20E-06	5.81E-06
ZP4	-0.256006621	2.57E-09	2.01E-08
PARL	-0.155648544	0.000339516	0.000986618
TRIM39	-0.029705342	0.49662348	0.591007892
KIAA1305	0.001550451	0.971701358	0.978544325

CRNN	-0.039181276	0.369816572	0.467997963
GRN	0.160618464	0.000216439	0.000656577
HSH2D	0.404068282	4.45E-22	3.04E-20
SCAMP1	0.209183546	1.30E-06	6.26E-06
KIAA1913	-0.065067231	0.136138494	0.20343028
PTS	-0.138158108	0.001491563	0.003805653
BANP	-0.162264975	0.000185903	0.000571273
PRKACG	0.054455586	0.212442941	0.294945054
ADCY6	0.316195692	1.12E-13	1.91E-12
C16orf46	0.03145591	0.472014323	0.568321142
CYP51A1	0.217318845	4.84E-07	2.55E-06
DDC	-0.229462571	1.03E-07	6.00E-07
ANPEP	0.0564667	0.196012058	0.276313192
PROM1	-0.430066958	4.31E-25	5.44E-23
SIGLEC10	-0.129170043	0.002998716	0.007144171
COPG	0.169570047	9.30E-05	0.000304004
FAM26E	0.083837844	0.054655517	0.09286454
TRIP4	0.238120637	3.24E-08	2.05E-07
SNX3	-0.170946062	8.14E-05	0.000268881
C1orf175	0.021152796	0.628362571	0.711041857
PPY2	-0.006378505	0.883966789	0.918315896
C14orf152	0.030207367	0.489373144	0.584302032
FTSJ1	0.031116732	0.476385984	0.572575727
DST	-0.109423712	0.01203307	0.02454922
LOC554235	0.078200539	0.073135642	0.119795168
GLRX5	-0.039965045	0.360311953	0.458707702
C20orf12	0.151262271	0.000499641	0.001401592
CAB39	-0.061994829	0.155661386	0.227570581
MSH2	-0.346598278	2.72E-16	7.81E-15
PIP4K2C	0.344265949	4.42E-16	1.20E-14
CYLD	-0.043462043	0.319791108	0.415201758
WTAP	-0.284385766	3.04E-11	3.27E-10
MGAT4A	-0.019529627	0.654959551	0.735047061
TSC22D4	-0.023989537	0.583031059	0.670354896
CHRM2	-0.307965067	5.10E-13	7.45E-12
PPYR1	-0.062224523	0.15413227	0.225820449
CCNH	0.213121342	8.10E-07	4.07E-06
RRM1	-0.148926482	0.000611225	0.001686903
ECAT8	-0.076717433	0.078765193	0.12755522
LOC400120	-0.051909662	0.23463605	0.319693568
GABRA4	-0.039786644	0.362461842	0.460681659
C14orf4	0.242674131	1.73E-08	1.17E-07
C1orf59	-0.259692974	1.49E-09	1.20E-08
CTDSPL	-0.094692798	0.029896071	0.055039204
NHEDC2	-0.207446439	1.60E-06	7.52E-06
PDE11A	-0.006023702	0.890379262	0.921718856
KLHL29	-0.245736018	1.13E-08	7.85E-08
CD5	-0.182181577	2.63E-05	9.57E-05
TSPAN9	0.027768153	0.525127523	0.617873926
WDR67	0.018219214	0.676757	0.753463478
THUMPD1	0.095014587	0.029340787	0.054114128

C18orf17	0.010403578	0.81184881	0.866641797
CLYBL	-0.280461305	5.80E-11	5.91E-10
FLJ13231	0.035695024	0.413947243	0.512454519
CMBL	0.432007138	2.50E-25	3.42E-23
LECT2	0.024499335	0.575048394	0.663285045
NKAPL	-0.05450172	0.212055229	0.294564846
LOC654780	-0.126295204	0.003716467	0.008634601
OR4C6	-0.07119726	0.103204213	0.161271336
RAB30	0.24106753	2.16E-08	1.43E-07
TSSK4	0.043026435	0.324670009	0.420366342
TMEM163	-0.028478099	0.514585219	0.608686865
OSBPL11	-0.169919127	8.99E-05	0.000295059
GNB5	-0.073362717	0.092799082	0.147561819
CCL21	-0.134716722	0.001958448	0.004851933
C1orf121	-0.335844439	2.47E-15	5.71E-14
FMO2	-0.231047272	8.37E-08	4.96E-07
RPTN	-0.014772779	0.738531914	0.805040372
MSTN	-0.013011277	0.766139923	0.829158262
VCL	0.007628151	0.861444555	0.903620363
FYTTD1	-0.130825489	0.002645055	0.006383218
C11orf1	-0.101911043	0.019396213	0.037659399
CCDC88C	0.165661225	0.000135219	0.000429475
HFE	0.032062103	0.463086694	0.560535449
MOGAT1	-0.212074312	9.19E-07	4.57E-06
FAM125B	-0.124251462	0.004317848	0.009862722
IRGQ	0.067758551	0.120636265	0.183885076
RAVER2	-0.159799379	0.000233322	0.000701221
AKAP5	0.052996232	0.224971815	0.309059806
SSSCA1	0.092923549	0.033112662	0.060150593
C11orf63	0.052952626	0.225354113	0.309437991
ACTG2	-0.150222578	0.000546738	0.001521908
PORCN	0.105372385	0.015620339	0.031043782
DTL	0.027022924	0.536311117	0.629587067
TMEM151	0.178993172	3.65E-05	0.000129927
FAM122C	-0.172666982	6.87E-05	0.000231253
RSAD2	-0.00410945	0.925089188	0.947209635
BAT4	-0.022794754	0.601938652	0.687594422
KRTDAP	-0.105290944	0.015701176	0.031174211
MYH8	0.131389611	0.00253351	0.006157532
CRTC3	-0.036293945	0.406152287	0.504370527
LRRFIP2	0.036685132	0.401108498	0.499577913
INTS4	-0.097430183	0.025447989	0.047750896
TTN	-0.165253985	0.000140527	0.000444267
SLC26A5	0.011137594	0.798848631	0.85591919
PLLP	0.065225087	0.135188826	0.202355426
RGS6	-0.127207115	0.003473502	0.008139696
SRGAP3	0.052211668	0.231920983	0.316501441
ZNF525	-0.032269306	0.460199627	0.557479808
NBR2	0.110216489	0.011423241	0.023468597
C13orf1	0.230798461	8.64E-08	5.10E-07
ZNF137	0.11132812	0.010614242	0.022012471

CEP27	-0.058507775	0.180306057	0.257717793
BEST2	0.055219585	0.206087922	0.288073343
RNF121	-0.138324277	0.001471849	0.003765229
DMRTC2	0.079765277	0.06755658	0.11172819
C8orf76	0.117600851	0.006931916	0.015013862
BCCIP	-0.121704833	0.005189669	0.01158304
MEST	0.037092872	0.395891407	0.493880354
HTRA2	-0.128554199	0.003140901	0.007439677
ANGPTL2	0.061077183	0.161885005	0.235604945
ILKAP	-0.041823759	0.338389678	0.434591602
ERAS	-0.00916561	0.83389032	0.882394008
HBS1L	-0.130924394	0.00262518	0.006345217
CPA5	-0.012586736	0.773348619	0.835351913
TMEM30A	-0.239607452	2.64E-08	1.70E-07
CD300LF	-0.10404546	0.016983941	0.033440363
WISP3	-0.232254102	7.55E-08	4.49E-07
CRK	-0.017163535	0.694516849	0.768505327
PDS5A	0.163315928	0.000168576	0.00052273
BRPF3	0.1445206	0.000886991	0.002372383
NEDD9	-0.016634465	0.703481519	0.776468832
SMPDL3B	-0.175255801	5.32E-05	0.0001834
PSG6	0.162345016	0.000184527	0.000567706
PSMD13	0.082565835	0.058444511	0.098512966
ETV5	-0.369074751	2.03E-18	8.39E-17
OR51A4	0.047094581	0.281436448	0.373366282
BTBD7	-0.024961485	0.567856649	0.657085158
GSTO1	0.01022516	0.815016754	0.868711156
hCG_16001	-0.010217647	0.815150208	0.868711156
MAD2L1BP	0.130374723	0.002737382	0.00658793
COX6A2	0.082085364	0.059931201	0.100632703
SCNN1A	0.345487325	3.43E-16	9.62E-15
LSM1	0.070594826	0.105827172	0.164742096
UGT2B11	-0.016451271	0.706595265	0.77934606
IDUA	0.306112873	7.13E-13	1.02E-11
PPP2R3C	-0.067831452	0.12023637	0.18354852
COX11	0.077911834	0.074204999	0.121255875
PDZK1	0.30341885	1.16E-12	1.59E-11
ZNF443	0.083658856	0.055175997	0.093619537
MGC21874	0.317249362	9.19E-14	1.61E-12
ZNF323	-0.044698026	0.306209521	0.400869139
KRTAP10-10	0.139121431	0.001380563	0.00355043
CXCL6	-0.239943702	2.53E-08	1.64E-07
SLC34A2	-0.279668409	6.60E-11	6.69E-10
LOC284402	-0.10886525	0.01247976	0.025384733
NPTN	0.23873327	2.98E-08	1.90E-07
UPP1	-0.21790936	4.50E-07	2.38E-06
SLC6A9	-0.081999901	0.060198892	0.100999451
OR7G3	0.010318739	0.813354808	0.867380977
CISD1	-0.108964353	0.012399439	0.0252297
ZNF545	-0.305734437	7.63E-13	1.08E-11
SYT14	-0.086122435	0.048363387	0.083747246

NT5C3L	-0.060665782	0.164735254	0.239526383
ZNHIT3	0.081293586	0.062449296	0.104149911
SNRPD3	-0.015346993	0.72546694	0.794602104
KIAA0701	0.070608805	0.105757916	0.164717686
UNC93B1	0.207262905	1.63E-06	7.64E-06
GMNN	-0.19305924	8.23E-06	3.34E-05
SPCS2	-0.017617089	0.686865451	0.762511328
LOC388524	-0.163661746	0.000163214	0.000507933
NAPRT1	0.217148808	4.94E-07	2.60E-06
PNLIPRP1	-0.088391723	0.042725687	0.075234894
OR6V1	-0.146323796	0.000762553	0.002064188
PRKAB1	0.228220446	1.21E-07	6.97E-07
EYA4	0.082817888	0.057676899	0.097432762
KIF20A	-0.007086785	0.871188858	0.910166567
ALG10	0.041332776	0.344095783	0.440709221
ITPKC	0.166737587	0.000122084	0.000392006
LMX1B	0.362173528	9.52E-18	3.40E-16
RPUSD4	-0.260882917	1.24E-09	1.02E-08
C7orf34	-0.12467379	0.004186807	0.009609809
DLGAP2	-0.050613822	0.246540358	0.333181684
PFN1	0.007790148	0.858532694	0.901675689
MICALL2	0.346202265	2.95E-16	8.44E-15
ZNF654	0.102028382	0.019256232	0.037434894
SS18L1	0.0278589	0.523773854	0.616635158
SLC16A8	-0.231995995	7.38E-08	4.41E-07
MKI67IP	-0.227007933	1.42E-07	8.11E-07
ITGB3	-0.04307339	0.32414179	0.419875262
TCEA3	0.228843856	1.12E-07	6.47E-07
CEP152	-0.032604584	0.455549361	0.552935851
CLIP1	0.1951336	6.54E-06	2.74E-05
ZNF75	0.14099849	0.001185795	0.003096159
ATP5C1	-0.130856509	0.002638806	0.006373142
NUDT5	-0.208852078	1.35E-06	6.49E-06
PSCDBP	-0.265342624	6.32E-10	5.43E-09
UBP1	-0.067275721	0.123311106	0.187219751
RBM27	0.072196442	0.098122886	0.154746762
C13orf15	-0.164867842	0.000145739	0.000459094
ZNF282	0.064946653	0.136867353	0.204320794
ZNF222	-0.155992768	0.000329233	0.000961278
COL10A1	0.165802846	0.000133418	0.000424963
PRDM15	-0.175042773	5.43E-05	0.000187028
TTTY5	-0.026992022	0.536777426	0.6300142
FAM9C	-0.031550316	0.470260642	0.566653476
C20orf67	0.191673254	9.57E-06	3.83E-05
GNG13	0.170626498	8.40E-05	0.000276346
F12	0.056913807	0.192488847	0.272095153
C1orf41	-0.23350061	6.04E-08	3.66E-07
CPXCR1	-0.08440028	0.054426833	0.092501546
GSK3A	0.06734319	0.122934583	0.186740304
SUPT6H	0.22484714	1.87E-07	1.06E-06
PI16	-0.047351324	0.278359278	0.369762627

ELL2	0.018798868	0.667080341	0.745653796
C9orf167	-0.171787692	7.49E-05	0.000249948
PVRL3	-0.081591776	0.061490905	0.102746624
FLJ38596	-0.000212327	0.996123845	0.997259122
ADAM20	0.100184087	0.02156129	0.041302297
GPR89A	-0.0596516	0.171922366	0.248215691
GPR87	0.002080062	0.962041303	0.972959206
ZNF30	0.053679836	0.219038892	0.302667449
SMR3B	-0.087883046	0.043938738	0.077061979
ZNF770	-0.000103467	0.998111133	0.998598334
TRPC4AP	0.19418621	7.26E-06	3.00E-05
DKFZP686E2158	0.218755909	4.05E-07	2.15E-06
C2orf28	0.063148885	0.148093542	0.218428205
FREM1	-0.084707058	0.052185658	0.08911125
LAMA4	0.017461608	0.689484827	0.764864189
ADPRHL2	-0.091033945	0.036869723	0.066136959
EIF4G2	0.052106427	0.232864578	0.317561387
GUCA1A	-0.00397843	0.927539975	0.948834355
CTNNA2	-0.021640367	0.620464356	0.704233186
NUDT15	-0.185281622	1.90E-05	7.17E-05
CEPT1	-0.083061394	0.056943269	0.096272797
ZNFX1	0.238938027	2.90E-08	1.85E-07
CCDC92	-0.039805298	0.362236663	0.460490643
TDRD1	-0.064975607	0.136692061	0.204108665
KCNK5	-0.311243533	2.80E-13	4.32E-12
ETNK1	-0.094020023	0.031086181	0.056889561
LTA	-0.12153054	0.005254772	0.011702859
TTPA	-0.081100205	0.063077391	0.105026503
B3GALNT2	-0.150393036	0.000538744	0.001502376
SC65	0.17879211	3.72E-05	0.000132192
PEX5L	0.078523297	0.071955077	0.118050098
EPS15L1	0.329670648	8.43E-15	1.76E-13
MGEA5	0.095155403	0.029100589	0.053687225
HIST1H3A	0.003767366	0.931308928	0.951423592
ING1	-0.122020091	0.005073756	0.011373869
BCAT1	-0.193815557	7.57E-06	3.11E-05
ORC6L	-0.083266384	0.056331697	0.095317448
KLK11	-0.178059827	4.01E-05	0.000141552
C19orf28	-0.012756587	0.77037515	0.832665987
DNER	-0.201435983	3.21E-06	1.43E-05
MED22	-0.111970298	0.010170414	0.021141946
ETV6	-0.474190369	7.61E-31	2.60E-28
CHAC2	-0.451313361	9.31E-28	1.97E-25
CD300E	-0.015491468	0.722988159	0.792593009
CEBPB	-0.249380706	6.72E-09	4.90E-08
ZNF398	-0.015510235	0.722666382	0.792381523
LRCH3	-0.136846903	0.001655842	0.004181075
HMGA1	-0.176324572	4.78E-05	0.000166646
CAPN7	-0.015359223	0.725256992	0.794513673
MGC5566	0.00640602	0.883469814	0.917954696
CCL3	-0.096392734	0.027061614	0.050455387

NANOS1	-0.216273461	5.51E-07	2.85E-06
ZFYVE19	0.278825432	7.56E-11	7.62E-10
APITD1	0.034899182	0.424440569	0.523013963
PARD3	0.007369449	0.866098527	0.907000858
IRAK4	0.1125747	0.009767825	0.020387765
SERPINI2	0.044393755	0.309517088	0.404252459
CEP170L	-0.292501218	7.77E-12	9.26E-11
TTC9	0.244736058	1.30E-08	8.94E-08
MYOM3	-0.004245275	0.922620879	0.946163407
MLPH	0.484676992	2.43E-32	1.36E-29
LOC222699	0.15779105	0.000280076	0.000829171
NRG1	-0.223233765	2.30E-07	1.27E-06
TBC1D9	0.41627518	1.85E-23	1.62E-21
TTK	-0.227244321	1.38E-07	7.87E-07
ZNF557	-0.058037673	0.183838133	0.262035392
DDX41	0.348244027	1.92E-16	5.66E-15
FANK1	0.164250568	0.000154451	0.000484025
UBE2D2	0.198194053	4.64E-06	2.00E-05
PSMB10	0.054938184	0.208412433	0.290201099
MYH7B	0.034364837	0.431572176	0.529198642
GABARAPL2	0.105208391	0.015783492	0.031317423
MARVELD2	0.444241703	7.53E-27	1.36E-24
DGCR2	-0.042260555	0.333364546	0.429289757
UNC45A	0.195055927	6.60E-06	2.76E-05
C6orf72	-0.021165576	0.628154991	0.710937795
ZNF683	-0.087254764	0.045476908	0.07930729
GIT2	-0.088722765	0.041951513	0.074083818
CASK	-0.007172228	0.869649594	0.909599866
C14orf161	-0.055958492	0.200073712	0.281072253
LRRC44	0.163212496	0.000170212	0.00052727
TIFA	-0.33847312	1.45E-15	3.54E-14
UTP11L	-0.410723256	7.98E-23	6.54E-21
C6orf65	-0.156473504	0.000315357	0.000924717
FDPS	-0.071161734	0.103047224	0.161230885
DUSP9	-0.083402213	0.055929472	0.094741135
SLC17A8	0.076725525	0.078733551	0.127537567
OR51G1	0.074053685	0.089755664	0.143539032
NANS	0.17527034	5.31E-05	0.000183237
OLFML1	0.133305617	0.002185933	0.005372222
ATP10B	-0.154058239	0.000391032	0.001120436
NPAS3	-0.201747093	3.10E-06	1.38E-05
PRKCA	-0.271696625	2.36E-10	2.17E-09
GGA2	0.10878367	0.012546224	0.025494624
LCE4A	0.044891897	0.304114274	0.39881652
SPANXN3	-0.006126046	0.888528818	0.920984352
CCDC115	-0.009004938	0.836760992	0.884974774
SDCCAG3	-0.056376147	0.196731325	0.277136521
GLIPR1L1	-0.051552628	0.237874694	0.323246738
TTC1	0.117520412	0.006970765	0.015082067
C17orf76	0.04054089	0.353427142	0.451344444
MAD2L2	-0.150072855	0.000553851	0.00153892

HIPK1	0.132687435	0.002292997	0.005616454
LRRC3B	-0.066831598	0.125812069	0.190546407
CLN3	0.151724544	0.000479936	0.001351248
C17orf47	0.011389264	0.79440387	0.852940352
FMN2	-0.007177439	0.869555716	0.909599866
TUBB1	0.061399931	0.159675068	0.232884723
WAPAL	-0.118870809	0.006343759	0.013881771
C3orf21	-0.016676677	0.70276473	0.775816934
SCN5A	-0.10682954	0.014234446	0.028529208
SMYD1	-0.177400211	4.29E-05	0.000150465
BEX5	-0.137744073	0.001541744	0.003915814
ZNF192	-0.169303703	9.69E-05	0.00031584
SEC22A	-0.108362197	0.012894602	0.026159322
GRIA2	0.088676585	0.042058793	0.074251944
KIAA0825	0.24429779	1.38E-08	9.47E-08
NUSAP1	-0.020005067	0.647121781	0.729049438
LANCL1	-0.08837769	0.042758771	0.075271595
C15orf40	-0.072895186	0.094904817	0.150365814
ZNF645	-0.028110314	0.520032895	0.613287739
GPR61	-0.062372152	0.153155505	0.224815755
NLRP14	-0.095658665	0.028255893	0.052363397
SNX21	0.165854797	0.000132763	0.000423423
C1QTNF8	-0.029967685	0.492827567	0.587513902
C17orf46	-0.007459802	0.864472565	0.906332788
IFNA8	-0.002495724	0.954463489	0.967682769
SPRR1B	0.031119538	0.476346199	0.572575727
FLRT1	-0.1143005	0.008695124	0.018398596
SNX17	-0.022209021	0.611307536	0.695711649
ASB2	-0.293228889	6.86E-12	8.24E-11
HBG1	0.084955406	0.051497322	0.088131654
RPRML	-0.298505957	2.75E-12	3.47E-11
JOSD2	0.316263008	1.11E-13	1.90E-12
PLSCR3	-0.194752836	6.82E-06	2.84E-05
SPOCD1	0.248976413	7.12E-09	5.17E-08
RAB39	-0.117460584	0.006999786	0.015128886
GHRH	-0.004391277	0.919968459	0.944203465
ITIH5L	0.165405719	0.000138527	0.000439073
C17orf37	0.222728409	2.46E-07	1.35E-06
SMCR8	0.229542372	1.02E-07	5.94E-07
DPY19L2P3	0.145311807	0.000830243	0.002233231
IL11RA	-0.123134953	0.004682327	0.010592946
GDF3	-0.007171665	0.869659719	0.909599866
RPS6KB1	0.274393883	1.54E-10	1.46E-09
DNAJC19	0.047871644	0.273106023	0.363512983
TOP1	0.113399852	0.009241077	0.019367207
CRCT1	-0.03359782	0.441929224	0.539706613
MPST	0.02940641	0.500967682	0.595256092
DPM2	0.233367898	6.15E-08	3.72E-07
FAM38B	0.092622005	0.033689353	0.061035897
SLC18A1	-0.049459969	0.25749004	0.346077871
FARP1	-0.184850864	1.99E-05	7.48E-05

PAX7	-0.077094365	0.077302147	0.125649195
TUBD1	0.104706345	0.016292291	0.032243739
GNL3	-0.085491432	0.050037442	0.08599224
BTG2	0.171277713	7.88E-05	0.000261722
NDUFS6	0.135071893	0.001904712	0.004728331
C1orf79	-0.247670629	8.58E-09	6.08E-08
ERAL1	0.191883512	9.36E-06	3.75E-05
ECHS1	0.166072024	0.000130057	0.000415654
VPS4A	0.151304765	0.000497798	0.001397061
CYP11A1	-0.118357243	0.00657599	0.014338924
ABCC6	0.050434277	0.248222467	0.335086706
PBX4	-0.174850408	5.54E-05	0.000190437
MOSC1	0.053529899	0.220330491	0.304042233
NCF4	-0.140385631	0.001246412	0.003237934
HYMAI	0.008778331	0.841414586	0.888891274
NAGPA	0.134975837	0.00191911	0.004760229
OTOP2	0.075769151	0.08254542	0.133011475
ACOT12	0.063620115	0.145085258	0.214665412
MTHFD2L	-0.087648331	0.044508167	0.077879365
LOC441376	0.007438947	0.864847806	0.906417106
C19orf34	-0.087422087	0.045062908	0.07871371
RAB1B	0.216281801	5.50E-07	2.85E-06
ALDOAP2	0.374261224	6.21E-19	2.79E-17
NTRK1	0.002625058	0.952106436	0.965930123
ARTS-1	-0.035721304	0.413603367	0.512131918
SLC6A11	-0.193595979	7.91E-06	3.23E-05
NAP1L2	-0.008322662	0.848974641	0.895120896
CNGB1	0.175738521	5.07E-05	0.000176138
EPB41L4B	-0.196557815	5.58E-06	2.37E-05
FAM134B	0.345192143	3.64E-16	1.01E-14
HS3ST3A1	-0.028035836	0.521139673	0.614357334
CPXM2	-0.067036453	0.124653626	0.189071324
SIRPB2	0.077045363	0.077774819	0.126317317
CHORDC1	-0.172497018	6.99E-05	0.000234879
TRIB3	0.309696276	3.72E-13	5.58E-12
SLC2A5	-0.19193531	9.30E-06	3.74E-05
C2orf49	-0.066276583	0.128992676	0.194403665
DDX5	0.13190249	0.00243584	0.005936575
OR5L1	0.054116477	0.215308454	0.298249985
ANAPC4	-0.079370465	0.068930244	0.113633263
ZSWIM1	0.247714675	8.53E-09	6.07E-08
LOC93622	0.313186914	1.96E-13	3.13E-12
KCNK3	0.034362486	0.431603706	0.529198642
RP11-35N6.1	-0.152981819	0.000429939	0.001219978
ZFP161	-0.22827368	1.20E-07	6.94E-07
AQP9	-0.085493497	0.050031888	0.08599224
SLC15A2	-0.252700365	4.17E-09	3.14E-08
MREG	0.333957066	3.60E-15	8.15E-14
OR9I1	-0.006199003	0.887210063	0.920293116
PDLIM2	-0.074578872	0.087496455	0.140364127
ADAM7	-0.037117179	0.39558169	0.493594118

GSTCD	0.22178146	2.77E-07	1.50E-06
WDR21A	-0.161683118	0.0001962	0.00059962
SLC12A8	-0.242355746	1.81E-08	1.21E-07
TMEM174	-0.025603682	0.557934937	0.648778731
IGSF3	-0.150269667	0.000544519	0.001516416
LRRN1	-0.342133498	6.86E-16	1.79E-14
LOC402117	0.016786202	0.700906172	0.774321245
SRPK1	-0.40442471	4.07E-22	2.81E-20
LY6K	-0.160458524	0.000219643	0.00066564
NFIA	-0.006493832	0.881884053	0.916917405
PTCD3	-0.187296458	1.53E-05	5.88E-05
LEP	-0.034633892	0.42797262	0.525794933
PCDH21	-0.209823169	1.20E-06	5.82E-06
MAPKAPK2	0.263587934	8.26E-10	6.96E-09
NMNAT1	0.00624971	0.886293683	0.919645605
LHFPL2	-0.054916689	0.208590768	0.290383661
C9orf43	0.099832441	0.022026989	0.042076408
DIP2A	0.017876447	0.682504373	0.758626065
ACTR8	0.018909286	0.665243225	0.743877176
CCDC34	-0.075761671	0.08257581	0.133025585
PTPN22	-0.226512262	1.51E-07	8.61E-07
ITGA3	0.259291323	1.58E-09	1.27E-08
FAM129C	-0.167505798	0.000113453	0.00036659
RABGGTA	0.243059507	1.64E-08	1.11E-07
UNC45B	-0.059000326	0.176659497	0.253507409
KIAA1033	0.023902483	0.584399342	0.671416846
ZNF510	-0.118505318	0.006508262	0.014206355
CYP2D6	-0.095594775	0.028361947	0.052460714
SLC26A10	-0.092626259	0.033681157	0.061035897
STX8	0.031063682	0.477138402	0.573255966
LUZP1	-0.203414496	2.56E-06	1.16E-05
WDR89	0.070935628	0.104148961	0.162664964
EIF4G3	0.055213441	0.206138473	0.288078516
C5AR1	0.008572783	0.844492789	0.891771623
ZNF623	0.137286429	0.001599011	0.004046221
A2M	-0.136191078	0.001744093	0.004373748
TGM7	0.087933493	0.043817153	0.076914552
GRPEL1	0.265372652	6.29E-10	5.41E-09
LMNB2	-0.237692608	3.43E-08	2.16E-07
ROCK2	-0.109176172	0.012229293	0.024899975
SNX16	-0.068104988	0.118745097	0.181452187
CCDC66	0.025556486	0.558661232	0.649500456
ANXA3	-0.24728424	9.06E-09	6.41E-08
KIAA1609	-0.091245652	0.036431537	0.065463916
EED	-0.328723816	1.02E-14	2.07E-13
RNF32	-0.063811772	0.143875179	0.213177946
HES1	0.096485834	0.026913298	0.050224543
CLC	-0.076975612	0.077760681	0.126317317
ISL1	-0.102068129	0.019209016	0.037366732
KIAA0528	-0.034863524	0.424914326	0.523253485
MANEA	-0.139968829	0.001289254	0.003337946

C1orf61	-0.222155827	2.64E-07	1.45E-06
hCG_2001000	-0.008748799	0.841341791	0.888891274
RAPGEF6	0.317043029	9.55E-14	1.66E-12
KIAA0020	-0.40531838	3.23E-22	2.31E-20
NEIL1	0.170366681	8.61E-05	0.000283268
C16orf45	0.237076941	3.73E-08	2.34E-07
RBM10	0.095392741	0.02869957	0.052995091
C10orf125	-0.205716916	1.96E-06	9.06E-06
MRS2L	-0.076184128	0.080873492	0.1305226
DNAH17	-0.100774739	0.020798289	0.039977705
C19orf10	0.116484238	0.007488914	0.016061852
C1orf160	0.156486716	0.000314984	0.000924063
SLFN12	-0.205020646	2.12E-06	9.76E-06
EXOC3	0.157203379	0.000295331	0.000870143
HIST3H3	0.136508116	0.001700906	0.004275909
NCOR2	0.156334995	0.000319298	0.000935691
TNFRSF9	-0.173530116	6.31E-05	0.000214005
MFSD8	0.070881945	0.104411905	0.162992841
ALX1	-0.274407632	1.54E-10	1.46E-09
NOL1	-0.182460396	2.55E-05	9.31E-05
PODN	0.137261941	0.00160213	0.004052445
TIAL1	-0.094577447	0.030097302	0.055376514
HIST1H1E	0.257173538	2.16E-09	1.72E-08
NPY6R	0.017453723	0.689617767	0.764873674
TM4SF4	-0.032814417	0.452652462	0.549853811
CORO2A	0.199680835	3.93E-06	1.72E-05
ETNK2	0.219236818	3.81E-07	2.03E-06
APOE	0.059035037	0.176404601	0.253200721
ANGPT4	0.034716988	0.426864463	0.525065545
HDGF2	0.017931072	0.681587212	0.757743584
G30	0.013514751	0.759629595	0.823512408
ST8SIA4	-0.145982983	0.000784757	0.002121086
F2RL1	-0.004614017	0.915923645	0.940691584
FAM19A4	-0.248131685	8.03E-09	5.77E-08
CCAR1	-0.002704566	0.950657658	0.964619462
B3GNT7	-0.123580758	0.004533589	0.010301937
OPHN1	0.037953012	0.385020681	0.483951792
DSCR6	0.174426786	5.78E-05	0.000197645
C21orf13	0.302056064	1.47E-12	1.99E-11
GAS2L1	0.014656973	0.737344914	0.80418333
RFX3	-0.338444179	1.46E-15	3.55E-14
COPS4	0.013057083	0.765122821	0.82865096
BCHE	-0.059483576	0.173135341	0.249381403
BCL2	0.142124977	0.001081407	0.00284892
HBZ	0.05354064	0.220237782	0.303982518
ARL13B	-0.063786572	0.144033847	0.21336163
MAPBPIP	0.267256188	4.71E-10	4.15E-09
MYO15B	0.152245662	0.00045859	0.001295136
SPZ1	0.001900118	0.965322907	0.974674968
KIAA1324	0.232883148	6.56E-08	3.94E-07
PLCL2	-0.248679416	7.43E-09	5.37E-08

C4orf29	0.033825714	0.438837238	0.536708393
WDFY2	-0.297556491	3.25E-12	4.05E-11
ZNF284	-0.182772831	2.47E-05	9.06E-05
NAALADL1	-0.026782523	0.539944125	0.632644135
DUSP5	0.284103752	3.19E-11	3.40E-10
PXDN	-0.044340256	0.310101079	0.404671378
SLMO1	-0.082743645	0.057902127	0.097759522
TNXB	0.045344474	0.299260123	0.39361508
BIRC7	0.040168736	0.357867086	0.456351039
A4GALT	0.277461907	9.42E-11	9.30E-10
TIMM22	0.098793207	0.023454638	0.044444551
FAM110C	0.287331412	1.86E-11	2.07E-10
TOMM34	0.029561859	0.498706131	0.593256722
ABHD9	-0.172937709	6.69E-05	0.000225535
ADAM32	-0.172248763	7.16E-05	0.000240044
CRHBP	-0.00986187	0.82147651	0.873763919
AQP2	0.003849648	0.929812479	0.950210559
LOC130355	0.147970341	0.000663245	0.001819855
ZNF187	-0.090269978	0.038488364	0.068757976
ZNF816A	0.016874025	0.699417182	0.77281514
F7	0.188299769	1.38E-05	5.37E-05
CNOT1	0.011811363	0.786964136	0.847023012
SLC13A4	0.262282925	1.01E-09	8.34E-09
ZBTB11	-0.009978769	0.819396541	0.872311173
B3GALT5	0.045944019	0.292909459	0.386336392
EXOC2	0.271551592	2.41E-10	2.21E-09
IRS1	0.289821851	1.23E-11	1.41E-10
TMEM1	-0.056259664	0.197659379	0.278125291
MRPL34	-0.04955202	0.256604356	0.345113776
SAMM50	-0.123929584	0.004420199	0.010077791
CDC42EP3	-0.347256906	2.37E-16	6.90E-15
HSF2	-0.310969489	2.95E-13	4.52E-12
MFN2	-0.064386383	0.140293451	0.208524155
TSPAN7	-0.112822583	0.009606849	0.020092692
NUCB1	0.055761661	0.201663155	0.282659388
RHOH	0.216068833	5.65E-07	2.91E-06
ARL16	-0.115902863	0.007794481	0.016630209
TACR1	-5.83E-05	0.99893493	0.999259945
SFRS5	0.000471427	0.991393927	0.993656277
SNX25	0.281638765	4.78E-11	4.93E-10
RHBDF1	0.262242825	1.01E-09	8.38E-09
PCDH18	-0.085955469	0.048801718	0.084387448
HMG1L1	-0.124967936	0.004097676	0.009429869
MYO5C	0.247489011	8.80E-09	6.24E-08
MAPK10	0.102002083	0.019287529	0.037472044
LDHAL6A	-0.056702457	0.194148463	0.274063108
NUDT12	0.272056101	2.23E-10	2.06E-09
NCAM1	-0.086259745	0.048005385	0.083197608
GLIS2	0.208708939	1.38E-06	6.58E-06
GGTL4	0.066772005	0.126150631	0.190871119
DAPP1	-0.23504555	4.91E-08	3.02E-07

ATF7	0.10759481	0.013550866	0.02731048
KIAA0748	-0.186666181	1.64E-05	6.26E-05
NFIL3	-0.331386976	6.01E-15	1.29E-13
TM6SF1	0.134958847	0.001921667	0.00476465
SEZ6	0.083389373	0.055967394	0.094753353
NANOS3	-0.138269407	0.001478332	0.003775799
DNAJA3	0.225543144	1.71E-07	9.71E-07
CLDN6	-0.093266177	0.032467645	0.059066139
CIITA	-0.194333431	7.15E-06	2.96E-05
EPHA4	-0.180540431	3.11E-05	0.000111929
FANCC	-0.212696621	8.52E-07	4.26E-06
CMTM3	-0.018733142	0.668174819	0.746069904
PSG3	0.066386934	0.128355381	0.193635239
MRPL15	-0.125674042	0.00389068	0.00899729
C21orf59	0.264858375	6.81E-10	5.80E-09
PLCXD2	-0.00023014	0.995798648	0.997095894
C2orf34	-0.180630886	3.08E-05	0.000110958
UBE2L6	0.089903489	0.039285988	0.069999866
MED14	0.036458916	0.404020632	0.502492489
HP1BP3	-0.123423522	0.004585555	0.010393136
C6orf208	-0.052204587	0.231984387	0.316501441
TPBG	0.381296837	1.20E-19	5.91E-18
OSR2	0.27340094	1.80E-10	1.68E-09
XPC	0.155193812	0.00035356	0.001023559
KLHL7	-0.256182974	2.50E-09	1.96E-08
CCR3	0.02572774	0.556028032	0.647173266
AGTPBP1	-0.340255768	1.01E-15	2.53E-14
PCSK6	0.245197141	1.22E-08	8.41E-08
STAT5A	-0.287394912	1.84E-11	2.05E-10
FAM18B	0.033733454	0.440087494	0.537884715
LONRF2	0.200408933	3.61E-06	1.59E-05
PTPN2	-0.336708058	2.07E-15	4.87E-14
SF3A3	-0.340806433	9.01E-16	2.28E-14
EFCBP2	0.118612991	0.006459406	0.01410973
HCFC1	-0.076020288	0.081530291	0.131479087
AHNAK	0.319039831	6.56E-14	1.17E-12
ACTR5	0.215864953	5.79E-07	2.98E-06
KIF14	-0.131997114	0.0024182	0.005898259
TENC1	0.175644689	5.12E-05	0.0001774
HEATR5B	-0.011116868	0.799214972	0.85616252
YIPF2	-0.111465524	0.010517859	0.021827309
MYEOV2	0.21681807	5.15E-07	2.69E-06
DUSP18	0.14950015	0.000581858	0.001612594
KIAA1012	-0.052552021	0.228887915	0.313309495
AHR	-0.16519973	0.000141248	0.000446319
C17orf53	0.012996212	0.766185924	0.829158262
PTPRH	0.176723811	4.59E-05	0.000160268
ATP6V1C1	0.239052307	2.85E-08	1.83E-07
TAS2R3	-0.064724727	0.138593732	0.206396914
LOC440356	0.114103821	0.008811831	0.018584402
COQ10B	0.262691406	9.46E-10	7.86E-09

PSMF1	0.191549812	9.70E-06	3.88E-05
SORBS2	-0.194973491	6.66E-06	2.78E-05
NFE2L2	-0.035074861	0.422110962	0.520987616
TMCO7	0.033099139	0.44873833	0.546178145
SH3PXD2A	-0.077137433	0.077136395	0.125412928
SH2D2A	-0.302287378	1.41E-12	1.92E-11
SPINK5	-0.0306315	0.483292321	0.579291321
MRPS24	-0.019522624	0.65507528	0.735047061
OPA3	0.026364834	0.546285424	0.638249872
TRAF7	0.102289952	0.018947354	0.036881064
C4orf35	-0.019197477	0.660457795	0.739871558
MT1G	-0.098434267	0.023966003	0.045287939
MGC39545	-0.068830822	0.114858035	0.17643319
HS1BP3	0.256668257	2.33E-09	1.84E-08
OR2B2	-0.04933022	0.25874204	0.347456826
CHRM4	0.026908913	0.538032557	0.631005568
SFRP2	0.126734055	0.003597682	0.008392316
RIC3	-0.052732643	0.227289779	0.311548117
ART1	0.102463321	0.018745017	0.036521898
C6orf1	0.336114544	2.34E-15	5.42E-14
DUS4L	0.059250097	0.174831412	0.251177186
C10orf104	-0.088212254	0.043150407	0.075830767
TNFAIP6	0.099732469	0.022160969	0.042266687
RTEL1	0.263347579	8.57E-10	7.19E-09
CCT4	-0.225338382	1.76E-07	9.95E-07
ZNF709	-0.141475007	0.001140554	0.00298691
CHMP6	0.211058416	1.04E-06	5.10E-06
UPP2	0.049168994	0.26030362	0.349401214
CYP19A1	-0.064623652	0.138834585	0.206705536
CD151	0.119962111	0.005874551	0.012979739
NDUFA13	0.241203808	2.12E-08	1.40E-07
ARFRP1	0.143336839	0.000978617	0.002602732
FAM26B	0.144779622	0.00086803	0.002326729
CRYBA1	0.059830807	0.171454739	0.247773253
MRPL41	0.330868965	6.66E-15	1.42E-13
NPFFR2	0.115814363	0.00784196	0.016714112
HRH2	0.08670659	0.046855682	0.081434593
SCAMP3	0.105366958	0.015625715	0.031044433
MTMR6	-0.09852758	0.023832145	0.045076549
MTG1	0.062023503	0.15546987	0.227403206
UBTD1	0.017306232	0.692106171	0.766615235
CRABP1	-0.305039437	8.65E-13	1.22E-11
FLJ33790	0.186089615	1.74E-05	6.61E-05
KIAA1908	0.186535956	1.66E-05	6.34E-05
GPR158	-0.061803152	0.156946178	0.22923089
PACSIN3	-0.088628506	0.042170733	0.074428196
OMD	0.093238525	0.032519301	0.059142615
CATSPER1	-0.006537727	0.881091541	0.916567736
HOXB8	0.044848765	0.304579592	0.398991491
FBXO46	-0.037403433	0.391945279	0.490915976
OAS1	0.170282431	8.68E-05	0.000285444

SVIL	-0.187960288	1.43E-05	5.54E-05
PHB2	-0.229576175	1.02E-07	5.92E-07
ADCY3	-0.224613371	1.93E-07	1.09E-06
NDRG2	-0.260883	1.24E-09	1.02E-08
ERMAP	-0.154579831	0.000373383	0.001076386
APBA2	-0.302358342	1.40E-12	1.90E-11
IGSF9	-0.092782891	0.033380608	0.060547893
WNT6	-0.329362148	8.96E-15	1.85E-13
MYCBPAP	0.098509716	0.02385772	0.045111046
ATP2B2	-0.106629064	0.014418462	0.028869789
CPVL	-0.197037567	5.29E-06	2.26E-05
TRAM2	-0.128669047	0.003113929	0.007384323
NOP5/NOP58	-0.174803841	5.56E-05	0.000191216
ZNRF4	-0.001942887	0.964542874	0.974367362
TLK1	-0.192224267	9.01E-06	3.63E-05
MTMR12	-0.220369345	3.31E-07	1.78E-06
ZNF384	-0.249618487	6.50E-09	4.76E-08
FAM9B	0.022596348	0.605104962	0.690185571
RPN1	0.01293602	0.767237581	0.830004202
PMVK	0.224252651	2.02E-07	1.13E-06
EIF3D	-0.313309764	1.92E-13	3.08E-12
SIX2	-0.097337339	0.025588934	0.047971449
HPS1	-0.08338412	0.055982913	0.094753353
RNF7	0.088504613	0.042460341	0.074853393
PSKH2	0.078163273	0.073272963	0.119956189
KCTD13	0.184780145	2.00E-05	7.51E-05
CSMD3	0.136636172	0.001683742	0.004236224
FBF1	0.099199709	0.022886941	0.043502875
IL8	-0.283185741	3.71E-11	3.91E-10
SERPINB13	-0.034470995	0.430149854	0.528153246
FBXL20	0.067547466	0.121800037	0.185245716
BLR1	0.092407644	0.034104499	0.06167899
SH2B1	0.025035437	0.566709834	0.656128558
RFNG	0.119116651	0.006235218	0.013663705
RAB20	-0.117825095	0.006824639	0.014804574
RBM7	-0.385728524	4.18E-20	2.18E-18
POLR1A	-0.087896365	0.04390661	0.077027602
TMPRSS4	0.223534909	2.22E-07	1.23E-06
TAF9	0.207088685	1.67E-06	7.80E-06
TERF2	0.000621947	0.988646295	0.991995742
TNFRSF1A	-0.067591441	0.121556872	0.184967385
ACADVL	0.186690841	1.64E-05	6.25E-05
GTF2H5	0.126954013	0.003539449	0.00827667
EDG8	0.017597458	0.687195969	0.762737908
C9orf140	-0.020497317	0.639047836	0.72084529
UST6	0.006508976	0.881610616	0.916797511
ZBTB8OS	-0.0961034	0.027527051	0.051198982
ZNF710	-0.015546461	0.722045396	0.792244531
GPR174	-0.166546888	0.000126189	0.000404132
ATP6V0A2	-0.224636701	1.93E-07	1.08E-06
KIAA0319L	0.20253036	2.83E-06	1.27E-05

XKRX	-0.024886954	0.569013546	0.6579286
DOPEY2	0.180751617	3.05E-05	0.000109652
SDHD	-0.32523483	2.00E-14	3.90E-13
SUMF1	0.195116394	6.55E-06	2.74E-05
OSM	-0.047307698	0.278802825	0.370191874
OPN3	-0.23718316	3.68E-08	2.31E-07
DAGLB	0.152763654	0.000438251	0.001240703
PPFIBP1	0.066017954	0.130495908	0.196430683
TRIM63	0.105681277	0.015317045	0.030490292
C10orf53	0.094810943	0.03032159	0.055705844
LYPD3	0.191713484	9.53E-06	3.82E-05
BCL7A	0.016700653	0.702357732	0.775506858
AGER	-0.124798819	0.004148709	0.009533039
TCF19	-0.163497147	0.000165746	0.000514733
SAT2	-0.037216118	0.394322537	0.492622771
PFTK1	0.011142393	0.798763814	0.85591919
GABRE	-0.250361093	5.84E-09	4.31E-08
C15orf38	0.052595093	0.228506089	0.312936289
FIS1	0.235174243	4.83E-08	2.98E-07
KCNV2	0.101261371	0.020187401	0.03890076
CLPS	-0.057601249	0.187162673	0.265604264
PPCDC	0.190511539	1.09E-05	4.31E-05
FOXN2	-0.071931949	0.099363457	0.156262378
NT5E	-0.118473268	0.006522868	0.014233184
CD83	-0.338420674	1.47E-15	3.55E-14
IL18	-0.216216301	5.55E-07	2.86E-06
VPS16	0.32048891	4.98E-14	9.06E-13
IGFBP2	0.289029157	1.40E-11	1.59E-10
NOTCH2	-0.184441627	2.08E-05	7.74E-05
SIGLEC1	0.00172584	0.968501745	0.976761888
CD93	-0.002188026	0.960072682	0.971447577
SULF2	0.184765626	2.01E-05	7.52E-05
CEP164	-0.097826183	0.024854324	0.046765372
P53AIP1	-0.020428963	0.640166472	0.721742508
TOR2A	0.327316733	1.34E-14	2.68E-13
ZNF136	0.039246846	0.36901552	0.467080369
MGP	-0.101506305	0.019885876	0.038403973
CCDC144A	-0.029114645	0.505227004	0.599506146
TRPC1	-0.076826103	0.07834111	0.127049559
SMS	0.073236239	0.093365008	0.148346624
MAPK7	-0.037296861	0.393296737	0.491771087
RRAGC	-0.29870154	2.66E-12	3.39E-11
PARD6A	0.185023715	1.95E-05	7.35E-05
NUB1	0.060755588	0.164109871	0.238786464
SYNGR4	0.119261514	0.006172043	0.013554248
OR11H12	-0.115332402	0.008165945	0.017344523
WIF1	-0.216647949	5.26E-07	2.74E-06
GCH1	0.059336576	0.174201764	0.250389585
OR11H4	0.049027544	0.261678995	0.351017483
SLC44A5	-0.094706432	0.030029324	0.055267978
GPRIN2	-0.478911613	1.64E-31	6.72E-29

LOC401431	-0.152869551	0.000434198	0.001230928
CPA4	-0.212795199	8.42E-07	4.22E-06
MELK	-0.126407169	0.003685828	0.008575163
IL15RA	-0.190419448	1.10E-05	4.35E-05
CUL3	0.164384595	0.000152519	0.000478489
HMBOX1	-0.064602622	0.138963413	0.20684726
PODXL	-0.236621825	3.97E-08	2.48E-07
CCT6B	-0.066003188	0.130582145	0.196512387
COMTD1	0.272292411	2.15E-10	1.99E-09
MUC20	0.037537917	0.390243885	0.489316813
GPX2	0.178295188	3.92E-05	0.000138528
ITK	-0.182649688	2.50E-05	9.16E-05
FBXL5	0.338249159	1.52E-15	3.66E-14
C13orf27	-0.288811888	1.45E-11	1.64E-10
DEFA5	-0.084514405	0.052724896	0.089932145
TRHDE	-0.00621432	0.88757873	0.920513005
MTP18	-0.04059658	0.352765742	0.450747641
UQCRQ	0.298647665	2.68E-12	3.40E-11
ITGB2	-0.190351522	1.11E-05	4.38E-05
CSRP2BP	0.109324002	0.012111773	0.024685214
TAS2R44	-0.025817281	0.55465365	0.646062757
PHPT1	0.296394804	3.98E-12	4.88E-11
FAM44C	0.134639076	0.001970379	0.004879525
ERH	0.159860353	0.000232024	0.000698685
MPHOSPH1	-0.084778653	0.051986438	0.088845083
MORC1	-0.032035092	0.463463796	0.56077113
PARVB	-0.116315886	0.007576277	0.01623224
LAMA1	-0.129964393	0.00282397	0.006772461
PGBD3	-0.210635154	1.09E-06	5.35E-06
GIMAP6	-0.072497984	0.09672363	0.152932271
AREG	0.2044612	2.27E-06	1.03E-05
LIPT1	-0.327741444	1.23E-14	2.48E-13
MGC99813	0.042269993	0.333256504	0.42924052
C1orf201	0.041810598	0.33854184	0.434591602
GRIN2A	-0.105310704	0.015681529	0.031145258
MAN2C1	0.191126251	1.02E-05	4.05E-05
NSUN5	0.181322233	2.87E-05	0.000103874
SF3B5	-0.208617031	1.39E-06	6.63E-06
MYC	-0.16939161	9.46E-05	0.000308709
NRXN1	-0.148039542	0.000659347	0.001809966
ZNF18	0.037361974	0.392470698	0.491196831
SPDYA	-0.155319948	0.000349611	0.001014037
SLC37A1	0.196239438	5.78E-06	2.44E-05
DECR2	0.314726014	1.47E-13	2.45E-12
ANKRD38	-0.160052541	0.000227977	0.00068852
SPTLC3	-0.173870767	6.10E-05	0.000207477
SUPT16H	0.088614772	0.042202753	0.074441976
DTWD2	0.181489046	2.82E-05	0.00010222
ULBP1	0.044274331	0.311747756	0.406044683
ZADH1	0.150520756	0.000532826	0.001486545
OIP5	-0.086867265	0.046447964	0.080794492

IL10RB	-0.082525861	0.058567026	0.09869242
OTUB2	0.122998197	0.004728828	0.010678504
VWA3A	0.136010981	0.001769072	0.004423758
SPIC	-0.19744638	5.05E-06	2.17E-05
OR6C4	0.013625557	0.75521612	0.820318658
PSCD4	-0.161916828	0.000192002	0.000587372
DPY19L2P2	0.017939372	0.681447886	0.757725687
TRAPPC6A	0.266790246	5.06E-10	4.44E-09
C21orf2	0.101666005	0.019691396	0.03812418
CEMP1	0.174018401	6.02E-05	0.000204915
LIN7B	0.226636012	1.49E-07	8.49E-07
E2F7	-0.034694005	0.427170791	0.525124589
VCP	0.078081215	0.073576081	0.120388325
LAMA3	0.109637651	0.01186573	0.024264174
BGN	0.101521147	0.019867732	0.038380987
GPR160	0.286860433	2.02E-11	2.23E-10
COCH	-0.183283082	2.34E-05	8.63E-05
GPR81	0.273657038	1.73E-10	1.63E-09
APOBEC3F	-0.230645164	8.82E-08	5.19E-07
tcag7.1017	0.122066517	0.005056884	0.011340183
C1orf32	-0.09189907	0.035106913	0.063324262
SCGB1D2	0.170836858	8.22E-05	0.000271467
FLJ43987	-0.03223246	0.460712287	0.557990911
C6orf170	-0.066208054	0.12938968	0.194860921
KLK9	0.21017827	1.15E-06	5.61E-06
GPD1L	0.254411672	3.25E-09	2.50E-08
VPS37B	-0.022992982	0.598782654	0.68462524
ATG3	-0.182582144	2.52E-05	9.22E-05
ADAMTS17	-0.038927375	0.37338554	0.471519769
KLHDC2	0.140638284	0.001221085	0.0031802
NDUFV2	0.158708055	0.000257736	0.000766724
BLK	-0.189290385	1.24E-05	4.87E-05
MATN4	-0.345543926	3.39E-16	9.55E-15
GPM6A	0.048602642	0.265840597	0.355668806
GBP4	-0.072218116	0.098021778	0.154626966
TMEM162	0.02260091	0.605032065	0.690185571
PKP2	-0.15775603	0.000280964	0.0008314
HRASLS	-0.264086331	7.66E-10	6.48E-09
MMP1	-0.065646637	0.13267777	0.199178615
SFXN3	0.260685377	1.28E-09	1.05E-08
FSD1	-0.171727558	7.54E-05	0.000251131
ST6GALNAC3	-0.254613095	3.15E-09	2.43E-08
CA12	0.447550946	2.85E-27	5.65E-25
NCOA6	0.117818929	0.006827568	0.014804574
C19orf58	-0.007147005	0.870103934	0.909749272
PPP4R1	-0.123719593	0.004488148	0.010209997
MAN1A2	-0.043137214	0.323424705	0.419211322
IKBKAP	-0.089034802	0.041232652	0.072961029
UPF1	0.049337085	0.258675689	0.347443603
KIAA1219	0.289884369	1.21E-11	1.40E-10
WNT16	-0.063324379	0.146967699	0.216923759

SNW1	-0.028294179	0.517305757	0.611087601
IL18RAP	-0.220208017	3.38E-07	1.81E-06
RPP30	-0.246724056	9.81E-09	6.90E-08
CDC40	-0.11411421	0.008805632	0.018584402
SETD3	0.085683951	0.049521629	0.085392176
SLAMF6	-0.182953749	2.42E-05	8.91E-05
ELK4	-0.110349717	0.011323487	0.023271431
TRIM47	-0.10625429	0.014768084	0.029521765
ACOX3	0.255148991	2.91E-09	2.27E-08
TRIM6	0.068662046	0.115752857	0.177453083
KIAA0372	0.053565836	0.220020421	0.303818901
TP53AP1	0.345426486	3.47E-16	9.70E-15
SMURF2	-0.140915963	0.001193797	0.003114406
ADAD1	0.030839784	0.480321178	0.576179267
EBP	0.128803798	0.003082552	0.007318382
KRTAP13-2	-0.063368805	0.146683738	0.216660654
FLJ36874	-0.160243159	0.000224028	0.000677926
TOR1A	0.090977706	0.036986875	0.066306791
P2RY4	0.064572039	0.139150932	0.207026151
GPBP1	0.175636478	5.12E-05	0.000177446
TRPV1	-0.003363276	0.938661402	0.956392537
ADAMTS12	0.051049729	0.242489664	0.328356957
PES1	-0.086739772	0.046771237	0.08131081
ATG4A	0.068741618	0.115330299	0.177025963
MAGEA10	-0.017782892	0.684076284	0.760098495
WFS1	0.446323151	4.09E-27	7.86E-25
CC2D1B	-0.116655235	0.007401101	0.015895693
PABPN1	0.016283503	0.709451133	0.781654724
SLC25A30	-0.123609898	0.004524017	0.010283987
SLCO1C1	0.01234001	0.777673798	0.83937444
SLC22A5	0.41460603	2.87E-23	2.42E-21
KIF23	-0.031950352	0.464647944	0.561651309
SYN2	-0.286391633	2.18E-11	2.39E-10
ASPN	0.136384715	0.001717598	0.004310821
CENTG2	0.027726039	0.525756338	0.618495451
QSOX2	-0.142832032	0.001020286	0.002703033
FLJ10815	0.270240226	2.96E-10	2.69E-09
STK24	-0.175407327	5.24E-05	0.000181254
SPEG	-0.101545738	0.0198377	0.038335016
STK10	0.076669715	0.078951999	0.12779043
DACT2	-0.216688869	5.23E-07	2.73E-06
AAAS	0.302779906	1.29E-12	1.78E-11
SSX3	0.076280663	0.080488511	0.129935378
ABCD3	0.06189068	0.156358493	0.228481077
C4orf12	0.107930068	0.01326062	0.026804586
PARVG	-0.175679197	5.10E-05	0.000176984
FIG4	0.058727495	0.17867255	0.255799188
C9orf46	-0.018579982	0.670728014	0.748512987
TMCO6	0.195746609	6.11E-06	2.57E-05
IGHMBP2	0.170946969	8.14E-05	0.000268881
DUS2L	0.106188021	0.014830675	0.02963725

FAM3C	-0.109455904	0.012007758	0.024513845
TMEM16D	-0.137829077	0.001531317	0.003894156
DCTN4	0.28628427	2.22E-11	2.42E-10
KCNH3	0.056624323	0.194764648	0.274806751
EIF2AK2	0.015362128	0.72520712	0.794513673
AP1S3	-0.097789061	0.024909464	0.046840457
CST4	0.113658391	0.009081317	0.01910401
PAM	-0.159756823	0.000234232	0.000703268
NUTF2	0.101386888	0.02003239	0.038626268
CITED2	-0.00531218	0.903259999	0.931903647
SLC39A4	0.105874728	0.015129744	0.030177314
C2orf52	-0.002771526	0.949437648	0.963949626
GRM3	0.118859102	0.00634897	0.013883292
C12orf49	-0.042704212	0.328309909	0.424291221
CCDC49	0.048163564	0.270188543	0.360388145
GRAMD1B	-0.227724259	1.29E-07	7.41E-07
FNDC4	-0.208596006	1.39E-06	6.64E-06
SIAH2	0.291899002	8.61E-12	1.02E-10
GDPD4	-0.005341718	0.902724746	0.931507713
C21orf87	0.013053917	0.765178103	0.82865096
ATP5A1	-0.140533334	0.001231548	0.00320067
C16orf63	0.068442811	0.116923345	0.178935204
LOC388135	0.222707356	2.46E-07	1.36E-06
ATP5J2	0.091090917	0.036751366	0.065966394
MMP3	-0.061894834	0.156330644	0.228481077
EMID2	0.060550221	0.16554261	0.240359271
CRHR1	0.056482552	0.19588635	0.276262653
WDR70	-0.193798482	7.58E-06	3.11E-05
C13orf31	-0.036142411	0.408116191	0.506357236
ZFAND1	-0.104446291	0.01656145	0.032702748
CCL18	-0.097643144	0.025127224	0.047192212
C3orf49	0.061006727	0.164390706	0.239138503
RINT1	-0.009870941	0.821315056	0.873748492
KIAA0408	-0.184503635	2.06E-05	7.70E-05
F13A1	0.066819671	0.125879774	0.190602003
SLC10A1	-0.013403006	0.759089792	0.823217484
OGN	0.082473719	0.05872715	0.098935136
GIPC2	-0.194967338	6.66E-06	2.78E-05
XPO6	-0.005563492	0.8987074	0.927829383
LCE1A	0.041795679	0.338714377	0.434635145
FMR1	0.00696569	0.873371227	0.911621062
LOC374920	-0.110821332	0.010976538	0.022656841
DUSP3	0.266834437	5.03E-10	4.41E-09
ANKMY1	0.215820877	5.82E-07	2.99E-06
C7orf50	0.307021632	6.05E-13	8.76E-12
BBS9	0.000973028	0.982238147	0.986916844
UNC119B	-0.125138631	0.004046743	0.009323126
C9orf72	-0.186846683	1.61E-05	6.15E-05
MGC35440	-0.172492235	6.99E-05	0.000234879
ENTPD6	0.121946946	0.005100439	0.01142952
PPP1R2P9	-0.046002346	0.292296484	0.385717685

ERCC4	0.223289596	2.29E-07	1.27E-06
FAHD2B	-0.062318354	0.153510909	0.225283671
HMHA1	0.069137599	0.113245464	0.174348112
HACL1	-0.081329318	0.062333807	0.103985507
RAD23A	0.091991033	0.034923816	0.063030979
FAM83B	-0.052278618	0.231322116	0.315948399
PPP5C	0.090167851	0.038709241	0.069092343
RNASEH2C	0.171068252	8.04E-05	0.00026641
C9orf153	-0.039664439	0.363939125	0.462177134
SCAMP4	0.455444721	2.68E-28	6.11E-26
GHITM	0.164567575	0.000149918	0.000471049
NDUFB7	0.100463639	0.021197189	0.040655495
ADCYAP1	-0.187068607	1.57E-05	6.02E-05
SP110	0.151484981	0.000490055	0.001376587
MAP3K7IP2	-0.189345319	1.23E-05	4.84E-05
DHH	-0.004686812	0.914602199	0.940209263
AGRN	0.04577166	0.294725859	0.388565461
WDR33	-0.006049824	0.889906898	0.921549293
CEP290	0.205354349	2.04E-06	9.42E-06
PRPS1L1	-0.172223368	7.18E-05	0.000240513
KLRA1	-0.151018359	0.000510338	0.001426395
GPR97	-0.013451409	0.758246792	0.822696607
CHD7	0.011429173	0.793699633	0.85263086
TLR10	-0.196473992	5.63E-06	2.38E-05
SLC30A8	0.235484644	4.63E-08	2.87E-07
HIC1	0.127082253	0.003505895	0.008206223
IAPP	-0.009999493	0.819027951	0.872069761
RXFP4	-0.081352299	0.062259623	0.103889937
GP1BB	0.078181084	0.073207308	0.119880622
SHQ1	0.112353402	0.009913557	0.020663886
NKX2-3	0.06068516	0.164600159	0.239386561
API5	-0.100615152	0.021002092	0.04030645
FTHP1	-0.025797435	0.554958135	0.646294995
MOV10L1	0.030369327	0.487046281	0.582541837
TRIM6-TRIM34	0.143013853	0.001005093	0.002667378
ADHFE1	-0.09102834	0.036881384	0.066136959
FAM117A	-0.014878162	0.733530399	0.801862831
DDI1	0.002345643	0.957199132	0.969816685
CDON	0.142948415	0.001010537	0.002680669
TRIM73	0.089742243	0.040020916	0.071144438
IGKC	-0.12768848	0.003351147	0.007876988
MMP14	-0.026897621	0.538203199	0.63108533
DYNC1LI1	0.041173117	0.345964429	0.442918026
C11orf66	0.182761102	2.47E-05	9.07E-05
TRBV3-1	-0.142832213	0.001020271	0.002703033
FASTKD5	0.012984715	0.766386756	0.829229661
BIVM	-0.193575225	7.77E-06	3.18E-05
LHX4	0.091606344	0.035695177	0.064253409
CXCL2	-0.301810178	1.54E-12	2.07E-11
RAB2B	0.022661628	0.604062345	0.689507956
IZUMO1	-0.122393423	0.004980903	0.01118311

MAP3K15	-0.159337876	0.00024337	0.00072786
FAM19A2	-0.022270201	0.61032595	0.695109144
ZC3H8	-0.164423867	0.000151957	0.000477213
ZMAT1	0.140207879	0.00126452	0.003280815
SPINK5L3	0.069863251	0.109501875	0.169475719
SLC10A6	-0.088830077	0.041703107	0.073708654
APPL2	0.153343812	0.000416469	0.001186687
CARD10	0.248597961	7.52E-09	5.41E-08
LOC402176	-0.312753759	2.12E-13	3.38E-12
EEF1D	0.182959096	2.42E-05	8.91E-05
RAB6A	-0.073341743	0.092892743	0.147672565
C12orf5	-0.173481281	6.34E-05	0.000214923
PAPOLG	-0.284792306	2.85E-11	3.08E-10
MSRB2	0.185468239	1.86E-05	7.04E-05
BCR	-0.1586035	0.000260196	0.000772548
PUS3	-0.283128417	3.75E-11	3.94E-10
TIAM2	-0.359338919	1.78E-17	6.14E-16
ZNF317	-0.094331521	0.030530205	0.055988736
CHD2	-0.119484306	0.006076005	0.013367211
FZD5	-0.062590034	0.15172251	0.223085058
NUDT8	-0.157229929	0.000294625	0.000868627
ZNF763	-0.000256219	0.995322561	0.996781503
PRC1	-0.019727329	0.651695711	0.73259176
ABCB9	0.18954494	1.21E-05	4.75E-05
SPATA3	-0.036033733	0.409528151	0.507811177
TRAK2	0.174924367	5.50E-05	0.000189144
STAB1	-0.037792511	0.38703521	0.485987239
LRRTM2	0.002126654	0.961191704	0.97241984
psiTPTE22	0.084503235	0.052756303	0.089960762
DBI	-0.074239302	0.088951897	0.142438858
SERPINA11	0.243538511	1.54E-08	1.04E-07
NAT5	0.155825962	0.000334179	0.000974332
C20orf58	0.084942487	0.051532942	0.088168074
RPS6KA4	-0.024322833	0.577806304	0.665716876
FLJ90650	-0.019759102	0.651171795	0.73227055
TGFBRAP1	0.072240299	0.097918381	0.154543153
CHRDL2	-0.272719062	2.01E-10	1.87E-09
FAHD2A	-0.126384766	0.00369194	0.008586135
CNTN1	-0.172264721	7.15E-05	0.000239799
BBS4	0.31469567	1.48E-13	2.46E-12
TMEM181	0.109442306	0.012018444	0.024527519
MINPP1	0.072301791	0.097632213	0.15413106
MPHOSPH6	-0.269027766	3.58E-10	3.24E-09
HOXC10	0.15532879	0.000349336	0.001013716
ITPKB	-0.121736506	0.005177917	0.011575661
CLPTM1L	0.112811859	0.009613765	0.020100319
MEOX2	-0.060390619	0.16666254	0.241757008
ATP6V0C	0.461253616	4.53E-29	1.33E-26
PRPF8	0.012762003	0.770280389	0.832665987
TMC5	0.15523418	0.000352292	0.001020849
FKBP3	0.14224678	0.001070642	0.002825485

PLEKHB2	0.242517413	1.77E-08	1.19E-07
OR4D6	-0.0575211	0.187778003	0.266293113
ZNF544	-0.017354431	0.69129261	0.766040414
D2HGDH	0.147080597	0.000715312	0.001946219
RPL18A	-0.328362575	1.09E-14	2.21E-13
HEL308	-0.099873263	0.021972483	0.041996535
MPP6	-0.445679442	4.94E-27	9.21E-25
TCERG1	-0.06939077	0.111928118	0.172740807
KRT16	-0.337919012	1.62E-15	3.90E-14
KLF17	-0.065719883	0.132245161	0.198577655
KLF5	-0.218529137	4.16E-07	2.21E-06
CDR1	-0.010622689	0.807962531	0.863577543
VCX3A	0.153627461	0.000406191	0.001159549
FBLN2	-0.09029127	0.038442451	0.068695911
C14orf104	-0.156116806	0.000325599	0.000952025
HBE1	0.03992835	0.36075351	0.458984757
OR4S2	0.019751657	0.651294546	0.732274668
C1orf108	-0.238694972	3.00E-08	1.91E-07
ROBO4	0.016888511	0.699171675	0.772682716
CPEB4	0.342373238	6.53E-16	1.72E-14
C11orf80	0.17416944	5.93E-05	0.000202539
BCKDHA	0.113061219	0.009454111	0.019786702
MYOC	-0.057541569	0.187620717	0.266192845
GIF	0.040664529	0.352881347	0.450747641
CKMT1A	0.062930048	0.149506629	0.220248266
RPL3	-0.234962888	4.97E-08	3.05E-07
THBS1	0.332283312	5.03E-15	1.10E-13
APOO	0.00182228	0.966742614	0.975787407
ARMCX1	-0.066542353	0.12746195	0.19252359
HSZFP36	0.153940836	0.00039511	0.001130541
SNAPC5	0.108320062	0.012929894	0.026213622
EIF4ENIF1	-0.142778423	0.001024806	0.00271267
ZNF433	0.095964115	0.027753555	0.051557887
TNFRSF21	-0.312199501	2.35E-13	3.71E-12
TMPRSS7	-0.040737685	0.351553868	0.44960581
SPATA18	0.038305696	0.380616452	0.479297678
HPDL	-0.338711004	1.38E-15	3.40E-14
MKL2	0.295611664	4.55E-12	5.57E-11
TBX3	0.195253113	6.46E-06	2.71E-05
C21orf93	0.081743012	0.061009488	0.102121658
DAXX	0.068109295	0.118721733	0.181452187
ELMO1	-0.204248623	2.32E-06	1.06E-05
RGS13	-0.048864972	0.263265941	0.352838333
TAF11	-0.216607554	5.28E-07	2.76E-06
UNC13A	0.048666404	0.265213212	0.354906647
LOC653314	0.08165274	0.061296466	0.10253318
ORC3L	-0.237536602	3.51E-08	2.21E-07
IMAA	0.059579898	0.172439205	0.248725895
TARBP2	0.291588948	9.08E-12	1.06E-10
CABIN1	-0.065254798	0.135010655	0.20213794
TRIOBP	-0.022231189	0.610951787	0.695551614

HIST1H2AC	0.124643916	0.004195957	0.009627216
RGS22	0.304113698	1.02E-12	1.41E-11
NCOA1	-0.065966227	0.130798181	0.196732369
IL25	-0.086687062	0.04690544	0.081498036
SNCG	0.154405065	0.000379212	0.001091144
GPR6	-0.004503793	0.917924976	0.942447934
AMDHD1	0.11017629	0.011453492	0.023515033
CHEK2	-0.252363655	4.37E-09	3.29E-08
C6orf142	-0.099647081	0.022275962	0.042472834
DRD4	0.082348001	0.059114712	0.09950626
C14orf68	0.020834579	0.633540462	0.715715653
GDF11	0.025181213	0.564452467	0.654518864
SEMG2	0.054212614	0.215371028	0.298269472
CD247	-0.208645422	1.39E-06	6.62E-06
CDAN1	0.240650425	2.29E-08	1.50E-07
RBMX2	-0.152790851	0.000437207	0.001238316
TGS1	-0.138275113	0.001477657	0.003775799
OIT3	-0.120247636	0.005757052	0.012733854
SYF2	-0.228961969	1.10E-07	6.38E-07
MCM4	-0.064830331	0.137573325	0.20520272
PKHD1L1	-0.111076546	0.010792742	0.022344973
CEP192	-0.33469069	3.11E-15	7.09E-14
IFT88	0.173439776	6.37E-05	0.00021557
RPL9	-0.15869914	0.000257945	0.000766974
RAB32	-0.126223264	0.003736275	0.008659765
DDX43	-0.098621077	0.023698673	0.044873546
P2RX2	0.138330601	0.001471104	0.003765229
OR5D18	-0.088885674	0.041770119	0.07380588
UBE1	0.10732818	0.013785678	0.027738264
SLC24A1	0.174064879	5.99E-05	0.000204142
ARHGAP5	-0.066751085	0.126269652	0.190957228
CETP	-0.022687312	0.603652362	0.689295892
KIAA1731	-0.169021854	9.81E-05	0.000319255
SLC9A4	0.064556409	0.139246843	0.207118731
PTPN6	0.10809414	0.013120584	0.026556442
BAHD1	0.173278711	6.47E-05	0.000218789
GRIK3	0.299288264	2.40E-12	3.10E-11
CACNB2	-0.16601729	0.000130734	0.000417384
PDE10A	-0.220697097	3.17E-07	1.72E-06
DGCR14	-0.053377326	0.221650372	0.305520765
PCDHB9	-0.111842073	0.010257695	0.021316176
RHOQ	-0.216533567	5.33E-07	2.78E-06
MAP3K4	-0.102579011	0.018611048	0.03629538
KTI12	-0.265592636	6.08E-10	5.25E-09
RPL23AP13	0.017273743	0.692654748	0.766923279
GNG11	0.031214698	0.474998188	0.571354342
CLCN3	0.13518565	0.001887789	0.004692002
GPAM	-0.087680299	0.044430248	0.077768744
VSTM2A	0.131936035	0.002500529	0.00608218
SLAMF7	-0.136056287	0.001762757	0.004413516
INTS2	0.211475198	9.88E-07	4.88E-06

PPP2CA	0.209322983	1.28E-06	6.17E-06
LRP12	-0.228351087	1.19E-07	6.88E-07
SEC14L2	0.096674148	0.026615433	0.049713943
DKFZP586H2123	0.045417873	0.298477765	0.392922238
MC3R	0.070203027	0.107782861	0.167236137
CIRH1A	-0.185218193	1.91E-05	7.21E-05
HIST1H2AB	0.351821682	9.01E-17	2.81E-15
POLH	-0.033069086	0.449150562	0.546463555
MGC16703	0.073931395	0.090288382	0.14424091
SNAPC2	0.225378111	1.75E-07	9.91E-07
FILIP1L	0.107426351	0.013698809	0.027581525
RASGRP4	0.127939449	0.003288919	0.007748491
LRRC1	0.040075031	0.35899051	0.457498994
GAS1	-0.160244807	0.000223994	0.000677926
PRAC	-0.054794681	0.209605103	0.291663675
DGKA	0.037021491	0.396801773	0.494815271
NT5C3	0.022146449	0.612312183	0.69644858
PEG3	-0.131594592	0.002494054	0.006071234
NADK	0.049792499	0.25430049	0.342314736
PRR17	0.215650009	5.95E-07	3.05E-06
LOC374569	-0.113928228	0.008917203	0.018778042
SGSH	0.165392832	0.000138696	0.000439381
NLRP8	-0.029979908	0.492651087	0.5874174
GALT	-0.030934958	0.478966849	0.57500335
MCF2	-0.021832383	0.61770198	0.701690278
ZNF263	0.194220062	7.24E-06	2.99E-05
TACSTD1	-0.073015156	0.094360874	0.149619653
TYR	0.001961623	0.964201186	0.974342332
ATP6AP2	0.000971703	0.982262333	0.986916844
RNUXA	0.239601613	2.65E-08	1.70E-07
ABHD10	-0.047989312	0.271927461	0.362157669
GDPD2	0.081635434	0.061351611	0.102541739
SLC35C1	-0.162762733	0.000177498	0.000547635
UBE2A	0.118982568	0.006294208	0.013778243
HERC5	-0.13651144	0.001700459	0.004275909
FAM112B	-0.059588108	0.172379969	0.248725895
FBXL16	0.22364199	2.19E-07	1.22E-06
DKFZP434A0131	0.052260963	0.231479933	0.31609374
ELA3A	-0.025255224	0.563308053	0.653543626
RBM41	-0.111167006	0.010728254	0.022222414
HAO2	-0.083465522	0.055742815	0.094477004
RNH1	0.208869969	1.35E-06	6.48E-06
SHANK2	0.008007986	0.854620154	0.898915383
OSBP2	0.081107365	0.063054042	0.105016064
DAK	0.345946379	3.11E-16	8.82E-15
C3orf58	-0.30334907	1.17E-12	1.61E-11
TCL1B	0.266138877	5.59E-10	4.88E-09
KBTBD2	-0.026475903	0.5445956	0.636759525
SUGT1L1	0.096687268	0.026594788	0.049690474
UBE2E2	-0.089679474	0.039780391	0.070737311
MYL9	0.080999858	0.063405358	0.105437919

CDC23	0.298562535	2.72E-12	3.44E-11
PBXIP1	0.073683921	0.091374194	0.145635023
CXorf40B	0.308639318	4.51E-13	6.66E-12
NBL1	0.121727954	0.005181088	0.011576493
RTBDN	0.178039302	4.02E-05	0.000141765
RAB11FIP5	0.082261825	0.059381597	0.099818874
TTTY13	0.08926346	0.040712508	0.072227701
SCOTIN	0.319809257	5.67E-14	1.02E-12
SOHLH1	0.075946051	0.081829312	0.13189212
CDKN1A	0.282210294	4.36E-11	4.52E-10
NCK1	-0.389642259	1.62E-20	9.51E-19
ZNF550	-0.159821907	0.000232842	0.000700345
SAPS3	0.174743453	5.60E-05	0.000192262
SPIN3	0.117863522	0.006806405	0.014773239
MAGEE2	-0.155596882	0.000341085	0.000990709
MIS12	-0.027206378	0.533546986	0.627060477
OR8H2	0.066312887	0.128782747	0.194137071
KIAA0774	-0.044470895	0.308676327	0.403240012
UNC5D	-0.077300791	0.077930204	0.126502857
CUL7	0.091271206	0.036378946	0.065388524
LIPC	-0.070908111	0.104283674	0.162834006
DIO1	0.238166836	3.22E-08	2.04E-07
C20orf11	0.169026201	9.80E-05	0.000319255
CTRL	-0.158830627	0.000254879	0.000758961
HS3ST2	0.068947325	0.11424353	0.175708721
PAK4	0.193434848	7.89E-06	3.23E-05
CCRL1	0.019587123	0.654009674	0.734253329
RNF10	0.29428957	5.72E-12	6.95E-11
ZNF567	-0.386651814	3.35E-20	1.79E-18
ZNF660	-0.141788485	0.001123941	0.002949685
TCEAL3	0.24146836	2.05E-08	1.35E-07
MAGOH	-0.21099749	1.05E-06	5.13E-06
CENPB	0.168828157	9.99E-05	0.000324415
C19orf7	-0.041889721	0.337627723	0.433871028
LOC388965	0.068880958	0.114593275	0.176158512
ZCCHC13	0.010660644	0.807289821	0.8631586
JMJD1A	-0.177773709	4.13E-05	0.000145383
HIST1H4H	0.251993046	4.62E-09	3.46E-08
TBRG1	-0.06872102	0.115439566	0.177149461
GPC3	-0.108837979	0.012501944	0.025421446
TAF1C	-0.048314508	0.268688377	0.3586984
EBNA1BP2	-0.148316037	0.000643981	0.001769365
CIAPIN1	0.017377895	0.690896695	0.765877732
PDGFRA	-0.113957197	0.008899742	0.018747692
CSTB	-0.07069234	0.105344813	0.164199051
CENPI	-0.09830218	0.024156593	0.045578058
GTF2E2	-0.134630661	0.001971676	0.004880771
RPP21	0.05519145	0.206319485	0.28822294
CCNF	0.110633968	0.011113231	0.022892883
KCNQ3	0.000923618	0.983139956	0.987475922
FAM79A	-0.09630742	0.027198145	0.050663857

SLC22A12	0.026317654	0.547004002	0.638725334
NOVA1	0.067366401	0.122805253	0.186589944
FZD3	0.038758026	0.375013253	0.472822738
AKAP8	-0.279962495	6.29E-11	6.39E-10
SOCS5	-0.373530154	7.35E-19	3.27E-17
CFDP1	-0.136832593	0.001657723	0.004182742
DLG5	0.240818115	2.24E-08	1.47E-07
PGM5	-0.050017884	0.252154306	0.339777543
C1orf144	-0.057646817	0.186813498	0.265292425
HDAC10	0.107472528	0.013658115	0.027508597
RND2	-0.002090044	0.961859273	0.972935133
C20orf199	0.079130555	0.069776082	0.114904427
RNMT	-0.277319424	9.64E-11	9.48E-10
SLURP1	-0.07455702	0.087589537	0.140476804
ASTN1	-0.085382366	0.050331651	0.086449532
SH3BGR	0.065898486	0.131194854	0.197193145
MYCL1	-0.065295824	0.134764926	0.201819174
ZHX1	-0.098328718	0.024118196	0.045530718
CENPK	-0.017355575	0.691273306	0.766040414
FOSB	0.096254427	0.02728325	0.050806996
LOC643406	0.087035917	0.046023211	0.080169044
C2orf59	0.055054384	0.207450276	0.289254364
TMEM135	0.086017217	0.048639228	0.08413013
SLC27A2	0.26846692	3.90E-10	3.49E-09
KRT33A	0.329079485	9.47E-15	1.94E-13
OVOL1	0.274037225	1.63E-10	1.54E-09
PAMCI	-0.336417047	2.20E-15	5.12E-14
S100A7	-0.040364383	0.355528591	0.453651236
ZNF789	-0.327263613	1.35E-14	2.69E-13
HARS2	0.318720675	6.96E-14	1.24E-12
RPL23A	-0.034432942	0.430659382	0.528567773
TCF23	0.05761562	0.1870525	0.265570497
UPF3B	-0.292309628	8.03E-12	9.53E-11
C17orf78	0.033505987	0.443614529	0.541334736
HLA-DOB	-0.370067136	1.62E-18	6.83E-17
C14orf142	-0.053859929	0.217494702	0.300939451
TEKT5	0.2346596	5.17E-08	3.17E-07
DMWD	0.109372614	0.012073345	0.024623217
POLD1	-0.171194696	7.94E-05	0.000263568
GSCL	0.05275827	0.227063677	0.311307591
CALD1	-0.220580854	3.22E-07	1.74E-06
SCRT1	0.087643526	0.044519887	0.077879365
AIG1	0.124971264	0.004096677	0.009429869
UNC84B	-0.2228958	2.40E-07	1.33E-06
ZNF404	-0.030890189	0.47960365	0.575655444
TMED6	-0.053162038	0.223522393	0.307412032
KIAA1462	0.070114484	0.108228759	0.167843288
LRRC27	0.183418894	2.31E-05	8.53E-05
PYGO1	-0.067228508	0.123575119	0.18757428
PIGU	0.105078886	0.015913387	0.031524296
ALAS2	-0.045081216	0.302077415	0.396726618

WRNIP1	-0.11826787	0.006617174	0.014408288
CNNM3	0.233737884	5.85E-08	3.55E-07
ZNF2	-0.0696202	0.110744742	0.171183866
ST3GAL5	-0.007067646	0.871533704	0.910166567
MRPL23	0.145948303	0.00078705	0.002125435
TSSK6	0.07299646	0.094445474	0.149676603
PSMA6	0.224391944	1.99E-07	1.11E-06
C16orf70	0.274621573	1.48E-10	1.41E-09
KIAA1602	0.032353897	0.459023867	0.556274686
ALMS1	-0.002857906	0.94786398	0.962897012
DCN	0.066453933	0.127969642	0.193148093
TMEM132D	-0.056303633	0.197308688	0.277758794
SUCLG2	0.108474223	0.012801181	0.02597837
ABHD14A	0.258136239	1.87E-09	1.50E-08
DEXI	0.037453899	0.391306291	0.490249059
AMPD2	-0.278308891	8.22E-11	8.22E-10
IFNAR2	-0.270081829	3.04E-10	2.75E-09
CYB5A	0.186283671	1.71E-05	6.50E-05
TLOC1	0.011765744	0.78776729	0.847739073
NXF5	-0.037187755	0.394683244	0.492873125
NRBF2	-0.301193366	1.72E-12	2.28E-11
KCTD3	0.230110609	9.46E-08	5.54E-07
ITGAE	-0.024591599	0.573609192	0.661997546
SLC30A3	-0.172396711	7.06E-05	0.000236837
ZRF1	-0.189607528	1.20E-05	4.72E-05
IFRD2	0.030275946	0.488387143	0.583500528
XAB1	-0.305889105	7.42E-13	1.06E-11
PYCR2	0.234366473	5.38E-08	3.28E-07
SERPINB3	-0.15436098	0.000380696	0.001093473
TMLHE	-0.19663938	5.53E-06	2.35E-05
GEFT	-0.028520236	0.513962977	0.608112054
ABCA5	0.081717495	0.061090494	0.102216448
EMR4	-0.000759047	0.986143759	0.990169493
TSFM	0.139501604	0.001338887	0.003453361
HIST3H2BB	0.222225131	2.62E-07	1.44E-06
ARHGEF19	-0.189016887	1.28E-05	5.01E-05
TSPAN17	0.214178037	7.12E-07	3.60E-06
ABCC8	0.271827523	2.31E-10	2.13E-09
MAP1S	0.019795164	0.650577359	0.731735903
C22orf36	0.111429227	0.010543245	0.021872608
BNC2	0.102838821	0.018313228	0.035794037
HIST1H4A	-0.015182847	0.728286706	0.796980771
NDUFS3	-0.009909787	0.82062377	0.873315258
WDR3	-0.319497573	6.01E-14	1.08E-12
XKR4	-0.125380933	0.003975428	0.009172573
TTC33	-0.230966314	8.45E-08	5.00E-07
STMN2	0.101860241	0.019457092	0.037765675
CPN2	-0.019112171	0.661872866	0.741051758
HSPC105	-0.193028017	8.25E-06	3.35E-05
PCOLCE2	-0.244445033	1.35E-08	9.29E-08
C3orf55	-0.248633329	7.48E-09	5.39E-08

KLHDC9	0.27617127	1.16E-10	1.13E-09
TBC1D23	-0.070250179	0.107546001	0.166910741
ATXN2L	0.120728288	0.00556402	0.012346864
MAP2K3	-0.128409379	0.003175215	0.007515164
SCAP	0.14859126	0.000629016	0.001732118
ZNF486	0.138392341	0.001463844	0.003748927
C20orf96	0.12436516	0.00428221	0.009791157
NARS	-0.043021462	0.324725992	0.420366342
ADAMTSL1	-0.014795038	0.734963153	0.803286247
PRCC	0.003523484	0.935745737	0.95468733
CCDC126	0.036007921	0.409863936	0.508013172
ZNF675	0.049041028	0.261547671	0.350917876
CALCOCO1	0.097062698	0.026009811	0.048694449
ANKRD43	-0.011479358	0.792814318	0.851977497
CWF19L2	-0.257046458	2.20E-09	1.74E-08
ZBTB32	-0.138755247	0.00142183	0.003647406
BRAF	-0.025243431	0.563490319	0.653631763
ODF4	-0.022943796	0.59956505	0.685392358
MGC14376	0.105829887	0.015172979	0.030242674
HORMAD1	-0.388575848	2.10E-20	1.21E-18
AAK1	0.008482076	0.846117572	0.893027283
PEBP1	0.124686164	0.004183023	0.009604707
TNFSF5IP1	-0.225624554	1.70E-07	9.62E-07
DKFZp564N2472	-0.050135617	0.251038231	0.338590499
RMND1	0.252706641	4.16E-09	3.14E-08
IGKV1-5	-0.134990906	0.001916845	0.004756529
COL1A2	0.165278396	0.000140203	0.0004437
SERPINA5	0.38998488	1.49E-20	8.83E-19
AANAT	0.003562324	0.935039007	0.954157459
C19orf21	0.433247152	1.77E-25	2.47E-23
GEMIN5	0.213325889	7.90E-07	3.97E-06
UBR4	-0.180424154	3.15E-05	0.000113209
LTBP3	0.030581037	0.484013656	0.579929846
AMHR2	0.184842728	1.99E-05	7.48E-05
PROCR	0.048102922	0.270792841	0.360959284
MYBBP1A	-0.003167165	0.94223161	0.959235458
C20orf39	0.094994816	0.029374646	0.054144094
ZNF697	-0.02254915	0.605859277	0.690633535
PASK	0.086573222	0.047196386	0.081934098
ZNF776	0.188557132	1.34E-05	5.25E-05
RFXDC2	0.101770882	0.019564581	0.037949866
KIAA0467	-0.176768141	4.57E-05	0.000159796
C10orf96	0.014466468	0.740874394	0.80702155
ZNF503	-0.205033339	2.12E-06	9.75E-06
GULP1	-0.092563701	0.033801838	0.061203623
KCNE4	0.18741532	1.52E-05	5.82E-05
DKFZp434K191	-0.178025545	4.02E-05	0.000141882
LOC196913	0.02314898	0.597358725	0.684014674
BHLHB4	0.06784771	0.120147326	0.183458135
CH25H	-0.082413134	0.058913658	0.099194985
LOC81691	0.101678808	0.019675876	0.038112627

ALPL	-0.282551464	4.12E-11	4.29E-10
COL12A1	0.104424866	0.016583797	0.032736363
FOLR3	-0.196124665	5.86E-06	2.47E-05
GPR123	0.009186876	0.833510535	0.882318131
TRIM62	0.295589405	4.57E-12	5.58E-11
ABLIM1	-0.311309827	2.77E-13	4.28E-12
MAST3	0.008896934	0.838691864	0.886711876
RHBDD1	0.117817884	0.006828065	0.014804574
LOC338809	0.110822467	0.011207144	0.023055446
RYBP	0.139370652	0.00135311	0.00348566
TTC26	0.164728269	0.000147668	0.000464693
ZNF22	-0.376378235	3.80E-19	1.76E-17
ISCA2	0.142672141	0.001033822	0.002734181
RDM1	-0.005024929	0.908467523	0.936176772
PIGM	0.128684825	0.00311024	0.007378421
GNB3	-0.042912627	0.325952608	0.421599198
ACTR2	-0.182436217	2.56E-05	9.33E-05
HMGB1	-0.13303753	0.002231789	0.005476166
EDG1	-0.007282407	0.867665436	0.907977325
SOAT2	0.003962378	0.927762692	0.948904324
OR10AD1	0.044295822	0.311512705	0.405977644
RAP1GDS1	0.156331296	0.000319404	0.000935691
LCE1F	-0.008213491	0.85093239	0.896570127
ESM1	-0.016234911	0.710279059	0.782247034
RCN3	0.026976199	0.537016281	0.630174258
CREBL1	0.082441876	0.058825117	0.099073033
DBNL	0.052804204	0.226658808	0.310891147
PTGER3	0.154356205	0.000380857	0.001093473
USP30	0.268804869	3.70E-10	3.34E-09
BCL2L12	-0.056565202	0.195231843	0.275402753
KIF26B	0.049348485	0.258565537	0.347371529
ZNF416	-0.023067278	0.59760171	0.684038145
ZNF225	-0.09837326	0.024053868	0.04544001
C17orf70	0.181428171	2.84E-05	0.000102804
ZNF554	0.045475936	0.297859836	0.392192748
RAE1	0.246497696	1.01E-08	7.09E-08
TNIK	-0.068573401	0.116225018	0.178043756
ACTN3	-0.007551078	0.862830578	0.904765556
MGC45922	0.085268288	0.050640923	0.086907908
CCNA1	-0.171432751	7.76E-05	0.000258203
RYK	-0.299940779	2.14E-12	2.78E-11
IL26	0.039576276	0.365007216	0.463325136
LRP3	-0.014046639	0.747903683	0.813813439
QARS	-0.010365106	0.812531652	0.866881602
SOX7	-0.048035379	0.271467005	0.361701108
BID	-0.316259011	1.11E-13	1.90E-12
OR2S2	0.008741297	0.841476029	0.888891274
CXCL14	0.101307942	0.020129765	0.038801858
C11orf47	-0.037529926	0.390344851	0.489343626
MGC29891	0.032537059	0.456483796	0.553856952
HSPB8	0.417704164	1.26E-23	1.17E-21

PRDM14	0.027473373	0.529930459	0.623048258
NUFIP2	0.057956117	0.184456065	0.262672613
MNAT1	0.248652616	7.46E-09	5.38E-08
ZDHHC2	-0.056132449	0.198676585	0.279291834
MBNL2	-0.243368059	1.57E-08	1.07E-07
ADD3	-0.220613868	3.21E-07	1.73E-06
CSNK2A1P	-0.025277789	0.56295935	0.653262321
KLK6	-0.272843539	1.97E-10	1.84E-09
TMEM111	0.310599763	3.16E-13	4.78E-12
KIAA1279	0.242989478	1.66E-08	1.12E-07
NUBP2	0.347258222	2.37E-16	6.90E-15
RAB42	-0.135697412	0.001813346	0.004528945
ID3	0.054713663	0.210280631	0.292339046
TM9SF1	0.329259087	9.14E-15	1.88E-13
MDP-1	0.331799622	5.54E-15	1.20E-13
POU4F2	0.107694168	0.013464269	0.027153753
IQCK	0.145046367	0.000848896	0.002279415
C16orf14	0.288660241	1.49E-11	1.68E-10
CAPN3	-0.183451247	2.30E-05	8.51E-05
FAM43B	0.126951805	0.00354003	0.00827667
RECQL	-0.324384555	2.36E-14	4.57E-13
AP1G1	0.119117666	0.006234773	0.013663705
CTNNBL1	0.25181066	4.74E-09	3.55E-08
ECHDC1	-0.315020349	1.39E-13	2.33E-12
SMARCC1	-0.034893334	0.424518245	0.523013963
FOXQ1	-0.086068184	0.048505446	0.083922338
GNAI3	-0.084792581	0.051947756	0.088803656
POLG2	0.1319697	0.002423298	0.005908351
CD4	-0.062023044	0.155472935	0.227403206
ITLN1	-0.069467319	0.111532183	0.172227874
EBI2	-0.182552326	2.53E-05	9.24E-05
IRF1	-0.077748421	0.074815899	0.122009816
PTPRE	0.031515092	0.470756638	0.566917855
PTK2B	-0.071613676	0.100872781	0.15815062
NXNL2	0.207385173	1.61E-06	7.57E-06
SOX4	-0.162818927	0.000176572	0.000545599
TSPAN3	-0.005241589	0.904539356	0.93259767
SH2D1A	-0.190974696	1.03E-05	4.12E-05
C8orf58	-0.079506203	0.068455416	0.113001974
USP20	0.07239033	0.097221344	0.153561275
DUSP22	-0.301839722	1.53E-12	2.06E-11
CALB1	0.014354155	0.742577619	0.808590363
L3MBTL2	0.012747796	0.770528969	0.832685875
MCRS1	0.313995334	1.69E-13	2.75E-12
TMEM118	-0.05943873	0.173460162	0.249787877
C18orf8	-0.128283807	0.003205243	0.007580399
FLJ10241	0.188402329	1.36E-05	5.33E-05
GJA12	-0.010759387	0.805540358	0.861737589
PKD1	0.098324636	0.024124098	0.045530718
ZFP3	-0.187818623	1.45E-05	5.61E-05
JAM3	0.110434035	0.011260754	0.023157985

LAPTM4A	0.018540793	0.671381905	0.749106757
DIRC2	0.156714199	0.000308617	0.000906682
KIAA2022	-0.083011932	0.057091656	0.096497139
MYOM1	-0.210488652	1.11E-06	5.44E-06
TRPM8	-0.222659676	2.48E-07	1.36E-06
MOP-1	0.060418661	0.166465358	0.241527958
PHKG2	0.302597666	1.34E-12	1.83E-11
ZNF650	0.03573534	0.41341978	0.512007699
KIAA1522	0.060177151	0.168169317	0.243655308
PSG8	0.09033824	0.038341327	0.068535123
DDX19B	-0.055055994	0.207436972	0.289254364
MOBKL1B	-0.123682053	0.004500394	0.010234069
DIAPH2	-0.276591524	1.08E-10	1.06E-09
PTPN12	-0.098618092	0.023702924	0.044873546
CLN8	-0.001298692	0.976294954	0.98226924
CRYZL1	0.033417757	0.444381014	0.541947412
CRY2	0.015729979	0.718902319	0.789380421
FCGR2B	-0.160213536	0.000224637	0.000679102
PNPLA4	0.271522154	2.42E-10	2.22E-09
ZNF454	-0.346770807	2.62E-16	7.60E-15
DKFZp434B1231	-0.033615365	0.441690739	0.539629714
CLDN11	-0.003894155	0.929003144	0.949618617
RFWD2	-0.051445343	0.238853988	0.324219243
CIB2	-0.23115407	8.25E-08	4.89E-07
MXRA8	0.117048188	0.007202786	0.015518546
HRK	-0.199973193	3.80E-06	1.66E-05
MAML2	-0.382549419	8.93E-20	4.46E-18
C4orf31	0.089773405	0.039572448	0.07044904
C6orf192	-0.447807727	2.64E-27	5.41E-25
COG6	0.037954993	0.384995855	0.483951792
FAM5B	-0.020642984	0.636666683	0.718587268
NFATC1	-0.304273643	9.92E-13	1.38E-11
SEPT10	-0.208687614	1.38E-06	6.59E-06
SCYL1	0.041054935	0.347351769	0.444509059
RPP40	-0.315979749	1.17E-13	1.97E-12
SCOC	0.042741558	0.327886695	0.423833358
KIAA1450	-0.059209636	0.175126594	0.251542496
CTDSPL2	-0.162001944	0.000190494	0.000583339
TBX5	0.024480942	0.575335499	0.663491745
NAPG	0.004976206	0.909351202	0.93677342
RHD	-0.058121559	0.183204144	0.26131345
C14orf45	0.158803433	0.000255511	0.000760472
ZBTB22	-0.03505593	0.422361639	0.521192397
PLCG1	-0.046000591	0.292314915	0.385717685
ANKRD10	-0.181680106	2.77E-05	0.000100339
AQP7P2	0.004894534	0.910832728	0.937357397
TAGLN2	0.001664458	0.96962148	0.977089885
HTR2C	0.022364331	0.608817059	0.6936476
SLC16A7	-0.165803644	0.000133408	0.000424963
C17orf83	-0.117211466	0.007121791	0.01537097
TSGA14	0.078412313	0.07235926	0.11868154

MDH1	-0.25459398	3.16E-09	2.43E-08
PPP3R2	0.071628963	0.100799872	0.158076616
DCBLD2	-0.301997959	1.49E-12	2.01E-11
RBM33	0.055630737	0.202725457	0.284083599
DPH3	-0.109561696	0.011924904	0.024377072
SYT10	0.035408993	0.417700874	0.516581391
FMO4	0.038245622	0.381364456	0.480042997
THYN1	-0.348858994	1.69E-16	4.99E-15
DRD5	-0.142120106	0.001081839	0.00284892
OTOR	0.24875511	7.35E-09	5.32E-08
PGRMC2	0.129918094	0.002833895	0.006790966
KATNAL1	-0.023007611	0.598550049	0.68462524
PAQR6	-0.063021693	0.148913613	0.219490217
UBE2I	0.113528227	0.00916144	0.019213403
C14orf28	0.136066764	0.0017613	0.004413298
C8orf70	0.036018578	0.409725274	0.507943691
FLYWCH1	0.40644047	2.42E-22	1.78E-20
ANGPTL3	-0.030569895	0.484590717	0.580395075
GLRX2	-0.003320747	0.939435547	0.956864201
ATP11A	-0.307444664	5.61E-13	8.15E-12
ARL5B	-0.205381138	2.04E-06	9.40E-06
MUC16	-0.134048558	0.002063316	0.005083064
SLC25A5	-0.116512642	0.007474263	0.016036023
ACRC	-0.117666529	0.006900341	0.014950739
MYO1C	0.212146204	9.11E-07	4.54E-06
FAM89B	-0.075509955	0.083603814	0.134575877
FAS	-0.282016711	4.50E-11	4.65E-10
KIFAP3	-0.002913871	0.94684452	0.962656573
GLRA2	0.005954154	0.892049322	0.923125426
BTN3A2	-0.173905586	6.08E-05	0.000206877
CNKSR3	-0.3698528	1.70E-18	7.13E-17
CSTF3	-0.179807362	3.36E-05	0.000120111
ARPM1	-0.219679881	3.61E-07	1.93E-06
KIAA1530	0.268744353	3.74E-10	3.36E-09
C9orf150	0.250933529	5.38E-09	3.98E-08
PRKCI	-0.031016328	0.477810598	0.573727273
tcag7.1015	-0.112322336	0.009934169	0.020699831
SOD3	-0.249833762	6.30E-09	4.63E-08
ZNF574	0.09932834	0.022709799	0.043219608
CYP21A2	0.192697605	8.56E-06	3.46E-05
RPL12	-0.207574611	1.57E-06	7.43E-06
COMMD2	-0.322084253	3.67E-14	6.85E-13
WIZ	-0.177667857	4.17E-05	0.000146781
LOC344405	-0.013716384	0.753636965	0.818748003
ALDH4A1	0.19702703	5.30E-06	2.26E-05
CRYAB	-0.237976661	3.30E-08	2.08E-07
COPA	-0.090131803	0.03878746	0.069211867
PCDHGA7	0.24519805	1.22E-08	8.41E-08
KIF11	-0.114083975	0.008823685	0.018600218
RASD2	-0.304298754	9.88E-13	1.37E-11
SLC26A3	-0.004187295	0.923674435	0.946914759

ZNF175	0.046273116	0.289462136	0.382445784
JAKMIP2	-0.119609841	0.006022484	0.013268453
C8orf4	0.029367792	0.501530352	0.595694443
PTHLH	0.083064334	0.056934458	0.096272797
SLC40A1	0.066415784	0.128189169	0.193431951
OR7D4	0.056706336	0.194117911	0.274063108
PCDHB17	-0.03240883	0.458261236	0.555459953
CD36	0.021090873	0.629368733	0.711675081
C6orf203	-0.165255606	0.000140505	0.000444267
PRKG2	0.049770727	0.257708846	0.346296262
LOC400566	0.207078015	1.67E-06	7.80E-06
ANAPC13	0.13042526	0.002726887	0.006570386
SLCO3A1	-0.06995993	0.109010567	0.168842815
ZNF692	-0.053842355	0.217645046	0.301079728
FANCL	-0.310993204	2.94E-13	4.51E-12
SH3GLB1	-0.281208225	5.13E-11	5.25E-10
C12orf61	0.000557667	0.989878182	0.99265064
KBTBD6	-0.065598872	0.13296047	0.199505596
SUPT5H	-0.155730315	0.000337046	0.000980832
XRCC6	-0.147260255	0.000704503	0.001917658
HUS1B	0.151067471	0.000508167	0.001420973
FAM133B	-0.016342551	0.708445509	0.78082657
LOC728276	0.088308306	0.042922657	0.075495257
KCTD18	-0.079490884	0.068508867	0.113059855
SOS2	-0.055517801	0.203645072	0.28522895
CCDC99	-0.146791459	0.000733031	0.001991785
C1QTNF5	0.101145629	0.020331263	0.039165707
NNAT	0.029204552	0.503912479	0.598177188
USP16	-0.155809732	0.000334664	0.000975206
LARS	-0.03960747	0.364629077	0.462957711
ZBTB2	0.065557304	0.133206871	0.199680413
ABO	-0.094080599	0.030977388	0.05670734
TRAF3	-0.185387589	1.88E-05	7.09E-05
GALNT5	0.08912649	0.041023413	0.07269538
NAP5	0.127144585	0.00348969	0.008174515
ALG14	-0.116017565	0.007733327	0.016516924
KIAA0515	0.042423098	0.331506887	0.427255471
WDR75	-0.265102086	6.56E-10	5.62E-09
TEX261	-0.1299118	0.002835247	0.006791559
LY86	0.007658629	0.860896573	0.903353759
LOC389072	-0.216378916	5.44E-07	2.82E-06
FLJ13611	0.203009439	2.68E-06	1.21E-05
MRGPRX2	0.162393008	0.000183706	0.000565937
SNRPA	-0.075209535	0.08484422	0.136358366
OR2G2	0.0896828	0.039773012	0.070737311
GPRASP2	-0.097538318	0.025284677	0.047458936
C7orf42	-0.008353478	0.84842219	0.894845291
C9orf163	-0.084116382	0.053853684	0.091603403
CYP11B2	-0.023206725	0.596444387	0.683349457
FCRL3	-0.156732834	0.000308101	0.000905599
PRDX1	0.145556896	0.000813358	0.002191647

FGB	0.069780031	0.109926187	0.170046824
COX17	0.387603969	2.66E-20	1.47E-18
C16orf33	0.32944958	8.81E-15	1.82E-13
PIWIL1	-0.005406223	0.901556002	0.930457848
FOLR1	-0.211141284	1.03E-06	5.06E-06
KIAA0082	0.028069198	0.520643744	0.613890389
FREQ	-0.190814825	1.05E-05	4.18E-05
TMCC2	-0.285199583	2.66E-11	2.89E-10
TCF12	0.009792166	0.822717332	0.874386481
ZNF721	0.0543975	0.212931816	0.295423677
FAM130A2	-0.178985712	3.65E-05	0.00012995
POU4F1	-0.291770337	8.81E-12	1.03E-10
SNRPF	-0.021643047	0.620421054	0.704233186
SGIP1	0.103723061	0.017330577	0.034057436
ZNF641	-0.02299324	0.598778543	0.68462524
EMG1	-0.12627737	0.003721369	0.00863824
PRRG4	0.149380405	0.000587877	0.001626116
HIRA	-0.277742304	9.01E-11	8.95E-10
MYNN	-0.260115009	1.40E-09	1.13E-08
AEBP2	-0.286517438	2.14E-11	2.34E-10
TBXA2R	-0.001489237	0.972818174	0.979508589
ISL2	-0.107670613	0.013484755	0.02718615
PCDHB11	-0.116209245	0.007632087	0.016334739
RNF144A	-0.255004508	2.98E-09	2.31E-08
5-Mar	-0.10016559	0.021585571	0.041323063
DULLARD	-0.088078511	0.043469221	0.076347398
DCLRE1B	-0.194668761	6.89E-06	2.86E-05
ITGA8	0.063344568	0.146838604	0.216837314
TP73	0.172147568	7.24E-05	0.000242046
PRKCD	0.363495174	7.11E-18	2.63E-16
NDUFB4	0.281549661	4.85E-11	4.99E-10
ATP13A4	-0.122369147	0.004948142	0.011116596
ANTXR2	-0.149467495	0.000583493	0.001615444
COL4A3	-0.172104252	7.27E-05	0.000242945
MYO10	-0.343930716	4.73E-16	1.28E-14
SLC6A18	0.31607945	1.14E-13	1.95E-12
PEX1	0.154979659	0.00036036	0.001041774
TMEM74	-0.301746503	1.56E-12	2.08E-11
RBM19	0.04837507	0.268088077	0.35813026
TAPBP	0.037818909	0.386703434	0.485868291
RUNX1	0.119441268	0.006094452	0.013398207
MID1	-0.38923544	1.79E-20	1.04E-18
GPR64	-0.245787635	1.12E-08	7.81E-08
RASEF	0.244520201	1.34E-08	9.20E-08
GABRG1	-0.016246758	0.710077177	0.782204329
MYO16	-0.006943749	0.873766746	0.911724372
DBF4	-0.14366073	0.000952715	0.002537136
TSHZ2	0.006702101	0.878124908	0.914409832
RIPK2	-0.08763879	0.044531444	0.077879365
PPTC7	0.036467315	0.403912292	0.502459373
KIF4B	-0.005873258	0.893100481	0.923629952

LRRC31	0.006114943	0.888729535	0.920984352
ZNF540	-0.072025078	0.098925235	0.155692671
EFNB3	0.02419426	0.579819214	0.667160993
LOH12CR1	-0.177452028	4.26E-05	0.00014982
STON2	0.139064065	0.001386954	0.003562397
GLP1R	-0.071895326	0.099536214	0.156374087
CSTF2T	-0.060031339	0.169204377	0.244924133
IREB2	-0.007390932	0.865711873	0.906858996
GRSF1	0.021313819	0.625749381	0.708606435
PDCD7	-0.024280997	0.578460928	0.666096675
LRRC43	-0.063680324	0.145088543	0.214665412
CNR1	-0.08062774	0.064633857	0.107269525
IL1F7	0.017754959	0.684545883	0.760482861
C12orf64	-0.059433402	0.173498784	0.249787877
FAM69B	-0.047055689	0.281374417	0.373364543
NR2E1	-0.259974623	1.42E-09	1.15E-08
MS4A6A	-0.119551443	0.006047329	0.01331365
FTL	-0.021628008	0.620664022	0.704274418
C7orf36	-0.093704014	0.031659038	0.057834647
PCLO	0.278023227	8.61E-11	8.59E-10
DYRK2	-0.007138243	0.870261768	0.909749272
ARIH2	0.029847218	0.494568708	0.589089556
SAMD7	0.013394139	0.759467222	0.823481564
SCNN1D	0.208275904	1.45E-06	6.88E-06
SLC32A1	-0.125608052	0.003909616	0.00903768
C22orf25	0.146750074	0.0007356	0.001997883
MRPS18A	-0.052786835	0.226811836	0.311031663
GPR112	0.054244881	0.215097637	0.298025094
EARS2	0.243776927	1.48E-08	1.01E-07
ERN2	0.065205938	0.135303754	0.202476022
ATPBD3	0.073	0.094429452	0.149676603
PRH2	-0.098183573	0.024328848	0.045818709
CDKN2D	-0.013868002	0.751003145	0.81631931
PGLYRP2	0.284650868	2.91E-11	3.15E-10
TRIM40	-0.059319802	0.17432376	0.25050638
SEC14L3	0.012070854	0.782400025	0.843587192
SLC22A1	-0.147778002	0.000674194	0.0018466
BTN2A3	-0.043443227	0.320000858	0.415386378
RASA4	0.058985457	0.176768772	0.253605035
CCNL2	0.034295296	0.432505366	0.530092784
MYBPC3	-0.096090611	0.027547781	0.051208367
GJA4	0.084990951	0.051399426	0.088037624
CDC42SE1	-0.058524406	0.180182024	0.257600387
TRPV2	-0.122740842	0.00481747	0.010862716
MYPN	-0.02663202	0.542224796	0.634953393
SIM1	-0.168944845	9.88E-05	0.000320955
CDADC1	0.074831942	0.086424272	0.13878894
ZFHX4	0.142815057	0.001021715	0.002705654
NIBP	0.313313876	1.91E-13	3.08E-12
ADAMTS19	0.055037752	0.207587798	0.289314907
ABTB2	-0.208398419	1.43E-06	6.79E-06

TSPYL2	0.007862049	0.857240914	0.900747501
EIF2S3	-0.130511413	0.002709079	0.006532599
SOX30	-0.143285138	0.000982811	0.002612757
AP2A1	0.302157312	1.45E-12	1.96E-11
DKFZP56400523	-0.09622845	0.027325051	0.050869433
LOC285398	-0.001758414	0.967907547	0.976445139
CDH18	0.001311656	0.976058384	0.98226924
CHL1	-0.02862666	0.512393139	0.606838484
GATS	0.045528069	0.297305746	0.391714812
TBC1D2B	-0.037032398	0.39666258	0.494742029
OR1J1	-0.005764961	0.895760047	0.925408019
GSN	-0.136655746	0.001681133	0.004231389
DPCR1	0.058571874	0.17982837	0.257154569
GARNL4	-0.052564077	0.228780994	0.313243004
SMARCA5	-0.11170028	0.010354984	0.021496555
PLEKHG3	0.043667143	0.317510588	0.412763764
ZBTB45	0.169677138	9.21E-05	0.000301416
FRMD6	0.039073189	0.371139411	0.469526354
PLS1	0.138424008	0.001460133	0.003740981
DGKZ	-0.08731613	0.045324703	0.07907922
EFNA1	0.025407502	0.560956916	0.651799712
WDR85	-0.172720149	6.84E-05	0.000230171
ANK2	-0.099431966	0.022567958	0.042962964
PAGE4	0.154702034	0.000369357	0.001065779
SENP6	-0.105872764	0.015131635	0.030177314
AKR7A2	0.232137213	7.24E-08	4.34E-07
FKBP10	0.053731889	0.218591764	0.302185422
VEGFC	0.08612517	0.048356234	0.083747246
LARP1	0.246607418	9.97E-09	7.00E-08
SRBD1	-0.056408205	0.196476468	0.276840926
ITGB6	-0.076741605	0.078670702	0.12746934
SLC1A2	0.325450326	1.92E-14	3.75E-13
INVS	-0.057169408	0.190495673	0.269711696
MPO	-0.326917671	1.45E-14	2.86E-13
MOBKL3	0.047894308	0.272878751	0.363345916
CUTL2	-0.029384384	0.501288563	0.59552229
KLK2	0.019620866	0.653452478	0.73389576
VIM	-0.150380445	0.000539331	0.00150333
REG1B	0.075954408	0.081795603	0.131872356
PCDHGC4	0.028028142	0.521254071	0.614374407
C3orf34	0.04138126	0.343529603	0.440167437
SUMO3	-0.196574179	5.57E-06	2.36E-05
CST9L	0.294276648	5.73E-12	6.95E-11
MLL4	-0.110075356	0.011529761	0.023640047
SPR	0.499827231	1.36E-34	1.04E-31
SAMD9L	-0.168200275	0.000106147	0.000343888
ABCE1	-0.196851871	5.40E-06	2.30E-05
SUPT3H	-0.089078131	0.041133661	0.072806817
ACTBL1	0.383647308	6.88E-20	3.52E-18
ADAMTS4	-0.147823203	0.000671606	0.001840332
SLIT3	0.062905572	0.14966531	0.22042922

RHEBL1	-0.144587406	0.000882065	0.002360233
NPM2	-0.291914533	8.59E-12	1.02E-10
MAN1C1	0.139667258	0.001321092	0.003411756
KIAA1856	0.176764415	4.57E-05	0.000159796
HSPA6	0.03644915	0.404146639	0.50254756
LOC388152	-0.039401046	0.367135919	0.465293129
C10orf140	-0.006555373	0.880772999	0.916391399
ZDHHC12	0.239729211	2.60E-08	1.67E-07
LIN7A	0.272362436	2.12E-10	1.97E-09
PHC2	-0.222954667	2.39E-07	1.32E-06
SPHK1	-0.164685003	0.00014827	0.00046635
TRIM26	-0.062466092	0.152536412	0.224029542
FAM83E	0.201265424	3.28E-06	1.45E-05
C18orf24	-0.164177352	0.000155516	0.000486898
ZNF578	0.042144399	0.334696157	0.430553696
ORAI1	-0.0938819	0.031335468	0.057294616
RUVBL1	-0.008972911	0.837333459	0.885427935
C7orf20	0.188917701	1.29E-05	5.05E-05
APAF1	-0.048533812	0.266518988	0.356343826
SLC36A4	-0.340291186	1.00E-15	2.52E-14
MYH11	0.094415514	0.030381758	0.05579971
NEK1	-0.008829147	0.839904252	0.887688423
MPP2	0.259026298	1.64E-09	1.32E-08
C12orf24	-0.106961231	0.014114694	0.028335375
TNK2	0.159130257	0.000248021	0.000741049
ZNF289	0.08163783	0.061343975	0.102541739
MATN3	0.211090128	1.03E-06	5.09E-06
IFNGR2	-0.186685511	1.64E-05	6.25E-05
ITPR1	0.119133155	0.006227992	0.013657603
EBF3	0.003302501	0.939767693	0.957043979
TBC1D20	0.253646605	3.63E-09	2.77E-08
OR10P1	0.156980557	0.000301315	0.00088735
DDAH2	0.129341492	0.002960188	0.007063328
SHPRH	-0.197785658	4.86E-06	2.09E-05
STX7	-0.192912231	8.36E-06	3.39E-05
LOC554248	-0.167910906	0.000109136	0.000353198
BCAR1	0.280598515	5.67E-11	5.79E-10
ATXN3	-0.10657534	0.01446813	0.028959808
TRIM27	0.024209361	0.579582629	0.667138448
CDC42EP2	-0.064872898	0.137314656	0.20488906
CHP	0.104583033	0.016419439	0.032443165
SOX17	0.08232425	0.059188169	0.099602642
ZNF259	-0.208676059	1.38E-06	6.60E-06
CHCHD1	0.15032525	0.00054191	0.001509835
ZDHHC19	-0.055832635	0.201088966	0.282111807
GBP2	-0.061428569	0.159480083	0.232655523
GARNL3	-0.015270685	0.726777328	0.795895599
MRC2	0.067716148	0.120869345	0.184103444
C1orf52	-0.230745219	8.71E-08	5.13E-07
AOF2	-0.290276227	1.13E-11	1.31E-10
LRPPRC	-0.056211012	0.198047952	0.278480872

ACVR1C	-0.095315009	0.028830384	0.053220664
TM4SF18	-0.232423382	6.97E-08	4.18E-07
TMEM169	-0.093064034	0.03284688	0.059685422
PPP1R16A	0.194674058	6.88E-06	2.86E-05
EBF1	0.070278636	0.107403249	0.166773378
RRS1	0.063522828	0.145702472	0.215418249
SNX2	0.097884868	0.024767372	0.046616031
OR2T2	-0.159325012	0.000243655	0.00072836
RBX1	-0.056978213	0.191985174	0.271528543
ANKRD54	0.084981246	0.05142614	0.088058852
TSNAX	0.048460257	0.267245259	0.357159551
TMEM83	0.209038284	1.32E-06	6.36E-06
ZBTB7A	0.365366375	4.68E-18	1.80E-16
ATM	-0.204454434	2.27E-06	1.03E-05
LOC338328	0.101454873	0.019948863	0.038477278
TIE1	0.101607334	0.019762651	0.038230861
HIST1H3G	0.15253358	0.000447179	0.001265396
PASD1	0.009472536	0.828412775	0.878714879
TINAG	0.064035605	0.142471734	0.211353122
PCDHAC2	0.136050614	0.001763547	0.004413516
LRRC15	0.095546633	0.028442087	0.0525512
WBSCR17	-0.0843822	0.053097619	0.090492589
TFF2	-0.124490669	0.004243178	0.00973193
PARP2	-0.07802159	0.073796974	0.12068553
NDFIP2	-0.040600101	0.352723944	0.450747641
PCDHGB2	0.128088119	0.003252553	0.007680471
WDR60	0.129067747	0.003021921	0.007191096
MAP7D2	-0.154749502	0.000367804	0.001061796
USP45	-0.203823772	2.44E-06	1.11E-05
GSDML	0.010397483	0.811956967	0.866641797
TNS1	-0.12658989	0.003636318	0.0084728
PLCD4	0.291384466	9.40E-12	1.10E-10
IQCD	0.366513041	3.62E-18	1.43E-16
SMPX	-0.266582669	5.22E-10	4.57E-09
CD9	0.03737235	0.392339166	0.49114282
SRGN	-0.180383672	3.16E-05	0.000113614
CASP7	0.205032132	2.12E-06	9.75E-06
INOC1	0.082670886	0.058123563	0.098052618
DKFZp451M2119	-0.00467442	0.91482713	0.940209263
VMAC	0.022200224	0.611448741	0.69574358
USP53	0.046755331	0.284460277	0.376971173
CAMK1G	-0.060111412	0.168635383	0.244157986
TMEM106A	-0.078726531	0.071219717	0.116968494
CDC20	-0.191275295	1.00E-05	3.99E-05
ACSL5	-0.242626353	1.74E-08	1.17E-07
CBWD5	-0.010335829	0.813051382	0.867207797
C1orf87	-0.006876618	0.874977086	0.912193797
KIAA1274	-0.125470507	0.003949353	0.009119254
PRUNE2	-0.120685135	0.00558111	0.012375855
LYPLA2	0.011317777	0.795665744	0.853996973
DOK6	0.024978943	0.567585816	0.656895386

GPR149	0.007404791	0.865462447	0.906752187
FAM30A	-0.186312709	1.70E-05	6.49E-05
TMEM129	0.171271225	7.88E-05	0.000261746
SLC35B3	-0.111806469	0.010282047	0.021359564
ACPP	0.126618001	0.003628755	0.008458382
LOC200261	0.052555447	0.229748768	0.31414836
SLC4A7	0.208743286	1.37E-06	6.56E-06
CCDC40	0.258855133	1.68E-09	1.36E-08
GART	-0.300954262	1.79E-12	2.37E-11
THOP1	0.066372025	0.12844134	0.19371739
SCARB1	0.063550201	0.14552861	0.215212945
CACNA1F	0.14120441	0.001166046	0.00304718
TRIAP1	-0.040484574	0.354096771	0.451918025
SYT14L	0.039347647	0.373333934	0.471519769
SFRS8	0.17598512	4.94E-05	0.000172033
PBOV1	0.077217484	0.076829078	0.124974501
GOLSYN	0.353161008	6.77E-17	2.14E-15
GJB7	0.035157328	0.421019993	0.519849786
CAMK2N1	0.192090988	9.15E-06	3.68E-05
GREM1	0.071758072	0.100185779	0.157274024
FLJ20433	0.117028677	0.007212519	0.015533078
QPCT	-0.16980113	9.10E-05	0.000298135
PRKAG2	-0.156503991	0.000314496	0.000923072
H2AFX	0.01982083	0.650154414	0.731527812
C6orf154	0.3516447	9.36E-17	2.89E-15
PLOD3	-0.01643494	0.706873094	0.779512671
ZBTB39	-0.117569386	0.00694709	0.015041428
WASF3	-0.313211672	1.95E-13	3.12E-12
DRG1	-0.245675208	1.14E-08	7.91E-08
PRR4	-0.275868901	1.22E-10	1.18E-09
SPCS1	0.262774501	9.35E-10	7.79E-09
KDELR3	-0.1451741	0.000839872	0.002257157
SRP19	0.12350719	0.004557836	0.010341748
GABRA6	0.016102751	0.712532578	0.784206698
MFSD1	-0.12346128	0.004573027	0.010372388
MMEL1	0.361475285	1.11E-17	3.93E-16
PDXDC2	0.095006951	0.02935386	0.054122005
BUB1	-0.134141598	0.002048412	0.005050394
RNF138	-0.482949884	4.33E-32	1.90E-29
MYLPF	0.118014424	0.006735225	0.014639412
AIF1	-0.17258397	6.93E-05	0.000233023
DYNLRB1	0.369780967	1.73E-18	7.19E-17
HCN3	0.140794217	0.001205691	0.003144103
HIST1H2AI	0.382868768	8.28E-20	4.17E-18
MAP4K5	-0.093164543	0.032657848	0.059359477
LASP1	0.297437551	3.32E-12	4.11E-11
LOC130951	0.132965969	0.002244178	0.005504367
PLAA	-0.137937965	0.001518055	0.003867392
KRT6A	-0.268964079	3.61E-10	3.26E-09
C6orf117	-0.283999146	3.24E-11	3.45E-10
ARHGAP23	0.006840929	0.875738546	0.912543013

PTF1A	-0.088375689	0.042962266	0.075543315
GPHA2	0.107331779	0.013782485	0.027738264
LCE3B	0.130302179	0.002752512	0.006615924
MCL1	-0.113008134	0.0094879	0.019850663
EHBP1	-0.210139246	1.16E-06	5.62E-06
PRNP	-0.25505209	2.96E-09	2.29E-08
ZSCAN1	0.130948763	0.002620304	0.006338415
C1orf113	0.179220646	3.56E-05	0.000127167
FOXA3	-0.060598953	0.165201788	0.239921066
NEB	-0.098779325	0.023474238	0.044467989
ASGR1	-0.21194566	9.33E-07	4.63E-06
CTGF	-0.061348845	0.160023346	0.233116218
RAB17	0.378664319	2.23E-19	1.05E-17
MST101	0.099527851	0.022437397	0.042767376
JARID1B	0.079451664	0.068645876	0.113225185
USP37	0.011223944	0.797322854	0.855327675
PTBP1	0.093501597	0.0320307	0.058394317
PTPN7	-0.180852123	3.01E-05	0.00010865
CDC7	-0.274737361	1.46E-10	1.39E-09
SNX7	-0.124184699	0.004338899	0.009907127
ZNF335	0.19698765	5.32E-06	2.27E-05
CPT2	0.129307687	0.002967749	0.007078622
HEATR1	-0.331302152	6.11E-15	1.31E-13
HSPC152	0.276965207	1.02E-10	1.00E-09
C5orf40	-0.053019995	0.224763672	0.308911895
PSME1	0.29248135	7.80E-12	9.28E-11
STAG3	-0.298682263	2.67E-12	3.39E-11
TMEM154	-0.035413008	0.417648044	0.516581391
KLHL32	-0.048368062	0.268157498	0.35814519
TSGA10IP	-0.041795441	0.338717127	0.434635145
SUV420H2	0.211983004	9.29E-07	4.62E-06
SF1	0.014003245	0.748656216	0.814344078
