# Supplementary material for: Evaluating the expression of heat shock protein 27 and topoisomerase II α in a retrospective cohort of patients diagnosed with locally advanced breast cancer and treated with neoadjuvant anthracycline-based chemotherapies
Source: Front Oncol. 2023 Aug 15;13:1067179. doi: 10.3389/fonc.2023.1067179 (PMC10478710; doi:10.3389/fonc.2023.1067179)
Supplement: Supplementary file 3 [file Table_2.pdf]

Supplementary Table 2 Enrichment analyses of KEGG pathway based on Hsp27-related genes with correlation coefficients greater than 0.3 or less than -0.3

Pathway	Total	Expected	Hits	P.Value	FDR
Metabolic pathways	1430	34	47	0.0102	0.755
cAMP signaling pathway	212	5.04	10	0.0296	0.836
Oxytocin signaling pathway	153	3.64	9	0.0106	0.755
Estrogen signaling pathway	138	3.28	14	4.52E-06	0.0014
Insulin resistance	108	2.57	7	0.0143	0.755
Inflammatory mediator regulation of TRP channels	100	2.38	6	0.0316	0.836
Choline metabolism in cancer	99	2.35	6	0.0302	0.836
Longevity regulating pathway	89	2.12	6	0.019	0.836
VEGF signaling pathway	59	1.4	5	0.0128	0.755
Regulation of lipolysis in adipocytes	55	1.31	4	0.0412	0.9
Ovarian steroidogenesis	49	1.17	5	0.00589	0.755
Endocrine and other factor-regulated calcium reabsorption	48	1.14	4	0.0267	0.836
Ether lipid metabolism	47	1.12	4	0.0249	0.836
Galactose metabolism	31	0.737	3	0.0365	0.893
Glycosaminoglycan biosynthesis - keratan sulfate	14	0.333	2	0.0424	0.9
