# Supplementary material for: Evaluating the expression of heat shock protein 27 and topoisomerase II α in a retrospective cohort of patients diagnosed with locally advanced breast cancer and treated with neoadjuvant anthracycline-based chemotherapies
Source: Front Oncol. 2023 Aug 15;13:1067179. doi: 10.3389/fonc.2023.1067179 (PMC10478710; doi:10.3389/fonc.2023.1067179)
Supplement: Supplementary file 4 [file Table_3.pdf]

Supplementary Table 3 Related biological processes through enrichment analyses of GO database based on Hsp27-related genes with correlation coefficients greater than 0.3 or less than -0.3

Pathway	Total	Expected	Hits	P.Value	FDR
Sexual reproduction	645	14.6	23	0.0216	1
Gamete generation	543	12.3	20	0.023	1
Anion transport	374	8.48	16	0.0115	1
Response to extracellular stimulus	320	7.26	13	0.0311	1
Response to nutrient levels	295	6.69	12	0.0371	1
Lipid transport	268	6.08	13	0.0083	1
I_kappaB kinase/NF_kappaB cascade	246	5.58	12	0.0105	1
Organic acid transport	246	5.58	11	0.0249	1
Carboxylic acid transport	246	5.58	11	0.0249	1
Cell projection assembly	229	5.19	11	0.0156	1
Regulation of I_kappaB kinase/NF_kappaB cascade	210	4.76	11	0.0085	1
Developmental maturation	177	4.01	9	0.0197	1
Epidermal growth factor receptor signaling pathway	167	3.79	9	0.014	1
Actin polymerization or depolymerization	136	3.08	7	0.0354	1
Digestion	132	2.99	8	0.0104	1
Monocarboxylic acid transport	103	2.34	7	0.009	1
Vesicle localization	82	1.86	6	0.0107	1
Cytoskeleton_dependent intracellular transport	76	1.72	5	0.029	1
Activation of phospholipase C activity	56	1.27	4	0.0379	1
One_carbon metabolic process	36	0.816	3	0.0476	1
Cofactor catabolic process	16	0.363	2	0.0499	1
