# Supplementary material for: Evaluating the expression of heat shock protein 27 and topoisomerase II α in a retrospective cohort of patients diagnosed with locally advanced breast cancer and treated with neoadjuvant anthracycline-based chemotherapies
Source: Front Oncol. 2023 Aug 15;13:1067179. doi: 10.3389/fonc.2023.1067179 (PMC10478710; doi:10.3389/fonc.2023.1067179)
Supplement: Supplementary file 5 [file Table_4.pdf]

Supplementary Table 4 Related cellular components through enrichment analyses of GO database based on Hsp27-related genes with correlation coefficients greater than 0.3 or less than -0.3

Pathway	Total	Expected	Hits	P.Value	FDR
Cytosol	2660	53.7	69	0.015	0.775
Vesicle	1210	24.4	34	0.0305	0.775
Cytoplasmic vesicle	1110	22.4	32	0.0259	0.775
Neuron projection	685	13.8	22	0.0216	0.775
Cell body	290	5.85	13	0.00615	0.775
Apical part of cell	289	5.83	12	0.0146	0.775
Apical plasma membrane	216	4.35	10	0.0124	0.775
Nuclear chromatin	159	3.2	7	0.0424	0.895
Microtubule associated complex	141	2.84	7	0.0242	0.775
Lamellipodium	127	2.56	6	0.0437	0.895
Brush border	59	1.19	4	0.031	0.775
