# Supplementary material for: Evaluating the expression of heat shock protein 27 and topoisomerase II α in a retrospective cohort of patients diagnosed with locally advanced breast cancer and treated with neoadjuvant anthracycline-based chemotherapies
Source: Front Oncol. 2023 Aug 15;13:1067179. doi: 10.3389/fonc.2023.1067179 (PMC10478710; doi:10.3389/fonc.2023.1067179)
Supplement: Supplementary file 6 [file Table_5.pdf]

Supplementary Table 5 Related molecular functions through enrichment analyses of GO database based on Hsp27-related genes with correlation coefficients greater than 0.3 or less than -0.3

Pathway	Total	Expected	Hits	P.Value	FDR
Purine nucleotide binding	1900	40.8	52	0.0376	0.627
Purine ribonucleotide binding	1890	40.7	52	0.0358	0.627
Adenyl ribonucleotide binding	1530	32.8	45	0.0173	0.574
Adenyl nucleotide binding	1530	32.9	45	0.018	0.574
ATP binding	1490	32	45	0.0115	0.574
Enzyme binding	1200	25.8	43	0.000592	0.212
Cytoskeletal protein binding	738	15.8	26	0.00916	0.574
Ligase activity	572	12.3	21	0.012	0.574
Kinase binding	418	8.98	18	0.00412	0.32
Ligase activity, forming carbon_nitrogen bonds	393	8.44	15	0.023	0.574
Protein kinase binding	376	8.08	17	0.00322	0.312
Actin binding	373	8.01	14	0.031	0.627
Acid_amino acid ligase activity	349	7.5	13	0.0388	0.627
Small conjugating protein ligase activity	313	6.72	12	0.0383	0.627
Small conjugating protein ligase activity	313	6.72	12	0.0383	0.627
Ubiquitin_protein ligase activity	291	6.25	12	0.0237	0.574
GTPase binding	150	3.22	7	0.0432	0.659
Kinase regulator activity	144	3.09	10	0.0011	0.212
Small GTPase binding	138	2.96	7	0.0294	0.627
Heparin binding	130	2.79	7	0.0221	0.574
Ras GTPase binding	125	2.68	7	0.0183	0.574
Protein kinase regulator activity	123	2.64	6	0.0494	0.659
Oxidoreductase activity, acting on the CH_CH gr	80	1.72	7	0.00164	0.212
Microtubule motor activity	75	1.61	5	0.0225	0.574
Actin filament binding	71	1.52	5	0.0182	0.574
Ubiquitin binding	63	1.35	4	0.0463	0.659
Carbonate dehydratase activity	14	0.301	2	0.0353	0.627
